# Designing Safer Functionalized Ionic Liquids: Monoterpene-Derived Compounds for Greener Agrochemical Development

**DOI:** 10.1021/acs.jafc.5c12979

**Published:** 2026-02-02

**Authors:** Przemysław Pietrusiak, Barbara Pawłowska, Magdalena Bendová, Robert Biczak, Joanna Feder-Kubis

**Affiliations:** † Faculty of Chemistry, Wrocław University of Science and Technology, Wybrzeże Wyspiańskiego 27, Wrocław 50-370, Poland; ‡ Faculty of Science and Technology, 69695 Jan Długosz University in Częstochowa, 13/15 Armii Krajowej Av., Częstochowa 42-200, Poland; § Department of Physical Chemistry, Faculty of Chemical Engineering, 52735 University of Chemistry and Technology, Prague, Technická 5, 166 28 Prague 6, Czech Republic; ∥ Faculty of Chemistry, Technische Universität Dresden, Bergstrasse 66, Dresden 01062, Germany

**Keywords:** structure–activity relationships in ionic liquids, terrestrial higher plants, growth inhibition, soil phytotoxicity, photosynthetic pigments, antioxidant enzymes

## Abstract

The environmental behavior and phytotoxicity of functionalized ionic liquids (FILs) featuring imidazolium cations bearing a cyclic (−)-menthol or a linear 1-decanol-derived substituent and fluorinated anions were evaluated in a soil bioassay (*Raphanus sativus*). Structure–activity relationships were determined by integrating measurements (water solubility, surface tension, molecular volume) with in-silico descriptors (*n*-octanol–water partition coefficient, log *K*_OW_; bioconcentration factor, log BCF; asymmetry). Lipophilicity and bioaccumulation correlated with fresh-mass inhibition (EC_50_). More-fluorinated, larger, and charge-delocalized anions were more hydrophobic, elevating bioaccumulation and toxicity. Within menthol-based series, sulfonylimide-based FILs were more phytotoxic (EC_50_ 74.7, 107.3 mg·kg^–1^ soil dry weight) than sulfonate analogues (e.g., 299.1 mg·kg^–1^). FILs with linear cations were more phytotoxic and showed higher log *K*_OW_/BCF than cyclic analogues, consistent with greater molecular volume, higher asymmetry, and reduced hydration. Antioxidant responses paralleled EC_50_, implicating oxidative stress. This workflow improves risk assessment and provides green-by-design criteria to prioritise safer ILs.

## Graphical abstract

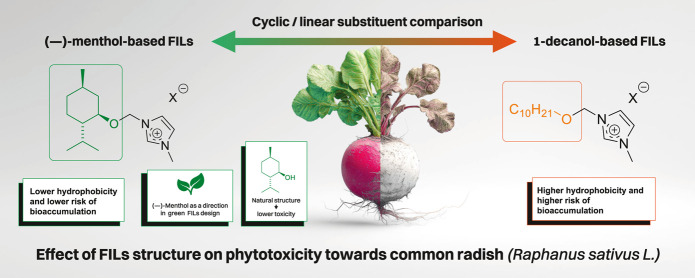

## 1 Introduction

The increasing use of ionic liquids (ILs) has raised concerns about their potential environmental impact, particularly their persistence and toxicity after use. Although their tunable physicochemical properties have driven applications across chemistry, materials science, and various industrial sectors, their long-term environmental behavior remains insufficiently understood. By varying anions and cations, ILs can be synthesized with tailored properties suited to specific applications.^1,2^ However, this structural versatility also influences their fate and ecotoxicity in ecosystems, necessitating careful evaluation to ensure that new ILs align with the principles of green chemistry and cleaner production through hazard reduction and improved environmental safety. Consequently, research into the toxic effects of ILs has become a crucial aspect of IL chemistry and an essential step toward developing less toxic and more environmentally compatible compounds.^3–7^ Over the years, studies have generated extensive data on the toxicity of ILs, including their effects on aquatic ecosystems, microorganisms, invertebrates and vertebrates, as well as their potential for enzyme inhibition, cytotoxicity and phytotoxicity.^8,9^

Among the various toxicological aspects of ILs, phytotoxicity is particularly relevant because plants are primary producers and play a central role in both ecosystem functioning and agricultural productivity. Adverse effects on plants can influence crop yields and quality, while also triggering cascading impacts on soil microbiota, nutrient cycling, and biodiversity. Several structural elements of ILs contribute to their phytotoxicity, affecting plant growth and ecosystem stability.

Phytotoxicity assessment of ILs can generally be approached from two different perspectives. The first focuses on the development of herbicidal ionic liquids (HILs),^10–12^ where phytotoxicity is not considered a side effect, but rather a deliberately introduced functional property to enhance agrochemical performance. These salts reduce the need for additives and provide excellent herbicidal performance at lower doses, which has driven research into herbicidal ionic architectures. HILs are defined as salts in which at least one of the constituent ions contains a structural motif characteristic of conventional herbicides.^13–16^ Their use in the agrochemical industry is particularly advantageous due to improved bioavailability and uptake of active substances.^17^ Recent studies have highlighted the potential of bio-HILs incorporating naturally derived components such as choline, d-glucose, geranic acid, and oils from coconut and rapeseed.^18–20^

The second approach addresses various ILs that are widely used in research and industrial contexts, for which phytotoxicity is not a target property, but an unintended outcome that must be considered within an environmental safety framework. In this case, phytotoxic effects constitute an important element of environmental compatibility assessment, as they reflect the level of potential risk posed by the applied compounds to primary producers and ecosystem functioning. Clear identification and classification of such effects are therefore necessary to determine hazard levels, to contextualise the use of ILs across different applications, and to support informed decisions regarding exposure levels and concentration ranges required for environmentally balanced processes.

It should be noted that, irrespective of the perspective adopted, the relationship between molecular structure and phytotoxicity remains a central issue in both approaches outlined above. Key factors include the alkyl chain length, the cationic core structure, the anion type, and functionalization. Understanding how these parameters influence phytotoxicity is essential not only for environmental hazard identification and risk assessment, but also for but also for contextualising the safe use of ILs across different applications within the framework of environmental compatibility and cleaner production. However, to the best of our knowledge, not all key structural parameters have been extensively studied in the literature.

### 1.1 Influence of the Alkyl Chain Length on IL Phytotoxicity

Many research articles have investigated the influence of the alkyl chain length in IL cations on the phytotoxicity of ILs.^21^ Studzińska et al.^22^ evaluated the toxicity of 1-ethyl-3-methylimidazolium chloride, 1-butyl-3-methylimidazolium chloride and 1-hexyl-3-methylimidazolium chloride against watercress (*Lepidium sativum* L.) under hydroponic conditions. Similarly, Larson et al.^23^ investigated the toxicity of other ILs, including 1-octyl-3-methylimidazolium bromide, 1-hexyl-3-methylimidazolium bromide, 1-butyl-3-methylimidazolium bromide, 1-butyl-3-methylpyridinium bromide and tetrabutylammonium bromide against duckweed (*Lemna minor* L.), also under hydroponic conditions. Both studies concluded that IL phytotoxicity increased with alkyl chain length. These studies refer to aqueous systems, in which IL phytotoxicity typically increases with chain length. By contrast, soil-based studies often show a different pattern, indicating that the phytotoxicity of ILs does not consistently increase with alkyl chain length. In soil-based experiments, ILs with shorter substituents containing three to four carbon atoms were more toxic, due to greater mobility, faster transport in the soil solution, and enhanced contact with plant roots. Conversely, longer alkyl chain ILs tend to adsorb to soil colloids, reducing their availability to plants and thus their phytotoxicity.^24,25^ In hydroponic systems, soil properties such as sorption capacity, pH, humus content and interactions with other soil components are not considered,^21^ which partly explains the differences observed between aqueous and soil-based assays. These matrix-dependent effects indicate that careful optimization of alkyl chain length could be used to reduce plant toxicity in environmental exposure scenarios, in line with cleaner production principles.

### 1.2 Influence of the Cation Core Structure on IL Phytotoxicity

A study from our group evaluated cation core variation, testing 1-butyl-1-methylpyrrolidinium, 1-butyl-1-methylpiperidinium and 1-butyl-4-methylpyridinium hexafluorophosphates for their effects on spring barley (*Hordeum vulgare* L.) seedlings and common radish (*Raphanus sativus* L. *radicula* Pers.) leaves.^26^ The results indicated that radish was more resistant to the tested ILs than spring barley. Differences in cation core structure did not significantly affect phytotoxicity in either species. In this data set, the cation core (amine type) had little influence on phytotoxicity in either species. However, comparative studies of this kind are rare, and it is possible that more pronounced differences could emerge with a larger, more diverse data set. Expanding the scope of this research could improve our ability to identify cation cores that minimize plant toxicity. This would support the assessment and classification of ILs with respect to their environmental impact, in line with cleaner production principles.

### 1.3 Influence of the Anion Structure on IL Phytotoxicity

Chen et al.^27^ investigated the influence of different anions on the growth and development of wheat (*Triticum aestivum* Liao Chun No. 18) seeds, focusing on the widely used cation 1-butyl-3-methylimidazolium. In their study, the phytotoxic effects of 1,3-dialkylimidazolium salts were evaluated with the following anions: alaninate [Ala]^−^, lactate [Lac]^−^, tetrafluoroborate [BF_4_]^−^, chloride [Cl]^−^ and trifluoromethanesulfonate [OTF]^−^. Their results demonstrated that the anion structure significantly affects the phytotoxicity of imidazolium salts, in order of highest phytotoxicity, as follows: [C_4_-Im-C_1_]­[OTF] > [C_4_-Im-C_1_]­[Cl] > [C_4_-Im-C_1_]­[BF_4_] > [C_4_-Im-C_1_]­[Lact] > [C_4_-Im-C_1_]­[Ala]. The high toxicity of the trifluoromethanesulfonate anion was attributed to its significant chemical stability. This stability potentially renders sulfonate-containing compounds resistant to both biological and abiotic degradation, thereby increasing their bioaccumulation potential in various algae and plants. Further analysis led to the conclusion that the incorporation of natural amino acid groups into the anion moiety of ILs reduces toxicity. This finding highlights the importance of using naturally derived components when designing ILs with improved environmental profiles. The influence of common anions, specifically nitrate [NO_3_]^−^, bromide [Br]^−^ and tetrafluoroborate [BF_4_]^−^, was also evaluated in a study using another widely used cation, 1-hexyl-3-methylimidazolium.^28^ The results showed that 1-hexyl-3-methylimidazolium nitrate [C_6_-Im-C_1_]­[NO_3_] was the least toxic, while 1-hexyl-3-methylimidazolium bromide [C_6_-Im-C_1_]­[Br] and 1-hexyl-3-methylimidazolium tetrafluoroborate [C_6_-Im-C_1_]­[BF_4_] were more toxic to *Vicia faba* L. These results confirm that anion structure and alkyl chain length are key factors determining IL phytotoxicity, while the cation core plays a lesser role. However, such conclusions are still based on a limited number of comparative studies, and broader data sets could reveal specific cation–anion combinations that further minimize plant toxicity. Prioritising anions with lower environmental persistence and incorporating naturally derived functionalities could therefore be an effective strategy for developing ILs for agrochemical applications with reduced ecological impact, in line with cleaner production principles. It is also important to note that phytotoxicity outcomes are strongly influenced by the plant species tested and the IL concentrations applied,^26,29,30^ underscoring the need for standardized protocols when comparing data across studies.

### 1.4 Influence of the Functional Component Structure on IL Phytotoxicity

A fundamental aspect of modern compound design is the tailoring of molecular structures to achieve specific functional properties. This approach has been extensively applied to ILs, where the deliberate incorporation of functional groups covalently bonded to the cation and/or anion has led to the development of functionalized ionic liquids (FILs), also referred to as task-specific ionic liquids (TSILs).^31^ These organic ionic compounds represent an emerging class of specialized compounds with broad and growing application potential,^32,33^ and, given this diversity of uses, their environmental profile—including phytotoxicity—should be considered an integral part of their overall environmental characterization and hazard identification. Although the number of publications addressing the synthesis, physicochemical characterization and application-oriented testing of FILs in various fields has increased significantly, a closer examination of the literature reveals that phytotoxicity assessment of truly functionalized ionic structures—understood as systems with a functional group covalently incorporated into the cationic or anionic framework—remains relatively uncommon.

In the majority of cases reported, the phytotoxicity effects are associated with the anionic moiety, which is typically introduced *via* a straightforward metathesis reaction.^34,35^ Alternatively, another common strategy is to modify simple ionic structures, such as choline or betaine, by introducing hydrophobic alkyl chains, where the functional group—in these cases a hydroxyl or ester moiety—is already present in the parent molecules.^36^

To date, the phytotoxic properties of FILs have mainly been investigated with respect to their basic structural components, such as the alkyl chain (Scheme 1, block A),^37^ and the anion,^38,39^ similar to studies on nonfunctionalized ILs. Kaczmarek et al.^37^ investigated the effect of the alkyl chain substituent attached to the quaternary nitrogen atom in a series of betaine-based ILs (Scheme 1, block A). The authors reported that the elongation of one alkyl substituent from methyl (−CH_3_) through butyl (−C_4_H_9_) to dodecyl (−C_12_H_25_), corresponding to betaine, butylbetaine, and dodecylbetaine resulted in increased shoot and root growth in *L. sativum* L. This inverse relationship between alkyl chain length and phytotoxicity was attributed to enhanced hydrophobicity, which reduced compound uptake by plant tissues and thereby diminished toxic effects. However, this trend was only observed for salts containing linear, carboxylate anions, such as glycolate, *d* -gluconate, and α-ketoglutarate.

**Scheme 1:**
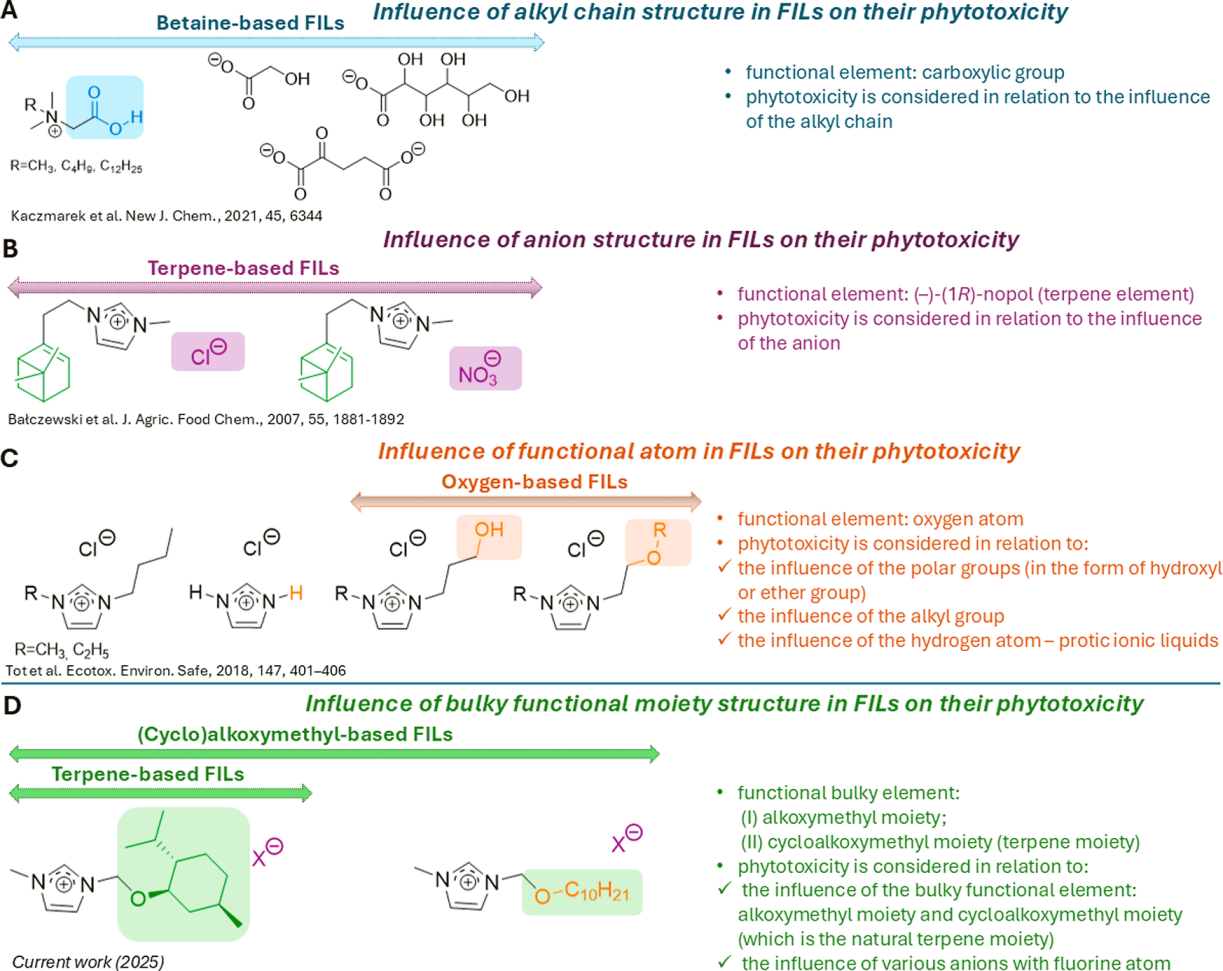
Structural Aspects Considered in Phytotoxicity Studies of FILs

One of the few studies on the phytotoxicity of FILs with naturally occurring functionalized moieties was carried out by our research group and concerned anion-dependent phytotoxicity (Scheme 1, block B).^39^ In this work, the optically active terpene (−)-(1*R*)-nopol (monoterpene) was selected as a renewable, chiral functionalized moiety to synthesize a series of FILs with different anions. Some of these were then used in the model Diels–Alder reaction involving cyclopentadiene and ethyl acrylate. From this group of FILs, two salts were selected for phytotoxicity studies: (−)-1-[(1*R*)-nopyl]-3-methylimidazolium chloride and (−)-1-[(1*R*)-nopyl]-3-methylimidazolium nitrate. These salts were tested on common radish and spring barley and the toxic effect varied with concentration. A significant reduction in germination and growth was observed at a minimum concentration of 100 mg·kg^–1^ soil dry weight (DW). Spring barley showed a greater tolerance to the tested FILs at 200 mg·kg^–1^ soil DW compared to common radish, which was affected at 100 mg·kg^–1^ soil DW. These findings confirm that the anion plays a decisive role in modulating the phytotoxicity of FILs containing monoterpene-derived cations, and illustrate the importance of anion-dependent effects in the environmental hazard assessment of FILs. The nitrate-based FIL did not inhibit germination at any of the tested concentrations, whereas the chloride-based FIL resulted in a marked reduction in germination rates, particularly in radish. In addition, at 100 mg·kg^–1^ soil DW, chloride reduced the fresh weight (FW) yield of the radish crop by approximately 21%, whereas nitrate caused a 39% reduction, indicating that although nitrate did not inhibit germination, it still had a significant effect on early plant development. These results provide clear evidence that chloride may have a greater inhibitory effect on germination, while nitrate has a greater effect on biomass accumulation at the seedling stage. This highlights the need to consider specific anion–plant interactions when assessing phytotoxicity, rather than attributing toxicity solely to the presence of a functionalized cationic moiety. *N*-Methylimidazole was chosen as the reference compound for the phytotoxicity comparison. However, we believe that the comparison of phytotoxicity with the parent heterocyclic amine alone is insufficient for the evaluation of FILs. Most importantly, this study did not assess the specific effect of the functionalized terpene group on toxicity, as no direct comparison was made with nonfunctionalized classic quaternary imidazolium salts. A more informative benchmark would involve quaternary imidazolium salts containing a linear alkyl chain in place of the terpene group, enabling a direct assessment of the impact of functionalization on phytotoxicity within a structure–effect environmental hazard assessment framework.

It should be noted that the phytotoxicity of FILs has rarely been analyzed with an explicit reference to the introduced functional structural groups. Most studies do not assess the direct effects of specific functional moieties, which may hinder a comprehensive understanding of their environmental behavior. However, such an assessment is crucial as the number of FILs is rapidly increasing and potential applications are actively being explored.^31,32^ Accordingly, the environmental profile of FILs should be rigorously investigated and controlled, not only to ensure their suitability for specific applications, but also to comply with the principles of sustainable development.

Even through research specifically focusing on the phytotoxic effects of covalently attached functional groups in FILs remains limited, one of the few exceptions being the study by Tot et al.,^29^ which investigated the phytotoxicity of FILs modified with oxygen-based polar groups—namely hydroxyl (−OH) and ether (−O−) moieties—in the alkyl side chains (Scheme 1, block C). These structural modifications were introduced to evaluate their effect on toxicity toward wheat and barley, two economically important crops. The incorporation of oxygen into the alkyl chain resulted in reduced lipophilicity of the imidazolium cation, which translated into reduced phytotoxicity compared to nonfunctionalized analogues. Notably, hydroxyl-functionalized FILs exhibited even lower toxicity than their ether counterparts, probably due to the higher polarity of the −OH group. While the study by Tot et al.^29^ represents an important step in understanding the role of functional atoms, the literature remains remarkably sparse on the influence of more complex functionalized groups beyond single atom substitutions. Particular attention should be given to covalently bound, structurally elaborate moieties—especially those derived from renewable bioresources, such as monoterpenes—because they may profoundly influence both the biological activity and the environmental profile of FILs, enabling a more comprehensive hazard-oriented assessment of functionalized ionic compounds.

In the present study (Scheme 1, block D), we address this research need by investigating the phytotoxicity of two novel series of fluorine-based FILs. Both series, synthesized and structurally engineered by our group, share a common structural feature: a bulky functional element of ten carbon atoms attached to the imidazolium ring *via* a methoxy linker (−OCH_2_−). One series contains a linear alkoxymethyl chain derived from 1-decanol, whereas the other incorporates a cycloalkoxymethyl substituent based on (1*R*,2*S*,5*R*)-(−)-menthol, a naturally occurring monoterpene obtained from renewable bioresources. This structural divergence—linear *vs* cyclic—provides an opportunity to explore the influence of functional substituent architecture on phytotoxic effects. The cyclic derivatives introduce a rigid, bulky and chiral functional motif, enabling a direct comparison between linear and cyclic functionalization while maintaining the same cationic core and linker architecture.

Our investigation evaluates the impact of these functional groups on the phytotoxicity toward *R. sativus*, used here as a sensitive and well-established model species for hazard-oriented phytotoxicity assessment, providing one of the first detailed insights into how bulky, covalently bound functional moieties influence the ecological profile of FILs. This approach aims to determine whether covalent functionalization modulates phytotoxic responses and contributes to the environmental hazard profile of FILs, incorporating structurally defined functional components, including biobased moieties. We expect the correlations and analytical insights presented to support a more systematic interpretation of structure–phytotoxicity relationships in FILs, providing a scientifically grounded framework for environmental safety assessment, concentration management, and informed use of FILs, in contexts where environmental compatibility is a critical consideration.

## 2 Materials and Methods

### 2.1 Instruments and Chemicals

All materials used for synthesis and details of the instrumentation are provided in the Supporting Information. The structure and purity of all obtained salts were confirmed by spectral and analytical methods. Characterization included ^1^H, ^13^C and ^19^F NMR spectroscopy, FTIR spectroscopy, elemental analysis, and high-resolution mass spectrometry (HRMS) in both positive and negative ionization modes. A comprehensive description of the analytical procedures is available in the Supporting Information, including measurements of specific rotation, solubility, surface tension, refractive index, thermal behavior (differential scanning calorimetry, DSC), thermal stability (thermogravimetric analysis, TGA), in silico prediction of *n*-octanol–water partition coefficient (log *K*_OW_) and bioconcentration factor (log BCF), and calculated molecular properties. A detailed description of NMR chemical shifts for the obtained FILs, including their dependence on structure and anion type, is provided in the Supporting Information. The Supporting Information also contains refractive index data analysis with temperature dependence, representative TGA and DSC thermograms, as well as all recorded FTIR and NMR spectra for the synthesized FILs.

### 2.2 General Procedure for Synthesis of FILs

#### 2.2.1 Synthesis of Chloromethyl (1*R*,2*S*,5*R*)-(−)-Menthyl Ether [MenOC_1_Cl] (**2**) and Chloromethyl 1-Decyl Ether [C_10_OC_1_Cl] (**6**); Quaternisation Agents

Chloromethyl (1*R*,2*S*,5*R*)-(−)-menthyl ether and chloromethyl 1-decyl ether were synthesized according to the previously published method.^40^ HCl in the gaseous state was passed through a suspension of paraformaldehyde and the corresponding alcohol, (1*R*,2*S*,5*R*)-(−)-menthol (**1**) or 1-decanol (**5**), dissolved in toluene in an ice bath in a three-necked round-bottomed flask equipped with a thermometer and two glass tubes (gas inlet and excess gas outlet tubes). When the paraformaldehyde suspension had disappeared, the reaction was complete and two phases were visible: organic phase and water. The phases were separated and the organic phase was dried overnight with sodium sulfate. Drying agent was filtered and toluene was evaporated. The obtained chloromethyl ethers, serving as quaternising agents in subsequent steps, were purified by vacuum distillation to obtain the final pure liquids with a yield of not less than 92%. The purification *via* vacuum distillation had to be repeated before the Menschutkin reaction.

##### 2.2.1.1 Chloromethyl (1R,2S,5R)-(−)-Menthyl Ether [MenOC_1_Cl] (**2**)

93.0% yield, 0.341 mol, 69.85 g (boiling point = 82–87 °C; pressure 1–3 mbar).

##### 2.2.1.2 Chloromethyl 1-Decyl Ether [C_10_OC_1_Cl] (**6**):

92.0%, 0.312 mol, 64.51 g (boiling point = 112–117 °C; pressure ∼ 15 mbar).

#### 2.2.2 Synthesis of 1-[(1*R*,2*S*,5*R*)-(−)-Menthoxymethyl]-3-methylimidazolium [Men-Im-C_1_]­[Cl] (**3**) and 1-Decyloxymethyl-3-methylimidazolium [C_10_-Im-C_1_]­[Cl] (**7**) Chlorides *via* Menschutkin Quaternisation; Precursors of FILs

Quaternary imidazolium chlorides containing (1*R*,2*S*,5*R*)-(−)-menthol and 1-decanol moieties were synthesized under anhydrous conditions. Both reactions were carried out using 30 mL of dry *n*-hexane and freshly distilled 1-methylimidazole (0.125 mol) in a three-necked round-bottom flask equipped with a reflux condenser, mechanical stirrer and thermometer. In separate experiments, freshly distilled chloromethyl (1*R*,2*S*,5*R*)-(−)-menthyl ether (**2**) (27.38 g, 0.133 mol) and chloromethyl 1-decyl ether (**6**) (27.50 g, 0.133 mol) were independently added dropwise to vigorously stirred solutions of 1-methylimidazole. Precipitation of each chloride (**3** and **7**) occurred immediately upon addition of the corresponding quaternary agent (**2** or **6**), but stirring was continued for a further 2–3 h at room temperature to ensure completion. The resulting imidazolium chlorides [Men-Im-C_1_]­[Cl] (**3**) and [C_10_-Im-C_1_]­[Cl] (**7**) were isolated, washed with slightly warm dry *n*-hexane (3 × 30 mL) and dried overnight at 30 °C under reduced pressure (0.3 mbar) to give crude white solids (**3** and **7**). The yields of these crude quaternary imidazolium chlorides were above 98%. The products were further purified by crystallization from ethyl acetate/acetone or ethyl acetate/acetone/ethanol systems for [Men-Im-C_1_]­[Cl] and *n*-hexane/acetone or *n*-hexane/ethyl acetate systems for [C_10_-Im-C_1_]­[Cl] to give analytically pure products.

##### 2.2.2.1 1-[(1*R*,2*S*,5*R*)-(−)-Menthoxymethyl]-3-methylimidazolium Chloride [Men-Im-C_1_]­[Cl] (**3**)

Crude yield 99.5%; total yield (after purification and drying): 99.2%, 0.124 mol, 35.57 g.

##### 2.2.2.2 1-Decyloxymethyl-3-methylimidazolium Chloride [C_10_-Im-C_1_]­[Cl] (**7**)

Crude yield: 98.7%; total yield (after purification and drying): 98.1%, 0.123 mol, 35.42 g.

The synthesized quaternary imidazolium chlorides (**3** and **7**) were characterized by nuclear magnetic resonance (^1^H NMR and ^13^C NMR), with results comparable to those previously reported in the literature,^40,41^ and reported in the Supporting Information. Elemental analyzes were also performed on both chlorides (**3** and **7**) and the results are reported in the Supporting Information.

#### 2.2.3 Synthesis of 1-[(1*R*,2*S*,5*R*)-(−)-Menthoxymethyl]-3-methylimidazolium Sulfonates (**4a**, **4b**, **4e**), 1-[(1*R*,2*S*,5*R*)-(−)-Menthoxymethyl]-3-methylimidazolium Sulfonylimides (**4c** and **4d**) [Men-Im-C_1_]­[X] and 1-Decyloxymethyl-3-methylimidazolium Sulfonates (**8a** and **8b**) [C_10_-Im-C_1_]­[X]—Metathesis Process; Production of FILs

Several organic salts containing fluorinated anions in their structures were selected for the metathesis reactions. These included three sulfonate salts: potassium 1,1,2,2-tetrafluoroethanesulfonate (KTFES), potassium perfluorobutanesulfonate (KPFBS) and sodium trifluoromethanesulfonate (NaOTF). In addition, two sulfonylimide salts were used: lithium bis­(trifluoromethylsulfonyl)­imide (LiTFSI) and lithium bis­(pentafluoroethylsulfonyl)­imide (LiPFSI). The metathesis reactions were carried out in either aqueous or methanolic solutions, chosen on the basis of the water solubility of the respective salts.

Each of the above salts (0.053 mol) was dissolved either in 30 mL of distilled water (for water-soluble salts: LiTFSI and LiPFSI) or in 30 mL of methanol (for water-insoluble salts: KPFBS, KTFES and NaOTF). These homogeneous aqueous or methanolic solutions were then added to an aqueous or methanolic solution of 1-[(1*R*,2*S*,5*R*)-(−)-menthoxymethyl]-3-methylimidazolium chloride (**3**) (0.002 mol·mL^–1^) or 1-decyloxymethyl-3-methylimidazolium chloride (**7**) (0.002 mol·mL^–1^). In each case, the reaction was almost instantaneous and was monitored using ascending thin layer chromatography on silica gel G (Merck 1.05570.0001) with UV light visualization. The metathesis reaction mixture was stirred at room temperature for approximately 1 day to ensure completion. Aqueous reaction mixtures used for the preparation of [Men-Im-C_1_]­[TFSI] (**4c**) and [Men-Im-C_1_]­[PFSI] (**4d**) resulted in heterogeneous solutions. In these cases each product was washed three times with distilled water and the phases were separated. For the methanolic mixtures used to obtain all the remaining salts [Men-Im-C_1_]­[TFES] (**4a**), [C_10_-Im-C_1_]­[TFES] (**8a**), [Men-Im-C_1_]­[PFBS] (**4b**), [C_10_-Im-C_1_]­[PFBS] (**8b**) and [Men-Im-C_1_]­[OTF]) (**4e**), the crude products were obtained after evaporation of the alcoholic solvent. Subsequent purification steps were applied to all salts obtained (**4a**–**4e**, **8a**–**8b**). Each imidazolium salt was dissolved in acetone and stirred for 30 min to precipitate any residual halide salts (KCl, LiCl or NaCl), which were then removed by filtration through a 0.2 μm filter. The filtrate was placed in a freezer at approximately −5 °C overnight to allow any residual halide salts to precipitate. If halide salts precipitated, the mixture was filtered again, and the filtrate was evaporated. This process was repeated until no further inorganic salt precipitate was observed. The absence of chloride ions was additionally confirmed using AgNO_3_ to ensure that no AgCl precipitate was formed. The crude salts were repeatedly dissolved in solvents of varying polarity, namely chloroform and acetonitrile, in order to obtain high purity of imidazolium salts: [Men-Im-C_1_]­[TFES] (**4a**), [C_10_-Im-C_1_]­[TFES] (**8a**), [Men-Im-C_1_]­[PFBS] (**4b**), [C_10_-Im-C_1_]­[PFBS] (**8b**), [Men-Im-C_1_]­[TFSI] (**4c**), [Men-Im-C_1_]­[PFSI] (**4d**), and [Men-Im-C_1_]­[OTF] (**4e**).

All the resulting compounds (**4a**–**4e**, **8a**–**8b**) in which the anions are as follows: 1,1,2,2-tetrafluoroethanesulfonate (two salts **4a** and **8a**), perfluorobutanesulfonate (two salts **4b** and **8b**), bis­(trifluoromethylsulfonyl)­imide (one salt **4c**), bis­(pentafluoroethylsulfonyl)­imide (one salt **4d**) and trifluoromethanesulfonate (one salt **4e**) anions, were dried under vacuum (5 mbar) for several days at temperatures ranging from 45 to 55 °C using a cold trap and an inert gas to obtain pure products. The salts obtained in the form of solids were crystallized using a water/ethanol system for [Men-Im-C_1_]­[TFES] (**4a**) and [Men-Im-C_1_]­[PFBS] (**4b**) and *n*-hexane/ethyl acetate for [Men-Im-C_1_]­[OTF] (**4e**), giving high-purity salts. Total yields for all salts obtained, including purification (e.g., washing with suitable solvents, drying or crystallization for solids) ranged from 97.9% for the [C_10_-Im-C_1_]­[TFES] salt (**8a**) to 99.1% for the [Men-Im-C_1_]­[TFES] (**4a**) and [Men-Im-C_1_]­[TFSI] (**4c**) FILs.

### 2.3 General Method for the Soil Phytotoxicity Test

Studies to determine the phytotoxicity of FILs were carried out as a pot experiment under controlled conditions in the vegetation hall of the Department of Biochemistry, Biotechnology and Ecotoxicology of Jan Długosz University in Częstochowa, based on the OECD/OCDE 208/2006 guideline.^42^ A dicotyledonous plant—common radish (*R. sativus* L. subvar. *radicula* Pers.) of the Mila cultivar, produced by PlantiCo Zielonki Sp. z o.o. company, was used in the experiment. Twenty identical seeds were sown in each pot. Each determination was performed at least 3 times. The pots were filled with reference soil and the soil was mechanically mixed with the tested FIL. The treatment concentration of FIL was set at 0, 1, 10, 50, 100, 400, 700, and 1000 mg·kg^–1^ soil DW. The soil used in the experiment was clayey sand with an organic carbon content of 0.9% and a pH (KCl) of 6.0. Throughout the study, constant soil moisture was maintained at the level required for the plants (70% field water capacity), a constant temperature of 20 ± 2 °C and constant light of 170 μmol·m^–2^·s^–1^ in a 16 h—day and 8 h—night system. Fourteen days after sowing the seeds in the soil, the length of the aboveground parts of the plants and their roots were measured and material was collected for further testing.

The FW yield of the plants and the stunting of their roots and aboveground parts were determined as indicators of FIL toxicity. The length of aboveground parts and roots was measured as described by Wang et al.^43^ The results obtained at this stage of the study were expressed as % inhibition of FW yield, aboveground part length and root length compared with the control, and the effective EC_50_ concentration was calculated and estimated by nonlinear regression analysis using GraphPad Prism software (GraphPad Software, Inc., La Jolla, CA, USA). DW content of the plants was determined using the drying-to-constant-weight method, according to the method described by Kowalska,^44^ by drying approximately 1 g of fresh plant material at 105 °C until a constant weight was achieved.

In addition, the contents of assimilation pigments, malondialdehyde (MDA) and H_2_O_2_ were determined in the fresh plant material, as well as the activity of the antioxidant enzymes superoxide dismutase (SOD), catalase (CAT) and peroxidase (POD). Samples treated with high concentrations of FILs were not included in some of the analyzes because the inhibition of growth of the aboveground parts of the radish plants did not allow sufficient plant material to be obtained for the study.

#### 2.3.1 Photosynthetic Pigments

The content of assimilation pigments (chlorophyll *a*, chlorophyll *b* and carotenoids) was determined by spectroscopy according to the method described by Oren et al.^45^ For this purpose, 200 mg of fresh leaf tissue was homogenized with an 80% acetone solution cooled to 4 °C and then centrifuged. The resulting supernatant was made up to a volume of 25 mL and the absorbance was measured at 470 nm, 647 nm and 664 nm. The content of assimilatory pigments was calculated and expressed as mg·g^–1^ plant DW.

#### 2.3.2 MDA and Hydrogen Peroxide (H_2_O_2_)

For the determination of MDA and H_2_O_2_, 0.5 g of fresh leaf mass was homogenized with a chilled (4 °C) 0.1% trichloroacetic acid solution, followed by centrifugation. The H_2_O_2_ content was determined by spectrophotometric measurement of a reaction mixture containing supernatant, phosphate buffer at pH of 7.0 and potassium iodide according to the method described by Singh et al.^46^ An extinction coefficient of 155 nm^–1^·cm^–1^ was used to calculate the H_2_O_2_ content, and the result was expressed in μmol·g^–1^ plant FW. The MDA content was determined by spectrophotometric measurement of a reaction mixture containing supernatant, phosphate buffer pH of 7.0 and thiobarbituric acid according to the method described by Hodges et al.^47^ The MDA content was calculated using an extinction coefficient of 155 nm^–1^·cm^–1^ and expressed as μmol·g^–1^ plant FW.

#### 2.3.3 Antioxidant Enzymes

To determine the activity of antioxidant enzymes in common radish leaves, 0.5 g of fresh leaf mass was homogenized with the addition of a chilled (4 °C) extraction mixture (phosphate buffer (pH = 7.4), 1 mM EDTA solution and 0.1% polyvinylpyrrolidone (PVP) solution), followed by centrifugation. The supernatant obtained was used to determine the activity of the enzymes tested and the protein content.

The total protein content was determined by Bradford method using Coomasine Blue pigment.^48^ Data on the protein content of plant leaves are necessary for the conversion of antioxidant enzyme activity. Superoxide dismutase (SOD) activity was determined spectrophotometrically by measuring the rate of reduction of nitrotetrazolium blue (NBT), according to the method described by Giannopolitis and Ries.^49^ SOD activity was expressed in units of activity—U·mg^–1^ protein. U—one unit of SOD activity, corresponds to the amount of enzyme that causes a 50% inhibition of the NBT reduction reaction rate. Catalase (CAT) activity was determined by the decomposition of H_2_O_2_ by the enzyme over 15 min. The remaining H_2_O_2_ in the mixture was titrated with 0.01 N KMnO_4_. Catalase activity was expressed as *U*—mg^–1^ protein·min^–1^.^50^ POD activity was determined using a spectrophotometric method by measuring the rate of oxidation of guaiacol in the presence of H_2_O_2_ at 470 nm for 1 min. POD activity was expressed as *U*—mg^–1^ protein·min^–1^.^51^

#### 2.3.4 Statistical Analysis

All experimental results obtained were statistically analyzed using STATISTICA 13.3. Data from three measurements (*n* = 3) were analyzed using one-way ANOVA followed by Tukey’s posthoc test. Significant differences were considered at the *p* < 0.05 level and are shown in the tables for each assay. Results are presented as the arithmetic mean of the individual measurements ± standard deviation (SD).

## 3 Results and Discussion

### 3.1 Structural Rationale and Selection of Menthol-Based Functionalized Ionic Liquids for Phytotoxicity Assessment

#### 3.1.1 Motivation and Scope of Structure–Effect Analysis

In the present study, the selection of compounds for phytotoxicity testing and structure–effect analysis was driven by the need to address the limited understanding of whether covalently bound functional elements influence phytotoxic responses in FILs with broad application potential.

Under environmental conditions, including soil and aqueous systems, ILs—including FILs—are present predominantly as solvated ions rather than as persistent cation–anion pairs.^3^ Their interactions with biological systems are therefore governed by ion-specific processes, with toxicity dominated by baseline, membrane-associated mechanisms that are well-known from surfactant chemistry. This interpretation is fully consistent with current ionic-liquid toxicology literature, which identifies membrane partitioning and nonspecific interactions with lipid bilayers as the dominant mode of action for ILs in aqueous environments.^3,4,52^ Importantly, while the biological mode of action of such systems is surfactant-like, extensive literature demonstrates that the resulting environmental and phytotoxic behavior is not generic. Instead, it is strongly modulated by molecular features intentionally introduced at the ionic-liquid design stage, including cation geometry, structural isomerism, covalent functional substituents and anion identity.^3,4,21^ In particular, studies by Beil et al.^3^ emphasized that spatial isomerism and molecular geometry significantly affect toxicity profiles of ILs, while Pawłowska and co-workers^21^ have demonstrated that phytotoxic responses depend sensitively on cation architecture even for compounds of comparable lipophilicity. Accordingly, the present study does not seek to distinguish ILs from surfactants solely on the basis of environmental mode of action. Instead, it applies the ionic-liquid design paradigm to investigate how covalent functionalization of the cation and anion identity modulate surfactant-like phytotoxic responses within a controlled and structurally well-defined chemical framework.

FILs are increasingly considered for diverse uses, which necessitates defining their environmental hazard profiles, including phytotoxicity, as an integral part of their evaluation. Comparing a structurally complex, functionally relevant substituent with a simpler structural analogue of the same carbon number provides an appropriate framework for assessing the specific contribution of functionalization to phytotoxic behavior, while maintaining a clear reference to more fundamental IL structures. To the best of our knowledge, direct phytotoxicity comparisons between functionally simple linear alkyl substituents and structurally complex cyclic functional components of identical carbon number remain largely unexplored. Such an approach enables the role of functional elements to be analyzed in a broader and more general context, supporting hazard identification for FILs intended for use across multiple application domains.

#### 3.1.2 Menthol as a Model Functional Component in FILs

Within this framework, we focused on a group of FILs containing the (1*R*,2*S*,5*R*)-(−)-menthol moiety as a model functional substituent, selected due to its well-documented multifunctional character.^53^ (−)-Menthol is a monoterpene alcohol characterized by three stereogenic centers and a rigid cyclohexane framework. It is readily available, cost-effective, and derived from renewable natural sources, being a major constituent of essential oils obtained from several *Mentha* species,^54^ and therefore represents a structurally and practically attractive compound consistent with the principles of sustainable and biobased chemical design. (−)-Menthol is an abundant and economically accessible compound (≈60 USD kg^–1^), widely used as a flavouring and cooling agent, and its physicochemical and biological properties have been extensively characterized in the literature,^55^ providing a reliable and well-established reference for the development of compounds with targeted functions, including menthol-based FILs.^56–58^

#### 3.1.3 Selection of Fluorinated Anions for the Studied FILs

The integration of anions containing one or more fluorine atoms into IL structures has become increasingly common, reflecting their numerous technologically relevant applications across diverse fields.^59–63^ Fluorinated anions are frequently employed to modulate key physicochemical properties of ILs, including hydrophobicity, thermal stability, and ionic character. At the same time, numerous studies have clearly demonstrated that fluorine-containing ILs are often associated with elevated toxicity and environmental persistence, raising concerns regarding their environmental compatibility and safety.^3,4^

Despite these concerns, fluorinated anions continue to be widely explored due to their functional advantages. One notable example is their application in metal extraction processes. Zhou et al.^64^ reported the use of 1-hydroxyethyl-3-methylimidazolium bis­(trifluoromethylsulfonyl)­imide [HOC_2_–Im-C_1_]­[TFSI] as a coextraction agent for lithium recovery from brines. Subsequent studies,^65,66^ demonstrated that hydroxyl functionalization within the imidazolium core significantly enhances extraction efficiency compared to nonfunctionalized alkyl imidazolium ILs. Fluorinated anions have also been shown to strongly influence biological activity. In our previous work,^67^ FILs bearing alkoxymethyl substituents combined with bis­(trifluoromethylsulfonyl)­imide anions exhibited pronounced and selective antibacterial activity, particularly against *Staphylococcus* spp., with performance comparable to or exceeding that of benzalkonium chloride. Importantly, the most active compound in the homologous series, 1-dodecyloxymethyl-3-ethylimidazolium bis­(trifluoromethylsulfonyl)­imide, did not induce hemolysis and did not disrupt artificial Gram-positive bacterial membranes, highlighting a complex relationship between functionalization, anion identity, and biological effects. Beyond antimicrobial systems, fluorine-containing ILs and FILs have been applied as solvents for metal oxides,^68,69^ sorbents,^70,71^ additives in perovskite devices,^72^ sensing materials,^73^ and components in photopolymerization processes.^74^ The coexistence of broad functional utility and documented toxicity underscores the necessity of detailed environmental hazard assessment for fluorinated FILs. In this context, investigating whether the incorporation of a bulky, naturally derived functional component can modulate phytotoxic responses represents a relevant and timely research direction.

#### 3.1.4 Functional Relevance of Menthol-Based FILs Bearing Fluorinated Anions

Optically active monoterpene (−)-menthol has been shown in our previous studies to serve as a key structural scaffold of FILs containing fluorinated anions, exerting strong, structure-dominant effects across diverse functional contexts, including antibacterial,^75^ antifungal,^40^ catalytic,^76^ and antielectrostatic applications,^77,78^ as well as active materials for electrically driven broadband mirrors.^58^ Those menthol-based FILs have also been employed in enzymatic processes as activators and stabilizers.^79^ The broad spectrum of demonstrated functionalities associated with menthol-based FILs highlights the need for a comprehensive evaluation of their environmental hazard profile, including phytotoxicity, as an integral aspect of their assessment. Consequently, menthol-derived FILs bearing fluorinated anions were selected in the present study as a representative and relevant model system for investigating whether covalently bound functional components influence phytotoxic responses in FILs.

#### 3.1.5 Comparative Framework for Phytotoxicity Analysis

Although the observed phytotoxic effects are consistent with membrane-associated, surfactant-like mechanisms, the comparative framework applied in this study is designed to test whether anion identity and cation architecture introduce systematic modulation of toxicity that cannot be explained solely by classical surfactant descriptors such as alkyl chain length or surface activity.^52^ This approach follows concepts established in studies on IL-based surfactants, where molecular design parameters derived from IL chemistry are explicitly linked to aggregation behavior and biological responses in aqueous systems.^52,80–82^

In environmental and biological research, ILs are inherently studied under aqueous conditions, where dissociation and solvation of ions are expected. Consequently, interactions with organisms are commonly interpreted in terms of amphiphilicity, aggregation behavior and structure–activity relationships characteristic of surfactant-like systems.^52,82,83^ Importantly, such systems are still analyzed within the broader framework of IL chemistry, in which molecular features intentionally introduced at the design stage—such as cation architecture, covalent functional substituents and anion identity—are treated as deliberate variables governing aggregation phenomena and biological responses in water, rather than as incidental properties of classical surfactants.^82,84,85^

Building on the functional relevance and environmental considerations associated with (−)-menthol-based FILs bearing fluorinated anions, a comparative framework was established to evaluate how anion identity and functional substituent architecture influence phytotoxic responses. Here we focus on five (1*R*,2*S*,5*R*)-(−)-menthol-based FILs, which differ exclusively in the type of anion, while all anions contain fluorine atoms.

This design leads to our first research question (Q1) for FILs with the same cation containing the naturally derived (1*R*,2*S*,5*R*)-(−)-menthol component (Scheme 2, Q1): How does phytotoxicity change with the type of fluorinated anion in FILs containing the natural (1*R*,2*S*,5*R*)-(−)-menthol component?

**Scheme 2:**
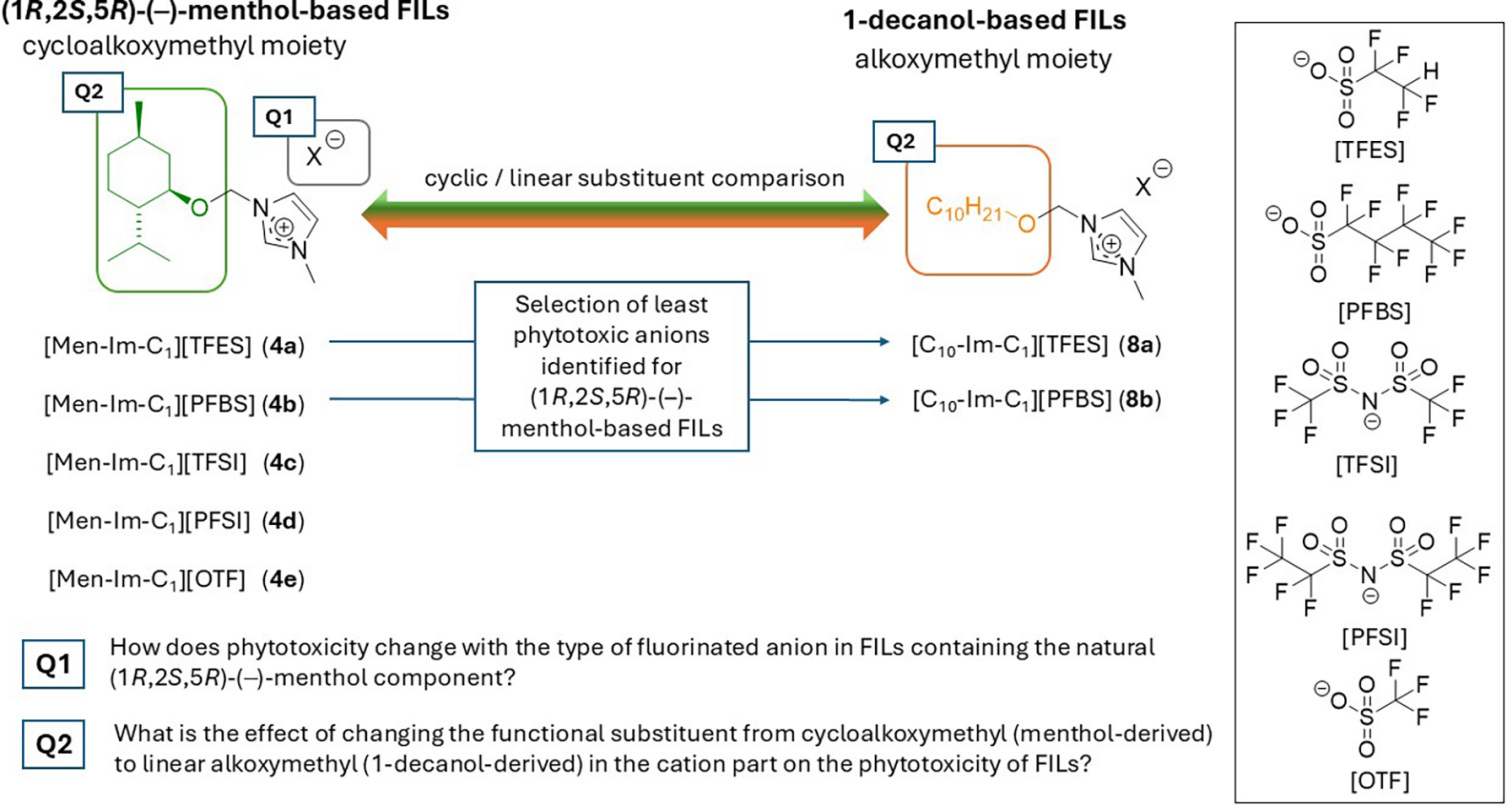
Structural Design of FILs for Phytotoxicity Comparison: (1*R*,2*S*,5*R*)-(−)-Menthol- and 1-Decanol-Derived Cations Combined with Fluorinated Anions^a^

a Selection of compounds enables the investigation of Q1 and Q2 research questions.

To address the second question regarding the role of the functionalized cation, a series of structural analogues were synthesized using 1-decanol in place of (1*R*,2*S*,5*R*)-(−)-menthol. These FILs incorporate linear alkoxymethyl substituents, in contrast to the cycloalkoxymethyl group of the menthol-based FILs; this enabled a direct comparison between cyclic and linear functionalization within the cationic framework. Accordingly, we formulated Q2: What is the effect of changing the functional substituent from cycloalkoxymethyl (menthol-derived) to linear alkoxymethyl (1-decanol-derived) in the cation part on the phytotoxicity of FILs?

The comparison between these two types of cationic substituents was conducted using the two least phytotoxic anions identified during the evaluation of Q1. 1-Decanol is a linear fatty alcohol, whereas (1*R*,2*S*,5*R*)-(−)-menthol is a cyclic monoterpene alcohol of natural origin. Both substituents contain ten carbon atoms, enabling a focused assessment of the impact of functional substituent geometry (linear *vs* cyclic) and overall molecular structures on phytotoxic behavior. Importantly, the comparative framework introduced here does not aim to exhaustively resolve cation- and anion-dependent effects, but rather demonstrates the necessity of continued, systematic structure-resolved investigations of both ionic components, as their individual and combined contributions to phytotoxicity cannot be generalized from single descriptors or limited data sets.

This comparative framework enables phytotoxicity trends to be interpreted in relation to clearly defined molecular features, contributing to hazard-oriented evaluation of the environmental profiles of FILs with broad application potential.

### 3.2 Synthesis of FILs Bearing (1*R*,2*S*,5*R*)-(−)-Menthol (**4a–4e**) and 1-Decanol (**8a–8b**)

Imidazolium-based FILs bearing either the (1*R*,2*S*,5*R*)-(−)-menthol or 1-decanol moiety were synthesized *via* a three-step process, as illustrated in Scheme 3. The first step involved the chloromethylation of (1*R*,2*S*,5*R*)-(−)-menthol (**1**) or 1-decanol (**5**) to produce the corresponding ether-type quaternising agents [MenOC_1_Cl] (**2**) and [C_10_OC_1_Cl] (**6**), following previously established procedures.^40^ In particular, this reaction must be carried out under strictly anhydrous conditions to prevent hydrolysis, which would otherwise regenerate the starting chemicals—hydrochloride, paraformaldehyde and the corresponding alcohol.

**Scheme 3:**
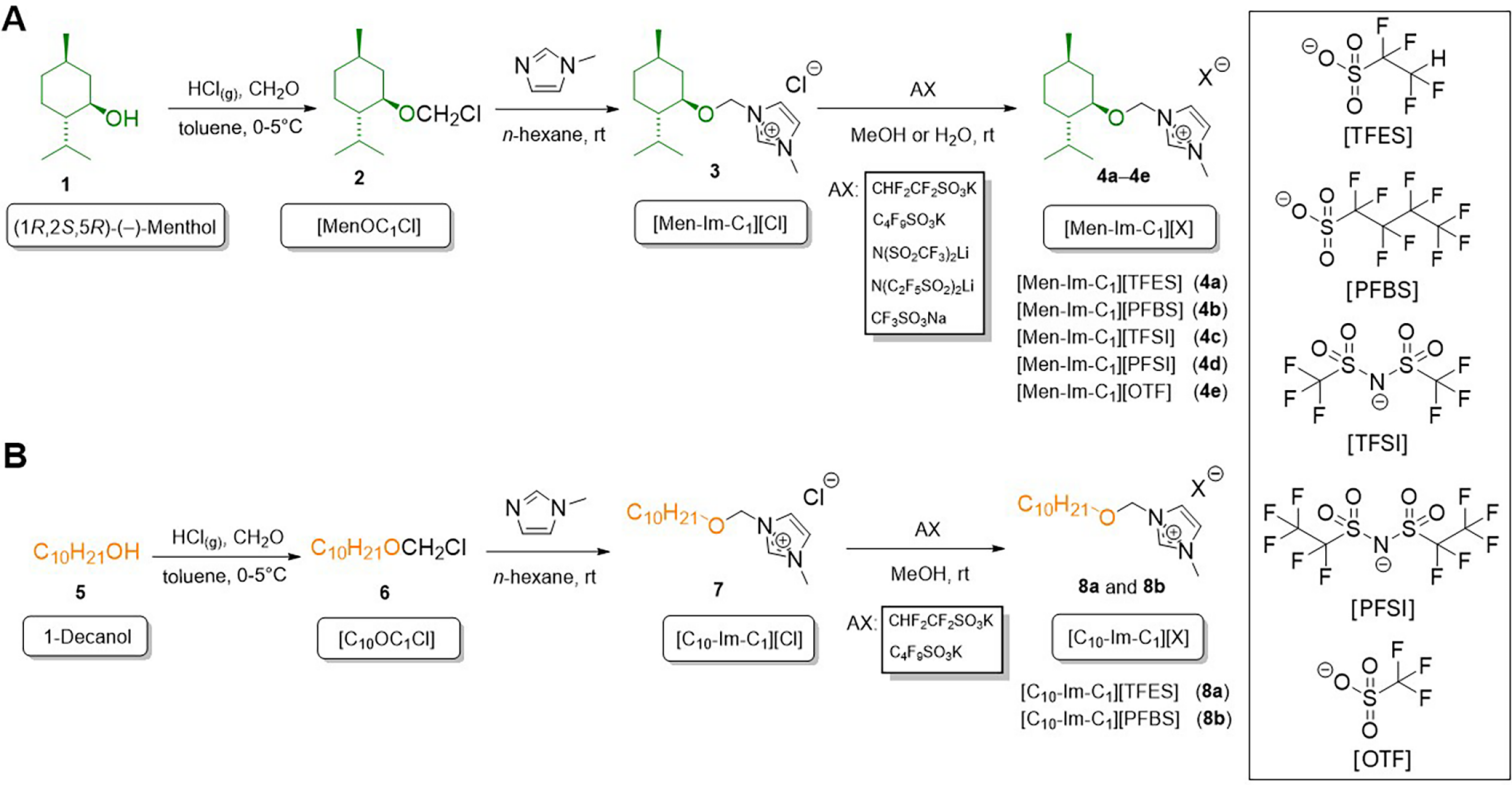
Synthesis Pathway of FILs: (A) FILs Bearing a Cycloalkoxymethyl Substituent ((1*R*,2*S*,5*R*)-(−)-Menthol-Derived); (B) FILs Bearing a Linear Alkoxymethyl Substituent (1-Decanol-derived)

In the second step, quaternisation was achieved *via* a Menschutkin reaction between 1-methylimidazole and the chloromethyl ether intermediates: chloromethyl (1*R*,2*S*,5*R*)-(−)-menthyl ether (**2**) or chloromethyl 1-decyl ether (**6**). To ensure high product purity and reaction efficiency in the synthesis of 1-[(1*R*,2*S*,5*R*)-(−)-menthoxymethyl]-3-methylimidazolium chloride [Men-Im-C_1_]­[Cl] (**3**) and 1-decyloxymethyl-3-methylimidazolium chloride [C_10_-Im-C_1_]­[Cl] (**7**), all reagents were distilled under reduced pressure prior to use. The quaternisation processes were performed in anhydrous *n*-hexane. In this nonpolar solvent, [Men-Im-C_1_]­[Cl] (**3**) and [C_10_-Im-C_1_]­[Cl] (**7**) precipitated directly from the reaction mixture. This process proceeded rapidly, affording the crude products of the quaternary imidazolium chlorides (**3** and **7**) in high yields, ranging from 92% (**7**) to 99% (**3**), in agreement with previous reports.^40,86^ The crude products were further purified by crystallization: [Men-Im-C_1_]­[Cl] (**3**) from an ethyl acetate/acetone/ethanol system, while [C_10_-Im-C_1_]­[Cl] (**7**) from an *n*-hexane/ethyl acetate system, yielding analytically pure quaternary imidazolium chlorides.

Although ether-type linkages were selected in this work for their chemical stability, mild reaction conditions, and high overall yields, other synthetic routes to quaternary imidazolium salts—most commonly involving ester-type linkages—are also known. These alternative methods often require elevated temperatures, prolonged reaction times, and more complex workup procedures, and in some cases deliver lower overall process efficiency or product yields. Such factors not only increase energy consumption but may also limit scalability and sustainability. Among the alternative methods, Berton et al.^87^ synthesized a quaternary imidazolium chloride by reacting a chloroester-based geraniol derivative ((*E*)-3,7-dimethylocta-2,6-dienyl 2-chloroacetate) with 1-methylimidazol, achieving a yield of over 97% for (*E*)-1-(2-(3,7-dimethylocta-2,6-dienyloxy)-2-oxoethyl)-3-methylimidazolium chloride. In this system, the ester bond served as a covalent yet easily cleavable linkage suitable for designing ILs intended for fragrance delivery media. Similarly, Akopyan et al.^88^ reported the synthesis of an ester-linked imidazolium chloride derived from (1*R*,2*S*,5*R*)-(−)-menthol, affording 1-[(1*R*,2*S*,5*R*)-(−)-menthoxycarbonylmethyl]-3-methylimidazolium chloride as a dark yellow solid in 82% yield. Although quaternary salts obtained using ester-based quaternisation agents can deliver high yields, the quaternisation step in both of these examples required more demanding reaction conditions compared to our ether-based approach.

In the final step, ion exchange process was carried out between the synthesized quaternary imidazolium chloride, namely 1-[(1*R*,2*S*,5*R*)-(−)-menthoxymethyl]-3-methylimidazolium chloride (**3**) or 1-decyloxymethyl-3-methylimidazolium chloride (**7**), and selected organic salts containing fluorinated atoms: KTFES, KPFBS, LiTFSI, LiPFSI and NaOTF. The metathesis reactions afforded seven salts that can be classified as ILs based on their physicochemical properties.^89^ Moreover, these compounds also meet the criteria for classification as FILs, due to the presence of covalently attached functionalized alkoxyl substituents. Notably, six of these ionic compounds are novel and have not been previously described in the literature. The exception is 1-[(1*R*,2*S*,5*R*)-(−)-menthoxymethyl]-3-methylimidazolium bis­(trifluoromethylsulfonyl)­imide (**4c**), which was previously reported by our research group.^40,90^ Among the synthesized salts, four—[Men-Im-C_1_]­[TFSI] (**4c**), [Men-Im-C_1_]­[PFSI] (**4d**), [C_10_-Im-C_1_]­[TFES] (**8a**) and [C_10_-Im-C_1_]­[PFBS] (**8b**)—exhibited a liquid physical state at room temperature, classifying them as room temperature ionic liquids (RTILs). The remaining salts—[Men-Im-C_1_]­[TFES] (**4a**), [Men-Im-C_1_]­[PFBS] (**4b**) and [Men-Im-C_1_]­[OTF] (**4e**)—were obtained as solids with melting points below 100 °C. These solid salts (**4a**, **4b** and **4e**) were further purified by crystallization from the following solvent systems: *n*-hexane/acetone, *n*-hexane/ethyl acetate, and water/ethanol, respectively, yielding analytically pure salts. Notably, [Men-Im-C_1_]­[TFES] (**4a**) was initially obtained as a colorless liquid, but solidified upon standing, suggesting partial supercooling or kinetic stabilization of the liquid phase. The synthesized FILs (**4a**–**4e**, **8a**–**8b**) were characterized by nuclear magnetic resonance (^1^H NMR, ^13^C NMR and ^19^F NMR) and Fourier transform infrared spectroscopy (FTIR). The results are reported in the Supporting Information (Figures S12–S45). The influence of anion variation on NMR chemical shifts is presented in Tables S2 and S3 as well as in Figures S46 and S47.

### 3.3 Physicochemical Properties of FILs Bearing (1*R*,2*S*,5*R*)-(−)-Menthol (**4a–4e**) and 1-Decanol (**8a–8b**)

Understanding the physicochemical characteristics of FILs is crucial for predicting their environmental fate, biological activity, and potential applications.^91–93^ Even subtle modifications to the cationic or anionic moieties can significantly affect parameters such as thermal stability, hydrophobicity, viscosity, water solubility, and surface activity in the context of structure–property relationships.^57,59,94^ These parameters collectively govern the bioavailability of FILs and their interactions with biological membranes, influencing critical end points such as biocompatibility, cytotoxicity and bioaccumulation potential. They also serve as a basis for targeted structural optimization to minimize environmental persistence and unintended toxicity, while maintaining performance in processing and formulation systems, and ensuring favorable environmental and toxicological profiles.

In this study, we focus on FILs that contain quaternary imidazolium cations functionalized with either (1*R*,2*S*,5*R*)-(−)-menthol or 1-decanol moieties. Due to their hydrophobic and partially rigid characteristics, these groups can markedly influence the polarity and amphiphilic properties of the resulting compounds. Furthermore, the nature and spatial arrangement of these substituents strongly affect molecular packing, aggregation tendencies and surface behavior—all important factors in how they behave within environmental matrices and agricultural soils. This section summarizes the key physicochemical characteristics of the synthesized FILs bearing either (1*R*,2*S*,5*R*)-(−)-menthol (**4a**–**4e**) or 1-decanol (**8a**–**8b**) as substituents.

Solubility data in various solvents are presented in Table 1. These results provide a starting point for understanding the solvation properties of the investigated salts, which determine their interactions in biological or environmental systems and inform predictions of their behavior under diverse conditions that are relevant for industrial and agrochemical applications. Table 2 compiles a range of experimentally determined parameters, including physical state, thermal behavior (e.g., melting points and phase transitions), thermal stability, refractive index (Figure S11), and optical rotation. These descriptors are important for assessing the thermal and thermodynamic stability, amphiphilicity and potential environmental persistence of tested imidazolium-based salts. Figure 1 provides an overview of both experimental and predicted parameters relevant to the environmental behavior of the investigated FILs. Part A summarizes a selection of molecular and physicochemical properties, including calculated molecular volume (Figure S48) and asymmetry index, as well as experimentally measured water solubility and surface tension for each FIL. Part B illustrates the structural divergence between the (1*R*,2*S*,5*R*)-(−)-menthol-based and 1-decanol-based substituents. Parts C and D present the results of predictive modeling, showing the predicted lipophilicity (log *K*_OW_) and bioconcentration factor (log BCF) values for all synthesized FILs, respectively. These parameters are essential for predicting the environmental behavior of the FILs, including their mobility, bioavailability, and potential to accumulate in biota.

**Table 1:**
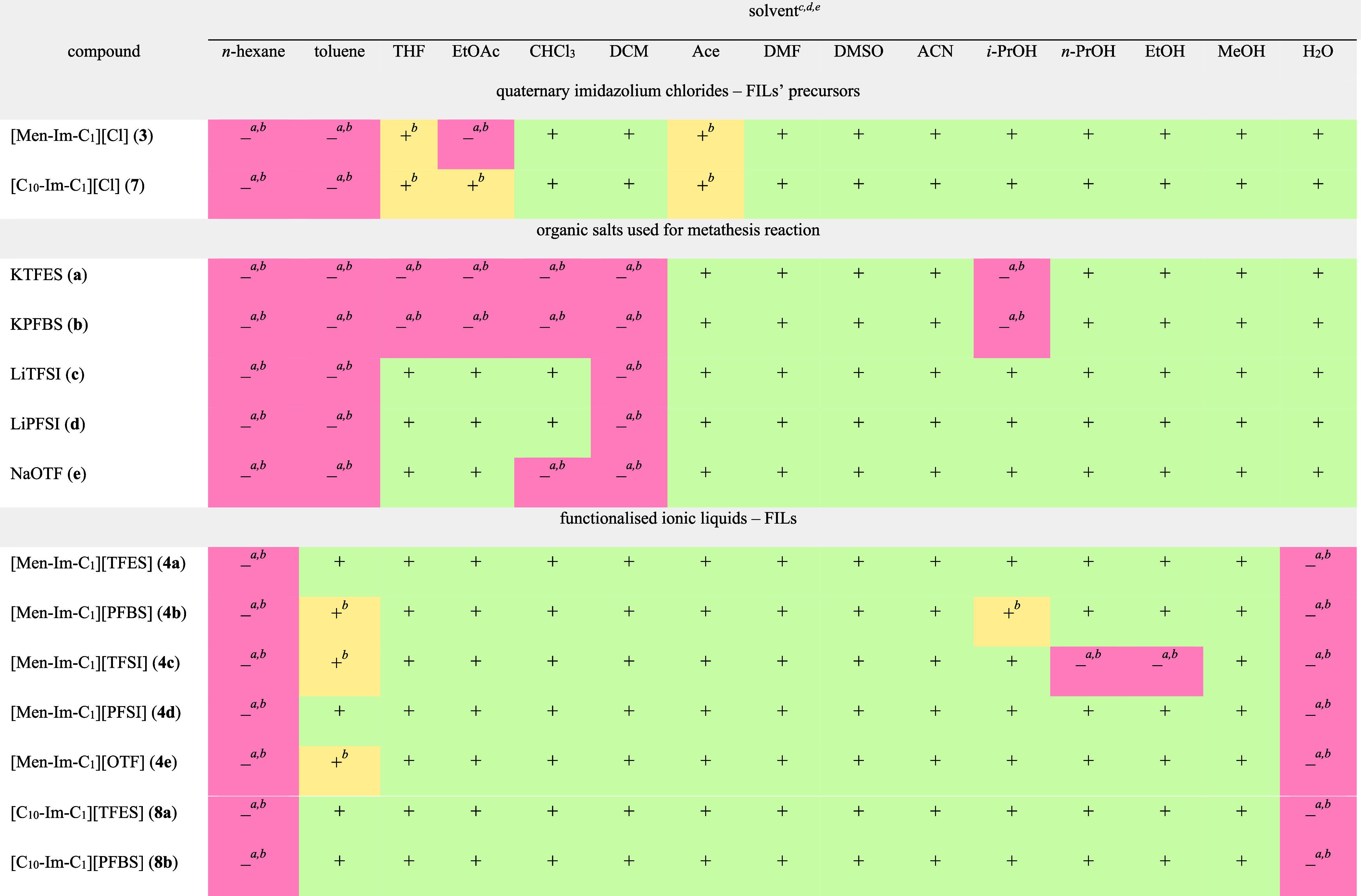
Solubility of Obtained FILs with (1*R*,2*S*,5*R*)-(−)-Menthol and 1-Decanol Group^a^ ^,^ ^b^

a Measurements performed at 25 °C.

b Measurements carried out at 50 °C.

c Solubility categories: complete soluble—green; limited solubility—yellow; insoluble—red.

d Ordered with increasing polarity.

e Solvents’ abbreviations: tetrahydrofuran (THF), ethyl acetate (EtOAc), chloroform (CHCl_3_), dichloromethane (DCM), acetone (Ace), dimethylformamide (DMF), dimethyl sulfoxide (DMSO), acetonitrile (ACN), *iso*-propanol (*i*-PrOH), *n*-propanol (*n*-PrOH), ethanol (EtOH), methanol (MeOH) and water (H_2_O).

**Table 2:** Physicochemical Properties of FILs with (1*R*,2*S*,5*R*)-(−)-Menthol (**4a–4e**) and 1-Decanol (**8a–8b**) Substituent

FIL	physical state^b^	thermal behavior^c^ ^,^ ^d^ (°C)										
		glass-transition point *T*_g_	crystallization *T*_c_	isomorphic to liquid crystal transition *T*_LC_	cold crystallization *T*_cc_	precrystallization relaxation temperature *T*_pr_	melting point *T*_m_		thermal stability^f^ *T*_d_		refractive index^g^ *n*_D_^25^	specific rotation^h^ ^,^ ^i^ ^,^ ^j^[α]_D_^25^
							digital *T*_m_ apparatus^e^	DSC	*T*_5%onset_	*T*_onset_		
**4a**	white crystal	–25.39	–	–	–	–	54.0–56.9	59.29	203.90	347.57	–	–70.435 (*c* 1.013)
**4b**	white crystal	–	34.61	–	–	–	87.2–89.1	86.17	197.25	350.16	–	–57.396 (*c* 1.026)
**4c**	liquid^a^	–39.28	–	–	–	–	–	–	207.56	343.98	1.44844	–59.601 (*c* 1.003)
		–44^k^							230^l^	250^l^	1.4483^m^	–63.3^n^(*c* 1.5)
**4d**	liquid	–36.79	–	–	–	–	–	–	223.37	374.30	1.43213	–49.684 (*c* 1.062)
**4e**	white crystal	–14.10	–	–	15.42	–	83.1–84.7	82.53	184.94	320.27	–	–76.441 (*c* 1.043)
					36.58							
**8a**	liquid	–39.93	–	–6.47	–25.98	–30.78	–	25.54	188.14	366.10	1.44602	–
				–41.62								
**8b**	liquid	–54.85	–	16.80	–34.46	–	–	25.25	198.81	373.08	1.41728	–
					–16.41							

a Compound known in the literature.^40,90^

b At 25 °C.

c Accuracy ± 0.01 °C.

d The values of *T*_m_, *T*_c_, *T*_cc_, *T*_pr_ and *T*_LC_, were taken as the peak temperature of the transition upon heating/cooling cycles, while *T*_g_ was taken as the midpoint. Experiments were carried out with a heating/cooling rate of 10 °C·min^–1^.

e Data obtained from an electrothermal digital melting point apparatus with accuracy ± 0.1 °C.

f Experiments were carried out with a heating rate of 5 °C·min^–1^. *T*_5%onset_ temperature refers to decomposition of the first 5% of the sample and *T*_onset_ decomposition of the 50% of the sample.

g Standard uncertainty of ± 0.00004 (*n*_D_^25^).

h In methylene chloride.

i Standard uncertainty for specific rotation u is *u*(α) = ± 0.5°.

j Standard uncertainty for concentration u is *u*(*c*) = ± 0.00002 g·mL^–1^.

k Phase transitions were measured by PerkinElmer differential scanning calorimeter at a rate of 20 °C·min^–1^.

l Experiment was performed using a MOM (Hungary) Derivatograph-PC.

m Experiment was performed using AR Abbe refractometer 2 (A. Krüss Optronic GmbH) with an uncertainty ± 0.0003 (*n*_*D*_^25^).

n Specific rotation was measured using a PerkinElmer 243 B polarimeter at 20 °C. Solution of **4c** was prepared in ethanol.^40^

**Figure 1:**
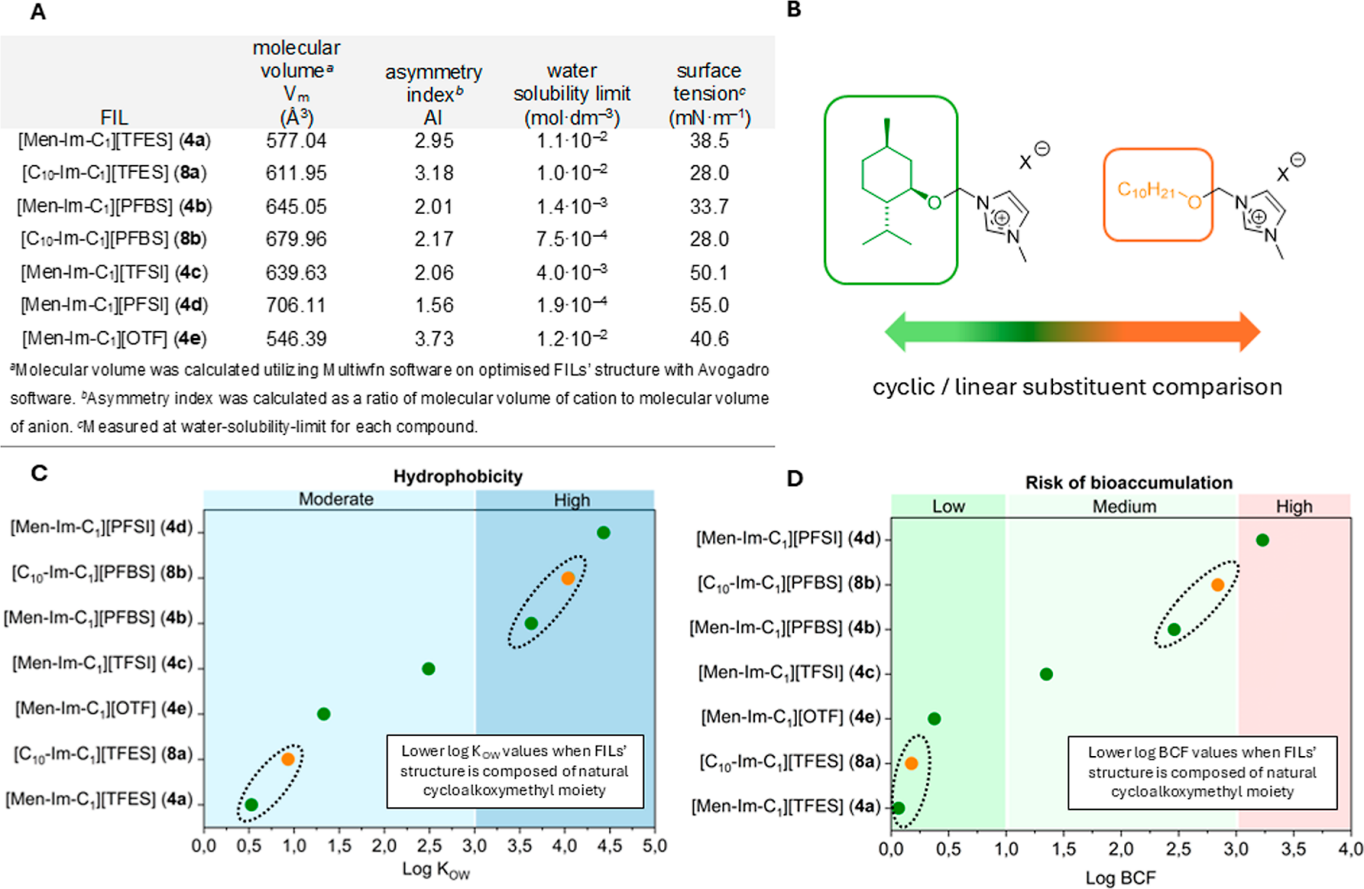
Experimental and predicted properties of functionalized ionic liquids (FILs) relevant to environmental behavior: (**A**) Overview of physicochemical properties: molecular volume and asymmetry index (calculated), water solubility limits and surface tension (measured). (**B**) Structural divergence between the (1*R*,2*S*,5*R*)-(−)-menthol-based and 1-decanol-based substituents. (**C**). Effect of cation and anion type on lipophilicity—predicted log *K*_OW_ of all obtained FILs with cycloalkoxymethyl (**4a**–**4e**) and linear alkoxymethyl (**8a** and **8b**) substituents. (**D**) Bioconcentration potential of all FILs—predicted log BCF values of all obtained FILs, with shaded ranges indicating low to medium, and high risk of bioconcentration in midtrophic level organisms.

The trends observed in part C and D of Figure 1 offer a valuable foundation for interpreting structure–property relationships, particularly in relation to phytotoxicity. In light of the observed differences in phytotoxicity between the (1*R*,2*S*,5*R*)-(−)-menthol-derived (**4a**–**4e**) and 1-decanol-derived (**8a**–**8b**) series, two key research questions have been formulated in the context of structural design (Scheme 2). The first question (Q1) addresses the impact of fluorinated anions within menthol-based FILs, while the second question (Q2) concerns the effect of replacing the functional substituent of the cyclic menthol-based structure with the linear 1-decanol-based structure. Based on this conceptual framework, the aim of this section is to identify the physicochemical parameters most likely to explain the divergent phytotoxic responses of these two structural series, such as surface activity, solubility and lipophilicity. By linking these descriptors to toxicity end points, this data set not only supports the interpretation of Q1 and Q2, but also provides actionable guidance for the design of safer, more sustainable ILs.

#### 3.3.1 Environmentally Relevant Physicochemical Properties of FILs (**4a–4e** and **8a–8b**): From Solubility to Bioaccumulation

##### 3.3.1.1 Solubility of FILs in Various Organic Solvents

To explore the relationship between structure and solubility, we evaluated the solubility of synthesized FILs containing either a (1*R*,2*S*,5*R*)-(−)-menthoxymethyl or 1-decyloxymethyl substituent (compounds **4a**–**4e** and **8a**–**8b**) in a selection of organic solvents, classified using empirical parameters for solvent polarity (see Table 1).^95^ This classification is based on the polarity index (*P*′), reflecting overall solvent strength, and selectivity parameters that describe the ability of solvents to engage in hydrogen bonding and dipolar interactions. These parameters collectively describe the general polarity and selectivity of solvent–solute interactions. Taken together, these descriptors provide a quantitative basis for linking solute structural features to solubility across solvents that differ in polarity, hydrogen-bonding capacity, and dipolar interactions. All solubility tests were performed following a standardized protocol established by Vogel et al.,^96^ which improves procedural consistency and facilitates comparison across the compounds examined in this study. In addition to FILs (compounds **4a**–**4e** and **8a**–**8b**), the study included their chloride precursors (compounds **3** and **7**) as well as the fluorinated alkali metal salts used in anion exchange reactions. This inclusion provides a broader context for interpreting solubility trends. Initially, the discussion focuses on a comparative analysis within FILs series, highlighting the structural influence of the cationic substituent [(1*R*,2*S*,5*R*)-(−)-menthoxymethyl *vs* 1-decyloxymethyl] and the identity of the anion (e.g., sulfonates *vs* sulfonylimides) on solubility behavior (Table 1).

These findings are essential for understanding how molecular architecture governs physicochemical properties relevant to solvent compatibility, environmental mobility, and potential partitioning phenomena. The latter part of this section briefly addresses solubility differences between the FILs and their chloride and alkali metal salt precursors to highlight the pronounced impact of IL formation on solvent interaction profiles (Table 1).

##### 3.3.1.2 Solubility of FILs in Various Organic Solvents—Internal Comparison

The investigated FILs exhibited a similar solubility profile across the solvent set. All compounds (**4a**–**4e** and **8a**–**8b**) were completely insoluble in nonpolar alkanes, such as *n*-hexane, *n*-heptane and *n*-octane, even upon heating. This is illustrated in Table 1 using *n*-hexane as an example. This behavior is consistent with the amphiphilic nature of ILs and the absence of favorable interactions—such as hydrogen bonding, ion–dipole, or π–π interactions—in purely hydrophobic media. Consequently, ILs are typically insoluble in aliphatic hydrocarbons such as *n*-hexane; this behavior is well documented for imidazolium-, ammonium-, and pyridinium-based ILs across different ionic compound types including fluorinated anions.^97^ In contrast, all tested FILs demonstrated good to excellent solubility in highly polar solvents, such as methanol, acetonitrile, acetone, and dimethyl sulfoxide (DMSO), as well as complete solubility at room temperature in moderately polar solvents such as tetrahydrofuran (THF), ethyl acetate (EtOAc) and chloroform (CHCl_3_). This indicates that the tested polar and moderately polar solvents have sufficient polarity and hydrogen-bond acceptor capability to solvate both ions in the FILs, regardless of specific cation or anion structure. The observed solubility is primarily due to ion–dipole and dipole–dipole interactions. In oxygen-containing solvents (e.g., acetone, EtOAc, THF and DMSO), additional stabilizing interactions arise from weak hydrogen bonds formed between the electron-rich sites of the solvent and the fluorinated substituents present in the FILs. This combination of interactions effectively counteracts the effects of steric hindrance and is consistent with established mechanisms reported in the literature.^98^

A comparison of the two series—the (1*R*,2*S*,5*R*)-(−)-menthol-derived (**4a** and **4b**) and the 1-decanol-derived series (**8a** and **8b**)—revealed highly similar solubility profiles across most solvents. Interestingly, only slight differences were observed for salts with perfluorobutanesulfonate [PFBS]^−^ anions: compound [Men-Im-C_1_]­[PFBS] (**4b**) exhibited limited solubility at room temperature in both toluene and *iso*-propanol, requiring an elevated temperature for complete dissolution. In contrast, its structural analogue **8b** (which bears the same anion but a 1-decyl substituent) was fully soluble under the same conditions. These solubility differences are likely due to the branched, rigid (1*R*,2*S*,5*R*)-(−)-menthol in **4b**, which limits dispersive and dipole–induced interactions in solvents of intermediate polarity with low hydrogen-bonding capacity.

Despite the broad emphasis in the literature on the critical role of anion identity in modulating the solubility of ILs,^37,76,99–101^ the present results reveal a notably consistent solubility pattern across the tested FILs. This suggests that, despite their structural diversity, the selected set of anions shares physicochemical characteristics that are sufficient to ensure solubility in both polar and moderately polar organic solvents. Indeed, almost all the compounds exhibited similar solubility behavior, with only a few isolated deviations. Compound [Men-Im-C_1_]­[TFSI] (**4c**) was insoluble in ethanol and *n*-propanol, whereas all the other salts were perfectly soluble in these solvents. Another minor difference is that the (1*R*,2*S*,5*R*)-(−)-menthol-based FILs bearing the anions [PFBS]^−^ (**4b**), [TFSI]^−^ (**4c**) and [OTF]^−^ (**4e**) exhibited limited solubility in toluene, in contrast to the full solubility observed for the other investigated FILs. These minor variations likely stem from subtle differences in steric bulk and interaction capacity with solvents of intermediate polarity.

##### 3.3.1.3 Solubility of FILs in Various Organic Solvents—A Comparison with Their Chloride Precursors and Fluorinated Alkali Metal Salts

Comparing the solubility profiles of the synthesized FILs with those of their chloride precursors (**3** and **7**) and alkali metal salts (KTFES, KPFBS, LiTFSI, LiPFSI and NaOTF) demonstrates the substantial impact of ion pair structure on solubility behavior. As shown in Table 1, all of the FILs (**4a**–**4e** and **8a**–**8b**) exhibited substantially broader solubility across a wide range of organic solvents than their respective precursors. Chloride salts **3** and **7** were only slightly soluble in aprotic solvents and were completely insoluble in low-polarity media. In contrast, the corresponding FILs were soluble in most polar and moderately polar solvents at room temperature. This difference can be attributed to the enhanced ion delocalization and reduced lattice energy of the FILs, as well as the increased dispersibility conferred by their bulky, often fluorinated, anions. Notably, chloride precursors were completely insoluble in toluene, even at elevated temperatures, and only partially soluble in THF. By contrast, all of the corresponding FILs were fully soluble under the same conditions. Compound **3** remained insoluble in ethyl acetate, even when heated, whereas compound **7** was only partially soluble at elevated temperatures—highlighting the clear solubility improvement of the FIL analogues compared to their chloride counterparts. These differences are likely due to the stronger ionic character and higher lattice energy of the chloride salts, as well as the absence of fluorinated anions or solubilizing alkyl chains.

The fluorinated alkali metal salts exhibited distinctly different solubility patterns in comparison to their FIL analogues. As expected, all of the metal salts tested were completely insoluble in nonpolar solvents such as *n*-hexane and toluene. Among the sulfonates, KTFES and KPFBS were completely insoluble in moderately polar solvents including THF, EtOAc, CHCl_3_ and dichloromethane (DCM), in contrast to their FIL analogues. A notable difference was also observed in *iso*-propanol, where these two sulfonates showed limited solubility, in contrast to the corresponding FILs, which were fully soluble. The observed limited solubility of KTFES and KPFBS in *iso*-propanol may result from the solvent’s lower hydrogen-bond donor efficiency and weaker solvation capacity for large alkali metal cations, which are poorly stabilized due to steric hindrance effects inherent to secondary alcohols. The smallest sulfonate, NaOTF, showed solubility in THF and EtOAc. Despite the high degree of charge delocalization and reduced lattice energy of sulfonylimides: LiTFSI and LiPFSI, which facilitates their solubility in moderately polar solvents such as THF, EtOAc and CHCl_3_, they remain insoluble in nonpolar solvents such as DCM. This enhanced solubility is likely due to significant ion–dipole interactions. Nevertheless, they remained insoluble in a low-to-moderate-polarity chlorinated solvent—DCM, consistent with the general behavior of tested alkali metal salts.

These observations highlight that molecular architecture and ionic composition are key factors controlling solubility across solvents of different polarity, directly affecting processing performance and environmental behavior. In light of the growing regulatory focus on ionic species containing fluorinated components, distinctions in solubility profiles may have implications for their mobility, persistence, and potential environmental impact—all of which are critical considerations for materials designed for use in chemically intensive or open-system applications.

##### 3.3.1.4 Solubility of FILs in Water

The solubility of ILs in water plays a crucial role in evaluating their environmental behavior, including their mobility in ecosystems, their potential bioavailability, and their phytotoxicity. Detailed solubility and surface tension data for the investigated compounds are provided in Table 1 and Figure 1A. In the present study, FILs bearing sulfonate anions [PFBS]^−^, [TFES]^−^ and [OTF]^−^, and sulfonylimide anions [TFSI]^−^ and [PFSI]^−^ (**4a**–**4e**, **8a**, **8b**) were found to be practically insoluble in water, even after prolonged stirring and mild heating. By contrast, the precursor chloride salts (compounds **3** and **7**) and the alkali metal salts (Li^+^, Na^+^, K^+^) used in the metathesis step showed full solubility in water (see Table 1). This is consistent with the classic ionic nature of these species and the strong hydration of both the cations and anions. The pronounced drop in water solubility observed after the anion exchange confirms the decisive role of the hydrophobic fluorinated anions, which, in combination with the imidazolium cation, limit ion dissociation and promote ion pairing and nanosegregation. This behavior has been widely reported in theoretical and modeling studies, including COSMO-RS, QSPR and molecular dynamics simulations.^98,102^

##### 3.3.1.4.1 Effect of Anion Structure

For the series of (1*R*,2*S*,5*R*)-(−)-menthoxymethyl-based FILs (**4a**–**4e**), a systematic variation in water solubility was observed as a function of the fluorinated anion used (Figure 1A). Measured water solubility limits ranged over 2 orders of magnitude—from 1.2·10^–2^ mol·dm^–3^ for [Men-Im-C_1_]­[OTF] (**4e**) to 1.9·10^–4^ mol·dm^–3^ for [Men-Im-C_1_]­[PFSI] (**4d**) (Figure 1A). This large variation underscores the decisive influence of anion identity on aqueous compatibility. In particular, (*i*) the sulfonates [OTF]^−^ and [TFES]^−^ (**4e** and **4a**) exhibited the greatest solubility, exceeding 10^–2^ mol·dm^–3^; (*ii*) the sulfonylimide [TFSI]^−^ (**4c**) showed intermediate solubility at 4·10^–3^ mol·dm^–3^; (*iii*) among the tested series, the perfluorobutanesulfonate [PFBS]^−^ (**4b**) and bis­(perfluoroethylsulfonyl)­imide [PFSI]^−^ (**4d**) derivatives exhibited the lowest solubility, with water solubility limits of 1.4·10^–3^ and 1.9·10^–4^ mol·dm^–3^, respectively. Notably, **4d** was almost an order of magnitude less soluble than **4b**, indicating substantially stronger hydrophobicity. These trends only partially correlate with the classical sulfonate vs sulfonylimide classification—indicating that specific structural features (e.g., length and degree of fluorination) can dominate over general class descriptors. Notably, [Men-Im-C_1_]­[PFBS] (**4b**), being a sulfonate, exhibited lower solubility than its sulfonylimide analogue [Men-Im-C_1_]­[TFSI] (**4c**). The observed solubility differences do not fully align with the conventional classification of anions as either sulfonates (e.g., [OTF]^−^, [TFES]^−^, [PFBS]^−^) or sulfonylimides (e.g., [TFSI]^−^, [PFSI]^−^). This outcome reflects the role of specific structural features, such as chain length, the number of fluorinated carbons, symmetry, and charge delocalization, which exert a stronger influence on hydration capacity and cohesive interactions than the anion class label alone. As discussed by Nordness and Brennecke in their comprehensive review of ionic interactions fluorinated anions introduce substantial entropic and enthalpic barriers to hydration, and their bulky, delocalized charge distribution can promote persistent ion pairs in aqueous environments.^98^ In this context, the long perfluorobutyl chain in PFBS significantly lowers its affinity for water, explaining its reduced solubility despite its classification as a sulfonate.

##### 3.3.1.4.2 Effect of Cation Structure

A comparison of cation structures further highlights the amphiphilicity effect. Switching from a compact cycloalkyl group (menthol) to a linear chain (decyl) decreased solubility for the same anion: (*i*) for [TFES]^−^: **4a** = 1.1·10^–2^ mol·dm^–3^, *vs* **8a** = 1.0·10^–2^ mol·dm^–3^ (virtually unchanged); (*ii*) for [PFBS]^−^: **4b** = 1.4·10^–3^ mol·dm^–3^, *vs* **8b** = 7.5·10^–4^ mol·dm^–3^ (almost 2-fold drop). This supports the interpretation that increasing alkyl chain length and linearity leads to greater hydrophobicity and reduced affinity for water, at least for [PFBS]^−^ salts. This trend is further corroborated by the log *K*_OW_ values discussed in the following section.

To further evaluate the compatibility of FIL with water, the surface tension (γ) was measured at the point of saturation solubility for each ionic compound (Figure 1A). The resulting values were clearly dependent on anion identity, but not directly correlated with solubility limits, indicating that surface activity and water compatibility reflect different molecular phenomena. For example, [Men-Im-C_1_]­[PFBS] (**4b**) exhibited a γ of 33.7 mN·m^–1^ at a solubility of only 1.4·10^–3^ mol·dm^–3^, whereas [Men-Im-C_1_]­[TFES] (**4a**) showed a higher γ of 38.5 mN·m^–1^ despite being nearly 1 order of magnitude more soluble. Such discrepancies highlight that interfacial behavior cannot be inferred directly from bulk aqueous compatibility. This has also been noted in previous studies on ILs with amphiphilic structures and fluorinated anions^103^ Among the (1*R*,2*S*,5*R*)-(−)-menthol-substituted FILs (**4a**–**4e**), sulfonate-based salts yielded lower γ values in the order: [PFBS]^−^ (**4b**): 33.7 mN·m^–1^, [TFES]^−^ (**4a**): 38.5 mN·m^–1^, [OTF]^−^ (**4e**): 40.6 mN·m^–1^. In contrast, sulfonylimide-based salts resulted in significantly higher surface tension: [TFSI]^−^ (**4c**): 50.1 mN·m^–1^, [PFSI]^−^ (**4d**): 55.0 mN·m^–1^. These results suggest that, due to their large volume (639.63 Å^3^ and 706.11 Å^3^, respectively) and strong cohesive interactions, sulfonylimides hinder efficient interfacial adsorption and exhibit weaker surface activity. In contrast, the lower γ values observed for sulfonates, particularly [PFBS]^−^ salts, point to more efficient partitioning at the air–water interface despite their lower bulk solubility.

A comparison of the cationic structures further confirms the impact of amphiphilicity. For both [TFES]^−^ (**4a** and **8a**) and [PFBS]^−^ (**4b** and **8b**), the (1*R*,2*S*,5*R*)-(−)-menthoxymethyl-based cations gave consistently higher surface tension values than their linear decyl analogues: γ = 38.5 mN·m^–1^ for [Men-Im-C_1_]­[TFES] (**4a**) *vs* 28.0 mN·m^–1^ for [C_10_-Im-C_1_]­[TFES] (**8a**); For [PFBS]^−^, the values were 33.7 mN·m^–1^ for [Men-Im-C_1_]­[PFBS] (**4b**) and 28.0 mN·m^–1^ for [C_10_-Im-C_1_]­[PFBS] (**8b**). This observation is consistent with reports in the literature that surface tension decreases with increasing linear alkyl chain length, while branched or cyclic substituents, such as isobutenyl or phenyl group, reduce interfacial packing efficiency.^103^ This pattern demonstrates that long, flexible alkyl chains enhance interfacial adsorption more efficiently than rigid or bulky moieties such as menthol, thereby lowering surface tension. Therefore, the structure of both the anion and the cation contribute independently to surface properties, which are not necessarily aligned with aqueous solubility.

These results clearly demonstrate that the aqueous solubility of FILs is governed by specific molecular features of both the cation and anion at a molecular level, which influence hydration thermodynamics more profoundly than gross class distinctions (sulfonate *vs* sulfonylimide). Along with predicted partition coefficients and bioaccumulation factors, these properties form a foundation for evaluating the environmental mobility and phytotoxicity potential of novel FILs.

##### 3.3.1.5 Lipophilicity of FILs: Predicted log *K*_OW_ Values and Structural Implications

The *n*-octanol–water partition coefficient (log *K*_OW_) provides a valuable descriptor of the lipophilic character of ILs and is widely used to predict their environmental fate, membrane affinity, and accumulation potential. In this section, log *K*_OW_ values were predicted using the QSAR Toolbox software for a series of FILs bearing either cyclic ((1*R*,2*S*,5*R*)-(−)-menthol-based, **4a**–**4e**) group or linear (1-decanol-based, **8a**–**8b**) side chain. The results are visualized in Figure 1C. These data are examined further in relation to both anion and cation structure, and considered alongside experimental water solubility limits to assess how lipophilicity is governed by molecular architecture.

##### 3.3.1.5.1 Effect of Anion Structure

For (1*R*,2*S*,5*R*)-(−)-menthol-based series (**4a**–**4e**), predicted log *K*_OW_ values varied by almost an order of magnitude, ranging from 0.527 for [Men-Im-C_1_]­[TFES] salt (**4a**) to 4.43 for [Men-Im-C_1_]­[PFSI] salt (**4d**). The general trend in lipophilicity followed the order: [TFES]^−^ < [OTF]^−^ < [TFSI]^−^ < [PFBS]^−^ < [PFSI]^−^. This sequence reflects the cumulative influence of specific structural features, including partial increases in fluorination and molecular volume (see Figure 1A), as well as anion charge delocalization. These features are known to reduce hydration enthalpy and enhance affinity to the organic phase, providing a explanation for the progressive increase in log *K*_OW_ across the series. This is consistent with the earlier findings of Vieira et al.,^97^ who demonstrated that the size and topology of the fluorinated anionic domain are key factors in determining lipophilicity and membrane-related activity in FILs. Similarly, Hodges et al.^104^ found that increasing the length of the perfluoroalkyl chain leads to higher log *K*_OW_ values, primarily due to reduced hydration and an increased van der Waals surface area.

Nevertheless, when comparing [TFES]^−^ and [OTF]^−^, a deviation from a strict polarity-based trend is evident. Although [OTF]^−^ is structurally less fluorinated, its predicted log *K*_OW_ (1.33 for **4e**) is higher than that of [TFES]^−^ (0.527 for **4a**). However, their water solubility limits are almost identical (0.012 *vs* 0.011 mol·dm^–3^), suggesting that simple polarity- or functional class-based measures are inadequate for explaining the observed differences. Consequently, factors such as conformational flexibility, molecular symmetry and the presence of hydration-active sites may influence solvation and partitioning behavior in ways more complex than those captured by standard fluorination-based descriptors.^97^

Among the most hydrophobic (1*R*,2*S*,5*R*)-(−)-menthol-based salts tested, [PFBS]^−^ (**4b**) and [PFSI]^−^ (**4d**) exhibited the highest log *K*_OW_ values (3.63 and 4.43, respectively), consistent with their low water solubility and large molecular volumes (645 and 706 Å^3^, respectively; Table 2). These findings reflect the influence of extended perfluoroalkyl chains, which enhance hydrophobicity by increasing steric bulk and suppressing hydration *via* strong cohesive interactions. Such structural traits have been previously associated with enhanced membrane affinity and resistance to biodegradation in perfluorinated anions.^97^

##### 3.3.1.5.2 Effect of Cation Structure

A systematic increase in predicted log *K*_OW_ was observed within both the [TFES]^−^ and [PFBS]^−^ series upon replacing the cyclic (1*R*,2*S*,5*R*)-(−)-menthyl group with a linear 1-decyl moiety. Specifically, [Men-Im-C_1_]­[TFES] (**4a**) had a log *K*_OW_ value of 0.527, whereas its decyl analogue, [C_10_-Im-C_1_]­[TFES] (**8a**), had a slightly higher value of 0.933. Similarly, for the [PFBS]^−^ pairs, log *K*_OW_ increased from 3.63 (**4b**) to 4.04 (**8b**). This trend is consistent with the recognized effect of increasing the length of the alkyl chain on the lipophilicity of imidazolium-based ILs, whereby linear and flexible substituents enhance affinity with organic phases due to improved van der Waals interactions and reduced steric hindrance.^105^ The increase in log *K*_OW_ upon switching to a linear chain also correlates with reduced water solubility, decreasing from 1.4·10^–3^ mol·dm^–3^ (**4b**) to 7.5·10^–4^ mol·dm^–3^ (**8b**) for the [PFBS]^−^ derivatives and from 1.1·10^–2^ mol·dm^–3^ (**4a**) to 1.0·10^–2^ mol·dm^–3^ (**8a**) for the [TFES]^−^ derivatives. These results confirm that both chain length and topological flexibility in the cation significantly influence hydrophobicity and aqueous compatibility, consistent with prior findings for fluorinated imidazolium ILs.^97^

Taken together, these results confirm that the lipophilicity of FILs is not solely governed by anion class (sulfonate *vs* sulfonylimide), but by specific structural attributes such as fluorine content, spatial extension and electrostatic delocalization. These trends are consistent with those observed in other fluorinated surfactants and FILs reported in the literature, where log *K*_OW_ was shown to correlate with fluorinated chain length and branching pattern, particularly when micellar or aggregate formation is inhibited.^97,104^

##### 3.3.1.6 Bioaccumulation Potential: Insights from in Silico log BCF Estimations

Bioconcentration factor (BCF) is a key ecotoxicological parameter used to evaluate the tendency of chemicals to accumulate in living organisms after they are exposed to them through water. It plays a central role in regulatory frameworks such as REACH (Registration, Evaluation and Authorisation of Chemicals) and ECHA (European Chemicals Agency), and is typically predicted using QSAR-based models when experimental data is unavailable. Here, log BCF values were predicted with the QSAR Toolbox (OECD/ECHA-endorsed) software, for all synthesized FILs (**4a**–**4e** and **8a**–**8b**) and analyzed in the context of structural features and lipophilicity (Figure 1D).

##### 3.3.1.6.1 Effect of Anion Structure

Among the menthol-based salts (**4a**–**4e**), the log BCF values ranged widely, from 0.064 for [TFES]^−^ (**4a**) to 3.23 for [PFSI]^−^ (**4d**). The trend in bioaccumulation risk followed the same general order as that observed for lipophilicity: [TFES]^−^ < [OTF]^−^ < [TFSI]^−^ < [PFBS]^−^ < [PFSI]^−^. This consistency supports the role of log *K*_OW_ as a predictor of bioaccumulation behavior in this system. FILs bearing anions with longer, more fluorinated chains (e.g., [PFBS]^−^, [PFSI]^−^) exhibit elevated log *K*_OW_ and log BCF values, indicating an increased potential for passive diffusion into lipid-rich biological compartments. The high log BCF value of **4d** (3.23) lies just below the REACH threshold of log BCF > 3.3, indicating a potential for high bioaccumulative behavior under EU guidance criteria.^104^ Although [PFBS]^−^ (**4b**) and [TFSI]^−^ (**4c**) belong to different anion classes, the former displayed a higher log BCF value (2.46 *vs* 1.35). This highlights that the potential for accumulation is better explained by detailed structural attributes than by general anion classification.

##### 3.3.1.6.2 Effect of Cation Structure

A consistent increase in log BCF was observed in both the [TFES]^−^ and [PFBS]^−^ series upon switching from the cyclic (1*R*,2*S*,5*R*)-(−)-menthol moiety to a linear 1-decyl chain. For the [TFES]^−^ salts, log BCF increased from 0.064 for [Men-Im-C_1_]­[TFES] (**4a**) to 0.179 for [C_10_-Im-C_1_]­[TFES] (**8a**). A similar trend was observed for the [PFBS]^−^ derivatives, with values of 2.46 for **4b** and 2.84 for **8b**. This pattern is consistent with earlier findings for log *K*_OW_ (Section 3.3.1.5) and reflects the stronger lipophilic character and membrane partitioning propensity of linear alkyl substituents. It also corroborates previous observations that cation topology, particularly the presence of flexible hydrophobic chains, significantly contributes to the accumulation potential of imidazolium-based ILs.^97^

Overall, the predicted log BCF values confirm that cation flexibility and anion fluorination both have a significant impact on the bioaccumulation potential of FILs. (1*R*,2*S*,5*R*)-(−)-Menthol-based derivatives generally exhibited lower accumulation potential than their decyl analogues, while the [TFES]^−^ and [OTF]^−^ anions were associated with the lowest bioaccumulation risk. These findings, considered together with the lipophilicity and solubility data, offer a coherent basis for assessing the environmental mobility, persistence, and bioaccumulation potential of FILs, thereby informing their risk evaluation under relevant regulatory frameworks.

#### 3.3.2 Thermal Properties of Obtained FILs (**4a–4e**, **8a–8b**)

The thermal behavior of FILs is a key determinant of their physicochemical profile and can indirectly affect their environmental interactions, including phytotoxic responses. In the context of structure–activity relationships, thermal characteristics such as melting point (*T*_m_), phase transitions and thermal stability (*T*_d_) can provide insights into molecular organization, anion–cation interactions and potential aggregation phenomena. These features are particularly relevant when considering the incorporation of bulky or rigid substituents, such as (1*R*,2*S*,5*R*)-(−)-menthoxymethyl or linear 1-decyloxymethyl groups, into the imidazolium framework, since these substituents may impact molecular packing, glass transition behavior or crystallinity.

From an environmental perspective, the thermal stability of FILs determines their persistence in different temperature conditions and may also be related to their environmental safety profiles. For example, FILs with high decomposition temperatures can resist abiotic degradation, whereas phase transitions, such as crystallization or glass formation, can affect solubility and bioavailability in soil matrices. Furthermore, the observed differences in thermal parameters between the (1*R*,2*S*,5*R*)-(−)-menthol- and 1-decanol-derived series may reflect the degree of structural organization or disruption, which could affect their interaction with biological membranes or plant tissues.

In the present study, the thermal profiles of the synthesized FILs (**4a**–**4e** and **8a**–**8b**) were evaluated using thermogravimetric analysis (TGA) and differential scanning calorimetry (DSC), allowing for comparison of phase transition behavior and thermal decomposition resistance across both structural series. The experimental data on melting points, decomposition temperatures and phase transitions are summarized in Table 2. Representative TGA thermograms are provided in the Supporting Information (Figures S1–S3), while DSC thermograms of individual compounds are shown in Figures S4–S10.

A comparison of the *T*_5%onset_ values of the chloride precursors (**3**, **7**) (see Table S1) with those of the studied FILs (**4a**–**4e**; **8a**–**8b**) (see Table 2) shows that introducing a fluorinated anion into the FIL structure significantly enhances its thermal stability. The TG results indicate that all of the studied FILs exhibit good thermal stability, with a 5% mass loss temperature (*T*_5%onset_) above 184 °C. [Men-Im-C_1_]­[PFSI] (**4d**) and [Men-Im-C_1_]­[TFSI] (**4c**) demonstrate the highest thermal stability with *T*_5%onset_ values of 223 and 207 °C, respectively. The thermal stability of the studied FILs increases according to the type of anion. For the (1*R*,2*S*,5*R*)-(−)-menthol-based FILs (**4a**–**4e**): [Men-Im-C_1_]­[OTF] (**4e**, 184.94 °C) < [Men-Im-C_1_]­[PFBS] (**4b**, 197.25 °C) < [Men-Im-C_1_]­[TFES] (**4a**, 203.90 °C) < [Men-Im-C_1_]­[TFSI] (**4c**, 207.56 °C) < [Men-Im-C_1_]­[PFSI] (**4d**, 223.37 °C). This trend is similar to that observed for chiral *N*-(2-hydroxyethyl)-pyrrolidinium ILs incorporating a (1*R*,2*S*,5*R*)-(−)-menthol moiety, as reported by Gano et al.,^106^ for which the thermal stability increases as follows: [Men-Pyrr-C_2_OH]­[OTF] (190.0 °C) < [Men-Pyrr-C_2_OH]­[PFBS] (192.1 °C) < [Men-Pyrr-C_2_OH]­[TFSI] (214.4 °C) < [Men-Pyrr-C_2_OH]­[PFSI] (220.4 °C). Among the salts with linear alkoxymethyl substituents, compound **8b** ([PFBS]^−^) is more thermally stable than **8a** ([TFES]^−^), with *T*_5%onset_ values of 198.81 and 188.14 °C, respectively. The relationship between the thermal stability and the structure of fluorinated anions sharing the same imidazolium cation is consistent with previous literature reports. In general, the thermal stability tends to increase with increasing anion size.^107,108^

When the difference in the cationic structure is considered, [Men-Im-C_1_]­[TFES] (**4a**) appears to be more stable, with a *T*_5%onset_ at 203 °C, whereas [C_10_-Im-C_1_]­[TFES] (**8a**) starts decomposing at 188 °C. Based on this comparison, it can be suggested that the presence of the cyclic and more complex (1*R*,2*S*,5*R*)-(−)-menthol moiety in the cationic structure may result higher thermal stability than that of an equivalent FIL substituted with a decyl chain. The same tendency is observed for their chloride precursors, [Men-Im-C_1_]­[Cl] (**3**) appeared to be more stable than [C_10_-Im-C_1_]­[Cl] (**7**), with *T*_5%onset_ values of 115.90 °C and 103.84 °C, respectively. For FILs with the [PFBS]^−^ anion, the thermal stability shows an opposite trend; however, the *T*_5%onset_ difference between [Men-Im-C_1_]­[PFBS] (**4b**, 197.25 °C) and [C_10_-Im-C_1_]­[PFBS] (**8b**, 198.81 °C) is minor (Δ*T*_5%onset_ ≈ 1.5 °C), with slightly higher stability of the latter. These results support and extend previous studies,^109,110^ which indicate that both the cation and the anion influence the thermal stability of FILs, with the anion playing a predominant role.^111^ Our findings additionally highlight that the cation structure also contributes significantly to this property.

For all the obtained FILs—[Men-Im-C_1_]­[TFES] (**4a**), [Men-Im-C_1_]­[PFBS] (**4b**), [Men-Im-C_1_]­[TFSI] (**4c**), [Men-Im-C_1_]­[PFSI] (**4d**), [Men-Im-C_1_]­[OTF] (**4e**), [C_10_-Im-C_1_]­[TFES] (**8a**) and [C_10_-Im-C_1_]­[PFBS] (**8b**)—a two-step decomposition was observed. The first step of decomposition and the associated mass loss for the (1*R*,2*S*,5*R*)-(−)-menthol-based FILs (**4a**–**4e**) indicates the breaking of the bond between the oxygen atom and the carbon atom adjacent to the imidazolium ring. Concerning the salts with the linear alkoxymethyl group (**8a** and **8b**), the bond between the decyl chain and oxygen atom breaks during the first step of the decomposition. The second decomposition step for all the studied FILs is caused by the anion hydrolysis.^106,112^

Three of the studied FILs are white solids below 100 °C: [Men-Im-C_1_]­[TFES] (**4a**), [Men-Im-C_1_]­[PFBS] (**4b**), and [Men-Im-C_1_]­[OTF] (**4e**). Their respective melting points are 59.29 °C, 86.17 °C, and 82.16 °C. Notably, compound **4a** is initially obtained as a colorless liquid immediately after synthesis and purification. After standing for a prolonged period, it gradually transforms into a white solid. When heated slightly above its melting point (DSC, *T*_m_ = 59 °C), this salt reverts to a liquid state and remains liquid. Similar behavior has been observed by Shiflett et al.^113^ and Harmer et al.^114^ for other imidazolium-based 1,1,2,2-tetrafluoroethanesulfonate ILs. In particular, the RTIL synthesized by these researchers, 1-ethyl-3-methylimidazolium-1,1,2,2-tetrafluoroethanesulfonate ([C_2_-Im-C_1_]­[TFES]), with melting points of 35 °C, and 45 °C,^113^ respectively, was also reported to solidify over time. In our laboratory, we have observed that the [Men-Im-C_1_]­[TFES] (**4a**) sample solidifies at room temperature after a prolonged period, e.g. several months. Such a transition was not observed on DSC scans, which represent a much shorter observation window. DSC measurements of an initially solid [Men-Im-C_1_]­[TFES] (**4a**) sample (Figure S4) show that **4a** remains liquid after melting during the first heating to 59 °C. No crystallization event is observed during subsequent cooling, with the sample remaining liquid throughout the second heating. Thus, no phase transitions are detected.

The DSC measurements of [Men-Im-C_1_]­[PFBS] (**4b**) show that it melts on the first heating at 86.17 °C, followed by crystallization at 34.61 °C on the first cooling. Similar behavior is observed during the second heating and cooling cycles (see Figure S5). The tendency to form crystals upon cooling and to have a distinct melting point upon heating is characteristic of the second type of the IL behavior and corresponds to structures that are good crystal formers.^115^ The trifluoromethanesulfonate salt, [Men-Im-C_1_]­[OTF] (**4e**), does not form crystals during either of the two cooling cycles. However, during the first heating cycle, a cold crystallization occurs at 38.17 °C, followed by melting at 83.18 °C. During the second heating [Men-Im-C_1_]­[OTF] (**4e**) first undergoes a glass transition at –14.10 °C. This is followed by what appear to be two cold crystallization events at 15.42 and 36.58 °C. These are then followed by melting at 82.53 °C (Figure S8). Cold crystallization is the transition from an amorphous phase to a partially crystalline solid, which typically occurs in amorphous materials such as polymers; however, it can also be observed in ILs.^115^ This type of phase transition is common in the third type of IL thermal behavior.^116,117^ Sulfonylimide salts containing the 1-[(1*R*,2*S*,5*R*)-(−)-menthol component, [Men-Im-C_1_]­[TFSI] (**4c**) and [Men-Im-C_1_]­[PFSI] (**4d**), remain in the liquid state at 25 °C (Figures S6 and S7). These two sulfonylimides represent the first type of IL thermal behavior.^118^ They appear to be glass-forming liquids, characterized by the formation of an amorphous glass and exhibit only glass transition temperatures at –39.28 °C and −36.79 °C, respectively. In this work, as well as in the previous studies, glass transitions are observed in both the cooling and heating cycles.^109,118^

Sulfonates containing the 1-decanol moiety, [C_10_-Im-C_1_]­[TFES] (**8a**) and [C_10_-Im-C_1_]­[PFBS] (**8b**), remain in the liquid state at 25 °C (Figures S9 and S10). They show a more complex behavior than the studied FILs containing the 1-[(1*R*,2*S*,5*R*)-(−)-menthol component. As a consequence, they cannot be classified according to the typical IL thermal behavior proposed by Gómez et al.^115^ The first heating from room temperature reveal no phase transitions. During the two cooling cycles of [C_10_-Im-C_1_]­[TFES] (**8a**), two exothermic events are observed, at −6.47 °C and −41.62 °C, respectively. During the second heating cycle, a glass transition is observed at −39.93 °C, followed by a precrystallization relaxation peak at −30.78 °C and, immediately afterward, cold crystallization at −25.98 °C. [C_10_-Im-C_1_]­[TFES] (**8a**) then undergoes complete melting at 25.54 °C to become room-temperature IL (RTIL). The fact that the IL undergoes a glass transition rather than melting on the second heating suggests that the two exothermic events during cooling might correspond to a transition from an isomorphous liquid phase to two subsequent liquid crystalline phases. Cold crystallization, although typical for polymers, is not unusual in ILs with long alkyl chain substituents, as demonstrated, inter alia, in our previous research on ammonium-based ILs with a decyl substituent.^109^ Transition between an isomorphic and a liquid crystalline phase have also been observed in hexadecyl-substituted imidazolium-based ILs.^119–121^ Similar type of thermal behavior is exhibited by [C_10_-Im-C_1_]­[PFBS] (**8b**). The first heating from room temperature does not reveal any phase transitions, with an exothermic event observed at 16.80 °C during both cooling cycles. During the second heating process, the studied FIL first undergoes a glass transition at −54.85 °C, followed by two exothermic events corresponding to cold crystallizations at −34.46 °C and −16.41 °C, after which melting temperature occurs at 25.25 °C. This unusual thermal behavior upon the heating and cooling cycles indicates a polymorphic character of [C_10_-Im-C_1_]­[PFBS] (**8b**), involving the formation of multiple crystalline phases. Such polymorphic behavior, including two cold crystallization events, has also been reported by Mezzetta et al.^122^ for methyltrioctylammonium oleate.

### 3.4 Phytotoxicity Characteristic of the Synthesized FILs

Any contaminant, including various types of chemical compounds used in agriculture, horticulture or industry, that finds its way into the soil will inevitably have an impact on soil organisms. Plants are particularly vulnerable to soil contamination and are unable to avoid exposure. Pollutants therefore have a direct impact on the entire development period of the plant, from germination to the end of its life. The negative impact of contaminants can manifest as inhibition of seed germination. After germination, plant roots are in constant contact with the soil and its constituents. At the same time as they take up water and mineral salts from the soil, plants also absorb various types of chemical compounds that affect their physiology, growth, and yield, with some compounds being absorbed and subsequently accumulated in the plant tissues.^123–126^ Therefore, in the present study, the effects of FILs on plant growth and development were evaluated.

The effects of the presence of the seven tested FILs in soil applied at different concentrations on radish seed emergence are presented in the Supporting Information, Table S4. The presence of the tested FILs in soil at concentrations up to 100 mg·kg^–1^ soil DW had no effect on radish seed germination. The application of concentrations of 400 mg·kg^–1^ soil DW and higher significantly reduced seed germination and thus plant emergence, with the higher the concentration applied, the lower the plant emergence. The greatest effect on radish emergence was shown by [Men-Im-C_1_]­[TFSI] (**4c**), which at concentrations of 400–1000 mg·kg^–1^ soil DW resulted in 100% inhibition of radish emergence. A similar inhibition of plant emergence with increasing IL dose was evidenced by Cruz et al.,^127^ who examined the effect of choline dihydrogen phosphate on the emergence rate of 8 plant species (*Eucalyptus globulus* Labill, *Pinus halepensis* Mill., *Pinus nigra* Arnold., *Pinus pinaster* Aiton., *Pinus sylvestris* L., *Pinus radiata* D. Don., *Avena sulcata* (Gay ex Boiss.) and *Daucus carota* L.). Similar conclusions were also reached by Chen et al.^128^ investigating, among other things, the effect of choline amino acid ILs on the germination strength of maize grain. Cruz et al.^127^ further emphasize that IL they tested affected seed germination of individual plants to varying degrees. However, they also reported that low concentrations of the tested IL not only did not inhibit seed germination, but at certain concentrations could stimulate seeds to germinate faster, possibly as a kind of protection against the negative effects of compounds present in the soil. However, the presence of higher concentrations of IL caused a decrease in seed germination.

Plant germination and emergence have a direct effect on plant root and shoot growth. Low concentrations of the FILs tested did not inhibit plant and root growth, and the presence of [C_10_-Im-C_1_]­[TFES] (**8a**) in soil at concentrations from 1 mg·kg^–1^ soil DW to 100 mg·kg^–1^ soil DW even resulted in faster radish root growth. However, as in the case of emergence, the presence of higher concentrations of FILs (400–1000 mg·kg^–1^ soil DW) in the soil significantly inhibited plant growth and root growth. It should be clearly emphasized that the factor that influenced the inhibition of radish shoot and root elongation was the chemical structure of the FILs used in the study. The presence of a natural component such as (1*R*,2*S*,5*R*)-(−)-menthol in the FILs molecule, in a certain range of concentrations, led to a lower phytotoxicity of these compounds, as clearly shown by comparing the effects of [Men-Im-C_1_]­[PFBS] (**4b**) and [C_10_-Im-C_1_]­[PFBS] (**8b**). In contrast, [Men-Im-C_1_]­[TFSI] (**4c**) showed the highest toxicity, causing 100% inhibition of plant shoot and root growth already at a concentration of 400 mg·kg^–1^ soil DW (Table S5).

The length of the plant shoots is reflected in the FW yield of the plants. Similar to the previously discussed phytotoxicity parameters, the effect of FILs on common radish yield was strongly dependent on the concentration used. Low concentrations of the tested FILs had no effect on the FW yield of common radish. In contrast, the application of higher concentrations of the tested compounds resulted in a significant reduction in FW yield. It is also evident that the compounds containing a cycloalkoxymethyl substituent showed less effect on yield, as a clear inhibition of FW yield of common radish was evident only after the application of [Men-Im-C_1_]­[TFES] (**4a**) and [Men-Im-C_1_]­[PFBS] (**4b**) at a concentration of 400 mg·kg^–1^ soil DW. For the counterparts of these FILs with linear alkoxymethyl component [C_10_-Im-C_1_]­[TFES] (**8a**) and [C_10_-Im-C_1_]­[PFBS] (**8b**), a clear decrease in the FW yield of radish plants was already observed at a concentration of 100 mg·kg^–1^ soil DW (Table S6).

Based on the obtained values for plant FW yield and shoot and root length inhibition, EC_50_ values were determined for all tested FILs (Figure 2 and Scheme 4). Scheme 4 demonstrates how phytotoxicity values change with differences in anion type (Q1) and with the change in functional substituent in the cation part (Q2) from cycloalkoxymethyl ((1*R*,2*S*,5*R*)-(−)-menthol-derived) to linear alkoxymethyl (1-decanol-derived). From the EC_50_ values obtained, it can be seen that the strongest effect of the tested compounds was the inhibition of FW yield of radish plants; moreover, the use of (1*R*,2*S*,5*R*)-(−)-menthol as a component in the construction of FILs led to a visible decrease in the phytotoxicity of these compounds. The calculated EC_50_ values, based on the inhibition of the FW yield of the plants and the inhibition of radish shoot and root elongation, indicate that the salt with the greatest negative effect on radish was [Men-Im-C_1_]­[TFSI] (**4c**).

**Figure 2:**
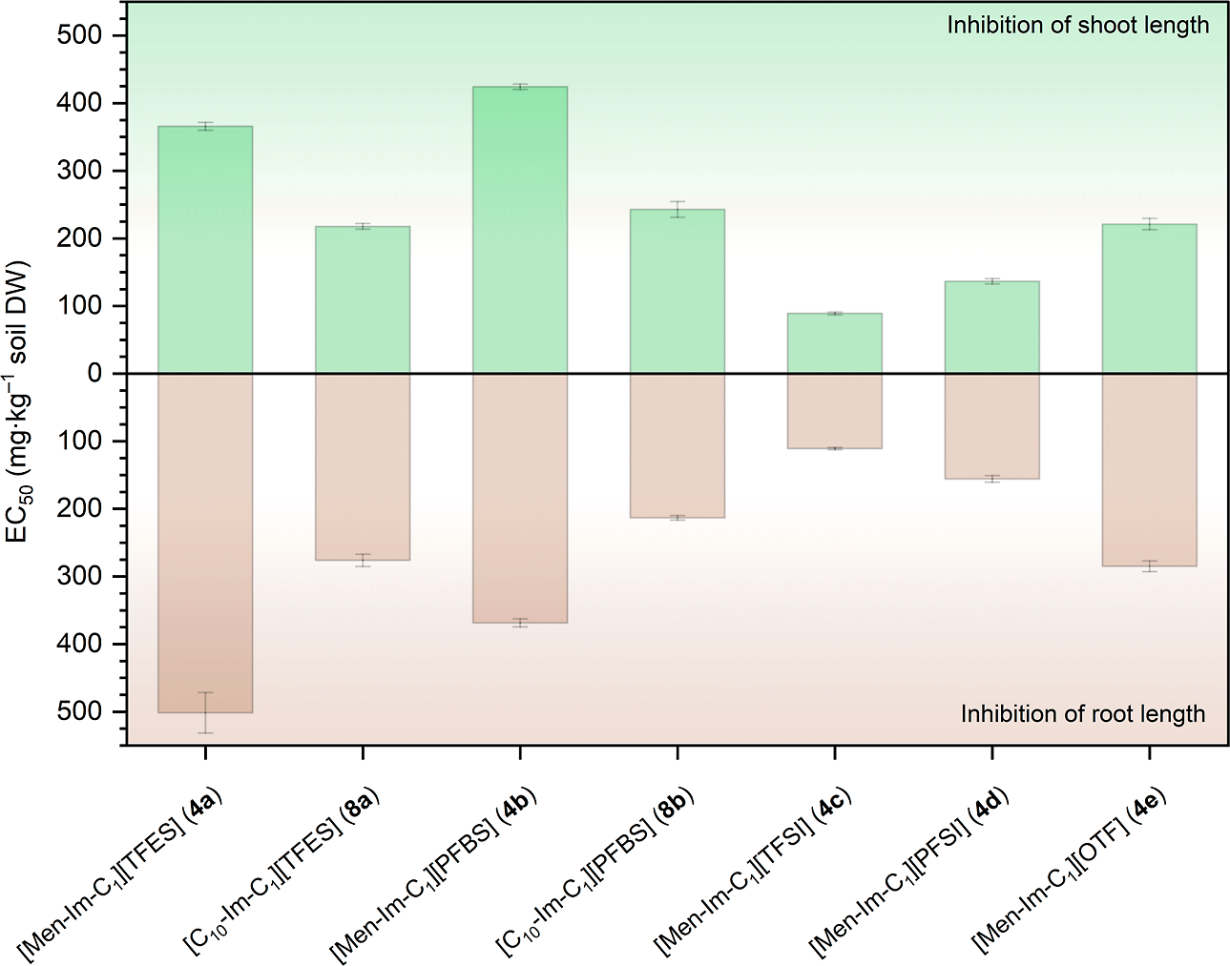
EC_50_ (mg·kg^–1^ soil dry weight, DW) values determined from inhibition of shoot length and root length for *R. sativus* growing in soil with the addition of FILs tested.

**Scheme 4:**
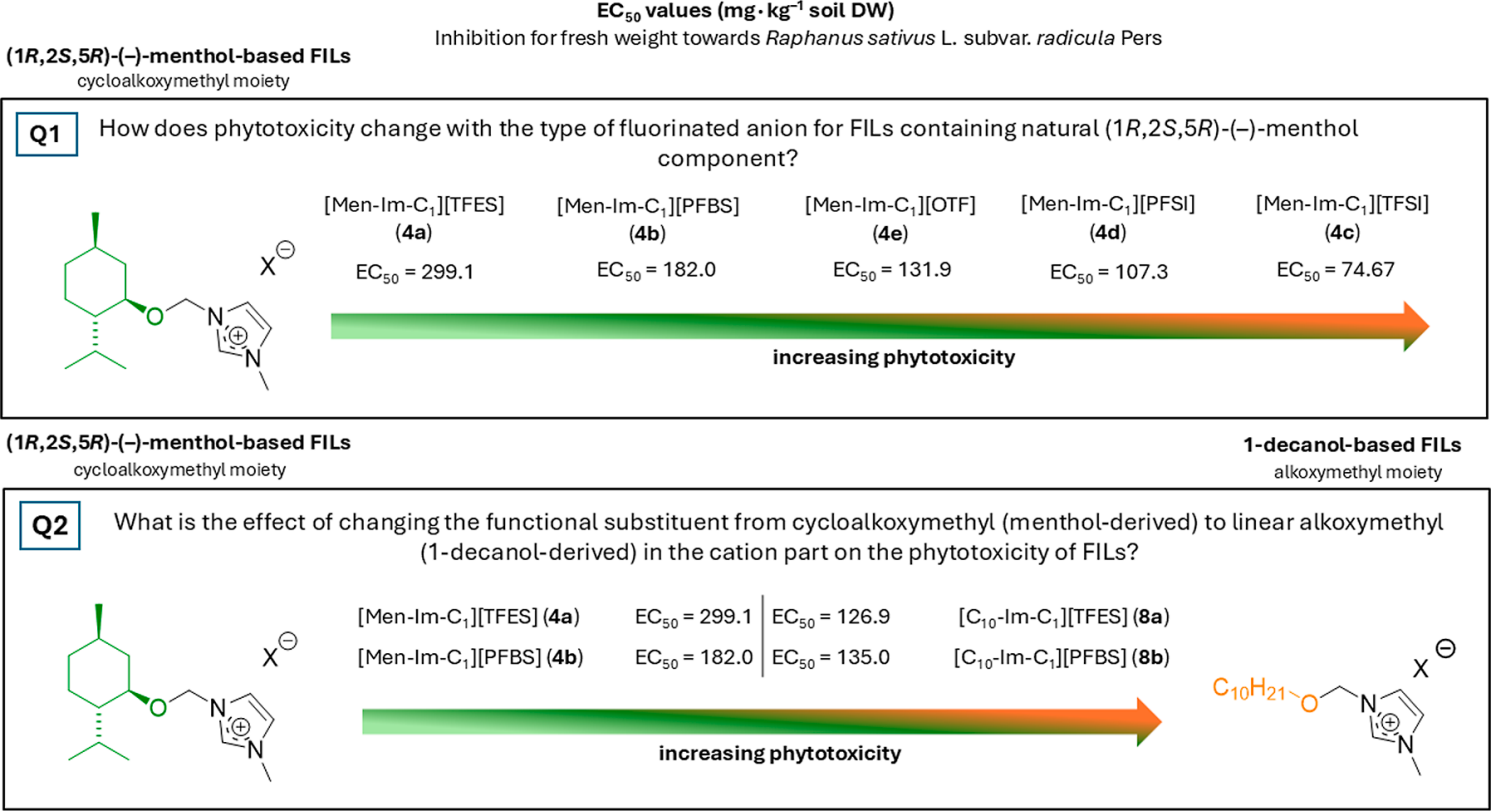
Experimental EC_50_ Values (mg·kg^–1^ Soil Dry Weight, DW; Inhibition for Fresh Weight) for FILs Based on 1-[(1*R*,2*S*,5*R*)-(−)-Menthoxymethyl]-3-methylmidazolium (**4a**–**4e**) and 1-Decyloxymethyl-3-methylimidazolium (**8a**–**8b**) Cations towards *R. sativus*

Inhibition of root length and aboveground parts are some of the most obvious and visible symptoms of phytotoxicity and signs of oxidative stress in plants. Low concentrations of ILs rarely show adverse effects on plants. Some ILs can also cause a hormesis effect in plants *i.e.,* at low concentrations they accelerate their growth and at high concentrations ILs exhibit phytotoxicity.^43,123,124^ Habibul et al.^129^ and Xu et al.^28^ demonstrated that ILs can be accumulated in various plant organs, with the highest presence usually observed in roots, which are in continuous contact with the substrate. However, the amount of compounds accumulated is dependent on the type of compound and the concentration used. Chapman et al.^130^ pointed out that the presence of contaminants in the substrate can lead to damage to the root cell wall. As a result of damage to this organ, toxins present in the soil can penetrate into the roots and readily reach other plant organs without much hindrance. Any damage to the root system and its improper development will have a direct impact on the growth and development of the entire plant and, consequently, on the yield obtained, since it is the root that holds the plant in the soil and is responsible for the uptake of water and nutrients.

Among the biomarkers for determining plant health is the DW content, which is also strongly related to plant yield. As was the case with the previously discussed phytotoxicity parameters, an effect of FILs on plant DW content was also observed here, especially after the application of higher concentrations of the compounds tested. High concentrations of FILs caused an increase in the dry matter content of radish plants (Table S7). The increase in dry matter content may have been due to the aforementioned effects of FILs on root development resulting in disruption of plant water management.^130^

#### 3.4.1 Photosynthetic Pigments Content in the Presence of FILs

The amount of yield obtained and the proper growth and development of plants are always closely linked to the photosynthesis. An essential part of the photosynthetic process is the content of photosynthetic pigments. Chlorophyll *a* (Chl a) and chlorophyll *b* (Chl *b*) are natural pigments responsible for absorbing and transmitting light energy. In addition to capturing and transmitting energy to the reaction center, carotenoids (Car) also play a key protective role by dissipating excess energy in the form of heat.^131–133^ As a result of the study, the effect of FILs with fluorinated anions on the content clearly indicate that all the FILs tested exerted an inhibitory effect on the photosynthetic pigment content of common radish. For the compounds tested, a progressive decrease in the assimilation pigment content in common radish leaves was observed, correlating with an increase in the concentration of the tested FILs in the soil (Figure 3 and Table S8). A decrease in the assimilatory pigment content of plants when exposed to ILs has also been reported by other authors.^132,134,135^ The decrease in photosynthetic pigments in plants may arise from several factors. ILs can cause disruption of the phospholipid double layer, thereby increasing membrane permeability, resulting in damage to chloroplast membrane structures through which chlorophyll can leak.^27,134–136^ Another reason for the decrease in chlorophyll content in plants grown on soils contaminated with ILs may be inhibition of chlorophyll biosynthesis. Some studies further indicate that the decrease in chlorophyll content may also result from demetalation, *i.e.*, removal of magnesium from the chlorophyll molecule by ILs, which consequently exerts an inhibitory effect on the entire photosynthetic process.^131,134,135^

**Figure 3:**
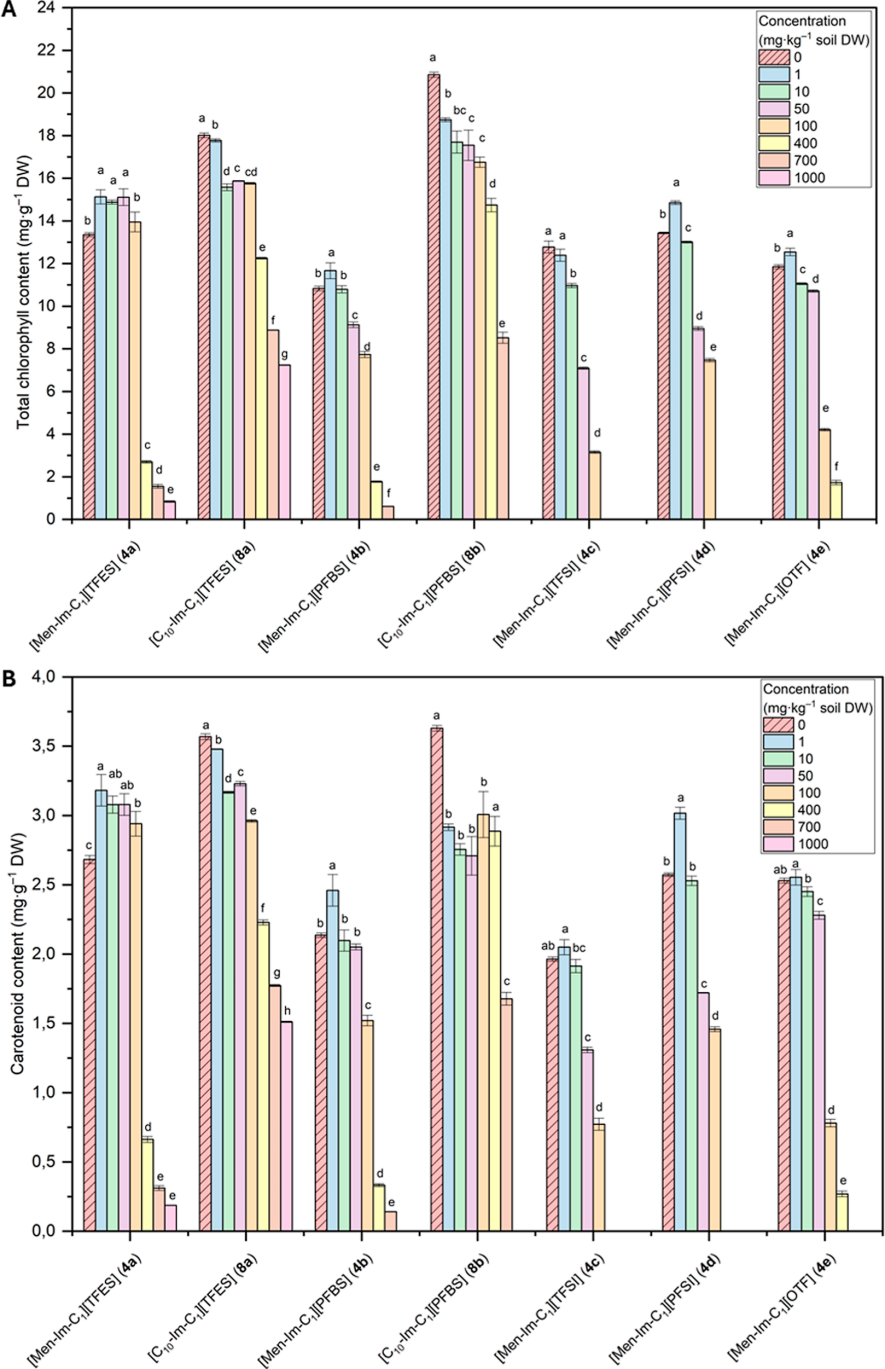
Physiological response of common radish to functionalized ionic liquids (FILs): (**A**) Total chlorophyll content (Chl *a* + Chl *b*) and (**B**) carotenoid content (Car) in common radish leaves growing on soil supplemented with FILs. ^a‑h^Values denoted by the same letters do not differ statistically at *p* < 0.05.

In addition to the photosynthetic pigment content itself, a very important indicator for assessing physiological changes in plants is the determination of the reciprocal ratio of chlorophyll *a* to chlorophyll *b* (Chl *a*/Chl *b*). An increase in the value of Chl *a*/Chl *b* indicates existing oxidative stress in the plant. A decrease in the value of this ratio, on the other hand, may be due to two independent factors. First, it may be due to an increase in Chl *b* content, which is beneficial for the plant, as it indicates an adequate level of light uptake for which this pigment is responsible.^137,138^

A second reason for the decrease in the Chl *a*/Chl *b* ratio is the decrease in the level of Chl *a* which, in turn, reflects extensive damage to the photosystems and photoinhibition. The second important indicator determined in phytotoxicity tests is the ratio of total Chl to carotenoids (Chl *a*+*b*/Car). A decrease in this value also indicates the existence of oxidative stress in the plant and, at the same time, indicates that the plant is trying to defend itself against the stress by increasing the content of carotenoids, which are effective scavengers of reactive oxygen species (ROS).^133^ On the basis of the obtained contents of photosynthetic pigments, the ratios of chlorophyll *a* to chlorophyll *b* and total chlorophyll to carotenoids were calculated. For both calculated values of the ratios, most of the tested compounds showed measurable changes with increasing FIL concentration in the soil, but these changes had different trends and were not linearly correlated with the applied concentration (Table S9). Changes in the Chl *a*/Chl *b* and Chl *a*+*b*/Car ratios of radishes grown in soil with high concentrations of FILs are indicative of oxidative stress, resulting in impaired growth and reduced yield.

#### 3.4.2 MDA and Hydrogen Peroxide (H_2_O_2_) Content in the Presence of FILs

MDA is a breakdown product of polyunsaturated fatty acids present in protein–lipid membranes. Excessive reactive oxygen species (ROS) that cause oxidative stress in plants can break down polyunsaturated fatty acids and lead to lipid peroxidation and, consequently, an increased MDA content in plant cells. MDA, in turn, can easily interact with functional groups found in proteins, lipoproteins, and DNA, thereby causing extensive cell damage.^133^ In this study, an increase in MDA content was observed in common radish grown on soils containing FILs. These changes were observed after application of the tested compounds at concentrations from 400 to 1000 mg·kg^–1^ soil DW. An increase in MDA content was not observed for [Men-Im-C_1_]­[TFSI] (**4c**) and [Men-Im-C_1_]­[PFSI] (**4d**). However, this is most likely due to the fact that these compounds caused such severe plant growth inhibition that it was not possible to obtain test material from plants growing on soil containing the tested compounds at concentrations of 400 mg·kg^–1^ soil DW and higher. It is also worth noting that the compounds containing the (1*R*,2*S*,5*R*)-(−)-menthol component had a much reduced effect on the increase in MDA content in common radish than their counterparts without such a component, which may indicate that they generate oxidative stress to a lesser extent (Figure 4A). The increase in MDA content in plants is also reported by Zhang et al.,^133^ Liu et al.^123^ and Cvjetko Bubalo et al.^139^ The observed increase in MDA content is explained by these authors as resulting from high concentrations of ILs leading to high oxidative stress in plants exceeding the capacity of plant defense mechanisms. Under such conditions, ROS cause the oxidation of fatty acids that constitute biological membranes, resulting in the formation of large amounts of MDA.

**Figure 4:**
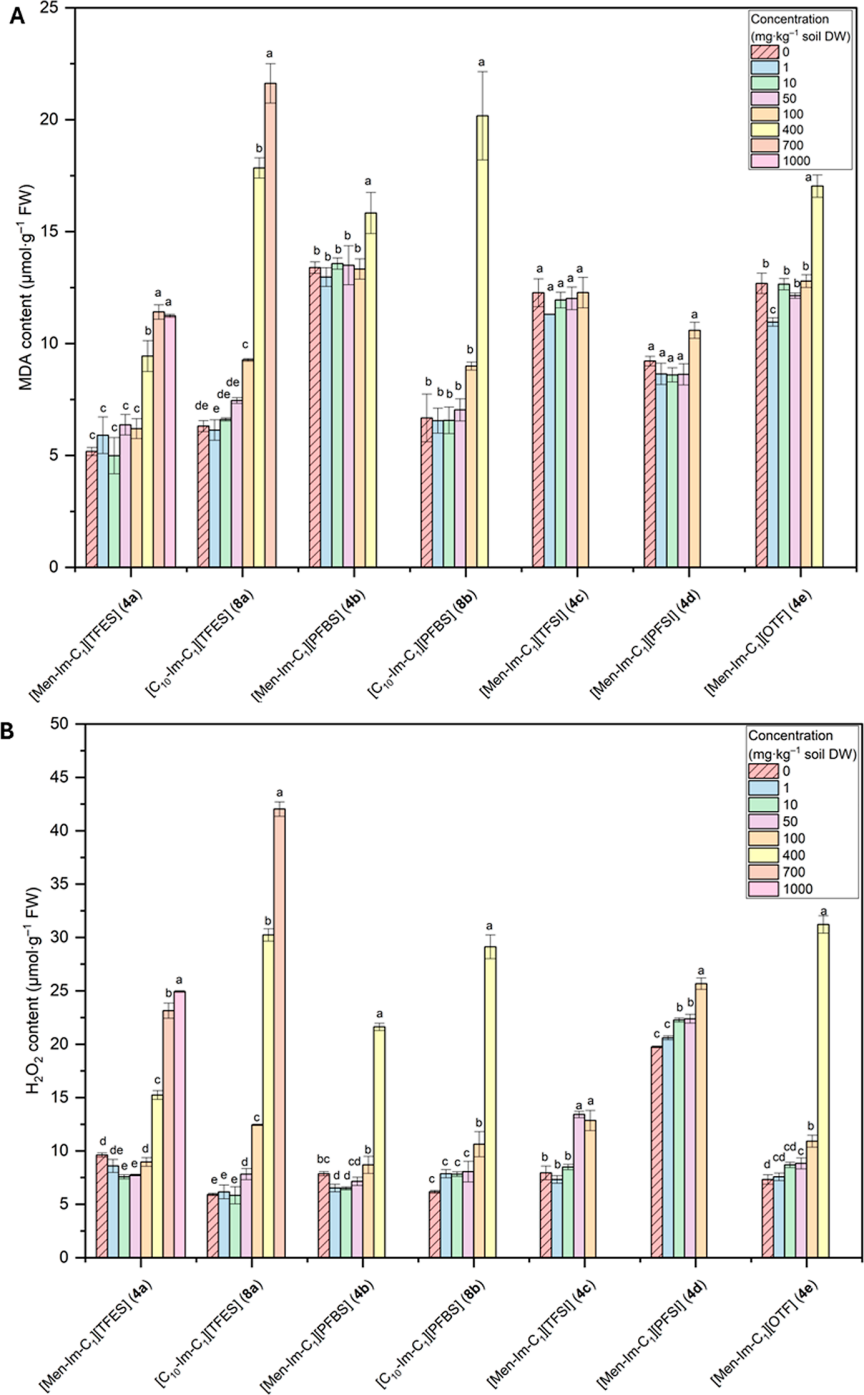
Oxidative stress indicators in common radish exposed to functionalized ionic liquids (FILs): (**A**) Malondialdehyde (MDA) content and (**B**) hydrogen peroxide (H_2_O_2_) content in radish leaves. ^a–e^Values denoted by the same letters do not differ statistically at *p* < 0.05.

Reactive oxygen species (ROS), *i.e.*, O_2_^–^, ^1^O_2_, HO•, ROO• or H_2_O_2_, are natural by—products of oxygen metabolism. Under normal conditions, ROS in plant cells are broken down by antioxidant enzymes. Under oxidative stress conditions, however, ROS are overproduced in plant cells. One of the most stable molecules categorized as ROS that can diffuse through biological membranes without difficulty is hydrogen peroxide (H_2_O_2_). H_2_O_2_ is constantly present in plants, as it is an important signaling molecule indicating oxidative stress—related disorders. Only an increase in H_2_O_2_ content in cells is dangerous for the plant, as it indicates increased superoxide dismutase (SOD) activity, which leads to overproduction of this ROS molecule as a result of detoxification of superoxide anion radical under oxidative stress conditions.^123,138,140^ The analysis of the results obtained for the determination of the effect of FILs on the hydrogen peroxide content of common radish indicates that all the compounds tested, especially when applied at higher concentrations, caused an increase in the content of this ROS in plants. The most significant effect on H_2_O_2_ content in plants was observed for [C_10_-Im-C_1_]­[TFES] (**8a**). When this FIL was applied at a concentration of 700 mg·kg^–1^ soil DW, the H_2_O_2_ content in cells increased more than 7-fold compared to that in control plants. A similarly large effect on H_2_O_2_ levels in common radish was also observed for [Men-Im-C_1_]­[PFSI] (**4d**), for which a significant increase in H_2_O_2_ content was detected even after application at a concentration of 10 mg·kg^–1^ soil DW. Similarly, as was the MDA content in plants grown on soil amended with FILs, the compounds containing the (1*R*,2*S*,5*R*)-(−)-menthol component in their molecules exerted less effect on the overproduction of H_2_O_2_ than their counterparts lacking this component (Figure 4B). An analogous increase in H_2_O_2_ content in plants grown on medium containing ILs was also reported by Zhang et al.^133^ for watercress, Cvjetko Bubalo et al.^139^ for barley, and Xu et al.^28^ for *V. faba*.

#### 3.4.3 Activity of Antioxidant Enzymes in the Presence of FILs

The inability of plants to escape from stress caused by a variety of both biotic and abiotic factors has led them to develop a complex system of protection against oxidative stress. As part of this system, they produce enzymes whose function is to defend plant cells against ROS. The action of antioxidant enzymes is specialized and linked together in a complex cycle of chemical reactions aimed at detoxifying excess ROS.^141–143^ The enzyme that constitutes the first line of defense and initiates the entire transformation cycle is superoxide dismutase (SOD). This enzyme catalyzes the dismutation reaction of superoxide anion radical to H_2_O_2_ and O_2_, thus protecting cell membranes from lipid peroxidation induced by this radical.^144,145^ Analysis of the results shows that the superoxide dismutase activity in common radish plants was dependent on the FILs used. [Men-Im-C_1_]­[PFBS] (**4b**) caused a decrease in SOD activity at lower concentrations, followed by an increase in the activity of this enzyme in common radish when higher concentrations of this compound were applied. [C_10_-Im-C_1_]­[PFBS] (**8b**) and [C_10_-Im-C_1_]­[TFES] (**4a**) caused a decrease in SOD activity starting at a concentration of 400 mg·kg^–1^ soil DW, which, when combined with the increase in H_2_O_2_ content in common radish, may suggest that the plant was unable to cope with the stress and that the antioxidant system had become damaged and inefficient.

[Men-Im-C_1_]­[TFES] (**4a**), on the other hand, caused a significant increase in SOD activity in radish when this FIL was applied at concentrations of 400–1000 mg·kg^–1^ soil DW. [Men-Im-C_1_]­[TFSI] (**4c**), [Men-Im-C_1_]­[OTF] (**4e**) and [Men-Im-C_1_]­[PFSI] (**4d**), meanwhile, did not cause statistically significant changes in SOD activity. However, this does not mean that these FILs did not cause oxidative stress in plants. On the contrary, these compounds had such a strong negative effect on plants that it was not possible to obtain test material after applying higher concentrations of the compounds tested (Table 3). Available literature data indicate that SOD activity in plants shows different directions of change depending on the applied compound or the plant species on which the compound acts.

**Table 3:** Antioxidant Enzyme Activities (SOD, CAT, POD) in Common Radish Residues on Soil Containing FILs in Yields (0–1000 mg·kg^–1^ Soil dry Weight, DW)

FIL	concentration (mg·kg^–1^ soil DW)							
	control	1	10	50	100	400	700	1000
	SOD (U·mg^–1^ protein)							
**4a**	10.911 ± 0.393^a^	11.266 ± 1.300^a^	11.329 ± 1.065^a^	11.928 ± 1.730^a^	11.907 ± 0.338^a^	25.905 ± 0.622^a^	22.151 ± 0.423^a^	22.118 ± 1.025^a^
**8a**	20.170 ± 0.868^a^	21.041 ± 0.306^a^	20.999 ± 0.194^a^	19.483 ± 0.473^a^	18.737 ± 1.056^a^	14.961 ± 0.765^a^	13.993 ± 0.116^a^	14.029 ± 0.233^a^
**4b**	9.998 ± 0.400^a^	9.362 ± 0.539^a^	7.028 ± 0.809^a^	7.138 ± 0.477^a^	9.549 ± 0.097^a^	9.976 ± 0.196^a^	12.341 ± 0.588^a^	–
**8b**	19.837 ± 0.237^a^	21.440 ± 0.420^a^	21.566 ± 0.332^a^	20.307 ± 0.300^a^	20.263 ± 0.747^a^	13.595 ± 0.945^a^	13.293 ± 0.445^a^	–
**4c**	13.611 ± 0.499^a^	13.547 ± 0.856^a^	13.288 ± 0.676^a^	12.584 ± 0.267^a^	12.691 ± 0.276^a^	–	–	–
**4d**	12.732 ± 0.278^a^	12.472 ± 0.492^a^	12.268 ± 0.296^a^	12.490 ± 0.495^a^	12.799 ± 0.255^a^	–	–	–
**4e**	11.059 ± 0.396^a^	10.752 ± 0.478^a^	10.124 ± 0.764^a^	11.645 ± 0.616^a^	11.189 ± 0.294^a^	9.943 ± 0.453^a^	–	–
	CAT (U·mg^–1^ protein·min^–1^)							
**4a**	0.0271 ± 0.0105^a^	0.0300 ± 0.0009^a^	0.0324 ± 0.0018^a^	0.0303 ± 0.0026^a^	0.0345 ± 0.0026^a^	0.0351 ± 0.0014^a^	0.0453 ± 0.0000^a^	0.0405 ± 0.0000^a^
**8a**	0.0388 ± 0.0023^a^	0.0417 ± 0.0000^a^	0.0483 ± 0.0024^a^	0.0485 ± 0.0040^a^	0.0583 ± 0.0021^a^	0.0920 ± 0.047^a^	0.1000 ± 0.0014^a^	0.0934 ± 0.0039^a^
**4b**	0.0186 ± 0.0012^a^	0.0169 ± 0.0014^a^	0.0162 ± 0.0007^a^	0.0234 ± 0.0028^a^	0.0462 ± 0.0063^a^	0.0636 ± 0.0025^a^	0.1087 ± 0.0000^a^	–
**8b**	0.0376 ± 0.0050^a^	0.0411 ± 0.0025^a^	0.0413 ± 0.0046^a^	0.0416 ± 0.000^a^	0.0417 ± 0.0045^a^	0.0494 ± 0.0030^a^	0.0589 ± 0.0025^a^	–
**4c**	0.0245 ± 0.0029^a^	0.0238 ± 0.0018^a^	0.0282 ± 0.0016^a^	0.0287 ± 0.0016^a^	0.0296 ± 0.0014^a^	–	–	–
**4d**	0.0220 ± 0.0015^a^	0.0253 ± 0.0030^a^	0.0267 ± 0.0000^a^	0.0281 ± 0.0028^a^	0.0298 ± 0.0000^a^	–	–	–
**4e**	0.0227 ± 0.0015^a^	0.0193 ± 0.0010^a^	0.0202 ± 0.0016^a^	0.0207 ± 0.0015^a^	0.0230 ± 0.0029^a^	0.0277 ± 0.0015^a^	–	–
	POD (U·mg^–1^ protein·min^–1^)							
**4a**	1.068 ± 0.058^a^	0.938 ± 0.042^a^	0.947 ± 0.020^a^	1.071 ± 0.036^a^	1.224 ± 0.093^a^	2.655 ± 0.069^a^	3.667 ± 0.123^a^	2.687 ± 0.116^a^
**8a**	1.381 ± 0.037^a^	1.493 ± 0.014^a^	1.602 ± 0.036^a^	1.830 ± 0.032^a^	2.398 ± 0.026^a^	4.386 ± 0.028^a^	4.447 ± 0.018^a^	4.350 ± 0.027^a^
**4b**	0.899 ± 0.042^a^	0.784 ± 0.012^a^	1.066 ± 0.043^a^	1.261 ± 0.034^a^	1.456 ± 0.067^a^	4.327 ± 0.032^a^	9.260 ± 0.424^a^	–
**8b**	0.749 ± 0.009^a^	0.763 ± 0.023^a^	0.761 ± 0.009^a^	0.814 ± 0.046^a^	1.159 ± 0.034^a^	3.308 ± 0.090^a^	5.687 ± 0.162^a^	–
**4c**	4.454 ± 0.039^a^	4.342 ± 0.006^a^	4.447 ± 0.015^a^	4.756 ± 0.554^a^	10.868 ± 0.064^a^	–	–	–
**4d**	0.692 ± 0.018^a^	0.483 ± 0.000^a^	0.510 ± 0.018^a^	0.898 ± 0.019^a^	1.256 ± 0.051^a^	–	–	–
**4e**	0.608 ± 0.032^a^	0.396 ± 0.018^a^	0.598 ± 0.034^a^	0.522 ± 0.005^a^	1.411 ± 0.038^a^	2.853 ± 0.015^a^	–	–

a Values denoted by the same letters in the columns do not differ statistically at *p* < 0.05.

Cvjetko Bubalo et al.,^139^ Fan et al.,^146^ and Zhu et al.^143^ observed no clear correlation between SOD activity and the concentration of ILs applied, as SOD activity initially increased and then decreased with higher concentrations of ILs applied. Liu et al.,^147^ on the other hand, observed a decrease in SOD activity in rice roots in contact with ILs, while in rice seedlings, changes in SOD activity were not linearly related to the concentration of ILs tested. Different results were obtained by Liu et al.,^123^ who, investigating the effect of [C_8_-Im-C_1_]­[PF_6_] on wheat, observed a progressive increase in SOD activity with increasing IL concentration.

Excess H_2_O_2_ formed in plant cells is removed by two groups of enzymes: catalase (CAT) and POD. Unlike the other enzymes, CAT does not require a reductant. This enzyme can exhibit dual catalytic functions. At high concentrations of H_2_O_2_, it mainly exhibits catalase activity, *i.e.*, it breaks down H_2_O_2_ into water and oxygen. In contrast, at low H_2_O_2_ concentrations, CAT exhibits POD activity, oxidizing, among others, methanol, ethanol, formate and quinones.^148,149^

Our results indicate that FILs caused an increase in CAT activity in radish especially after the application of higher concentrations of the tested compounds. The greatest increase in CAT activity was caused by the presence of [Men-Im-C_1_]­[TFES] (**4a**), [C_10_-Im-C_1_]­[TFES] (**8a**) [Men-Im-C_1_]­[PFBS] (**4b**) and [C_10_-Im-C_1_]­[PFBS] (**8b**) in the soil on which the radish was grown (Table 3). The increase in CAT activity unquestionably demonstrates the occurrence of oxidative stress. Moreover, this also indicates that the antioxidant system of the plants is functioning properly by increasing CAT activity to remove excess H_2_O_2_. Cvjetko Bubalo et al.^139^ in their study also observed an increase in CAT activity in barley seedlings in contact with ILs. However, the direction of changes in CAT activity in plants exposed to ILs is not always consistent. Liu et al.^123^ found a decrease in CAT activity in wheat seedlings under the influence of [C_8_-Im-C_1_]­[PF_6_].

Zhu et al.,^143^ on the other hand, observed an initial increase followed by a decrease in CAT activity in oilseed rape plants grown on medium with increasing concentrations of ILs. The second group of enzymes, important in the removal of H_2_O_2_, are PODs. In contrast to the previously discussed catalases, PODs require the presence of an electron donor, such as pyrogallol, benzidine, guaiacol or ascorbic acid, to catalyze the H_2_O_2_ degradation reaction.^144,150,151^ The results obtained in the present study indicate that all the FILs tested caused a significant increase in POD activity, which was positively correlated with increasing concentrations of the tested compounds in the soil on which radish was grown (Table 3). Similar increases in POD activity in plants exposed to ILs were also observed by Liu et al.,^123^ Xu et al.^152^ and our group.^25^ Cvjetko Bubalo et al.,^139^ investigating the effect of ILs with different anions and varying alkyl chain lengths, reported that POD activity was dependent on the compound used. An increase in the activity of antioxidant enzymes, including POD, combined with a decrease in photosynthetic pigments, may indicate premature aging of plants, which in turn may lead to earlier onset of plant senescence and death.

### 3.5 Linking Molecular Features with Phytotoxic Outcomes: A Multivariate Perspective

Although the overall phytotoxic response is consistent with membrane-associated, surfactant-like mechanisms, the multivariate analysis (Figures 5, S49) demonstrates that both anion identity and cation architecture systematically modulate toxicity. These effects cannot be rationalized solely by classical surfactant descriptors based on global amphiphilicity, such as alkyl chain length or surface activity, but instead arise from distinct, structure-dependent features deliberately encoded in the cation–anion architecture at the ionic-liquid design stage.^21^ The research questions Q1 and Q2 (Scheme 2) were formulated to elucidate the relationship between the molecular structure of FILs and their phytotoxic effects, based on a set of precisely designed ionic compounds. This study adopted a comparative and multivariate approach to identify the correlation between structural parameters, including lipophilicity (log *K*_OW_), water solubility, surface tension, molecular volume and bioaccumulation potential (log BCF), and the experimentally determined phytotoxicity (EC_50_; inhibition for fresh weight) toward common radish. Particular attention was devoted to identifying and quantifying the distinct roles of the anion and cation in modulating these properties.

**Figure 5:**
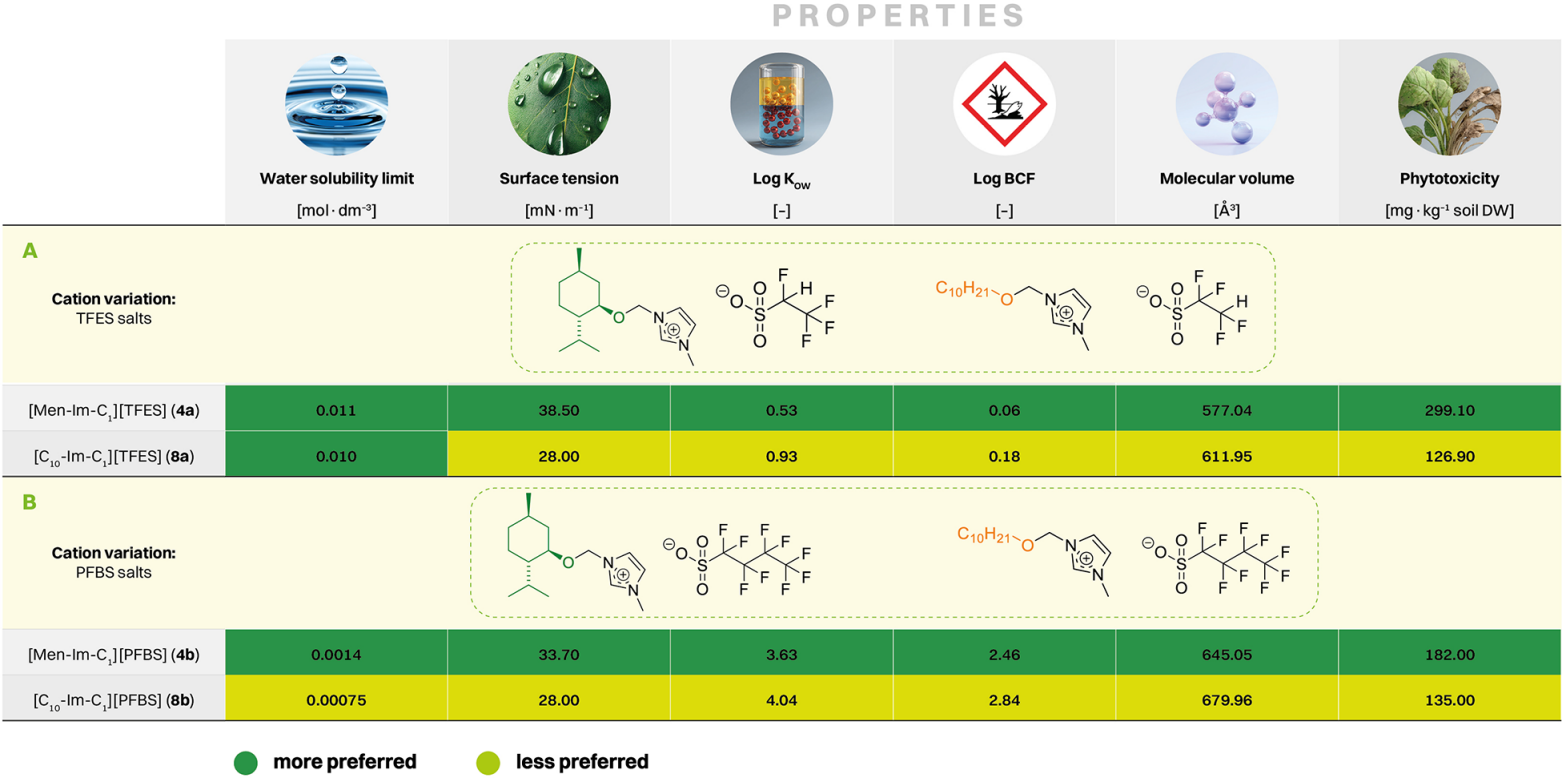
Heatmap visualization of structure–property–phytotoxicity relationships for selected FILs—cation-dependent comparisons. (**A**) Comparison of [Men-Im-C_1_]^+^ and [C_10_-Im-C_1_]^+^ cations for [TFES]^−^ salts. (**B**) Comparison of [Men-Im-C_1_]^+^ and [C_10_-Im-C_1_]^+^ cations for [PFBS]^−^ salts. Color gradients (green, more-preferred; yellow, less-preferred) depict relative trends in water solubility, surface tension, predicted log *K*_OW_ and log BCF, molecular volume, and phytotoxicity (EC_50_; inhibition for fresh weight).

Notably, the current literature contains relatively few studies that systematically examine how variations in anion structure influence phytotoxicity, particularly within coherent series of related compounds. Most available reports compare anions that differ substantially in both chemical composition and topology, which limits the ability to draw precise structure–toxicity relationships. In contrast, the present study evaluates structurally consistent fluorinated anions, enabling a more meaningful structure–toxicity comparison. Furthermore, no prior systematic investigation has directly compared a natural cyclic component with its linear synthetic counterpart within the same cationic framework in terms of their combined influence on structural and physicochemical properties. In this context, particular focus was placed on differences between FILs incorporating a bulky, chiral cycloalkoxymethyl moiety derived from (1*R*,2*S*,5*R*)-(−)-menthol and those bearing a linear, 1-decanol-derived alkoxymethyl substituent. This offers a direct assessment of the role of molecular geometry and substitution pattern.

#### 3.5.1 Anion-dependent Trends (Q1)

For the (1*R*,2*S*,5*R*)-(−)-menthol-based FILs (**4a**–**4e**), the phytotoxic effects varied significantly depending on the fluorinated anion. [Men-Im-C_1_]­[TFSI] (**4c**) and [Men-Im-C_1_]­[PFSI] (**4d**), both sulfonylimides, were among the most phytotoxic compounds (EC_50_ = 74.7 and 107.3 mg·kg^–1^ soil DW, respectively) compared to those with sulfonate anions: [Men-Im-C_1_]­[TFES] (**4a**), [Men-Im-C_1_]­[PFBS] (**4b**) and [Men-Im-C_1_]­[OTF] (**4e**) (Scheme 4). [Men-Im-C_1_]­[TFES] (**4a**) exhibited the lowest toxicity (EC_50_ = 299.1 mg·kg^–1^ soil DW). These trends correlate with predicted lipophilicity (log *K*_OW_: 2.49 for **4c**, 4.43 for **4d** *vs* 0.527 for **4a**) and bioaccumulation potential (log BCF: 1.35 and 3.23 *vs* 0.064), as shown in Figure 1C,D. Although [Men-Im-C_1_]­[PFBS] (**4b**) displayed high log *K*_OW_ (3.63) and log BCF (2.46) values, its EC_50_ value (182 mg·kg^–1^ soil DW) was lower than that of [Men-Im-C_1_]­[TFES] (**4a**), indicating that increased lipophilicity and bioaccumulation potential can correspond to greater toxicity in this pairwise comparison. However, the markedly higher EC_50_ of **4b** compared to [Men-Im-C_1_]­[TFSI] (**4c**, 74.7 mg·kg^–1^ soil DW) and [Men-Im-C_1_]­[PFSI] (**4d**, 107.3 mg·kg^–1^ soil DW) demonstrates that this relationship does not hold universally across the series.

Another noteworthy observation concerns the surface tension at the saturation concentration. In the menthol-based FILs (series A), the highest values were recorded for [Men-Im-C_1_]­[TFSI] (**4c**, 50.1 mN·m^–1^) and [Men-Im-C_1_]­[PFSI] (**4d**, 55.0 mN·m^–1^), which are also among the most phytotoxic in the series (EC_50_ = 74.7 and 107.3 mg·kg^–1^ soil DW, respectively). This demonstrates that, in series A, higher surface tension does not correspond to reduced phytotoxicity. By contrast, in series B and C (cation-dependent trends), an opposite trend is evident—ionic compounds with higher surface tension generally display lower phytotoxic effects—indicating that the influence of interfacial behavior on bioavailability is strongly series-dependent. Interestingly, compounds **4c** and **4d** exhibited relatively low water solubility limits and, as stressed above, elevated surface tensions (see Figure 1A), which may contribute to their prolonged presence in soil. Together with their higher log BCF values, this suggests that both salts are more likely to persist in the soil, thereby increasing exposure at the root level and amplifying their phytotoxic effects. By contrast, the least phytotoxic FIL, [Men-Im-C_1_]­[TFES] (**4a**), exhibits greater water solubility (0.011 mol·dm^–3^) and lower surface tension (38.5 mN·m^–1^), potentially limiting its bioavailability in soil and reducing contact with plant tissues. Overall, these findings provide partial support for Q1: although imide-based anions generally correlate with greater phytotoxicity than sulfonates, no single physicochemical parameter can predict toxicity on its own. Instead, in series A, low water solubility, high log *K*_OW_ and elevated log BCF appear to act synergistically to enhance the phytotoxic response as exemplified by [Men-Im-C_1_]­[TFSI] (**4c**) and [Men-Im-C_1_]­[PFSI] (**4d**), the most toxic menthol-based FILs, characterized by high lipophilicity and bioaccumulation potential. In contrast, [Men-Im-C_1_]­[TFES] (**4a**)—the least toxic compound—combines low log *K*_OW_ and log BCF with high water solubility and low surface tension. These observations highlight that the anion modulates multiple interdependent properties, which collectively determine the biological outcome. [Men-Im-C_1_]­[PFBS] (**4b**) illustrates that this relationship is not universal: despite high log *K*_OW_ and log BCF values, its phytotoxicity is substantially lower than that of **4c** and **4d**. This indicates that lipophilicity and bioaccumulation potential alone are insufficient predictors, and that other molecular features—such as hydration energy, interfacial tension (γ), and cohesive interactions—likely contribute. The absence of a clear surface tension–toxicity correlation in series A contrasts with series B and C, where higher surface tension generally corresponds to reduced phytotoxicity. These multidimensional relationships are visualized in a heatmap in the Supporting Information (Figure S49), which integrates all key physicochemical and biological parameters of the [Men-Im-C_1_]^+^ salts. This heatmap confirms that no single parameter, such as solubility, surface tension or lipophilicity, can predict phytotoxicity outcomes independently, emphasizing the importance of cumulative and context-specific interactions. Furthermore, the literature on IL phytotoxicity rarely examines structurally related sulfonates and sulfonylimides in such a systematic manner. Most studies focus on short-chain alkyl analogues or structurally divergent anions, with limited attention paid to fine structural effects or comparative assessments across homologous series. For instance, Pawłowska et al.^21^ observed that although lipophilicity frequently correlates with toxicity, soil conditions and ion interactions can significantly modify this relationship. They also noted that log *K*_OW_ > 3 may indicate a risk of bioaccumulation and environmental toxicity, which aligns with the properties of **4b** and **4d**. However, these descriptors require contextual interpretation due to experimental variability and matrix complexity (e.g., pH, organic matter content). These structure–activity relationships emphasize the importance of precise anion design for mitigating environmental risks. Although trends in lipophilicity and bioaccumulation support Q1, the interplay between anion structure, aqueous compatibility, and interfacial behavior is complex, and strongly series-dependent.

#### 3.5.2 Cation-dependent Trends (Q2)

To evaluate Q2, we compared pairs of FILs with identical anionsbut differing cationic structures: [Men-Im-C_1_]^+^, which contains a bulky, chiral cycloalkoxymethyl group derived from (1*R*,2*S*,5*R*)-(−)-menthol; and [C_10_-Im-C_1_]^+^, which bears a linear 1-decanol-based alkoxymethyl chain. These comparisons (**4a** vs **8a** and **4b** vs **8b**, both with [TFES]^−^ and [PFBS]^−^, respectively) allow for a direct assessment of the role of cation topology and geometry in modulating phytotoxicity and related physicochemical features (see Scheme 4).

Despite having nearly identical water solubility limits (**8a**, 0.010 and **4a**, 0.011 mol·dm^–3^ respectively), [C_10_-Im-C_1_]­[TFES] (**8a**) was significantly more phytotoxic (EC_50_ = 126.9 mg·kg^–1^ soil DW) than its (1*R*,2*S*,5*R*)-(−)-menthoxymethyl-based analogue [Men-Im-C_1_]­[TFES] (**4a**, EC_50_ = 299.1 mg·kg^–1^ soil DW). A similar trend was observed for the [PFBS]^−^ pair: [C_10_-Im-C_1_]­[PFBS] (**8b**) showed a lower EC_50_ of 135.0 mg·kg^–1^ soil DW compared to [Men-Im-C_1_]­[PFBS] (**4b**), although the magnitude of the difference was smaller than for the [TFES]^−^ pair. In this case, **4b** is almost twice as soluble as **8b** (0.0014 vs 7.5·10^–4^ mol·dm^–3^), yet it is the less phytotoxic, indicating that solubility alone does not dictate phytotoxicity for these cationic variants. These observations suggest that the phytotoxic effects cannot be fully accounted for by aqueous solubility alone. Instead, the enhanced toxicity of the **8a** and **8b** ionic compounds is more likely to be linked to their higher lipophilicity and bioconcentration potential, as evidenced by their predicted log *K*_OW_ and log BCF values (Figure 1C,D). Structurally, the [C_10_-Im-C_1_]^+^ cation has a larger molecular volume (611.95 Å^3^ for **8a** and 679.96 Å^3^ for **8b** compared to 577.04 Å^3^ for **4a** and 645.05 Å^3^ for **4b**) and is more asymmetric (an asymmetry index of 3.18 for **8a** *vs* 2.95 for **4a** and 2.17 for **8b** *vs* 2.01 for **4b**). These features likely increase the hydrophobic surface area, reduce hydration affinity and promote membrane interaction as well as passive diffusion into plant tissues. Taken together, these results strongly support the hypothesis that FILs with linear 1-decanol-based cations exhibit higher phytotoxicity than their menthol-derived analogues, irrespective of the anion studied. This is due to a combination of higher lipophilicity, greater molecular volume and asymmetry, and increased accumulation potential. The consistent enhancement of toxicity in both [TFES]^−^ and [PFBS]^−^ contexts suggests that cation geometry plays a dominant and broadly applicable role in modulating the biological impact of this system. These interrelated properties are visualized collectively in the heatmaps shown in Figure 5A–B, which compare cation-dependent changes for the [TFES]^−^ and [PFBS]^−^ salts. These heatmaps clearly demonstrate that FILs bearing the linear [C_10_-Im-C_1_]^+^ cation consistently exhibit higher lipophilicity (log *K*_OW_), greater bioaccumulation potential (log BCF), a larger molecular volume and lower EC_50_ values than their [Men-Im-C_1_]^+^ analogues. This graphical representation further highlights the synergistic role of structural and physicochemical factors in modulating phytotoxicity and emphasizes the significant impact of cation geometry on environmental behavior. Furthermore, these findings align with previous reports on imidazolium ILs, which indicate that toxicity and membrane disruption increase with longer and more linear alkyl chains.^21^ Notably, this comparison includes natural chiral building blocks, thereby extending the relevance of this conclusion to green IL design strategies.

The multivariate analysis links structure to biological response: phytotoxicity reflects the combined effects of aqueous compatibility, lipophilicity (log *K*_OW_) and accumulation potential (log BCF), rather than any single descriptor. The interplay between anion hydrophobicity and cation geometry is consistent with proxies of environmental fate and plant uptake and with the observed oxidative-stress markers in *R. sativus*. Within our series, replacing the linear C10 chain with a (−)-menthol moiety consistently reduced effects, indicating that natural, cyclic substituents can be used to attenuate hazard in FIL design. These structure–activity rules provide practical criteria for prioritizing safer FILs—favoring lower lipophilicity/BCF and higher aqueous compatibility—within the scope of the present data set.

## Supplementary Material



## Data Availability

The dataset related to this article is available at the RePOD repository: https://doi.org/10.18150/OIXK2Y.

## Notes

Materials and purification; experimental procedures; characterization (^1^H, ^13^C, ^19^F NMR, FTIR, HRMS, elemental analysis, specific rotation); solubility, surface tension, refractive index; thermal analysis (TGA/DSC); molecular descriptors and QSAR (log *K*_OW_, log BCF); supplementary *R. sativus* bioassay tables (emergence, shoot/root inhibition, fresh/dry mass, chlorophylls/pigment ratios); complete spectra sets and stacked comparisons; structures and molecular volumes; and a structure–property–toxicity heatmap (PDF)
