# Supplementary material for: Designing Safer Functionalized Ionic Liquids: Monoterpene-Derived Compounds for Greener Agrochemical Development
Source: J Agric Food Chem. 2026 Feb 2;74(8):7152–82. doi: 10.1021/acs.jafc.5c12979 (PMC12964537; doi:10.1021/acs.jafc.5c12979)
Supplement: Supplementary file 1 [file jf5c12979_si_001.pdf]

Supporting Information

Designing safer functionalised ionic liquids: Monoterpene-derived compounds for greener agrochemical development

Przemysław Pietrusiak, Barbara Pawłowska, Magdalena Bendová, Robert Biczak, Joanna Feder-Kubis*

**Corresponding author: Technische Universität Dresden, Faculty of Chemistry, Bergstrasse 66, 01062, Dresden, Germany; Wrocław University of Science and Technology, Faculty of Chemistry, Wybrzeże Wyspiańskiego 27, 50-370 Wrocław, Poland; Email: joanna.feder-kubis@pwr.edu.pl; joanna_maria.feder-kubis@tu-dresden.de*

Number of pages: 102

Number of Figures: 49

Number of tables: 9

Table of Contents

1. Materials and Purification Methods……………………………………………………………S4
2. Methods of Characterization of Obtained FILs……………………………………………S4–S6
2.1. Spectral and Elemental Analysis…………………………………………………………S4
2.2. Specific Rotation……………………………………………………………………………S4
2.3. Solubility…………………………………………………………………………………S4–S5
2.4. Surface Tension……………………………………………………………………………S5
2.5. Molecular Properties………………………………………………………………………S5
2.6. QSAR Prediction of Log K_{OW} and Log BCF…………………………………………S5–S6
2.7. Thermal Analysis……………………………………………………………………………S6
2.8. Refractive Index……………………………………………………………………………S6
3. Results……………………………………………………………………………………S6–S12
3.1. NMR Characterisation of 1-[(1*R*,2*S*,5*R*)-(–)-Menthoxymethyl]-3-methylimidazolium [Men-Im-C_1][X] Salts (**3**, **4a**–**4e**) and 1-Decyloxymethyl-3-methylimidazolium [C_{10}-Im-C_1][X] (**7**, **8a**, **8b**) Salts……S6–S10
3.2. The Impact of Anion Variation on NMR Chemical Shifts…………………………S10–S11
3.3. Refractive Index of FILs Obtained (**4c**–**4d**, **8a**–**8b**)…………………………………S12
3.4. Supplementary Tables……………………………………………………………S13–S21
Table S1. Physicochemical Properties of 1-[(1*R*,2*S*,5*R*)-(–)-Menthoxymethyl]-3-methylimidazolium [Men-Im-C_1][Cl] (**3**) and 1-Decyloxymethyl-3-methylimidazolium [C_{10}-Im-C_1][Cl] (**7**) Chlorides……S13
Table S2. The Comparison of ^{1}H Chemical Shifts for Selected Protons of 1-[(1*R*,2*S*,5*R*)-(–)-Menthoxymethyl]-3-methylimidazolium Cation Depending on Anions……S14
Table S3. The Comparison of ^{1}H Chemical Shifts for Selected Protons of 1-Decyloxymethyl-3-methylimidazolium Cation Depending on Anion……S15
Table S4. Effect of FILs on Common Radish Seed Emergence……S16
Table S5. The Inhibition Rate for Shoot Length and Root Length of Common Radish After Exposure to FILs..S17
Table S6. Fresh Weight Yield of Common Radish Plants Grown in Soil with the Addition of FILs……S18
Table S7. Effect of FILs on the Dry Weight in Common Radish Plants……S19
Table S8. Chlorophyll a Content (Chl a) and Chlorophyll b Content (Chl b) in Common Radish Leaves Growing on Soil Supplemented with FILs……S20
Table S9. Changes in Chl a/Chl b and Chl a+b/Car Values in Leaves of Common Radish Growing in Soil with the Addition of FILs……S21
3.5. Supplementary Figures……S22–S100
Figures S1–S3. TGA Thermograms of FILs……S22–S23
Figures S4–S10. DSC Thermograms of FILs……S23–S26
Figure S11. Refractive Indexes Chart of FILs in Liquid State Depending on Temperature……S27
Figures S12–S20. FTIR Spectra of 1-[(1*R*,2*S*,5*R*)-(–)-Menthoxymethyl]-3-methylimidazolium [Men-Im-C_1][X] salts (**3**, **4a**–**4e**) and 1-Decyloxymethyl-3-methylimidazolium [C_{10}-Im-C_1][X] (**7**, **8a**, **8b**) Salts……S28–S36
Figures S21–S45. NMR Spectra of 1-[(1*R*,2*S*,5*R*)-(–)-Menthoxymethyl]-3-methylimidazolium [Men-Im-C_1][X] salts (**3**, **4a**–**4e**) and 1-Decyloxymethyl-3-methylimidazolium [C_{10}-Im-C_1][X] (**7**, **8a**, **8b**) Salts in $CDCl_3$..S37–S96
Figures S46–S47. Cation Moiety-dependent Shifts in ^{1}H NMR Spectra of Studied of 1-[(1*R*,2*S*,5*R*)-(–)-Menthoxymethyl]-3-methylimidazolium [Men-Im-C_1][X] Salts (**3**, **4a**–**4e**) and 1-Decyloxymethyl-3-methylimidazolium [C_{10}-Im-C_1][X] (**7**, **8a**, **8b**) Salts……S97–S98
Figure S48. Structure and Molecular Volume in Å^3 of Cationic and Anionic Constituents of FILs……S99
Figure S49. Heatmap Visualisation of Structure–Property–Toxicity Relationships for [Men-Im-C_1]$^+$ Salts (Anion-Dependent Comparisons)……S100

References……………………………………………………………………..……..…………….S101–S101

1. Materials and Purification Methods

(1*R*,2*S*,5*R*)-(–)-Menthol (99%), 1-decanol (98%), 1-methylimidazole (≥99%), sodium trifluoromethanesulfonate (99.9%), paraformaldehyde (powder, 95%), hydrochloric acid (35-38%), sulphuric acid (≥96%) were purchased from Merck. Lithium bis(trifluoromethylsulfonyl)imide (99%), potassium perfluorobutanesulfonate (>98%), potassium 1,1,2,2-tetrafluoroethanesulfonate (>98%), lithium bis(pentafluoroethylsulfonyl)imide (>99%) were obtained by IoLiTec. Drying agents – sodium sulphate (anhydrous, pure) and phosphorus pentoxide (powder, anhydrous, ≥98%) – were purchased from Alchem (Gliwice, Poland). Solvents were sourced by Alchem, Merck and Fluka. All the reagents were purified, dried and stored on molecular sieves according to standard procedures. The heterocyclic amine, 1-methylimidazole, was purified by vacuum distillation each time before use (boiling point = 44–45 °C; pressure 1−3 mbar). The synthesised quaternary imidazolium salts were stored in Schlenk flasks and placed in a desiccator to reduce exposure to atmospheric moisture. They were also dried between experiments to ensure consistent compound quality.

2. Methods of Characterization of Obtained FLs

2.1. Spectral and Elemental Analysis

The chemical structures of FILs were identified by ^{1}H NMR, ^{13}C NMR and ^{19}F NMR spectroscopy. The ^{1}H NMR (400 MHz), ^{13}C NMR (100 MHz) and ^{19}F NMR (100 MHz) spectra were recorded on a Bruker DRX instrument using tetramethylsilane as a standard in $CDCl_3$. The spectra presented in the ESI are consistent with the proposed structure. Chemical shifts are given in ppm using tetramethylsilane as a standard. Residual $CDCl_3$ signals were used as the reference. Coupling constants *J* are given in Hz. Abbreviations used in the NMR spectrum analysis as follows: *s* = singlet, *d* = doublet, *t* = triplet, *q* = quartet, *hept* = heptet, *sept* = septet. Fourier transform infrared spectroscopy (FTIR) was performed on a Nicolet iS50 spectrometer (Thermo Fisher Scientific, Waltham, MA, USA) with a resolution of 4 cm^{-1} at wavenumbers in the range of 500–3500 cm^{-1}. The measured values are given in wavenumbers (cm^{-1}). Abbreviations used in FTIR description are as follows: s – symmetric, as – asymmetric, ν – stretching, δ – bending, ρ – rocking and τ – twisting. High-resolution mass spectra (HRMS) were recorded on an LCT Premier XE Waters spectrometer (TOF MS ES+/ TOF MS ES–). A VARIO EL-III was used for elemental analysis.

2.2. Specific Rotation

The specific rotation was measured using an Autopol I polarimeter from Rudolph Research Analytical. For the measurements, solutions of salts based on optically active (1*R*,2*S*,5*R*)-(–)-menthol were prepared at a concentration of 0.01 $g \cdot mL^{-1}$ (± 0.1 mg) using dichloromethane as the solvent at 25 °C. Measurements were repeated four times.

2.3. Solubility

The solubility test of quaternary imidazolium chlorides precursors, organic salts used for metathesis and synthesised FILs (**4a**–**4e, 8a** and **8b**) was carried out according to the procedure decribe in *Vogel's Textbook of Practical Organic Chemistry*.[1] All of those saltswere tested at ambient pressure using

common solvents of various polarity. A 0.10 g (± 0.01 g) sample of each salt was added to 1.00 mL of the chosen solvent and then thermostated in a water bath at 25 °C. For poorly soluble or insoluble samples, the analysis was repeated at 50 °C.

The solubility of the characterised salts falls into three categories: (*i*) **completely soluble** – 0.10 g (± 0.01 g) of the salt tested dissolves in 1.00 mL (± 0.10 mL) of the appropriate solvent; (*ii*) **limitedly soluble** – 0.10 g (± 0.01 g) of salt dissolves in 3.00 mL (± 0.10 mL) of a given solvent; (*iii*) **poorly soluble/insoluble** – 0.10 g (± 0.01 g) of salt does not dissolve in 3.00 mL (± 0.10 mL) of the solvent. The solvents used for this test were tested in order of increasing polarity: *n*-hexane, toluene, tetrahydrofuran (THF), ethyl acetate (EtOAc), chloroform ($CHCl_3$), dichloromethane (DCM), acetone (Ace), dimethylformamide (DMF), dimethyl sulfoxide (DMSO), acetonitrile (AcN), *iso*-propanol (*i*-PrOH), *n*-propanol (*n*-PrOH), ethanol (EtOH), methanol (MeOH), and water (H_2O).

2.4. Surface Tension

Surface tension (γ, in $mN \cdot m^{-1}$) was measured with a Krüss Digital Tensiometer, model K100 (Germany) using the Du Noüy ring detachment method. The temperature of the measuring cell was maintained at 22 °C with an accuracy of ± 0.1 °C controlled by a thermostat. The tensiometer was calibrated using double-distilled water prior to each measurement. The measurments were repeated at least three times. Due to the hydrophobic character of FILs, they were dissolved in water using a sonication process until a clear solution was obtained. Surface tension was measured at concentrations corresponding to the solubility limit in distilled water at 22 °C. Above this concentration, the solutions became turbid.

2.5. Molecular Properties

Molecular volume (V_m) was calculated with Multiwfn software on optimised structures of cations and anions using Avogadro software.[2,3] The structures of the cations and anions, as well as their molecular volumes, are presented in Figure S48. Molecular volume of each FIL was calculated as a sum of molecular volume of cation and anion according to Eq. 1:[4]

$$V_m = V_m(cation) + V_m(anion) \qquad \text{Eq. 1}$$

Assymetry index (AI) of each FIL was calculated as a ration of molecular volume of cation to molecular volume of anion (Eq. 2):

$$AI = \frac{V_m(cation)}{V_m(anion)} \qquad \text{Eq. 2}$$

2.6. QSAR Prediction of Log K_{OW} and Log BCF

The QSAR Toolbox software was used to predict the *n*-octanol-water partition coefficient (log Kow) and the bioconcentration factor (log BCF) for all FILs obtained. These estimations incorporated a read-across approach and QSAR models based on structural fragments available in the software database. The chemical structures of FILs were entered into the software, and their dissociation into cationic and anionic forms was analysed. The program then identified structural similarities to the database compounds and applied the relevant QSAR models to predict the log Kow and log BCF values. Where

direct prediction was unavailable, interpolation based on structural analogues was employed to improve the accuracy of the estimates.

2.7. Thermal Analysis

Thermogravimetric analysis (TGA) was performed using a Mettler Toledo TGA Q50 analyser. Samples weighing between 5 and 10 mg were heated from 0 °C to 500 °C at a heating rate of 5 °C·min^{-1}, with an isothermal hold at 85 °C. The isothermal stage was included to remove residual water and volatile impurities in the samples. From the thermal decomposition curve of each FIL, the onset temperature of decomposition corresponding to a 5% mass loss ($T_{5\%onset}$) and 50% mass loss ($T_{50\%onset}$) was determined.

Differential scanning calorimetry (DSC) was used to analyse the phase transitions of FILs (STARe system, Mettler-Toledo), cooled using a Huber TC100 immersion cooler. The procedure consisted of: (*i*) first heating cycle from 25 °C to 150 °C; (*ii*) first cooling cycle from 150 °C to −80 °C; (*iii*) second heating cycle from −80 °C to 150 °C; (*iv*) second cooling cycle from 150 °C to −80 °C, with isothermal holds at −80 °C and 150 °C between cycles. The heating and cooling rate was 10 °C·min^{-1}. The thermal events identified in the DSC thermograms are marked as follows: T_g – glass transition, T_m – melting point, T_c – crystallisation, T_{cc} – cold crystallisation, T_{pr} – pre-crystallization relaxation, T_{LC1} and T_{LC2} for liquid crystal phase transitions.

Melting points were additionally determined using an electrothermal digital melting point apparatus (model JA 9100). Measurement uncertainty was based on the instrument specifications: temperature resolution of ± 0.1 °C; accuracy of ± 1%; and ramp rate of 1.0 °C·min^{-1}.

2.8. Refractive Index

Refractive index measurements for liquid FILs were carried out using an ATAGO RX-5000α automatic digital refractometer. Measurements were performed at 25 °C (up to 60 °C) at 1000 mbar, with an uncertainty of ± 0.00004 n_D units and the temperature probe accuracy of ± 0.1 °C. Prior to each measurement, the refractive index of pure distilled water was measured and compared with the value reported in the literature.[5,6]

3. Results

3.1. NMR Characterisation of 1-[(1*R*,2*S*,5*R*)-(–)-Menthoxymethyl]-3-methylimidazolium [Men-Im-C$_1$][X] Salts (3, 4a–4e) and 1-Decyloxymethyl-3-methylimidazolium [C$_{10}$-Im-C$_1$][X] (7, 8a, 8b) Salts

1-[(1R,2S,5R)-(–)-Menthoxymethyl]-3-methylimidazolium chloride [Men-Im-C$_1$][Cl] (**3**).

^{1}H NMR (400 MHz, CDCl$_3$, 25 °C): δ 0.46 (d, *J* = 6.9 Hz, 3H, H15), 0.78-0.96 (m, 9H, H1a, H2a, H4a, H7, H14), 1.12-1.24 (m, 1H, H5), 1.28-1.47 (m, 1H, H3), 1.50-1.63 (m, 2H, H4b, H1b), 1.90 (pd, *J* = 7.0 Hz, *J* = 2.6 Hz, 1H, H8), 2.00-2.10 (m, 1H, H2b), 3.31 (td, *J* = 10.5 Hz, *J* = 4.3 Hz, 1H, H6), 4.08 (s, 3H, H10), 5.62 and 5.82 (d, *J* = 10.4 Hz, 2H, AB system, H13), 7.43 (t, *J* = 1.8 Hz, 1H, H11), 7.58 (t, *J* = 1.8 Hz, 1H, H9), 10.52 (s, 1H, H16). ^{13}C NMR (100 MHz, CDCl$_3$, 25 °C): *δ* 15.55 (C15), 20.92 (C7), 22.10 (C14), 22.74 (C1), 25.35 (C8), 31.12 (C3), 33.99 (C4), 36.73 (C10), 40.23 (C2), 47.71 (C5), 76.95 (C13), 79.61 (C6), 121.02 (C11), 123.86 (C9), 137.89 (C12). FTIR (cm^{-1}): 602 (ρ C–H, rocking),

674 (imidazole ring torsion), 735 (imidazole ring bending), 759 (δ C–H, imidazole), 869 (τ C–H, twisting), 1089 (ν_s C–O), 1145 (ν_{as} C–O), 1160 (ν C–N), 1365 (δ_s C–H, –CH_3), 1454 (δ C–H, –CH_2, bending), 1557 (imidazole ring stretching), 1647 (δ O–H, water residue), 2869, 2929, 2953 (ν_{s+as} C–H, –CH, –CH_2–, –CH_3 aliphatic), 3097 (ν_{s+as} C–H, imidazole), 3389 (ν_{s+as} O–H, water residue). HRMS (ESI +) m/z (%) calc. for $[C_{15}H_{27}N_2O]^+$: 251.2118, found: 251.2123. Elemental analysis: found: C, 62.79; H, 9.50; N, 9.77. Calc. for $C_{15}H_{27}ClN_2O$: C, 62.82; H, 9.52; N, 9.75%.

1-[(1R,2S,5R)-(–)-Menthoxymethyl]-3-methylimidazolium 1,1,2,2-tetrafluoroethanesulfonate [Men-Im-C_1][TFES] (**4a**). Yield: 99.1%, 0.049 mol, 21.43 g. ^1H NMR (400 MHz, $CDCl_3$, 25 °C) δ/ppm 0.48 (d, *J* = 7.0 Hz, 3H, H15), 0.73-0.99 (m, 9H, H1a, H2a, H4a, H7, H14), 1.16-1.28 (m, 1H, H5), 1.34-1.48 (m, 1H, H3), 1.54-1.67 (m, 2H, H1b, H4b), 1.87-2.05 (m, 2H, H2b, H8), 3.28 (td, *J* = 10.6 Hz, *J* = 4.2 Hz, 1H, H6), 3.99 (s, 3H, H10), 5.52 and 5.63 (d, *J* = 10.5 Hz, 2H, H13a and H13b, system AB), 6.17 (tt, *J* = 53.1 Hz, *J* = 6.1 Hz, 1H, H25), 7.44 (m, 2H, H9, H11), 9.33 (s, 1H, H16). ^{13}C NMR (100 MHz, $CDCl_3$, 25 °C) δ/ppm 15.49 (C15), 21.02 (C7), 22.13 (C14), 22.85 (C1), 25.47 (C8), 31.19 (C3), 34.11 (C4), 36.59 (C10), 40.18 (C2), 47.82 (C5), 77.11 (C13), 79.86 (C6), 109.22 (tt, *J* = 252.9 Hz, *J* = 29.8 Hz, C17), 113.85 (d, *J* = 284.3 Hz, C18), 121.47 (C11), 124.11 (C9), 137.10 (C12). ^{19}F NMR (100 MHz, $CDCl_3$, 25 °C) δ/ppm -135.34 (dt, *J* = 53.3 Hz, *J* = 8.6 Hz, F21, F22), -123.58 (td, *J* = 8.6 Hz, *J* = 6.0 Hz, F23, F24). FTIR (cm^{-1}): 525 (δ C–F, CF_2), 611 (ρ C–H, rocking), 653 (δ_{as} SO_3), 735 (imidazole ring bending), 759 (ν C–S), 813 (δ C–F, CF_2), 995 (ν_s SO_3), 1092 (ν_s C–O), 1102 (ν C–F, CF_2), 1123 (ν_{as} C–O), 1139 (ν C–N), 1220, 1237, 1264 (ν_{s+as} SO_3), 1371, 1389 (δ_s C–H, –CH_3), 1454 (δ C–H, –CH_2, bending), 1575 (imidazole ring stretching), 2872, 2918, 2952 (ν_{s+as} C–H, –CH, –CH_2–, –CH_3 aliphatic), 3065, 3143 (ν_{s+as} C–H, imidazole). HRMS (ESI+) m/z (%) calc. for $[C_{15}H_{27}N_2O]^+$: 251.2118; found: 251.2125. HRMS (ESI–) m/z (%) calc. for $[C_2HF_4SO_3]^-$: 180.9588; found: 180.9583. Elemental analysis: calc. (%) for $C_{17}H_{29}N_2O_4SF_4$ (432.47): C 47.21, H 6.53, N 6.48, found: C 47.33, H 6.46, N 6.40.

1-[(1R,2S,5R)-(–)-Menthoxymethyl]-3-methylimidazolium perfluorobutanesulfonate [Men-Im-C_1][PFBS] (**4b**). Yield: 98.0%, 0.049 mol, 26.97 g. ^1H NMR (400 MHz, $CDCl_3$, 25 °C) δ/ppm 0.49 (d, *J* = 6.9 Hz, 3H, H15), 0.74-0.99 (m, 9H, H1a, H2a, H4a, H7, H14), 1.20-1.27 (m, 1H, H5), 1.33-1.48 (m, 1H, H3), 1.55-1.68 (m, 2H, H1b, H4b), 1.87-2.05 (m, 2H, H2b, H8), 3.29 (td, *J* = 10.6 Hz, *J* = 4.3 Hz, 1H, H6), 4.00 (s, 3H, H10), 5.53 and 5.64 (d, *J* = 10.5 Hz, 2H, H13a, H13b, system AB), 7.41 (dt, *J* = 9.1 Hz, *J* = 1.9 Hz, 2H, H9, H11), 9.36 (s, 1H, H16). ^{13}C NMR (100 MHz, $CDCl_3$, 25 °C) δ/ppm 15.46 (C15), 21.01 (C7), 22.07 (C14), 22.84 (C1), 25.51 (C8), 31.17 (C3), 34.13 (C4), 36.62 (C10), 40.21 (C2), 47.85 (C5), 77.23 (C13), 80.02 (C6), 110.70-119.38 (m, 4C, C17, C18, C19, C20, weak), 121.42 (C11), 123.96 (C9), 137.21 (C12). ^{19}F NMR (100 MHz, $CDCl_3$, 25 °C) δ/ppm -125.92 (F21, F22), -121.59 (F23, F24), -114.80 (F25, F26), -80.80 (F27, F28, F29). FTIR (cm^{-1}): 524 (δ C–F, CF_3), 608 (ρ C–H, rocking), 654 (δ_{as} SO_3), 730 (imidazole ring bending), 750 (ν C–S), 802 (τ C–H, twisting), 844 (δ C–F, CF_2), 1005 (ν_s SO_3), 1047 (ν_s C–O), 1102 (ν C–F, CF_2, CF_3), 1132 (ν_{as} C–O), 1150 (ν C–N), 1230, 1254 (ν_{s+as} SO_3), 1352 (δ_s C–H, –CH_3), 1455 (δ C–H, –CH_2, bending), 1560 (imidazole ring

stretching), 2867, 2919, 2949 (ν_{s+as} C–H, –CH, –CH_2–, –CH_3 aliphatic), 3081, 3112, 3144 (ν_{s+as} C–H, imidazole). HRMS (ESI+) m/z (%) calc. for $[C_{15}H_{27}N_2O]^+$: 251.2118; found: 251,2125. HRMS (ESI–) m/z (%) calc. for $[C_4F_9O_3S]^-$: 298.9430; found: 298.9424. Elemental analysis: calc. (%) for $C_{19}H_{28}O_4N_2F_9S$ (550.48): C 41.455, H 4.94, N 5.09, found: C 41.38, H 5.05, N 5.00.

1-[(1R,2S,5R)-(–)-Menthoxymethyl]-3-methylimidazolium bis(trifluoromethylsulfonyl)imide [Men-Im-C_1][TFSI] (**4c**) Yield: 99.1%, 0.049 mol, 26.34 g. ^{1}H NMR (400 MHz, $CDCl_3$, 25 °C) δ/ppm: 0.50 (d, *J* = 7.0 Hz, 3H, H15), 0.75-1.00 (m, 9H, H1a, H2a, H4a, H7, H14), 1.19-1.30 (m, 1H, H5), 1.33-1.45 (m, 1H, H3), 1.56-1.69 (m, 2H, H1b, H4b), 1.96 (m, 2H, H2b, H8), 3.27 (td, *J* = 10.6 Hz, *J* = 4.3 Hz, 1H, H6), 3.98 (s, 3H, H10), 5.51 and 5.59 (d, *J* = 10.5 Hz, 2H, H13a, H13b), 7.37 (t, *J* = 1.8 Hz, 1H, H11), 7.43 (t, *J* = 1.8 Hz, 1H, H9), 8.96 (s, 1H, H16). ^{13}C NMR (100 MHz, $CDCl_3$, 25 °C) δ/ppm: 15.43 (C15), 21.01 (C7), 22.08 (C14), 22.86 (C1), 25.51 (C8), 31.23 (C3), 34.11 (C4), 36.62 (C10), 40.16 (C2), 47.81 (C5), 77.24 (C13), 80.13 (C6), 119.89 (q, $J_{F\text{-}C}$ = 321.0 Hz, 2C, C17, C18), 121.66 (C11), 124.17 (C9), 136.25 (C12). ^{19}F NMR (100 MHz, $CDCl_3$, 25 °C) δ/ppm: -78.92 (6F, F19, F20, F21, F22, F23, F24). FTIR (cm^{-1}): 510 (δ C–F, CF_3), 569 (ρ C–H, rocking), 611 (δ_{as} SO_2), 739 (imidazole ring bending), 788 (ν C–S), 841 (τ C–H, twisting), 952 (ν C–N), 1052 (ν_s C–O; ν S–N–S), 1097 (ν_{as} C–O), 1133 (ν_s SO_2), 1179 (ν C–F, CF_3), 1329, 1347 (ν_{as} SO_2), 1457 (δ C–H, –CH_2, bending), 1559, 1576 (imidazole ring stretching), 2873, 2928, 2957 (ν_{s+as} C–H, –CH, –CH_2–, –CH_3 aliphatic), 3152 (ν_{s+as} C–H, imidazole). HRMS (ESI+) m/z (%) calc. for $[C_{15}H_{27}N_2O]^+$: 251,2118; found: 251.2125. HRMS (ESI–) m/z (%) calc. for $[C_2F_6NO_4S_2]^-$: 279.9178; found: 279.9173. Elemental analysis: calc. (%) for $C_{17}H_{27}O_5N_3F_6S_2$ (531.53): C 38.41, H 5.12, N 7.905, found: C 38.48, H 5.19, N 7.830.

^{1}H NMR and ^{13}C NMR of *1-[(1R,2S,5R)-(–)-menthoxymethyl]-3-methylimidazolium bis(trifluoromethylsulfonyl)imide* (**4c**) reported previously.[7]

^{1}H NMR (600 MHz, $CDCl_3$, 25 °C): δ = 0.41 (d, *J* = 6.6 Hz, 3H,men.), 0.72-0.85 (m, 9H, men.), 1.14-1.17 (m, 1H, men.), 1.28-1.30 (m, 1H, men.), 1.51-1.57 (m, 2H, men.), 1.83-1.90 (m, 2H, men.), 3.18 (td, *J* = 10.2 Hz, *J* = 4.2 Hz, 1H, men.), 3.88 (s, 3H, CH_3-alkyl.im.), 5.42, 5.50 (d, *J* = 10.8 Hz, *J* = 10.2 Hz, 2H, AB system, N-CH_2-O), 7.28 (t, *J* = 1.8 Hz, 1H, im.), 7.34 (t, *J* = 1.8 Hz, 1H, im.), 8.86 (s, 1H, im.); ^{13}C NMR (151 MHz, $CDCl_3$, 25 °C): δ = 15.34 (men.), 20.89 (men.), 21.96 (men.), 22.77 (men.), 25.42 (men.), 31.12 (men.), 34.01 (men.), 36.50 (CH_3-alkyl.im.), 40.07 (men.), 47.72 (men.), 76.83 (men.), 80.06 (N-CH_2-O), 121.55 (im.), 124.06 (im.), 136.13 (im.); anion: 116.60, 118.72, 120.85, 122.98.

Both of the spectra show signals from functional groups at similar positions, indicating that the compounds share the same molecular structure, with differences in chemical shifts of between 0.1 to 0.2 ppm.

1-[(1R,2S,5R)-(–)-Menthoxymethyl]-3-methylimidazolium bis(pentafluoroethylsulfonyl)imide [Men-Im-C_1][PFSI] (**4d**). Yield: 98.6%, 0.049 mol, 31.14 g. ^{1}H NMR (400 MHz, $CDCl_3$, 25 °C) δ/ppm 0.49 (d, *J* = 7.0 Hz, 3H, H15), 0.74-1.00 (m, 9H, H1a, H2a, H4a, H7, H14), 1.19-1.30 (m, 1H, H5), 1.31-1.45 (m, 1H, H3), 1.56-1.69 (m, 2H, H1b, H4b), 1.87-2.02 (m, 2H, H2b, H8), 3.27 (td, *J* = 10.6 Hz, *J* = 4.3

Hz, 1H, H6), 3.96 (s, 3H, H10), 5.50 and 5.58 (d, J = 10.5 Hz, 2H, H13a and H13b, system AB), 7.38 (t, J = 1.8 Hz, 1H, H11), 7.42 (t, J = 1.8 Hz, 1H, H9), 8.94 (s, 1H, H16). ^{13}C NMR (100 MHz, $CDCl_3$, 25 °C) δ/ppm 15.33 (C15), 20.96 (C7), 22.01 (C14), 22.85 (C1), 25.50 (C8), 31.21 (C3), 34.10 (C4), 36.50 (C10), 40.14 (C2), 47.80 (C5), 77.19 (C13), 80.16 (C6), 108.04-120.28 (m, 4C, C17, C18, C19, C20), 121.64 (C11), 124.22 (C9), 136.20 (C12). ^{19}F NMR (100 MHz, $CDCl_3$, 25 °C) δ/ppm -117.23 (4F, F21, F22, F23, F24), -78.89 (6F, F25, F26, F27, F28, F29, F30). FTIR (cm^{-1}): 520 (δ C–F, CF_3), 609 (ρ C–H, rocking), 638 (δ_{as} SO_2), 730 (imidazole ring bending), 751 (ν C–S), 802 (τ C–H, twisting), 844 (δ C–F, CF_2), 1007 (ν C–N), 1032 (ν S–N–S), 1048 (ν_s C–O), 1099 (ν_s SO_2), 1132 (ν C–F, CF_2), 1148 (ν_{as} C–O), 1225 (ν C–F, CF_3), 1254 (ν_{as} SO_2), 1353 (δ_s C–H, –CH_3), 1455 (δ C–H, –CH_2, bending), 1561, 1576 (imidazole ring stretching), 2867, 2920, 2950 (ν_{s+as} C–H, –CH, –CH_2–, –CH_3 aliphatic), 3086, 3146 (ν_{s+as} C–H, imidazole). HRMS (ESI+) m/z (%) calc. for $[C_{15}H_{22}N_2O]^+$: 251.2118; found: 251.2125. HRMS (ESI–) m/z (%) calc. for $[C_4F_{10}NO_4S_2]^-$: 379.9115; found: 379.9109. Elemental analysis: calc. (%) for $C_{19}H_{27}O_5N_3F_{10}S_2$ (631.55): C 36.13, H 4.31, N 6.65, found: C 36.22, H 4.43, N 6.58.

1-[(1R,2S,5R)-(–)-Menthoxymethyl]-3-methylimidazolium trifluoromethanesulfonate [Men-Im-C_1][OTF] (**4e**). Yield: 99.0%, 0.049 mol, 19.82 g. ^{1}H NMR (400 MHz, $CDCl_3$, 25 °C) δ/ppm 0.49 (d, J = 7.0 Hz, 3H, H15), 0.73-0.99 (m, 9H, H1a, H2a, H4a, H7, H14), 1.17-1.28 (m, 1H, H5), 1.33-1.47 (m, 1H, H3), 1.55-1.68 (m, 2H, H1b, H4b), 1.87-2.05 (m, 2H, H2b, H8), 3.28 (td, J = 10.6 Hz, J = 4.3 Hz, 1H, H6), 4.00 (s, 3H, H10), 5.52 and 5.63 (d, J = 10.6 Hz, 2H, H13a, H13b, system AB), 7.45 (dd, J = 6.2 Hz, J = 2.0 Hz, 2H, H9, H11), 9.30 (s, 1H, H16). ^{13}C NMR (100 MHz, $CDCl_3$, 25 °C) δ/ppm 15.50 (C15), 21.03 (C7), 22.14 (C14), 22.85 (C1), 25.47 (C8), 31.18 (C3), 34.12 (C4), 36.61 (C10), 40.16 (C2), 47.83 (C5), 77.12 (C13), 79.85 (C6), 114.82-126.67 (m, C17), 121.51 (C11), 124.16 (C9), 136.99 (C12). ^{19}F NMR (100 MHz, $CDCl_3$, 25 °C) δ/ppm -78.54 (CF_3). FTIR (cm^{-1}): 518, 573, 625, 639, 728, 753, 875, 950, 1031, 1114, 1147, 1165, 1226, 1248, 1277, 1374 (δ_s C–H, –CH_3), 1456 (δ C–H, –CH_2, bending), 1560, 1576 (imidazole ring stretching), 2846, 2926, 2953 (ν_{s+as} C–H, –CH, –CH_2–, –CH_3 aliphatic), 3090, 3146 (ν_{s+as} C–H, imidazole). HRMS (ESI+) m/z (%) calc. for $[C_{15}H_{27}N_2O]^+$: 251.2118; found: 251.2125. HRMS (ESI–) m/z (%) calc. for $[CF_3SO_3]^-$: 148.9526; found: 148.9520. Elemental analysis: calc. (%) for $C_{16}H_{27}O_4N_2F_3S$ (400.46): C 47.99, H 6.80, N 6.995, found: C 48.07, H 6.72, N 6.91.

1-Decyloxymethyl-3-methylimidazolium chloride [C_{10}-Im-C_1][Cl] (**7**).

^{1}H NMR (400 MHz, $CDCl_3$, 25 °C) δ/ppm: 0.76- 0.85 (m, 3H, H15), 1.18 (s, 16H, H1-H8), 3.51 (t, J = 6.5 Hz, 2H, H14), 4.07 (s, 3H, H10), 5.69 (s, 2H, H13), 7.46 (d, J = 1.9 Hz, 1H, H11), 7.62 (d, J = 2.0 Hz, 1H, H9), 10.41 (s, 1H, H16). ^{13}C NMR (100 MHz, $CDCl_3$, 25 °C) δ/ppm: 14.15 (C15), 22.71 (C8), 25.93 (C2), 29.31 (C6), 29.34 (C5), 29.39 (C1), 29.57 (C3, C4), 31.92 (C7), 36.88 (C10), 70.80 (C14), 79.28 (C13), 121.01 (C11), 124.14 (C9), 138.10 (C12). FTIR (cm^{-1}): 610 (ρ C–H, rocking), 731 (imidazole ring bending), 867 (τ C–H, twisting), 1119 (ν_{s+as} C–O), 1167 (ν C–N), 1466 (δ C–H, –CH_2, bending), 1553, 1577 (imidazole ring stretching), 1630 (δ O–H, water residue), 2851, 2919 (ν_{s+as} C–H,

–CH_2–, –CH_3 aliphatic), 3029, 3077 (ν_{s+as} C–H, imidazole), 3394, 3449 (ν_{s+as} O–H, water residue). HRMS (ESI+) m/z (%) calc. for $[C_{15}H_{29}N_2O]^+$: 253.2274; found: 253.2273. Elemental analysis: calc. (%) for $C_{15}H_{29}N_2OCl$ (288.86): C 62.37, H 10.12, N 9.70, found: C 62.45, H 10.21, N 9.61.

1-Decyloxymethyl-3-methylimidazolium 1,1,2,2-tetrafluoroethanesulfonate [C_{10}-Im-C_1][TFES] (**8a**). Yield: 97.9%, 0.048 mol, 26.94 g. ^{1}H NMR (400 MHz, $CDCl_3$, 25 °C) δ/ppm 0.86 (s, 3H, H15), 1.24 (s, 14H, H2-H8), 1.54 (s, 2H, H1), 3.51 (s, 2H, H14), 3.99 (s, 3H, H10), 5.54 (s, 2H, H13), 6.15 (tt, J = 53.0 Hz, J = 6.0 Hz, 1H, H23), 7.42 (s, 2H, H9, H11), 9.23 (s, 1H, H16). ^{13}C NMR (100 MHz, $CDCl_3$, 25 °C) δ/ppm 14.21 (C15), 22.78 (C8), 25.92 (C2), 29.28 (C5 and C6), 29.41 (C1), 29.63 (C3 and C4), 31.99 (C7), 36.67 (C10), 70.94 (C14), 79.49 (C13), 109.24 (tt, J = 252.5 Hz, J = 29.3 Hz, C17), 113.87 (tt, J = 282.6 Hz, J = 24.3 Hz, C18), 121.20 (C11), 124.15 (C9), 137.26 (C12). ^{19}F NMR (100 MHz, $CDCl_3$, 25 °C) δ/ppm -135.40 (dt, J = 52.7 Hz, J = 7.9 Hz, 2F, F21, F22), -123.50 (m, F23 and F24). FTIR (cm^{-1}): 522 (δ C–F, CF_2), 608 (ρ C–H, rocking), 652 (δ_{as} SO_3), 738 (imidazole ring bending), 818 (δ C–F, CF_2), 994 (ν_s SO_3), 1103 (ν C–F, CF_2), 1136 (ν_{as} C–O), 1159 (ν C–N), 1244 (ν_{s+as} SO_3), 1388 (δ_s C–H, –CH_3), 1466 (δ C–H, –CH_2, bending), 1559, 1578 (imidazole ring stretching), 2854, 2924 (ν_{s+as} C–H, –CH_2–, –CH_3 aliphatic), 3111, 3151 (ν_{s+as} C–H, imidazole). HRMS (ESI+) m/z (%) calc. for $[C_{15}H_{29}N_2O]^+$: 253.2274; found: 253.2282. HRMS (ESI–) m/z (%) calc. for $[C_2HF_4O_3S]^-$: 180.9588; found: 180.9586. Elemental analysis: calc. (%) for $C_{17}H_{28}O_4N_2F_4S$ (432.47): C 47.21, H 6.53, N 6.48, found: C 47.31, H 6.59, N 6.41.

1-Decyloxymethyl-3-methylimidazolium perfluorobutanesulfonate [C_{10}-Im-C_1][PFBS] (**8b**). Yield: 99.1%, 0.049 mol, 21.43 g. ^{1}H NMR (400 MHz, $CDCl_3$, 25 °C) δ/ppm: 0.87 (t, J = 6.4 Hz, 3H, H15), 1.24 (s, 14H, H2-H8), 1.54 (q, J = 7.5 Hz, 2H, H1), 3.47-3.56 (m, 2H, H14), 3.99-4.01 (m, 3H, H10), 5.54-5.55 (m, 2H, H13), 7.39-7.41 (m, 2H, H9, H11), 9.26 (s, 1H, H16). ^{13}C NMR (100 MHz, $CDCl_3$, 25 °C) δ/ppm: 14.19 (C15), 22.78 (C8), 25.89 (C2), 29.27 (C5,C6), 29.41 (C1), 29.62 (C3, C4), 31.99 (C7), 36.66 (C10), 71.00 (C14), 79.56 (C13), 110.11-119.64 (m, 4C, C17, C18, C19, C20), 121.11 (C11), 124.03 (C9), 137.38 (C12). ^{19}F NMR (100 MHz, $CDCl_3$, 25 °C) δ/ppm: -125.88 (F21, F22), -121.59 (F23, F24), -114.77 (F25, F26), -80.83 (F27, F28, F29). FTIR (cm^{-1}): 524 (δ C–F, CF_3), 616 (ρ C–H, rocking), 655 (δ_{as} SO_3), 735 (imidazole ring bending), 746 (ν C–S), 802 (τ C–H, twisting), 869 (δ C–F, CF_2), 1005 (ν_s SO_3), 1056 (ν_s C–O), 1132 (ν C–F, CF_2, CF_3; ν_{as} C–O; ν C–N), 1209, 1232, 1251 (ν_{s+as} SO_3), 1352 (δ_s C–H, –CH_3), 1468 (δ C–H, –CH_2, bending), 1560, 1578 (imidazole ring stretching), 2856, 2926 (ν_{s+as} C–H, –CH_2–, –CH_3 aliphatic), 3112, 3151 (ν_{s+as} C–H, imidazole). HRMS (ESI+) m/z (%) calc. for $[C_{15}H_{29}N_2O]^+$: 253.2274; found: 253.2284. HRMS (ESI–) m/z (%) calc. for $[C_4F_9O_3S]^-$: 298.9430; found: 298.9424. Elemental analysis: calc. (%) for $C_{19}H_{27}O_4N_2F_9S$ (550.48): C 41.455, H 4.94, N 5.09, found: C 41.37, H 4.88, N 5.18.

3.2. The Impact of Anion Variation on NMR Chemical Shifts

^{1}H NMR spectroscopy reveals significant variations in the chemical shift values of specific protons within the imidazolium cation depending on the type of counterion present. The ^{1}H chemical shifts and assignments for the protons of the salts of 1-[(1*R*,2*S*,5*R*)-(–)-menthoxymethyl]-3-methylimidazolium

and 1-decyloxymethyl-3-methylimidazolium are summarised in Tables S2 and S3, respectively. Stacked ^{1}H NMR spectra are depicted in Figure S46 and Figure S47.

Notable downfield shifts were observed for protons in proximity to the positively charged nitrogen atoms of the imidazolium ring for all synthesised chlorides and FILs (refer to Tables S2 and S3). These changes are attributed to the steric effects of electron clouds and the modulation of electrostatic interactions between the cation and the respective anion. This aligns with previous studies in which the type and size of the anion were found to directly influence the electron density distribution within the cationic framework.[8]

The most pronounced effect is observed for the H16 proton, which is located at the C2 position of the imidazolium ring and consistently shows the lowest electron density due to its unique electronic environment. The chemical shift of H16 decreases from 10.52 ppm for the chloride salt ([Men-Im-C_1][Cl]) to 8.94 ppm for the FIL containing the $[PFSI]^-$ anion, resulting in a maximal $\Delta\delta$ of 1.58 ppm (see Table S2). This trend reflects increasing shielding as the anion becomes bulkier and more fluorinated, suggesting a redistribution of electron density from the anion towards the cationic centre. Other protons within the imidazolium and (1*R*,2*S*,5*R*)-(–)-menthoxymethyl moieties (*e.g.* H9, H11, H13a/b, H10 and H6) exhibit smaller, yet consistent, shifts in response to anion exchange, with maximum $\Delta\delta$ values of up to ~ 0.2 ppm. Signal splitting or multiplicity changes for H9 and H11 were observed for some anion systems, such as $[TFSI]^-$ and $[TFES]^-$, which is likely due to differing conformational dynamics or minor anisotropic effects introduced by the anion environment.

A similar pattern is observed in the corresponding [C_{10}-Im-C_1] salts (see Table S3 and Figure S47). The H16 chemical shift decreases from 10.41 ppm in the chloride salt to 9.23 ppm in the [TFES] salt and 9.26 ppm in the [PFBS] salt, which confirms that the shielding trend is consistent across imidazolium cation types.

Based on these data, the order of increasing shielding effect of the anion can be established as follows:

$$[Cl]^- < [PFBS]^- < [TFES]^- < [OTF]^- < [TFSI]^- < [PFSI]^-.$$

This sequence reflects the increasing size and delocalisation capacity of the anions, which supports the conclusion that electron-withdrawing, spatially extended anions reduce the deshielding effect around the imidazolium core. The most effective shielding is observed with sulfonylimides-based anions: $[TFSI]^-$ and $[PFSI]^-$.

On the other hand, ^{13}C NMR spectra show negligible changes in carbon chemical shifts upon anion exchange, particularly for the ring carbons and aliphatic side chains. This corroborates earlier findings that the impact of the anion is predominantly localised to the proton environment.

These observations are consistent with previous reports and emphasise the significance of anion selection in regulating local electron density.[9,10] This, in turn, may impact the physicochemical properties and reactivity of FIL systems.

3.3. Refractive Index of FILs Obtained (4c–4d, 8a–8b)

The refractive index (RI) of ILs is a fundamental physicochemical parameter describing the degree to which light is bent as it passes through a medium. It reflects the medium's electronic polarizability, molecular density, and degree of structural organisation.[11,12] For FILs, particularly those incorporating hydrogen-bond donors or acceptors within their molecular architecture, the RI becomes a sensitive optical probe for characterising the strength and nature of intermolecular interactions, including hydrogen bonding and ionic clustering.[13,14]

Table 1 shows the RI values for the synthesised FILs (**4c**, **4d**, **8a** and **8b**), which were measured in the liquid state over a temperature range of 20 to 60 °C. Figure S11 illustrates the temperature-dependent RI trends and reveals two consistent patterns:

- Linear decrease in RI with increasing temperature: All of the tested FILs display a nearly linear decrease in RI with temperature. This behaviour is consistent with the thermal expansion of the liquid and the resulting reduction in molecular packing density. This suggests a temperature-induced weakening of cohesive forces, including hydrogen bonds and ion–ion interactions, which reduces the medium's polarizability.[15–17]
- Anion-dependent modulation of RI: clear differences in RI values are observed within each structural family of FILs as a function of anion type. Specifically:

  For menthol-based FILs: $[PFSI]^- < [TFSI]^- \rightarrow$ RI (**4d**) < RI (**4c**).

  For 1-decanol-based FILs: $[PFBS]^- < [TFES]^- \rightarrow$ RI (**8b**) < RI (**8a**).

  This trend correlates with the hydrogen bond acceptor capacity of the anions. Those with greater basicity and a stronger affinity for hydrogen bonding (*e.g.* $[TFSI]^-$ and $[TFES]^-$) promote tighter ion-pair aggregation and increased electronic polarizability. This leads to elevated RI values.[11,13,14]

These observations are consistent with the reported effects of ionic structures on the optical properties of both protic and aprotic ILs.[16,18] Furthermore, the preservation of hydrogen-bond networks at elevated temperatures in ILs may contribute to their robustness as advanced functional media in optical, sensing and separation technologies.[18,19] The responsiveness of RI to both temperature and ionic structure confirms its usefulness as a diagnostic parameter for evaluating subtle molecular interactions in designer ILs, including those intended for use in environmentally responsive applications.

3.4. Supplementary Tables

Table S1. Physicochemical Properties of 1-[(1*R*,2*S*,5*R*)-(–)-Menthoxymethyl]-3-methylimidazolium [Men-Im-C_1][Cl] (3) and 1-Decyloxymethyl-3-methylimidazolium [C_{10}-Im-C_1][Cl] (7) Chloride

compound[*]	physical state[a]	melting point T_m			thermal stability[d]		specific rotation[e,f,g] $[\alpha]_D^{25}$
		digital T_m apparatus[b] (°C)	DSC curves[c] (°C)	literature values (°C)	$T_{5\%onset}$ (°C)	$T_{50\%onset}$ (°C)	
**3**	white crystal	132.6–133.0	131.20	137.0–138.0	115.90	208.17	−116.6
**7**	white crystal	57.9–60.9	57.96	48.5–52.6	103.84	177.25	–

[*]Compounds known in the literature.[20,21] [a]At 25°C. [b]Data obtained from an electrothermal digital melting point apparatus with accuracy ± 0.1 °C. [c]Accuracy ± 0.01 °C. The values of T_m were taken as the peak temperature of the transition upon heating/cooling cycles. Experiments were carried out with a heating/cooling rate of 10 °C min^{-1}. [d]Experiments were carried out with a heating rate of 5 °C min^{-1}. [e]In methylene chloride. [f]Standard uncertainty for specific rotation u is $u(\alpha) = \pm 0.5°$. [g]Standard uncertainty for concentration u is $u(c) = \pm 0.00002$ g·mL^{-1}.

Table S2. The Comparison of ^{1}H Chemical Shifts (ppm) for Selected Protons of 1-[(1*R*,2*S*,5*R*)-(–)-Menthoxymethyl]-3-methylimidazolium Cation Combined with Different Anions

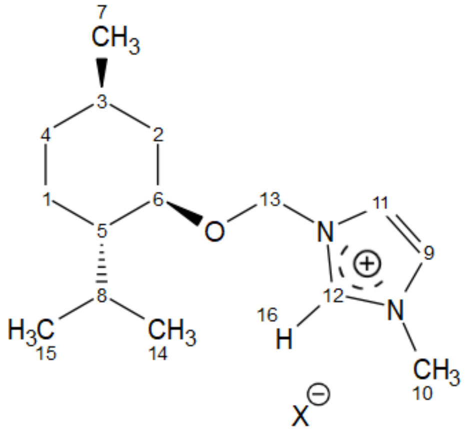

compound	shifts[a,b]						
	H16	H9	H11	H13a	H13b	H10	H6
[Men-Im-C_1][Cl] (**3**)	10.52	7.58	7.43	5.82	5.62	4.08	3.31
[Men-Im-C_1][TFES] (**4a**)	9.33	7.44	7.43	5.63	5.52	3.99	3.28
[Men-Im-C_1][PFBS] (**4b**)	9.36	7.42	7.40	5.64	5.53	4.00	3.29
[Men-Im-C_1][TFSI] (**4c**)	8.96	7.43	7.37	5.59	5.51	3.98	3.27
[Men-Im-C_1][PFSI] (**4d**)	8.94	7.42	7.38	5.58	5.50	3.96	3.27
[Men-Im-C_1][OTF] (**4e**)	9.30	7.45	7.45	5.63	5.52	4.00	3.28

[a]Spectra were performed in $CDCl_3$. [b]Shifts are presented in ppm.

Table S3. The Comparison of ^{1}H Chemical Shifts (ppm) for Selected Protons of 1-Decyloxymethyl-3-methylimidazolium Cation Combined with Different Anions

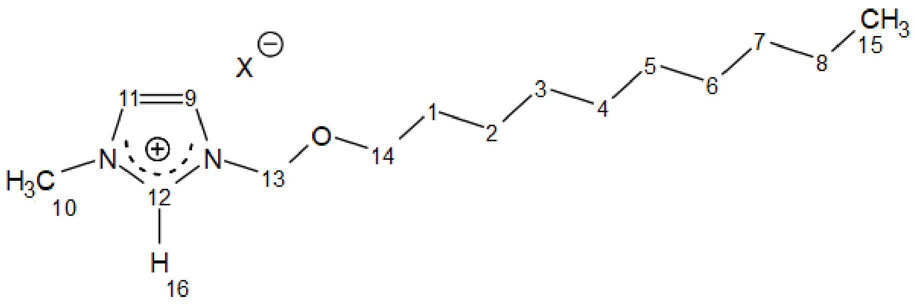

compound	shifts[a,b]				
	H16	H9	H11	H13	H10
[C_{10}-Im-C_1][Cl] (**7**)	10.41	7.62	7.46	5.69	4.07
[C_{10}-Im-C_1][TFES] (**8a**)	9.23	7.42	7.42	5.56	3.99
[C_{10}-Im-C_1][PFBS] (**8b**)	9.26	7.41	7.41	5.55	4.00

[a]Spectra were performed in $CDCl_3$. [b]Shifts are presented in ppm.

Table S4. Effect of FILs on Common Radish Seed Emergence. Data are Expressed as a Mean ± SD of Three Replicates for Each Concentration ($mg \cdot kg^{-1}$ Soil Dry Weight, DW)

FIL	concentration ($mg \cdot kg^{-1}$ soil DW)							
	control	1	10	50	100	400	700	1000
**4a**	$18{\pm}1^{a}$	$18{\pm}2^{a}$	$18{\pm}1^{a}$	$19{\pm}1^{a}$	$19{\pm}0^{a}$	$14{\pm}3^{ab}$	$14{\pm}2^{ab}$	$9{\pm}3^{b}$
**8a**	$18{\pm}0^{a}$	$18{\pm}2^{a}$	$19{\pm}1^{a}$	$19{\pm}1^{a}$	$18{\pm}2^{a}$	$19{\pm}1^{a}$	$16{\pm}3^{ab}$	$14{\pm}1^{b}$
**4b**	$19{\pm}1^{a}$	$19{\pm}0^{a}$	$20{\pm}0^{a}$	$19{\pm}1^{a}$	$19{\pm}1^{a}$	$15{\pm}1^{b}$	$9{\pm}2^{c}$	$2{\pm}1^{d}$
**8b**	$19{\pm}2^{a}$	$18{\pm}2^{a}$	$19{\pm}1^{a}$	$18{\pm}1^{a}$	$19{\pm}1^{a}$	$16{\pm}1^{ab}$	$12{\pm}2^{b}$	$7{\pm}1^{c}$
**4c**	$18{\pm}2^{a}$	$18{\pm}2^{a}$	$17{\pm}1^{a}$	$18{\pm}2^{a}$	$17{\pm}1^{a}$	–	–	–
**4d**	$19{\pm}0^{a}$	$18{\pm}1^{a}$	$19{\pm}2^{a}$	$18{\pm}1^{a}$	$17{\pm}2^{a}$	$8{\pm}4^{b}$	$2{\pm}1^{c}$	$1{\pm}1^{c}$
**4e**	$19{\pm}1^{a}$	$19{\pm}0^{a}$	$19{\pm}2^{a}$	$18{\pm}0^{a}$	$16{\pm}2^{a}$	$12{\pm}2^{b}$	$11{\pm}2^{b}$	$10{\pm}0^{c}$

[a-d]Values denoted by the same letters in the columns do not differ statistically at $p < 0.05$.

Table S5. The Inhibition Rate for Shoot Length and Root Length (%) of Common Radish After Exposure to FILs. Data are Expressed as a Mean ± SD of Three Replicates for Each Concentration (mg·kg^{-1} Soil Dry Weight, DW)

FIL	concentration (mg·kg^{-1} soil DW)						
	1	10	50	100	400	700	1000
	shoot length inhibition (%)						
**4a**	3.204±7.368^{c}	–0.596±5.960^{c}	0.580±5.836^{c}	5.537±5.914^{c}	41.398±8.232^{b}	65.597±4.636^{a}	68.512±8.621^{a}
**8a**	0.543±4.295^{d}	0.735±6.095^{d}	5.145±5.477^{d}	22.084±8.803^{c}	65.356±6.681^{b}	80.697±3.585^{a}	83.893±5.569^{a}
**4b**	–0.842±3.576^{d}	–1.799±3.043^{d}	–1.607±4.704^{d}	–3.138±4.534^{d}	28.243±5.741^{c}	51.014±6.181^{b}	59.434±4.586^{a}
**8b**	2.934±6.591^{e}	1.878±6.088^{e}	7.805±7.428^{e}	28.228±7.394^{d}	74.765±8.642^{c}	86.385±4.877^{b}	100.000±0.000^{a}
**4c**	–6.360±7.620^{e}	0.000±5.965^{d}	20.495±7.223^{c}	55.124±5.619^{b}	100.000±0.000^{a}	100.000±0.000^{a}	100.000±0.000^{a}
**4d**	–1.725±5.757^{d}	1.656±5.533^{d}	13.734±6.083^{c}	30.642±6.999^{b}	100.000±0.000^{a}	100.000±0.000^{a}	100.000±0.000^{a}
**4e**	–6.579±8.565^{f}	–7.754±9.598^{f}	7.135±5.066^{d}	18.890±8.941^{d}	77.274±4.368^{c}	87.775±4.719^{b}	100.000±0.000^{a}
	root length inhibition (%)						
**4a**	–2.509±7.944^{e}	5.381±7.488^{d}	10.093±5.370^{d}	10.201±11.951^{d}	51.976±5.594^{c}	65.670±4.026^{b}	84.042±2.779^{a}
**8a**	–25.208±8.419^{e}	–11.260±9.575^{d}	–32.103±7.517^{e}	–23.442±9.150^{e}	61.796±7.620^{c}	70.982±6.695^{b}	83.333±6.202^{a}
**4b**	8.775±9.328^{c}	8.874±9.202^{c}	6.989±7.490^{c}	1.893±8.820^{c}	57.664±8.663^{b}	86.681±5.080^{a}	93.094±4.944^{a}
**8b**	–4.474±9.638^{d}	–2.040±9.585^{d}	–4.134±9.548^{c}	12.626±6.931^{c}	72.949±11.284^{b}	85.987±4.586^{a}	90.291±2.291^{a}
**4c**	–11.842±7.433^{d}	–6.483±9.660^{cd}	–0.184±7.649^{c}	37.394±6.401^{b}	95.856±1.339^{a}	100.000±0.000^{a}	100.000±0.000^{a}
**4d**	–4.372±6.912^{e}	0.324±9.001^{e}	15.907±8.286^{d}	31.277±9.206^{c}	88.099±4.079^{b}	95.989±2.130^{a}	100.000±0.000^{a}
**4e**	–6.811±10.220^{e}	0.295±6.153^{de}	3.350±8.053^{de}	11.155±6.903^{c}	67.436±7.540^{b}	91.395±3.413^{a}	94.999±2.306^{a}

[a-f]Values denoted by the same letters in the columns do not differ statistically at $p < 0.05$.

Table S6. Fresh Weight Yield (g·pot^{-1}) of Common Radish Plants Grown in Soil with the Addition of FILs. Data are Expressed as a Mean ± SD of Three Replicates for Each Concentration (mg·kg^{-1} Soil Dry Weight, DW).

FIL	concentration (mg·kg^{-1} soil DW)							
	control	1	10	50	100	400	700	1000
**4a**	5.054±0.560^{a}	4.712±0.031^{a}	5.041±0.087^{a}	4.988±0.493^{a}	4.772±0.039^{a}	1.787±0.664^{b}	0.857±0.165^{bc}	0.667±0.199^{c}
**8a**	5.323±0.156^{ab}	5.554±0.139^{a}	4.729±0.053^{bc}	4.477±0.228^{c}	3.056±0.449^{d}	1.076±0.127^{e}	0.554±0.197^{e}	0.494±0.0048^{e}
**4b**	6.283±0.207^{ab}	6.263±0.687^{ab}	7.216±0.756^{a}	6.776±0.168^{b}	5.223±0.621^{b}	1.353±0.266^{c}	0.726±0.199^{c}	–
**8b**	6.342±0.059^{a}	6.237±0.721^{ab}	5.962±0.027^{ab}	5.384±0.306^{b}	3.742±0.047^{c}	0.803±0.346^{d}	0.355±0.110^{d}	–
**4c**	5.054±0.180^{a}	5.063±0.337^{a}	4.728±0.245^{a}	3.626±0.355^{b}	1.550±0.179^{c}	–	–	–
**4d**	4.632±0.142^{a}	4.507±0.362^{a}	4.762±0.157^{a}	3.862±0.554^{a}	2.512±0.632^{b}	–	–	–
**4e**	4.558±0.100^{a}	4.727±0.142^{a}	4.655±0.514^{a}	3.913±0.558^{a}	2.848±0.076^{b}	0.439±0.064^{c}	–	–

$^{a\text{-}e}$Values denoted by the same letters in the columns do not differ statistically at $p < 0.05$.

Table S7. Effect of FILs on the Dry Weight in Common Radish Plants ($g \cdot g^{-1}$ Plant DW). Data are Expressed as a Mean ± SD of Three Replicates for Each Concentration ($mg \cdot kg^{-1}$ Soil Dry Weight, DW)

FIL	concentration ($mg \cdot kg^{-1}$ soil DW)							
	control	1	10	50	100	400	700	1000
**4a**	0.0600±0.0035[de]	0.0518±0.0045[f]	0.0532±0.0020[ef]	0.0590±0.0041[def]	0.0615±0.0030[d]	0.0970±0.0011[c]	0.1656±0.0018[b]	0.1864±0.0035[a]
**8a**	0.0528±0.0010[d]	0.0533±0.0020[d]	0.0578±0.0012[d]	0.0600±0.0025[d]	0.0741±0.0072[c]	0.1027±0.0049[b]	0.1092±0.0008[ab]	0.1148±0.0003[a]
**4b**	0.0613±0.0026[c]	0.0647±0.0039[c]	0.0673±0.0030[c]	0.0630±0.0038[c]	0.0580±0.0029[c]	0.1399±0.0168[b]	0.2400±0.0264[a]	–
**8b**	0.0549±0.0013[cd]	0.0534±0.0007[d]	0.0548±0.0023[d]	0.0602±0.0009[c]	0.0753±0.0042[b]	0.1237±0.0021[a]	0.1252±0.0041[a]	–
**4c**	0.0631±0.0005[b]	0.0640±0.0039[b]	0.0664±0.0024[b]	0.0709±0.0048[b]	0.1090±0.0018[a]	–	–	–
**4d**	0.0692±0.0057[b]	0.0673±0.0007[b]	0.0662±0.0026[b]	0.0677±0.0039[b]	0.0926±0.0120[a]	–	–	–
**4e**	0.0693±0.0037[bc]	0.0679±0.0026[c]	0.0719±0.0028[bc]	0.0712±0.0021[bc]	0.0825±0.0110[b]	0.1251±0.0050[a]	–	–

[a-f]Values denoted by the same letters in the columns do not differ statistically at $p < 0.05$.

Table S8. Chlorophyll a Content (Chl a) and Chlorophyll b Content (Chl b) in Common Radish Leaves (mg·g^{-1} Plant Dry Weight, DW) Growing on Soil Supplemented with FILs (mg·kg^{-1} Soil Dry Weight, DW)

FIL	concentration (mg·kg^{-1} soil DW)							
	control	1	10	50	100	400	700	1000
	Chl a (mg·g^{-1} plant DW)							
**4a**	10.027±0.055^{c}	11.750±0.168^{a}	11.162±0.055^{b}	11.779±0.265^{a}	10.724±0.357^{b}	1.901±0.050^{d}	1.117±0.068^{e}	0.664±0.015^{e}
**8a**	14.028±0.062^{a}	13.865±0.052^{b}	12.261±0.084^{d}	12.448±0.029^{c}	12.433±0.040^{c}	9.764±0.047^{e}	7.093±0.007^{f}	5.872±0.014^{g}
**4b**	8.574±0.122^{b}	9.145±0.113^{a}	8.429±0.086^{b}	7.314±0.086^{b}	6.225±0.101^{d}	1.420±0.040^{e}	0.515±0.002^{f}	–
**8b**	14.467±0.119^{a}	12.487±0.085^{b}	11.798±0.363^{b}	11.912±0.562^{b}	11.724±0.136^{b}	10.821±0.230^{c}	5.919±0.185^{d}	–
**4c**	9.424±0.165^{a}	9.402±0.113^{a}	8.594±0.121^{b}	5.741±0.017^{c}	2.689±0.039^{d}	–	–	–
**4d**	10.134±0.022^{b}	11.500±0.077^{a}	9.919±0.022^{c}	7.028±0.054^{d}	5.713±0.033^{e}	–	–	–
**4e**	9.343±0.083^{b}	9.766±0.082^{a}	8.765±0.034^{c}	8.481±0.083^{d}	3.263±0.005^{e}	1.269±0.081^{f}	–	–
	Chl b (mg·g^{-1} plant DW)							
**4a**	3.325±0.041^{b}	3.375±0.200^{ab}	3.718±0.090^{a}	3.329±0.245^{b}	3.222±0.106^{b}	0.795±0.005^{c}	0.436±0.028^{d}	0.175±0.020^{d}
**8a**	3.979±0.060^{a}	3.901±0.025^{a}	3.314±0.078^{b}	3.421±0.031^{b}	3.323±0.014^{b}	2.486±0.009^{c}	1.781±0.012^{d}	1.363±0.011^{e}
**4b**	2.257±0.033^{a}	2.518±0.328^{a}	2.362±0.204^{a}	1.808±0.074^{b}	1.507±0.069^{b}	0.354±0.009^{c}	0.095±0.001^{c}	–
**8b**	6.386±0.062^{a}	6.252±0.042^{ab}	5.893±0.155^{bc}	5.630±0.150^{c}	5.024±0.357^{d}	3.919±0.087^{e}	2.596±0.080^{f}	–
**4c**	3.346±0.126^{a}	2.983±0.182^{b}	2.376±0.039^{c}	1.344±0.034^{d}	0.464±0.043^{e}	–	–	–
**4d**	3.301±0.011^{a}	3.349±0.011^{a}	3.087±0.029^{b}	1.920±0.035^{c}	1.750±0.049^{d}	–	–	–
**4e**	2.503±0.032^{b}	2.774±0.154^{a}	2.292±0.059^{c}	2.229±0.034^{c}	0.943±0.056^{d}	0.461±0.045^{e}	–	–

$^{a\text{-}g}$Values denoted by the same letters in the columns do not differ statistically at $p < 0.05$.

Table S9. Changes in Chl a/Chl b and Chl a+b/Car Values in Leaves of Common Radish Growing in Soil with the Addition of FILs (mg·kg^{-1} Soil Dry Weight, DW)

FIL	concentration (mg·kg^{-1} soil DW)							
	control	1	10	50	100	400	700	1000
	Chl a/Chl b							
**4a**	3.016±0.024^{bc}	3.488±0.173^{ab}	3.003±0.076^{bc}	3.550±0.249^{a}	3.329±0.021^{ab}	2.391±0.051^{d}	2.566±0.114^{cd}	3.824±0.382^{a}
**8a**	3.526±0.047^{e}	3.554±0.014^{de}	3.701±0.067^{c}	3.639±0.040^{cd}	3.742±0.023^{c}	3.927±0.032^{b}	3.982±0.026^{b}	4.308±0.036^{a}
**4b**	3.801±0.100^{b}	3.676±0.500^{b}	3.588±0.334^{b}	4.050±0.156^{b}	4.134±0.163^{b}	4.019±0.215^{b}	5.446±0.106^{a}	–
**8b**	2.266±0.033^{bc}	1.997±0.020^{d}	2.002±0.013^{d}	2.115±0.044^{cd}	2.342±0.184^{b}	2.761±0.010^{a}	2.280±0.022^{bc}	–
**4c**	2.818±0.072^{d}	3.158±0.168^{cd}	3.618±0.103^{bc}	4.274±0.100^{b}	5.822±0.529^{a}	–	–	–
**4d**	3.070±0.003^{d}	3.434±0.015^{b}	3.213±0.026^{c}	3.660±0.041^{a}	3.266±0.075^{c}	–	–	–
**4e**	3.732±0.050^{a}	3.527±0.196^{a}	3.826±0.109^{a}	3.806±0.094^{a}	3.467±0.203^{a}	2.766±0.247^{b}	–	–
	(Chl a+Chl b)/Car							
**4a**	4.978±0.050^{a}	4.756±0.077^{ab}	4.836±0.122^{a}	4.906±0.045^{a}	4.742±0.020^{ab}	4.077±0.073^{c}	5.000±0.159^{a}	4.502±0.231^{b}
**8a**	5.047±0.012^{cd}	5.109±0.024^{c}	4.918±0.051^{e}	4.915±0.028^{e}	5.321±0.012^{b}	5.498±0.029^{a}	5.005±0.028^{d}	4.788±0.024^{f}
**4b**	5.070±0.080^{ab}	4.751±0.358^{b}	5.152±0.263^{ab}	4.448±0.048^{c}	5.089±0.210^{ab}	5.342±0.086^{a}	4.365±0.084^{c}	–
**8b**	5.747±0.035^{ab}	6.429±0.058^{a}	6.427±0.325^{a}	6.488±0.246^{a}	5.600±0.566^{b}	5.114±0.181^{b}	5.079±0.066^{b}	–
**4c**	6.507±0.170^{a}	6.050±0.287^{ab}	5.737±0.129^{bc}	5.419±0.106^{c}	4.094±0.258^{d}	–	–	–
**4d**	5.224±0.024^{a}	4.923±0.052^{b}	5.143±0.054^{a}	5.202±0.049^{a}	5.121±0.108^{a}	–	–	–
**4e**	4.683±0.068^{c}	4.913±0.140^{bc}	4.512±0.061^{c}	4.698±0.039^{c}	5.390±0.236^{b}	6.438±0.329^{a}	–	–

$^{a\text{-}f}$Values denoted by the same letters in the columns do not differ statistically at $p < 0.05$.

3.7. Supplementary Figures

TGA thermogram of FILs

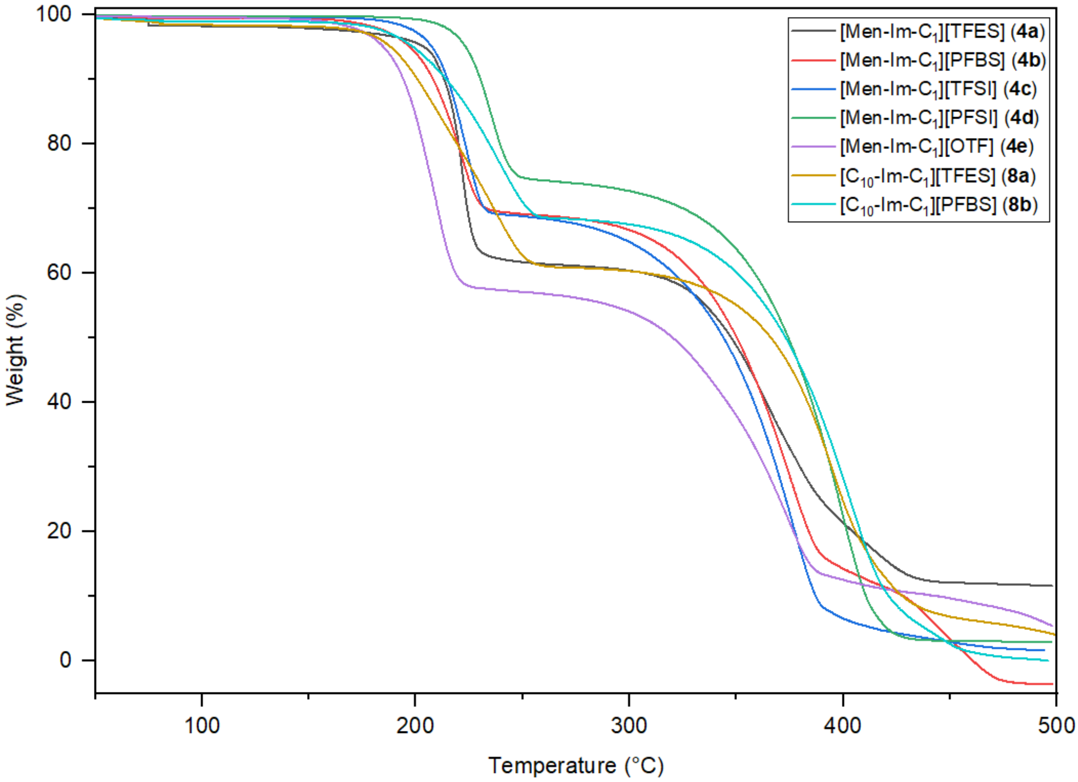

Figure S1. TGA thermograms of the synthesised FILs (**4a**–**4e**, **8a**, **8b**).

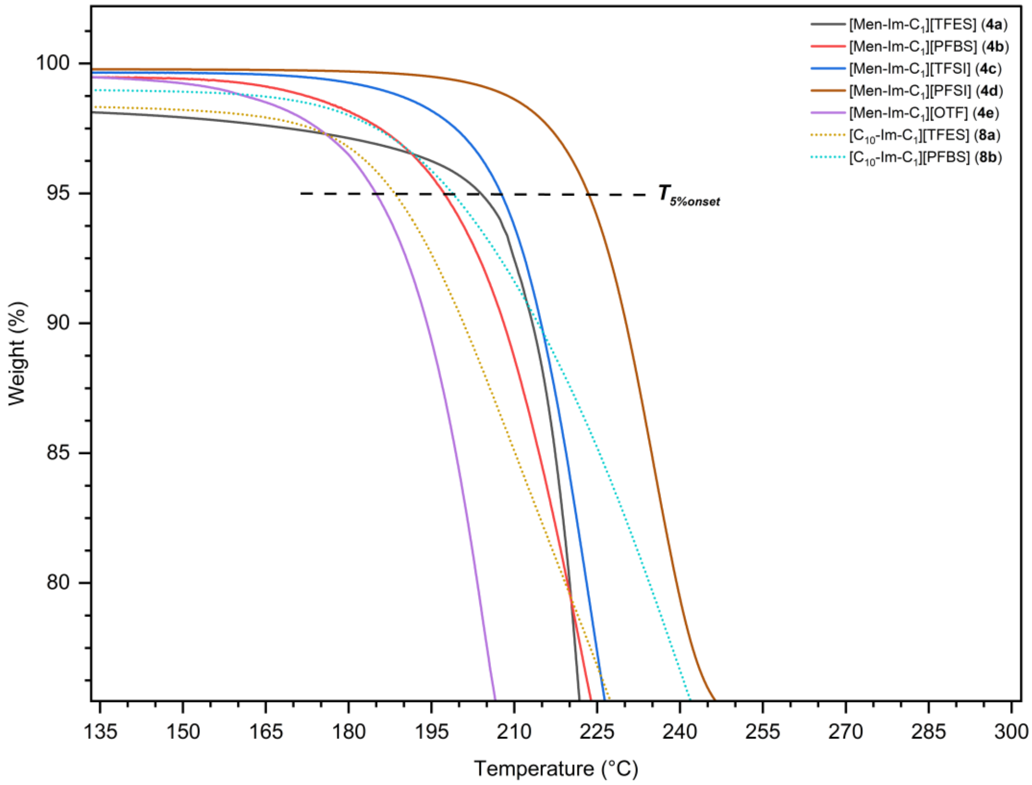

Figure S2. TGA thermograms of the synthesised FILs (**4a**–**4e**, **8a**, **8b**). Resolution of 5% mass loss.

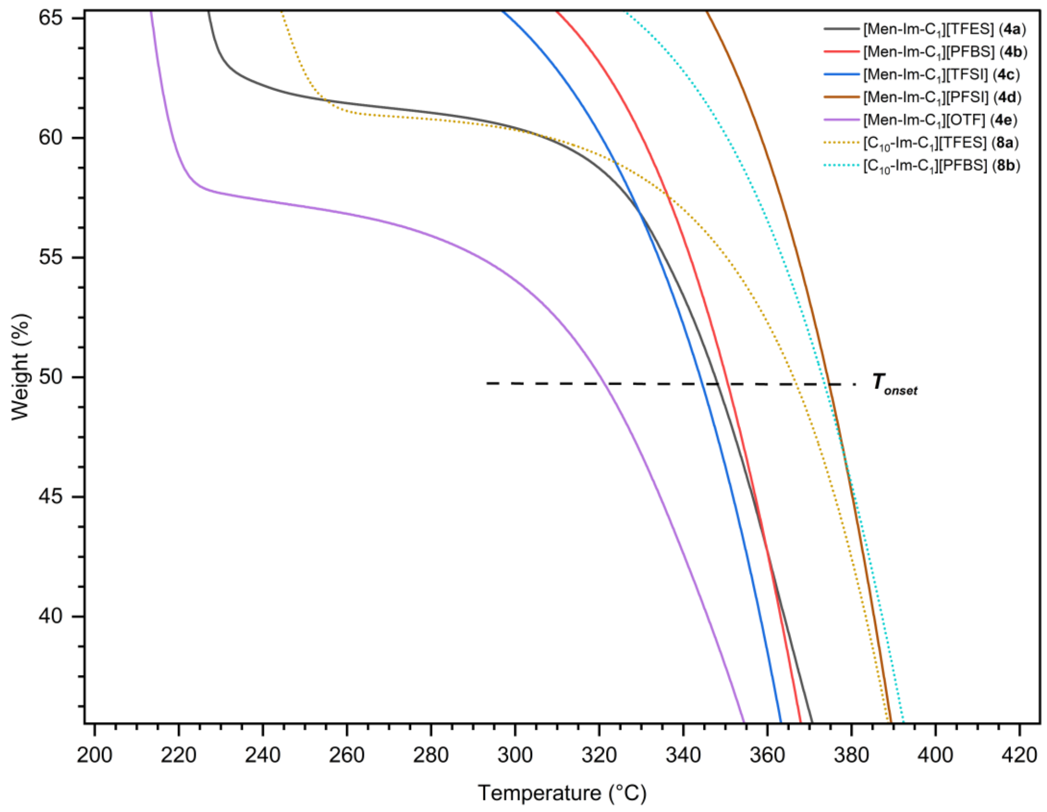

Figure S3. TGA thermograms of the synthesised FILs (**4a**–**4e**, **8a**, **8b**), Resolution of 50% mass loss.

DSC thermograms of FILs

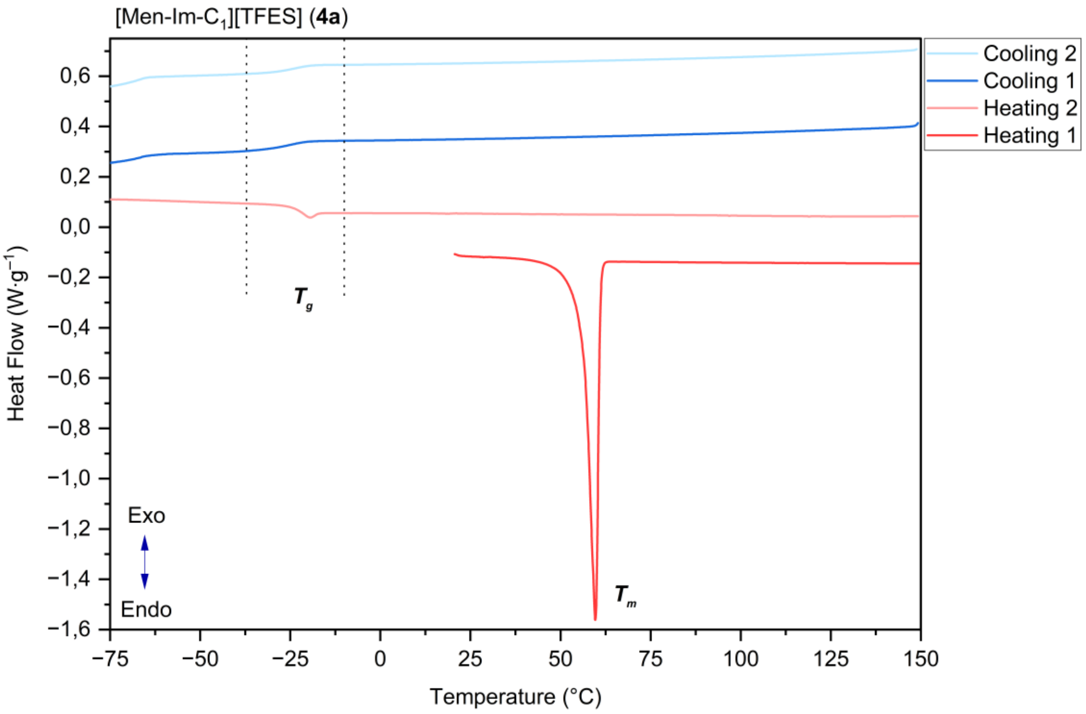

Figure S4. DSC thermogram of [Men-Im-C_1][TFES] (**4a**).

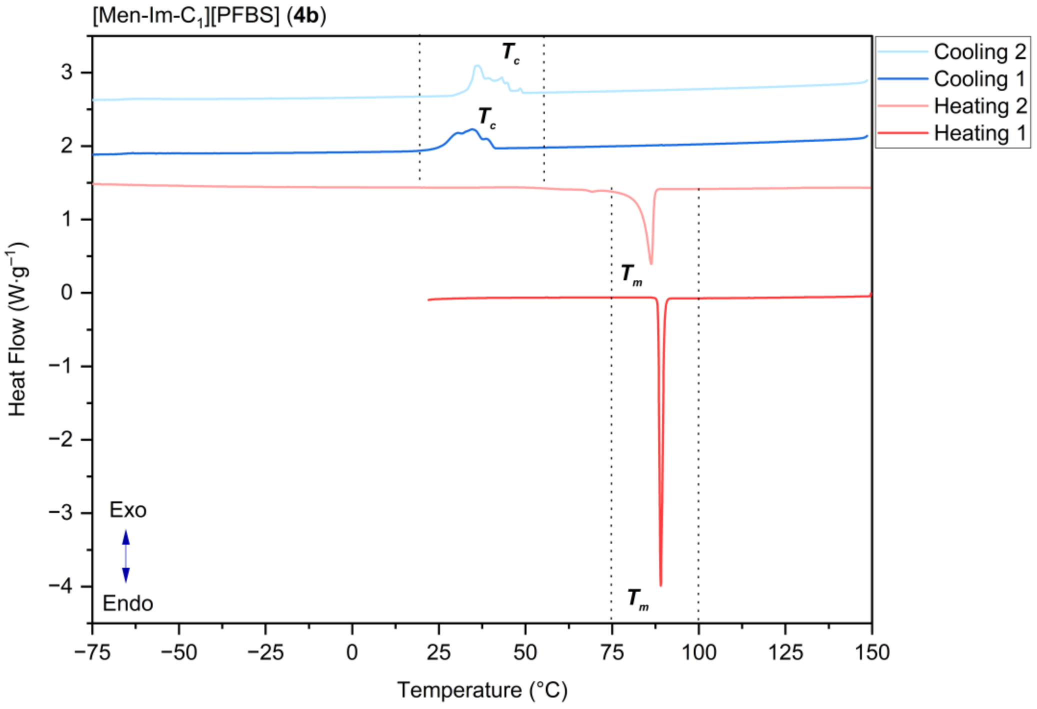

Figure S5. DSC thermogram of [Men-Im-C_1][PFBS] (**4b**).

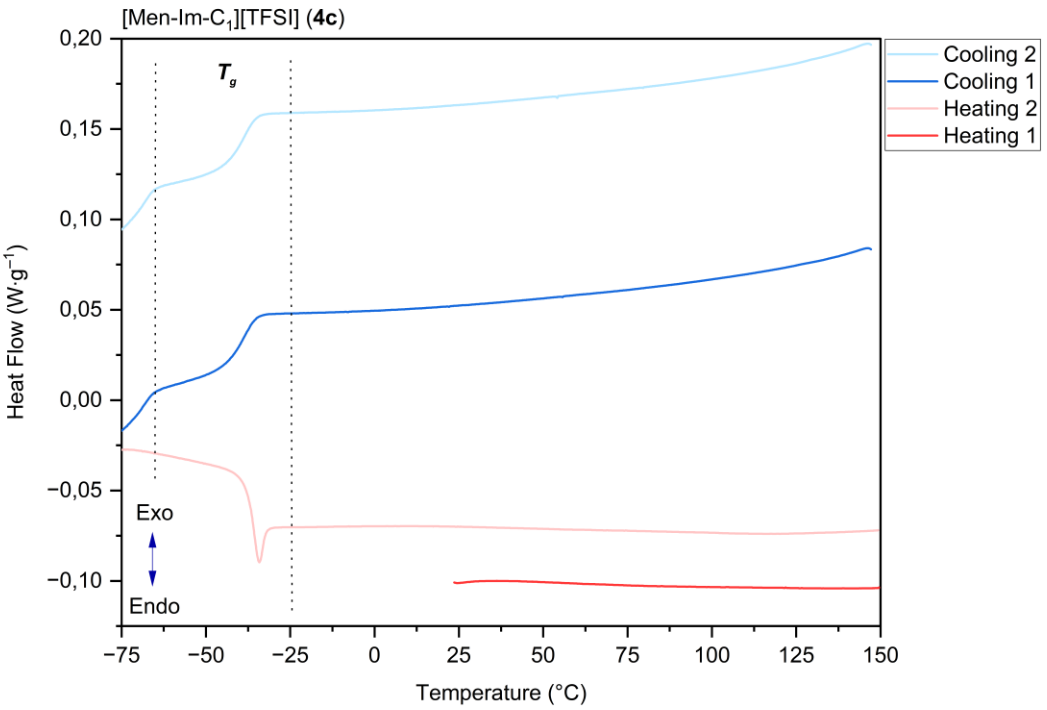

Figure S6. DSC thermogram of [Men-Im-C_1][TFSI] (**4c**).

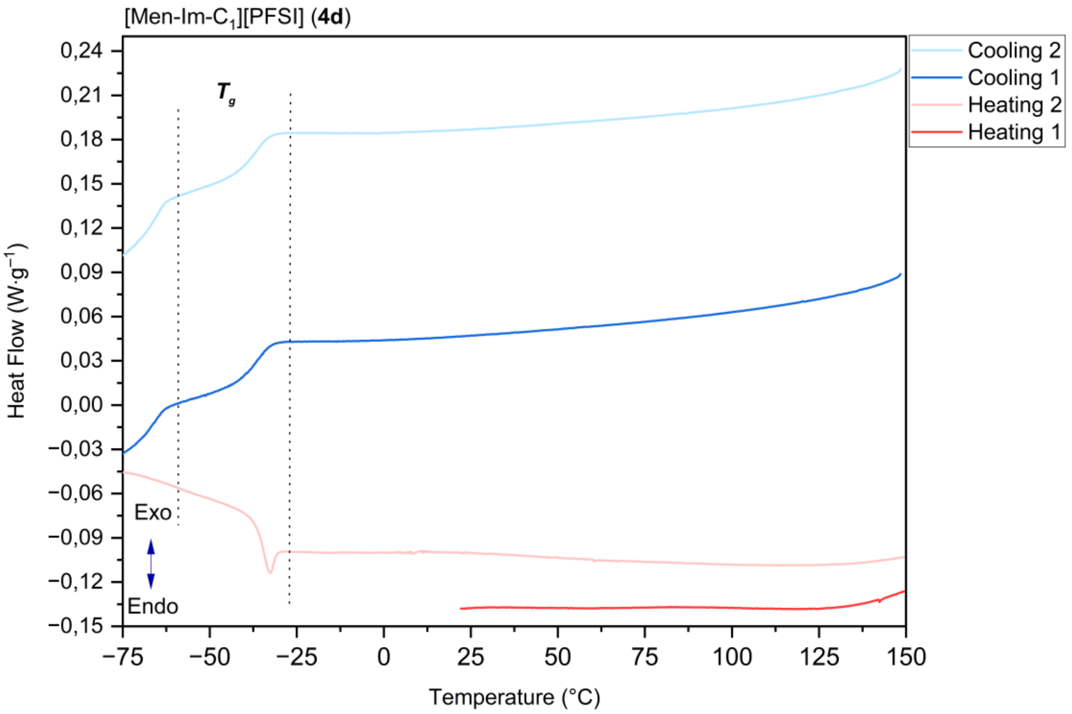

Figure S7. DSC thermogram of [Men-Im-C_1][PFSI] (**4d**).

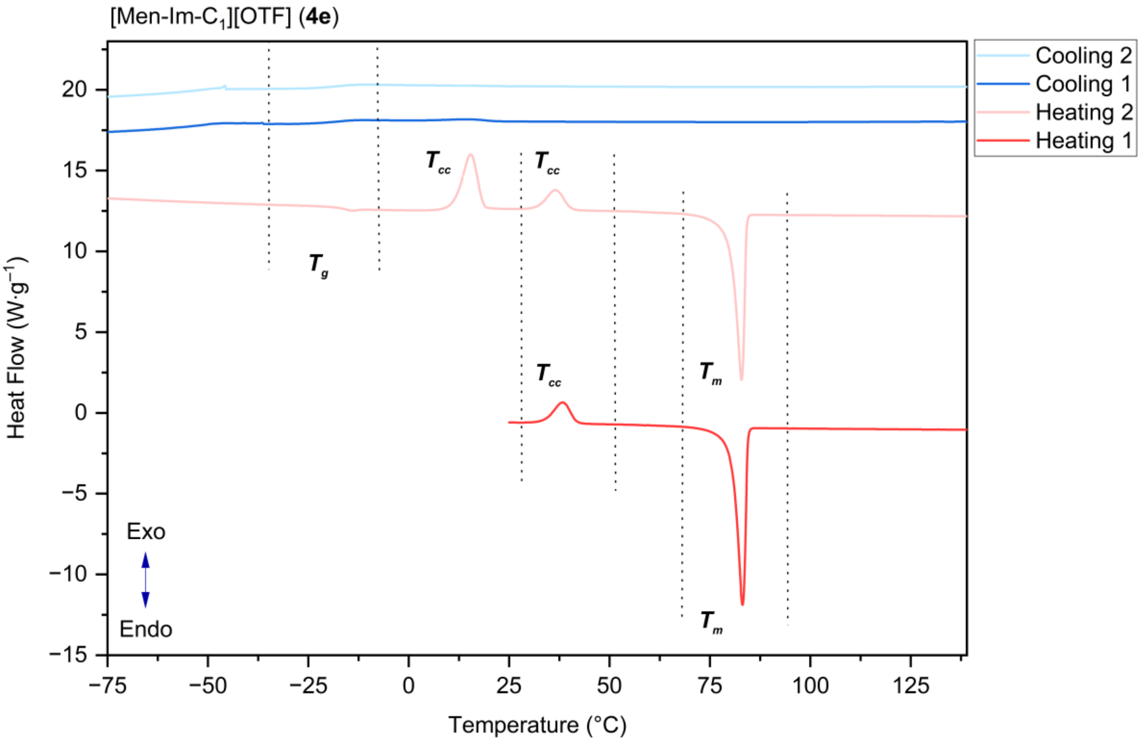

Figure S8. DSC thermogram of [Men-Im-C_1][OTF] (**4e**).

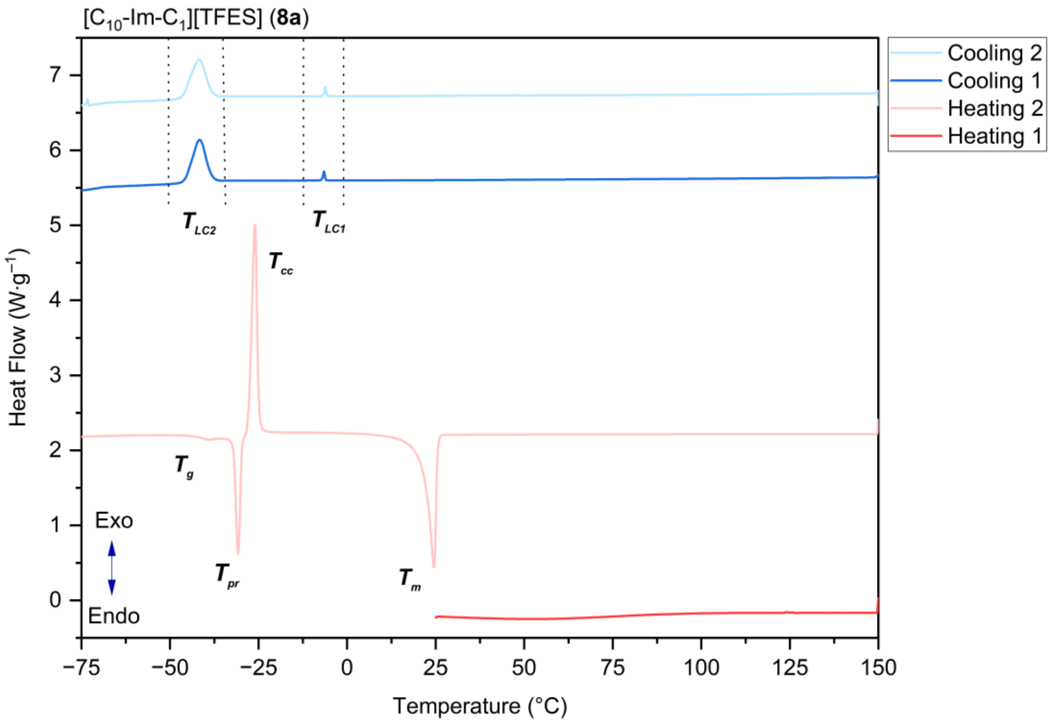

Figure S9. DSC thermogram of [C_{10}-Im-C_1][TFES] (**8a**).

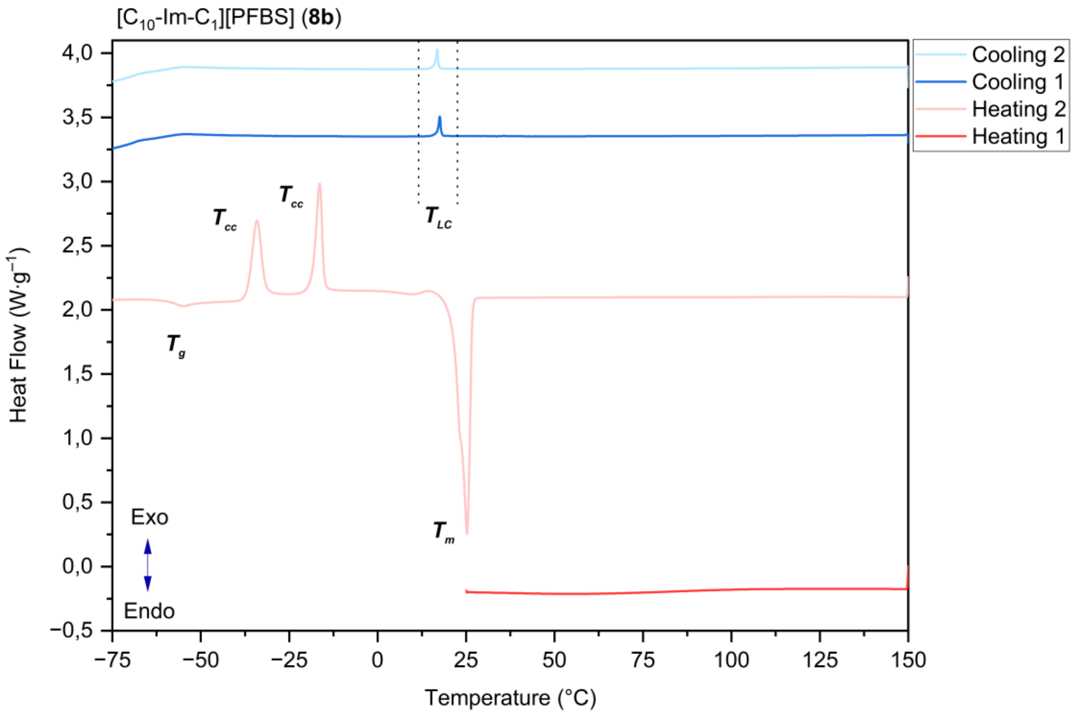

Figure S10. DSC thermogram of [C_{10}-Im-C_1][PFBS] (**8b**).

Refractive index chart of FILs in liquid state depending on temperature

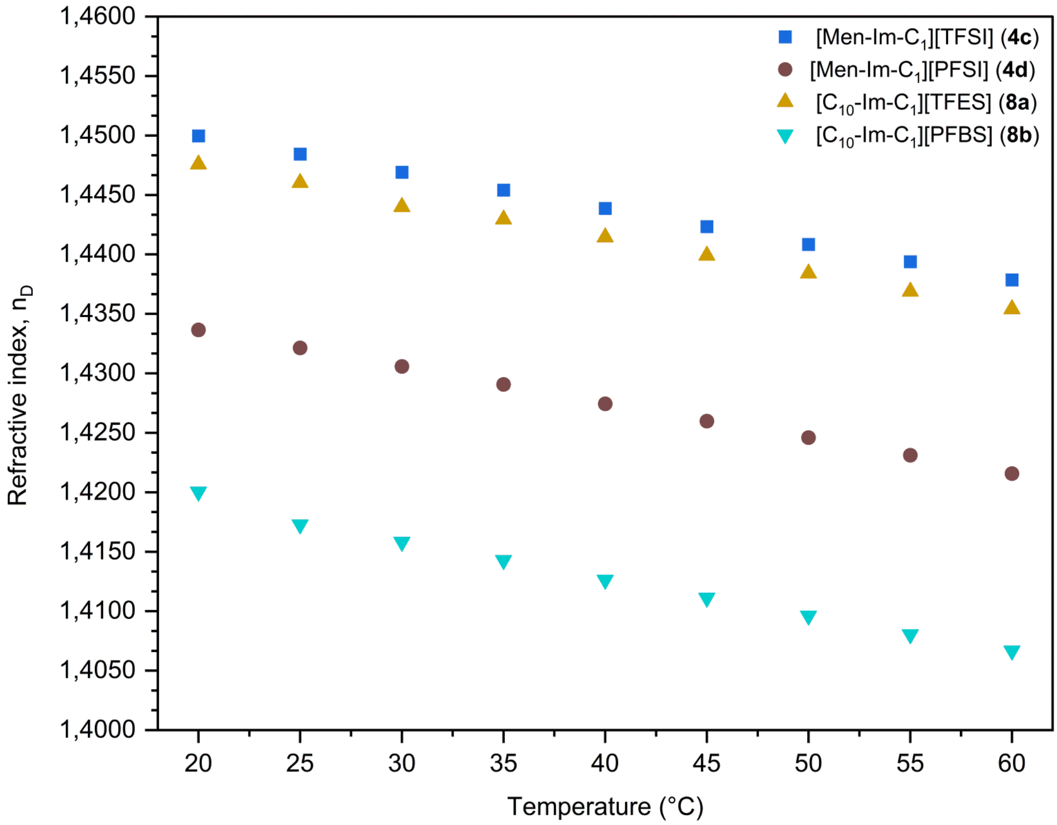

Figure S11. Refractive index of FILs in liquid state (**4c**, **4d**, **8a**, **8b**) as a function of temperature.

FTIR spectra of 1-[(1*R*,2*S*,5*R*)-(–)-menthoxymethyl]-3-methylimidazolium [Men-Im-C_1][X] salts (3, 4a–4e) and 1-decyloxymethyl-3-methylimidazolium [C_{10}-Im-C_1][X] (7, 8a, 8b) salts

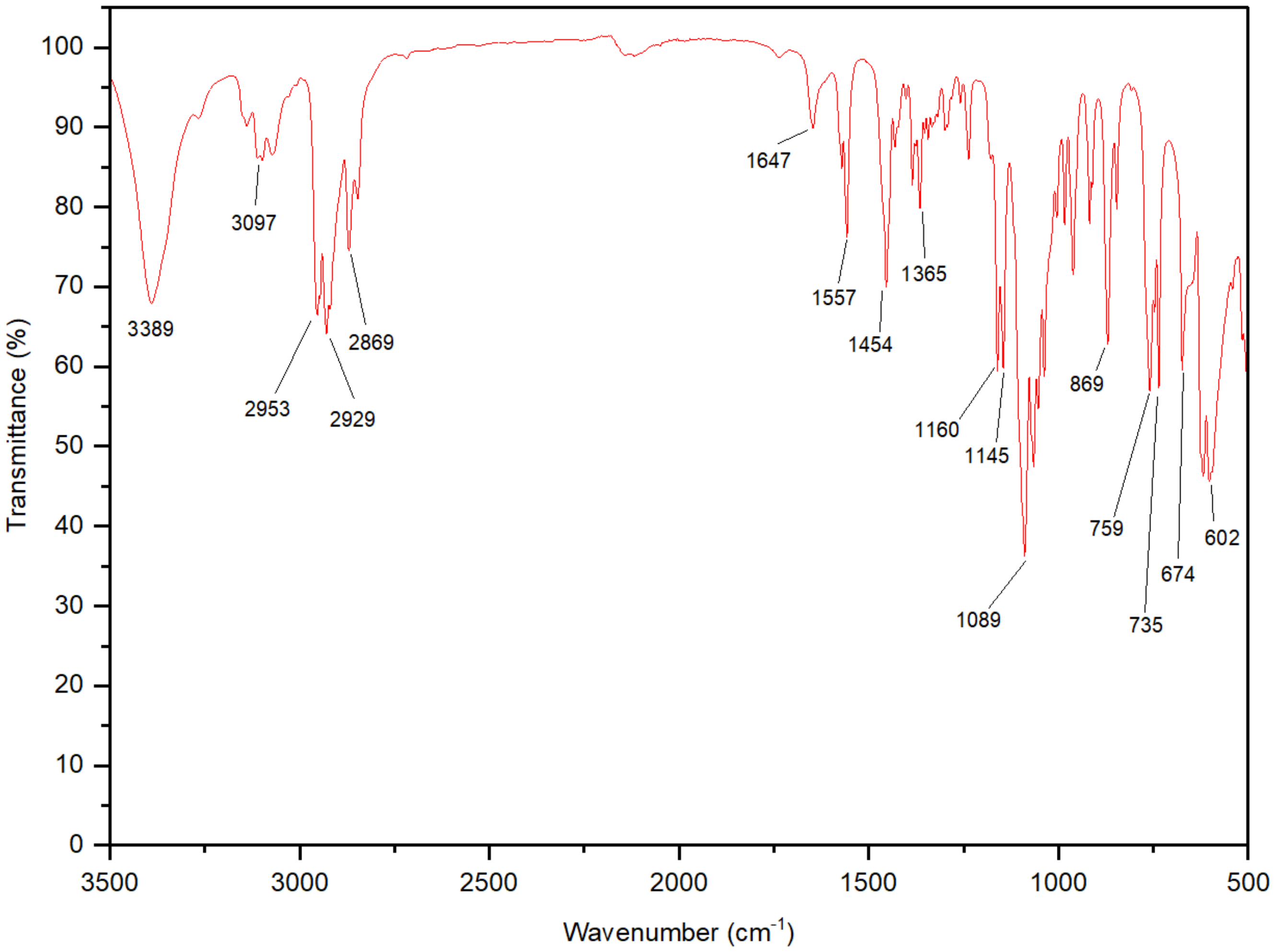

Figure S12. FTIR spectrum of [Men-Im-C_1][Cl] (**3**).

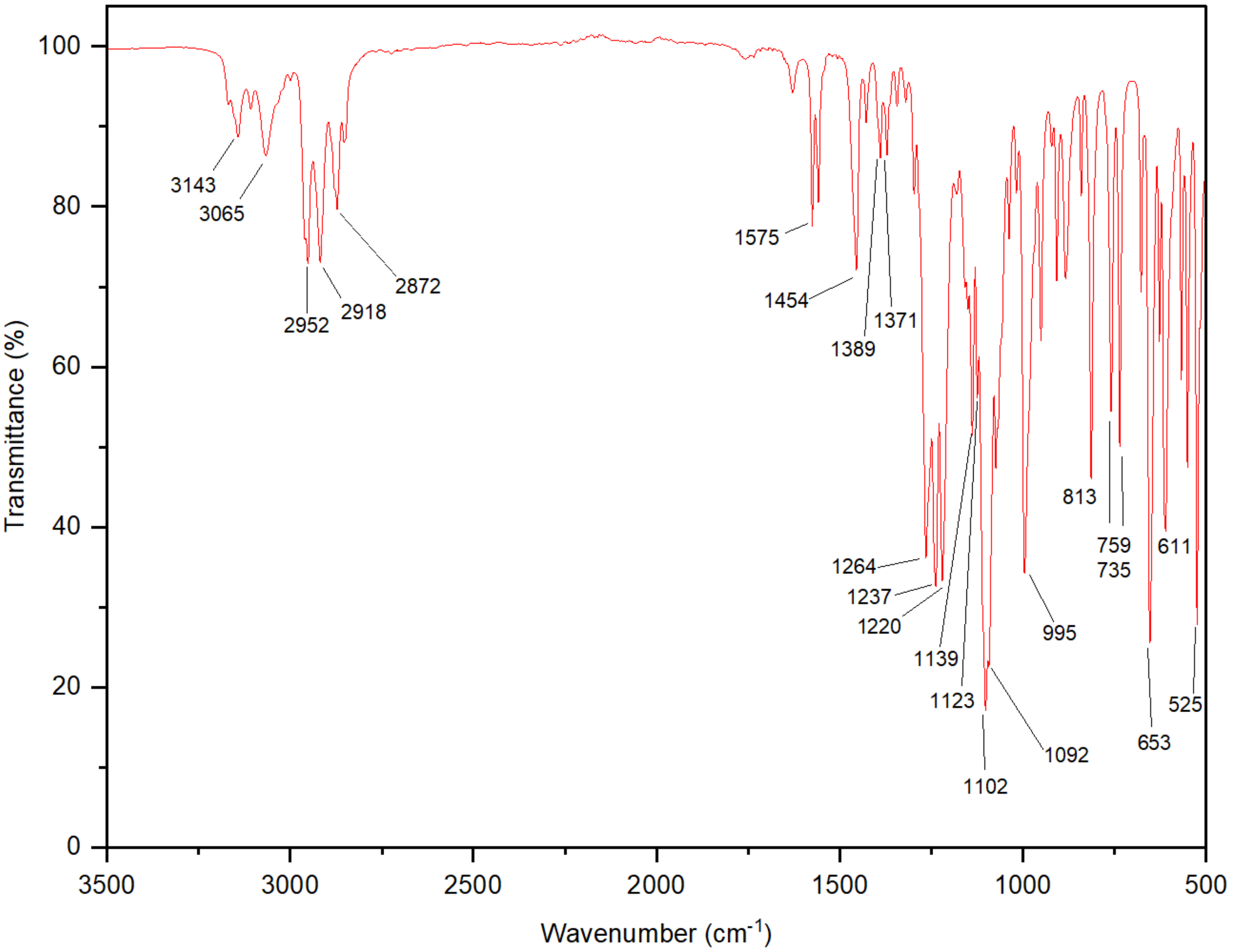

Figure S13. FTIR spectrum of [Men-Im-C_1][TFES] (**4a**).

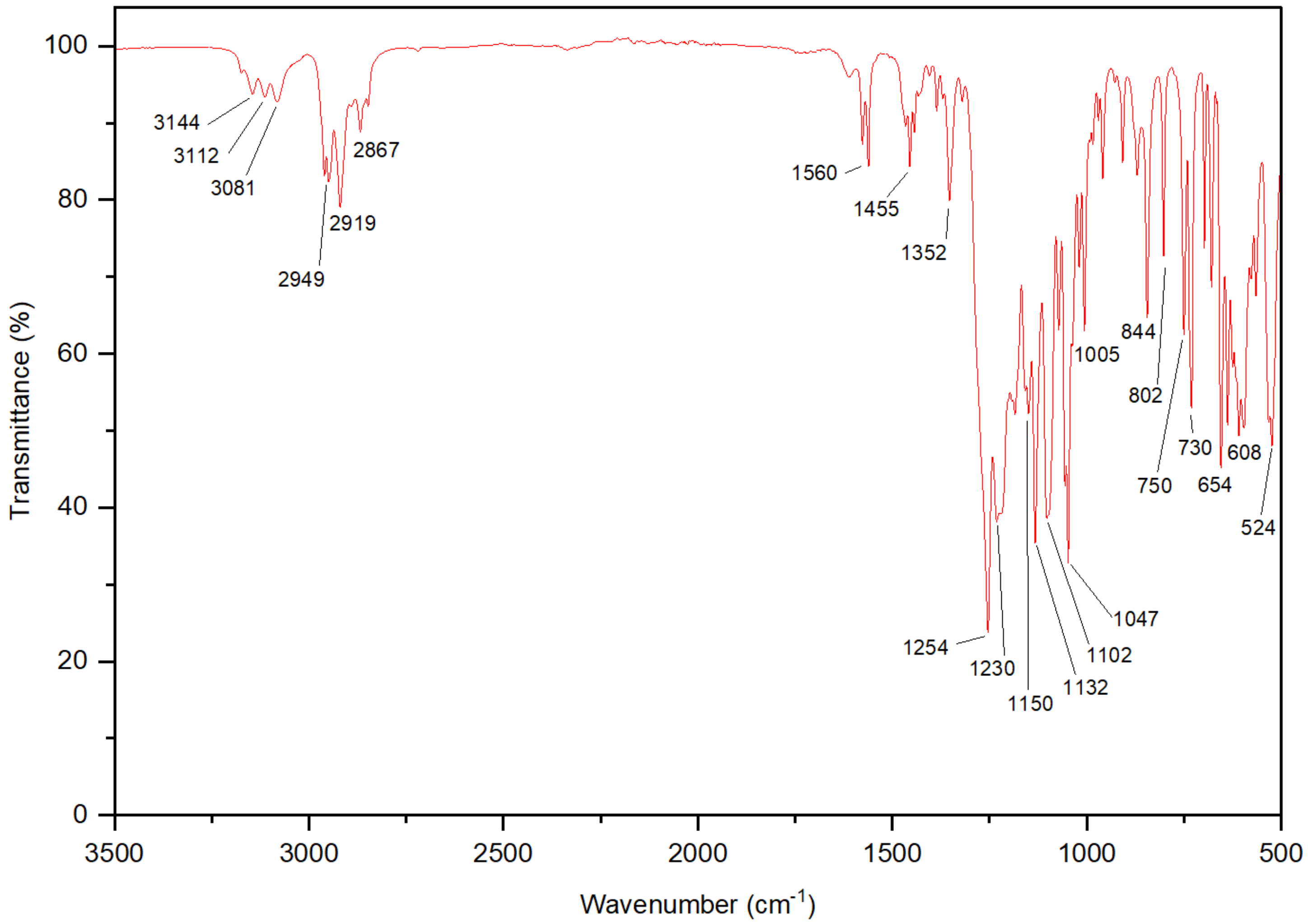

Figure S14. FTIR spectrum of [Men-Im-C_1][PFBS] (**4b**).

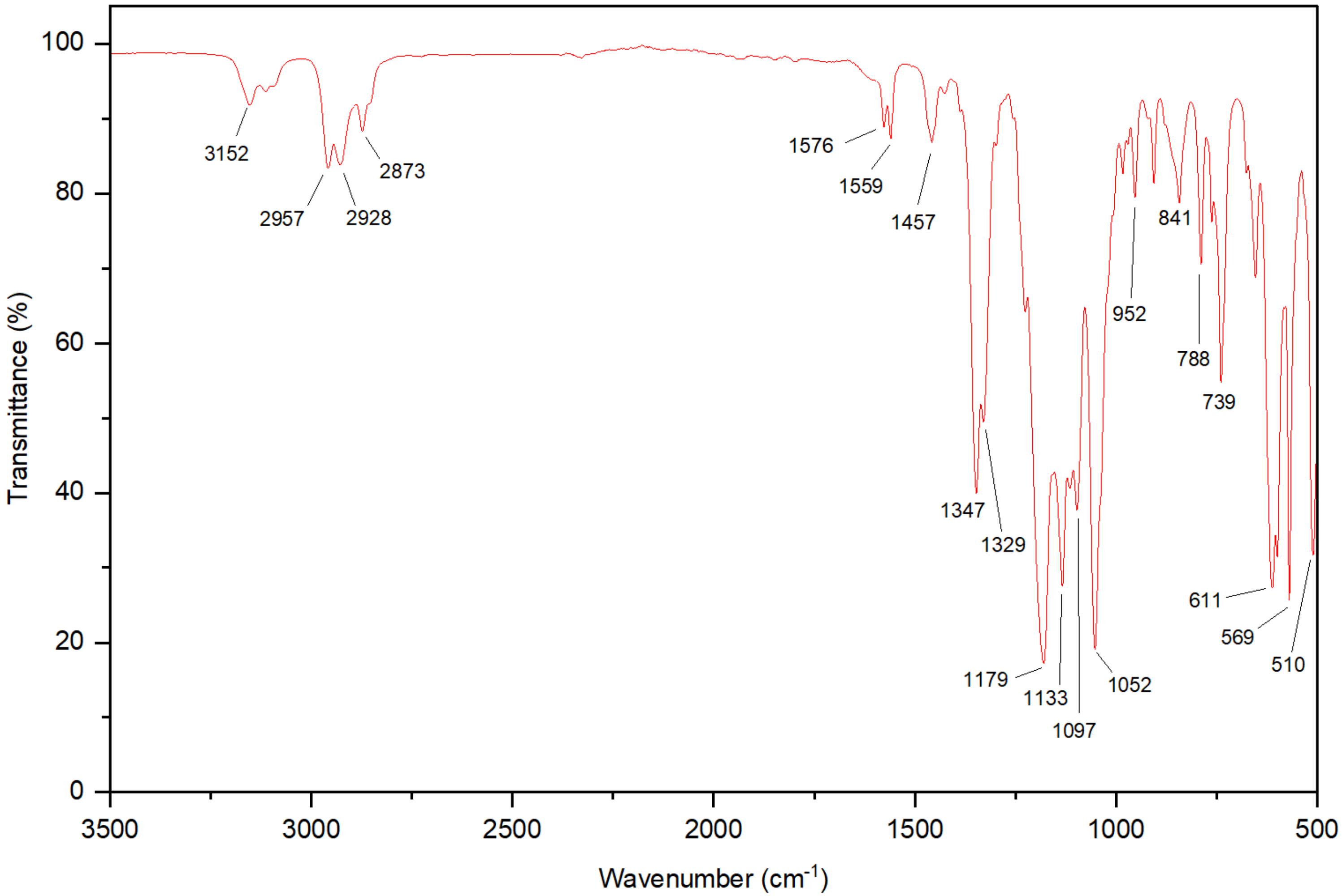

Figure S15. FTIR spectrum of [Men-Im-C_1][TFSI] (**4c**).

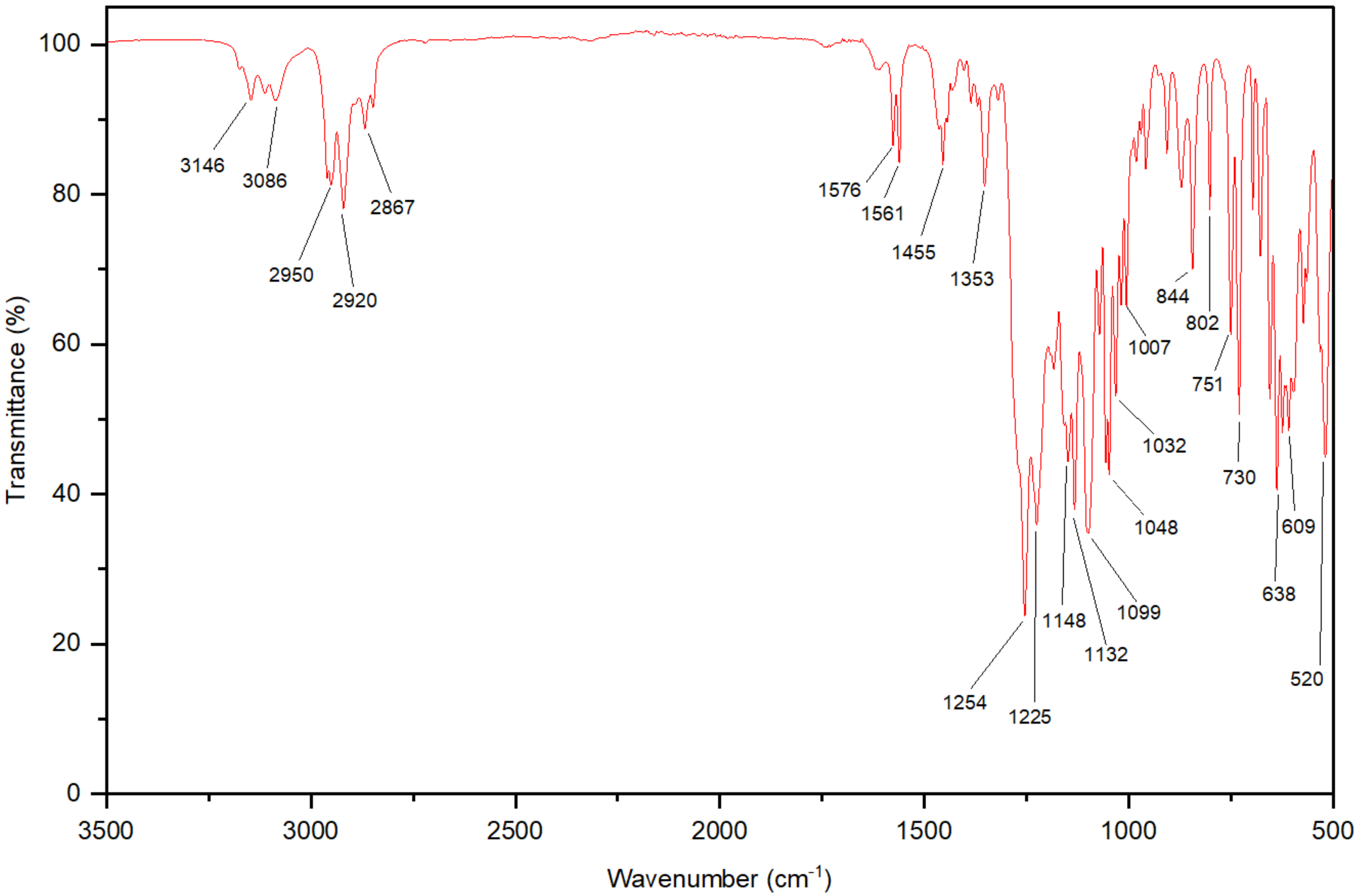

Figure S16. FTIR spectrum of [Men-Im-C_1][PFSI] (**4d**).

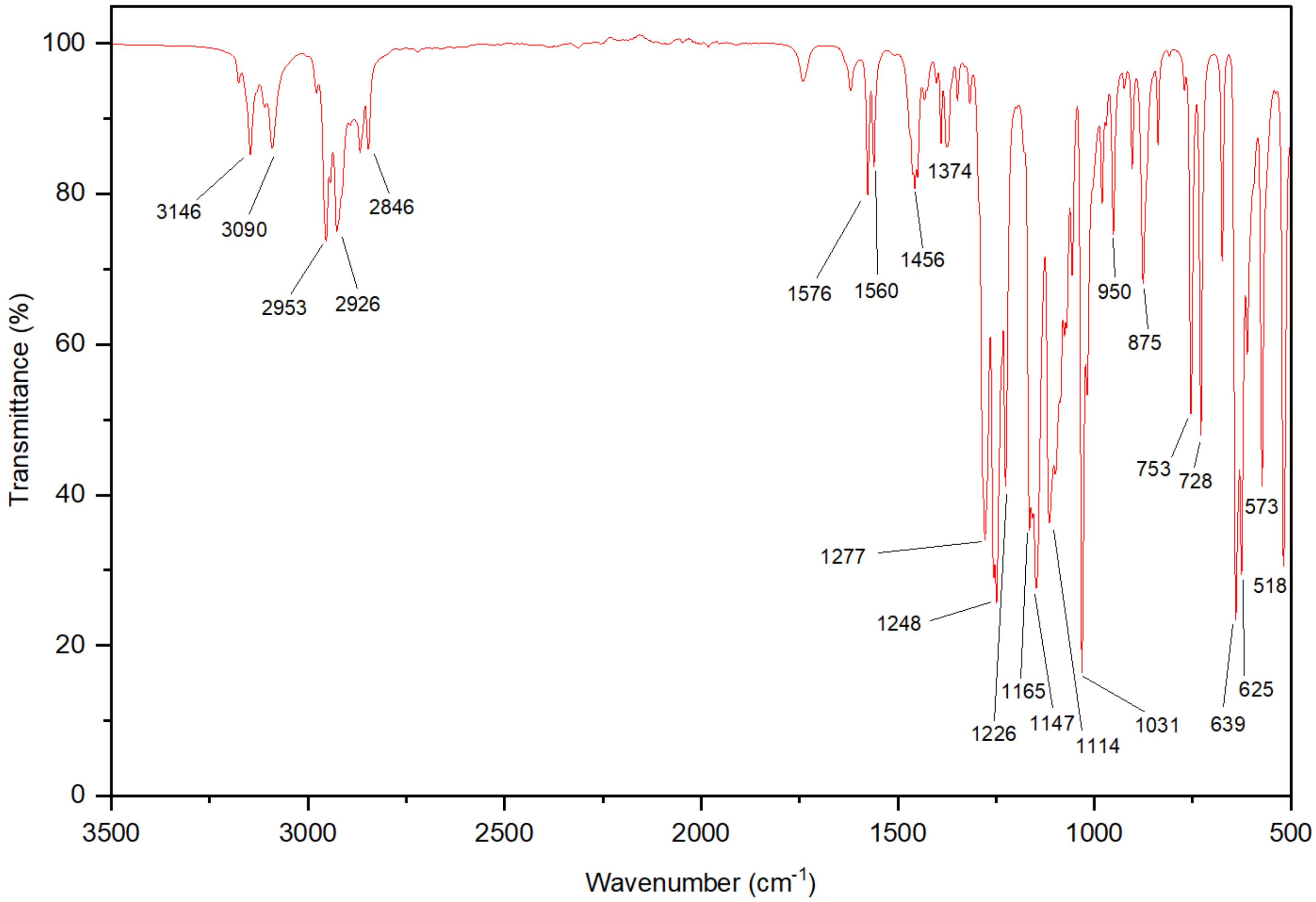

Figure S17. FTIR spectrum of [Men-Im-C_1][OTF] (**4e**).

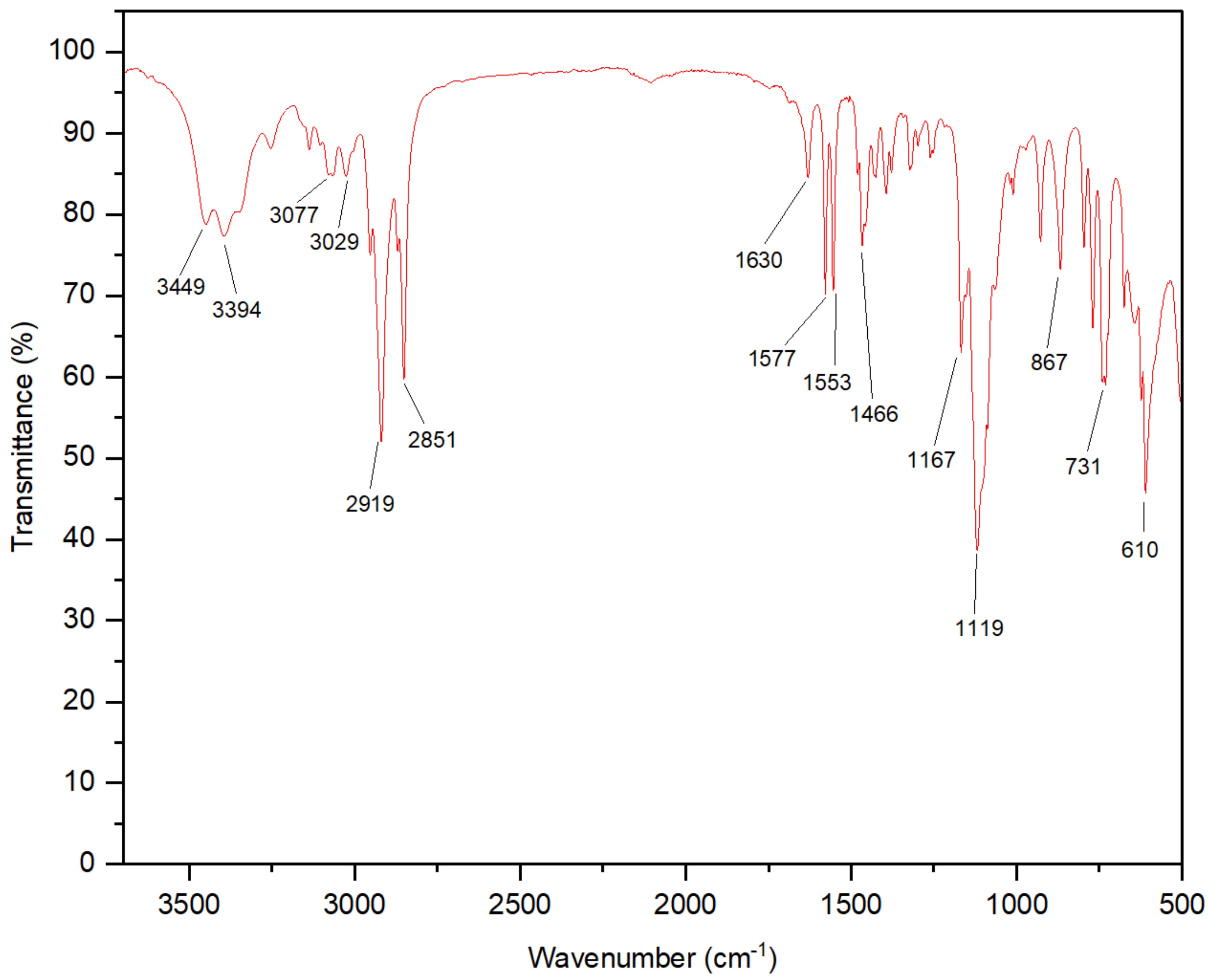

Figure S18. FTIR spectrum of [C_{10}-Im-C_1][Cl] (**7**).

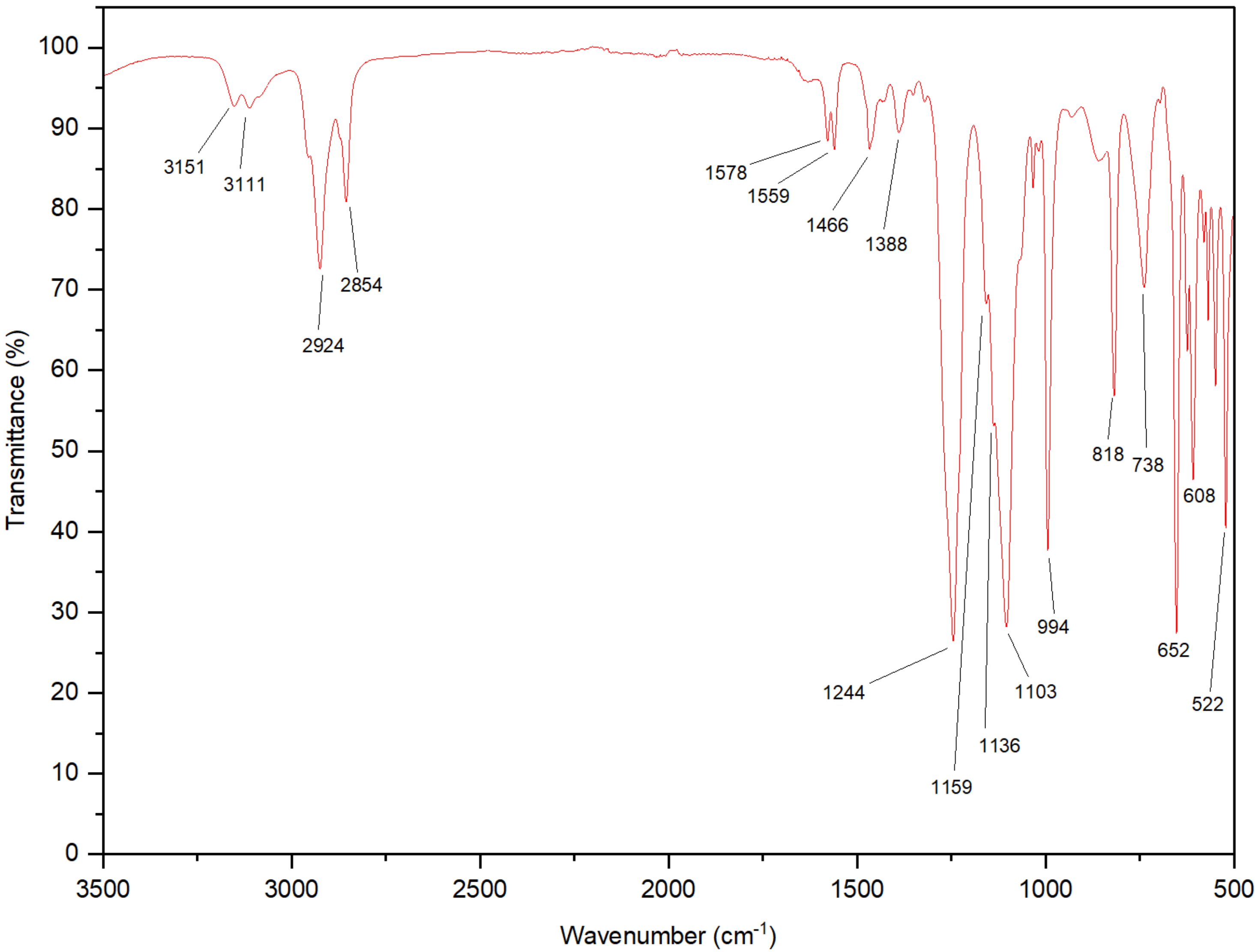

Figure S19. FTIR spectrum of [C_{10}-Im-C_1][TFES] (**8a**).

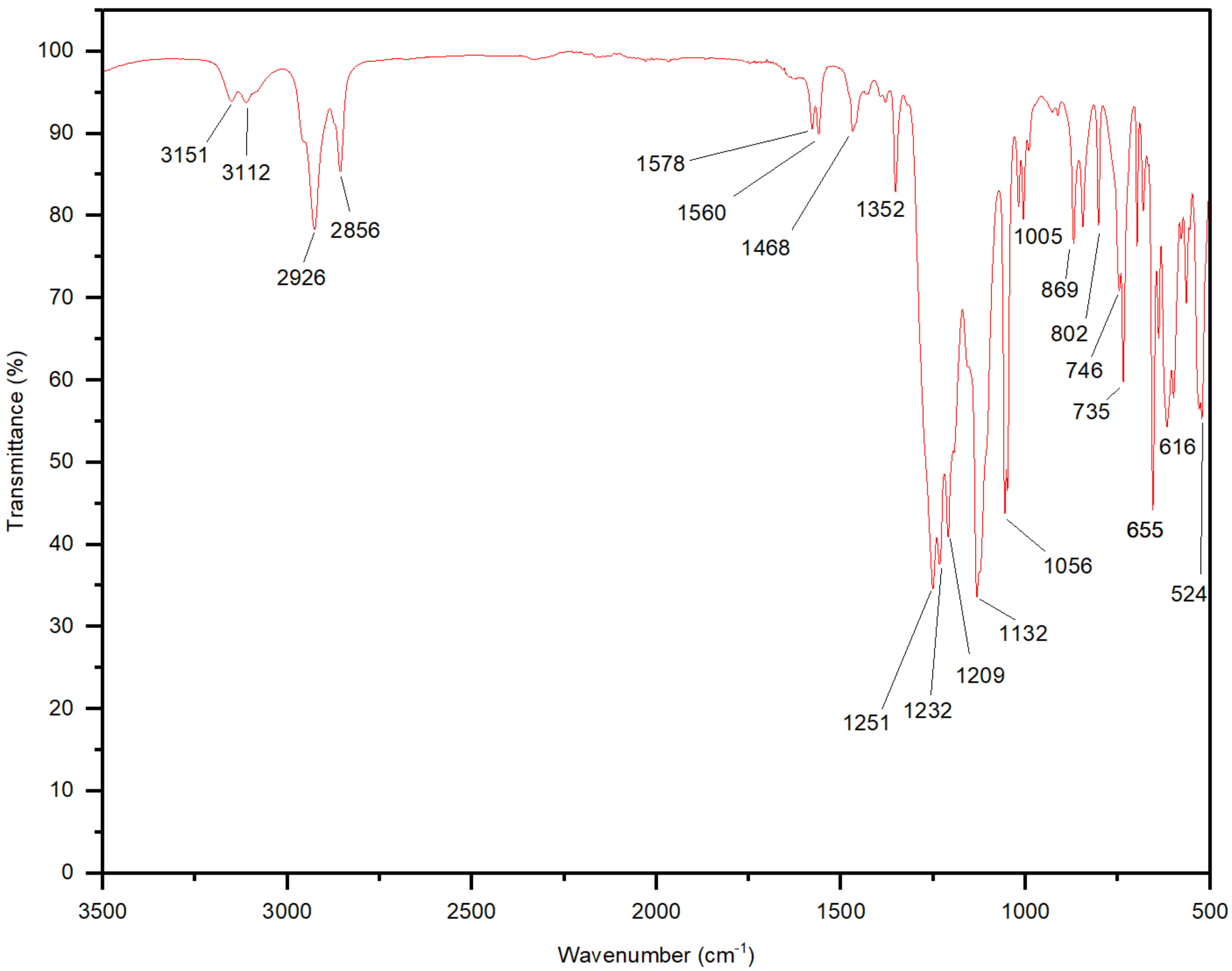

Figure S20. FTIR spectrum of [C_{10}-Im-C_1][PFBS] (**8b**).

NMR spectra of discussed 1-[(1*R*,2*S*,5*R*)-(–)-menthoxymethyl]-3-methylimidazolium [Men-Im-C_1][X] salts (3, 4a–4e) and 1-decyloxymethyl-3-methylimidazolium [C_{10}-Im-C_1][X] (7, 8a, 8b) salts in $CDCl_3$

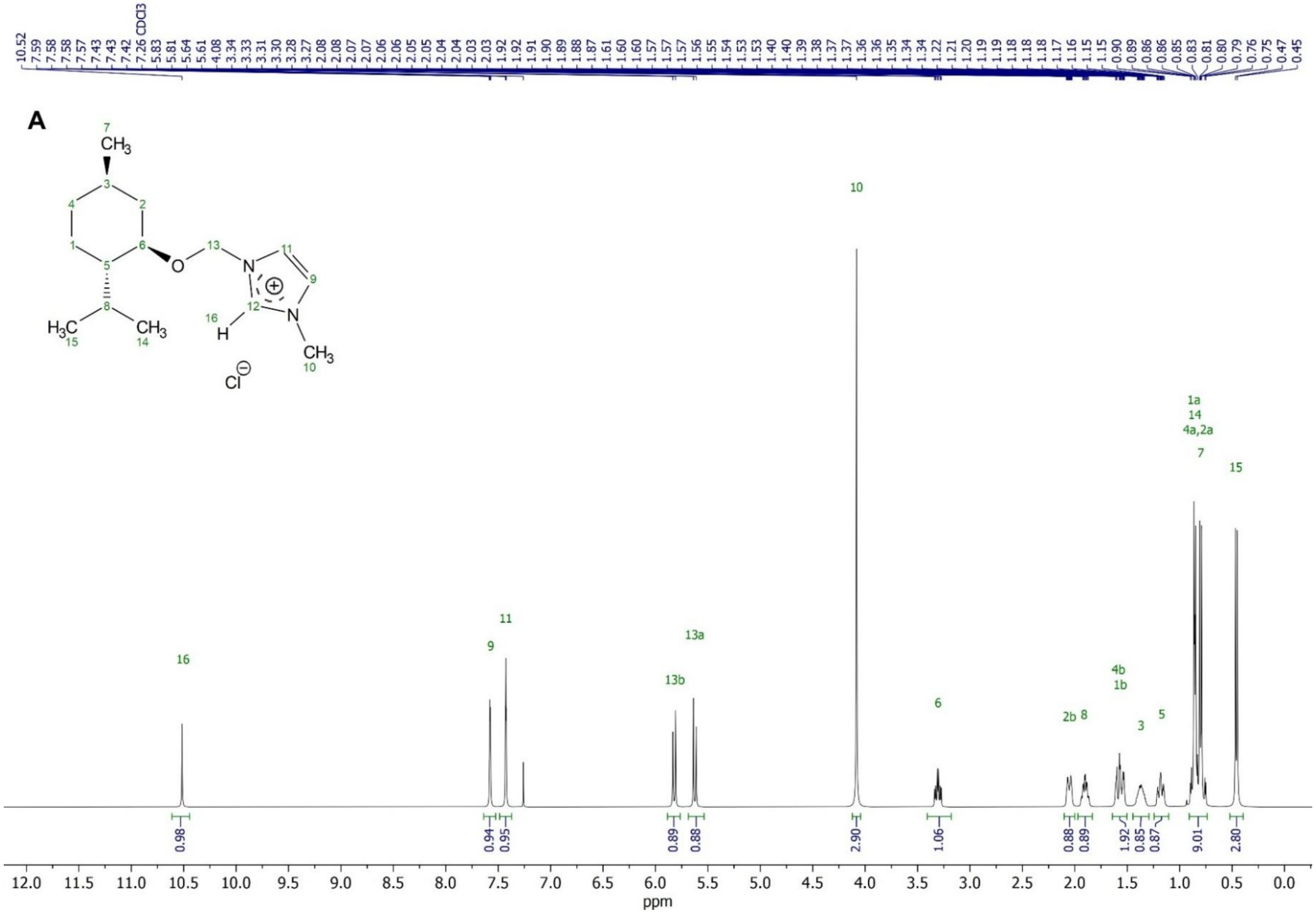

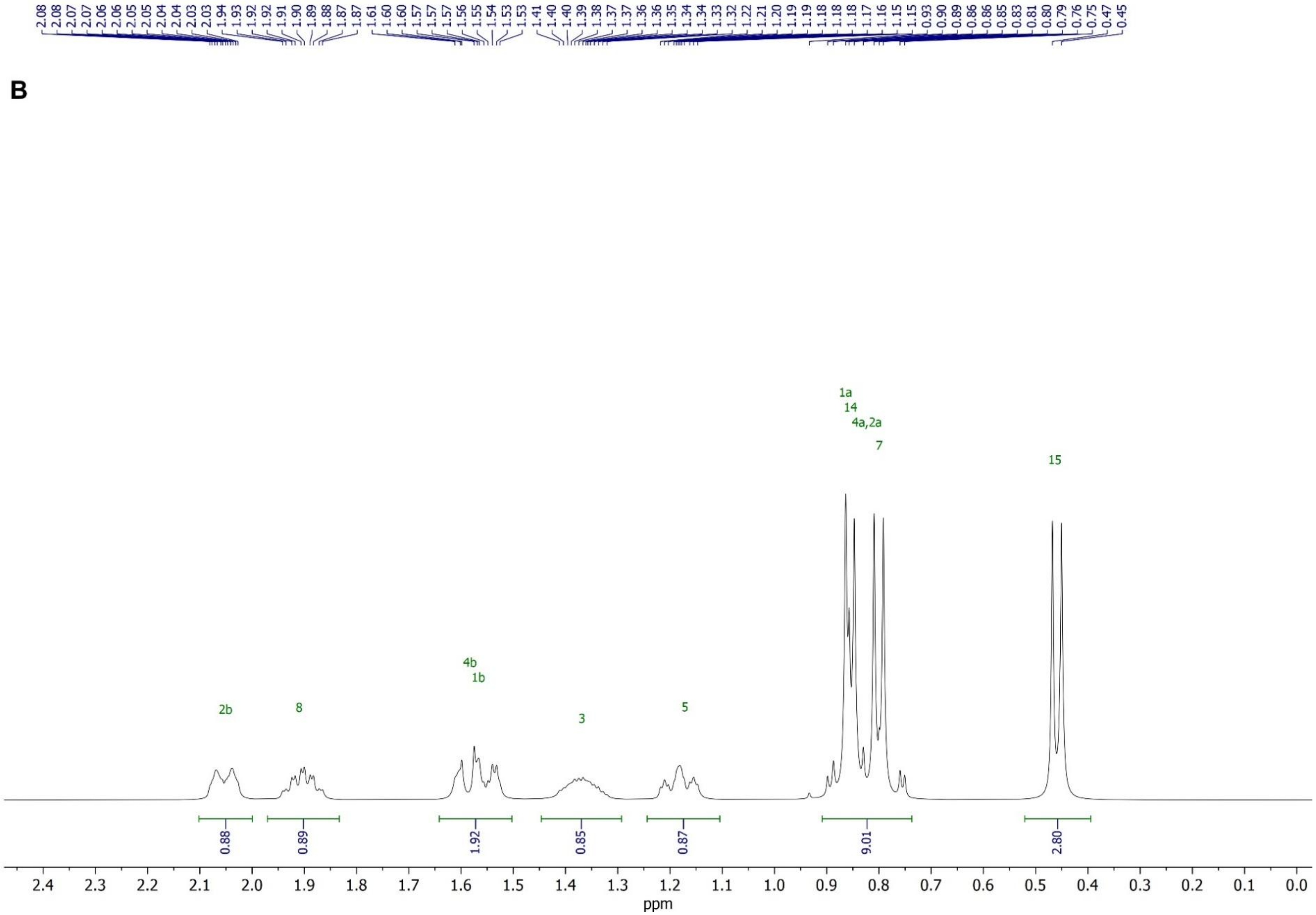

Figure S21. ^{1}H NMR (400 MHz) spectra of [Men-Im-C_1][Cl] (**3**) in $CDCl_3$. **A.** region from 0.0 ppm to 12.0 ppm. **B.** region from 0.0 ppm to 2.4 ppm.

A

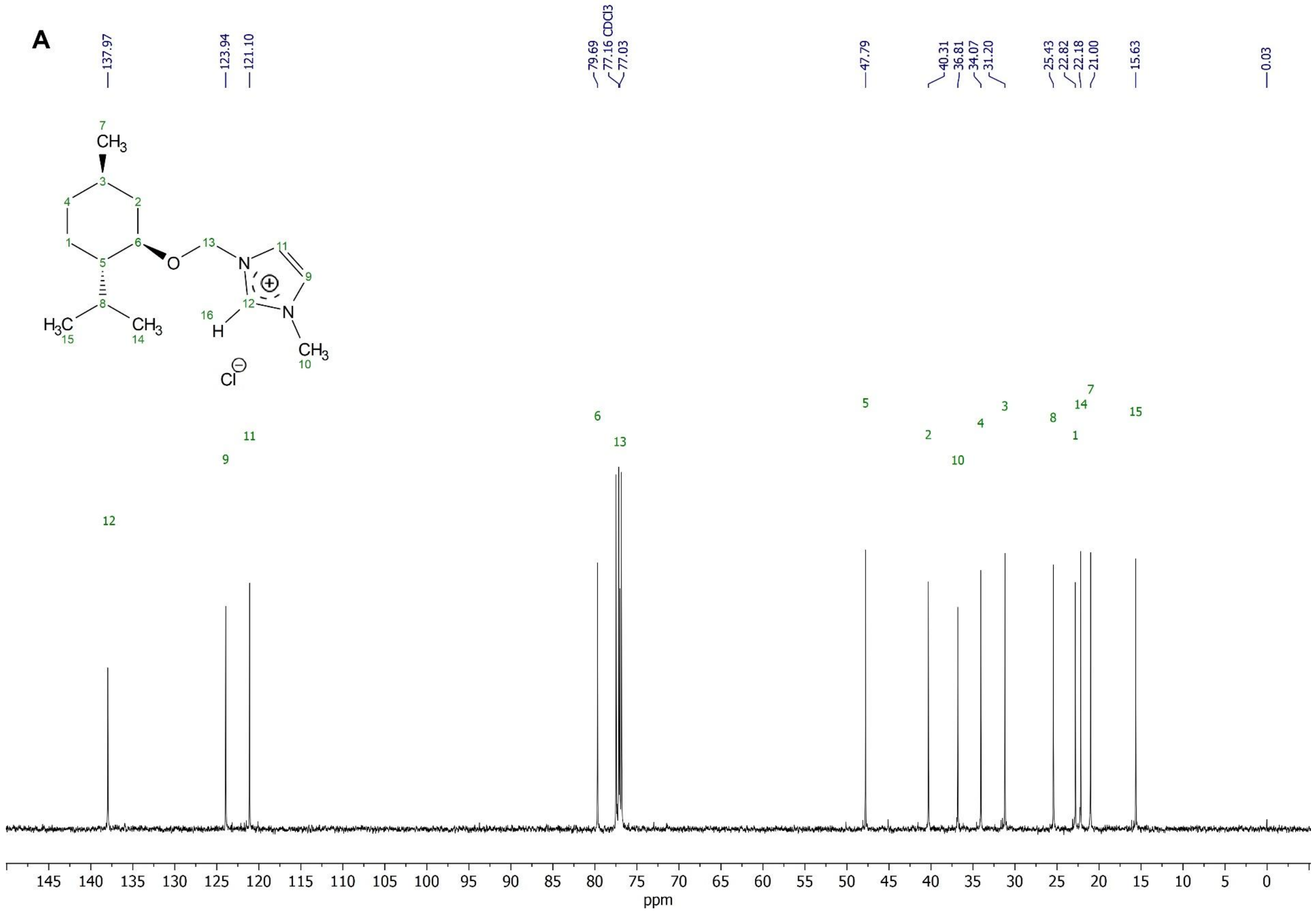
137.97
123.94
121.10
79.69
77.16 CDCl3
77.03
47.79
40.31
36.81
34.07
31.20
25.43
22.82
22.18
21.00
15.63
0.03
ppm

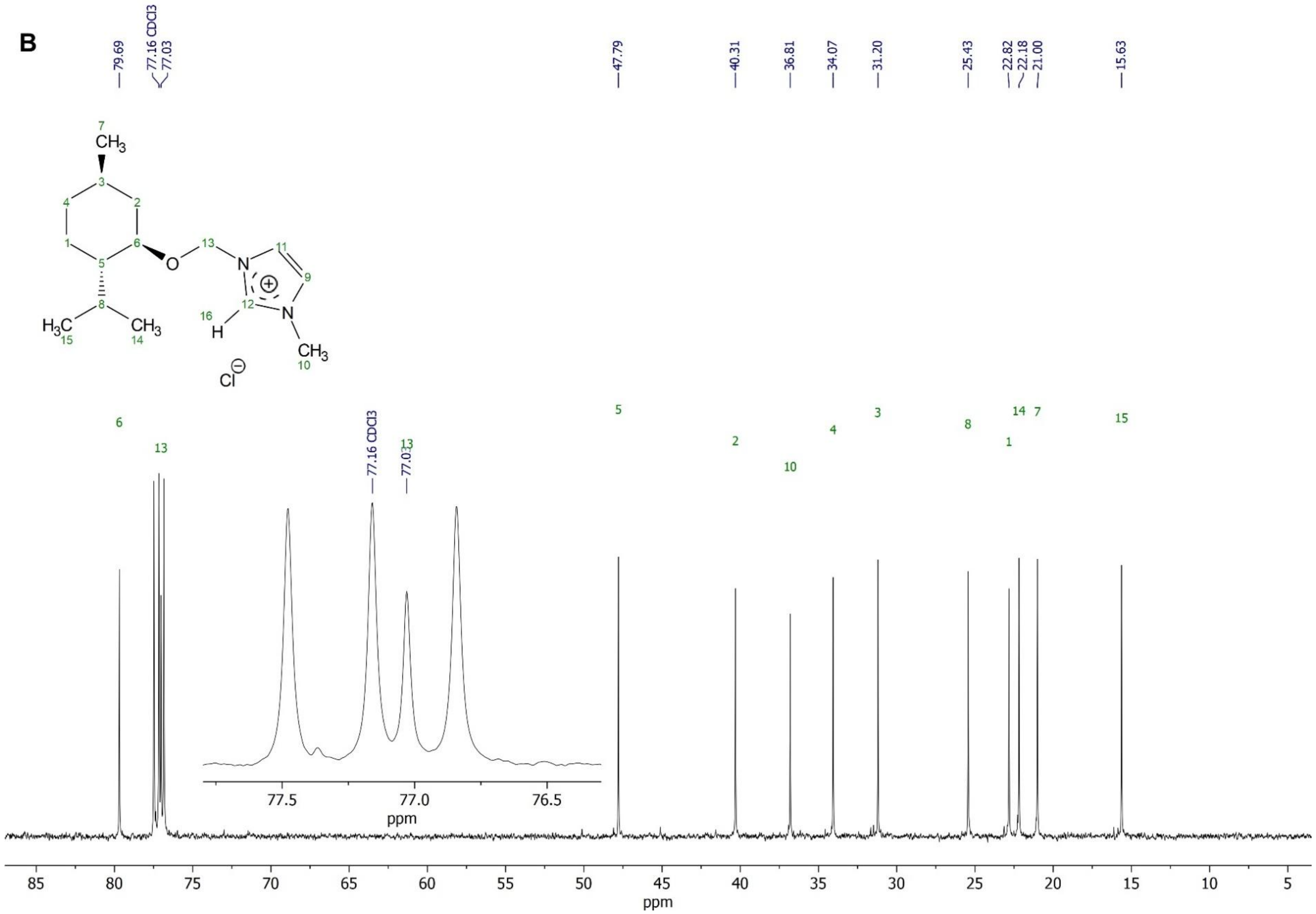

Figure S22. ^{13}C NMR (100MHz) spectra of [Men-Im-C_1][Cl] (**3**) in $CDCl_3$. **A.** region from 0.0 ppm to 145.0 ppm. **B.** region from 5.0 ppm to 85.0 ppm.

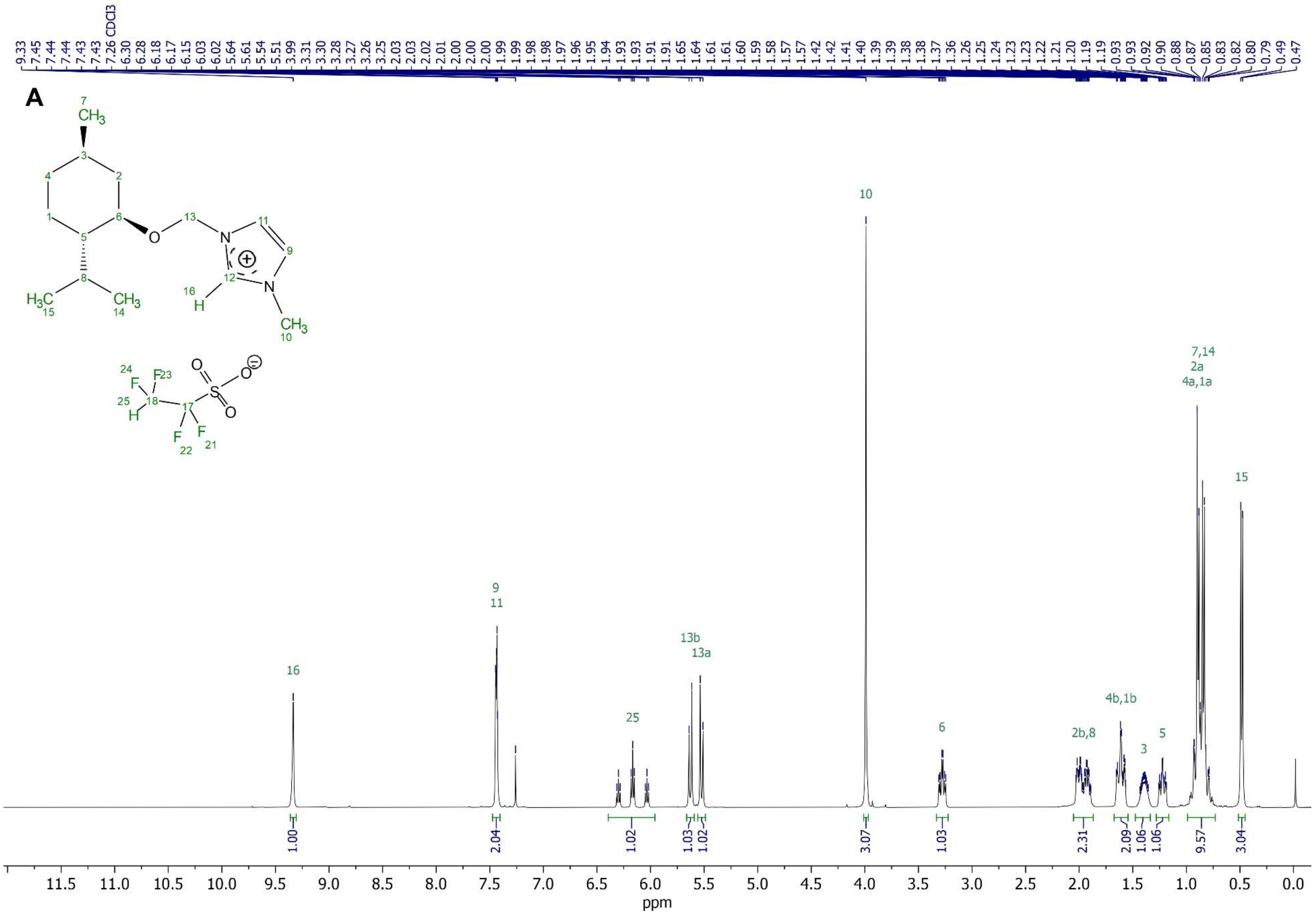
A
7,14
2a
4a,1a
16
9
11
25
13b
13a
10
6
2b,8
4b,1b
3
5
15
ppm

B

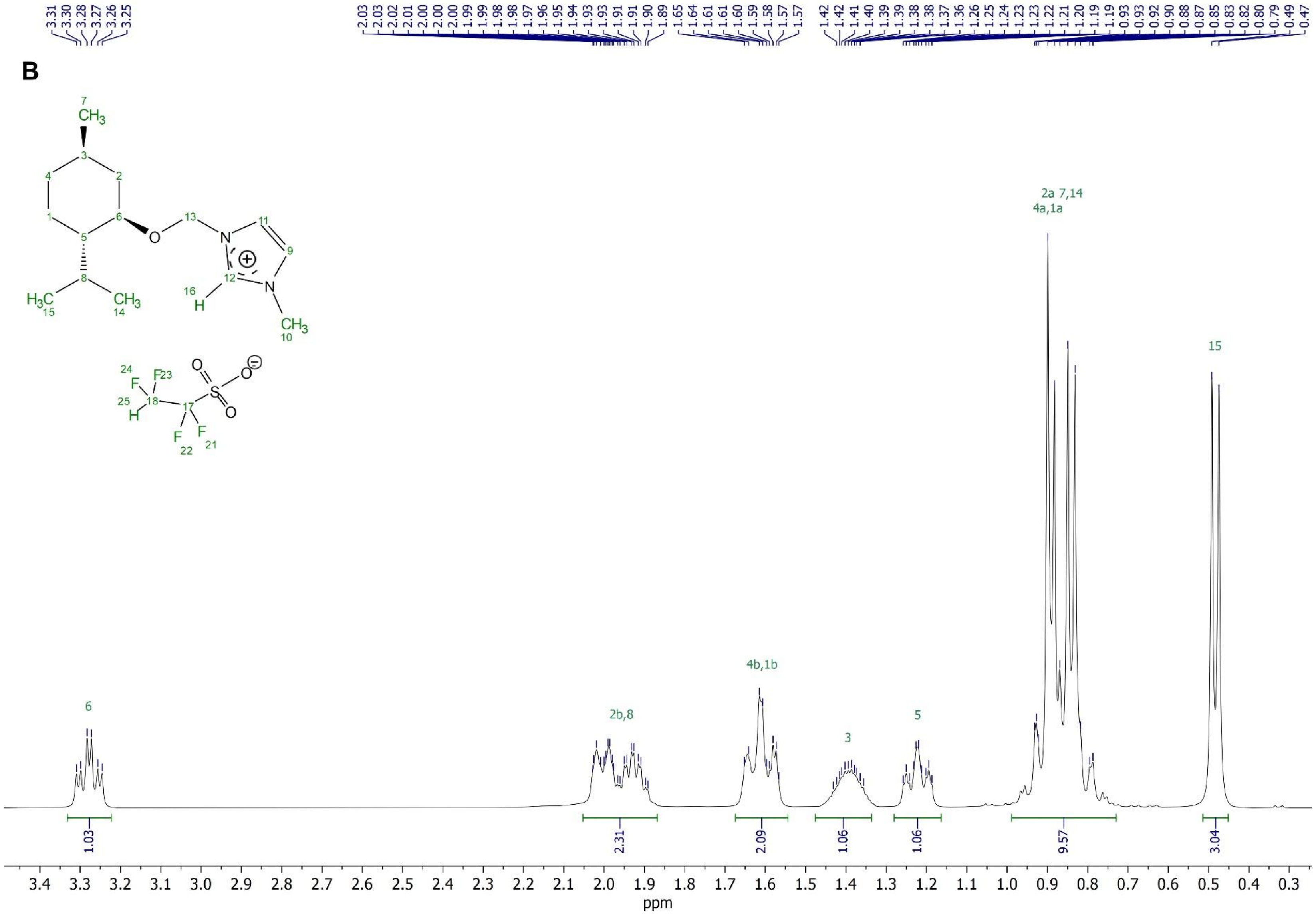

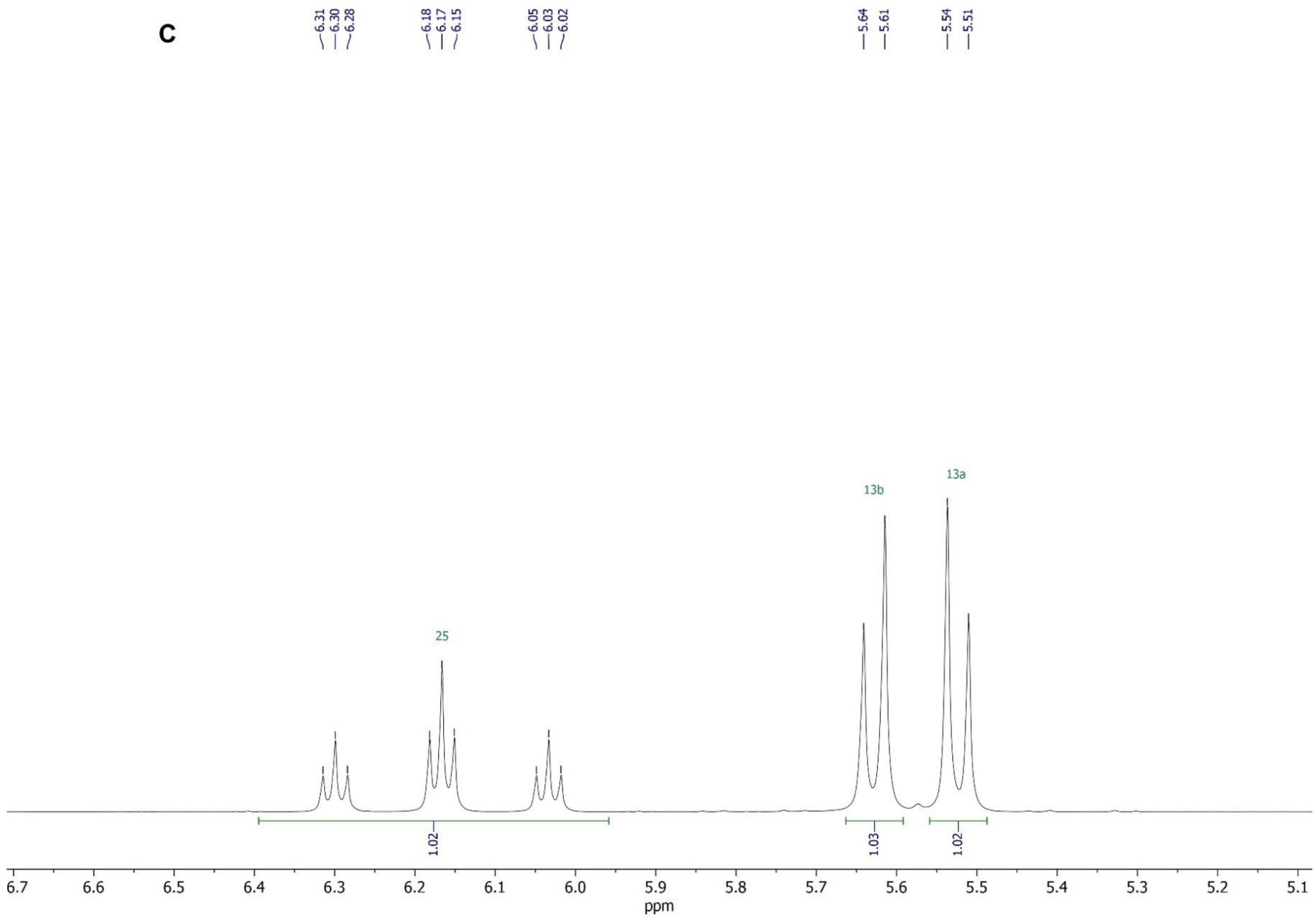

Figure S23. ^{1}H NMR (400 MHz) spectra of [Men-Im-C_1][TFES] (**4a**) in $CDCl_3$. **A.** region from 0.0 ppm to 11.5 ppm. **B.** region from 0.3 ppm to 3.4 ppm. **C.** region from 5.1 ppm to 6.7 ppm.

A

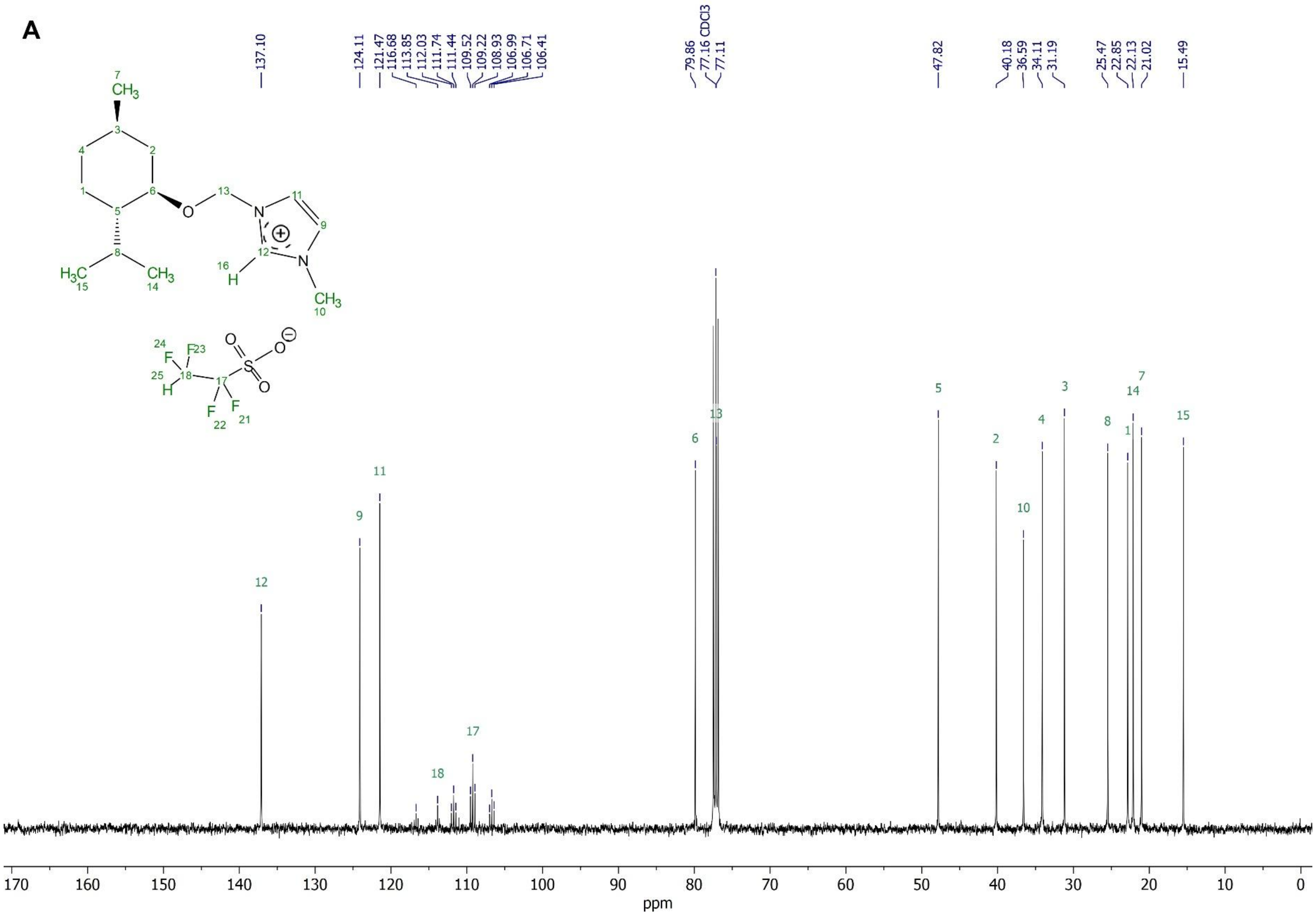
137.10
124.11
121.47
116.68
113.85
112.03
111.74
111.44
109.52
109.22
108.93
106.99
106.71
106.41
79.86
77.16 CDCl3
77.11
47.82
40.18
36.59
34.11
31.19
25.47
22.85
22.13
21.02
15.49
ppm

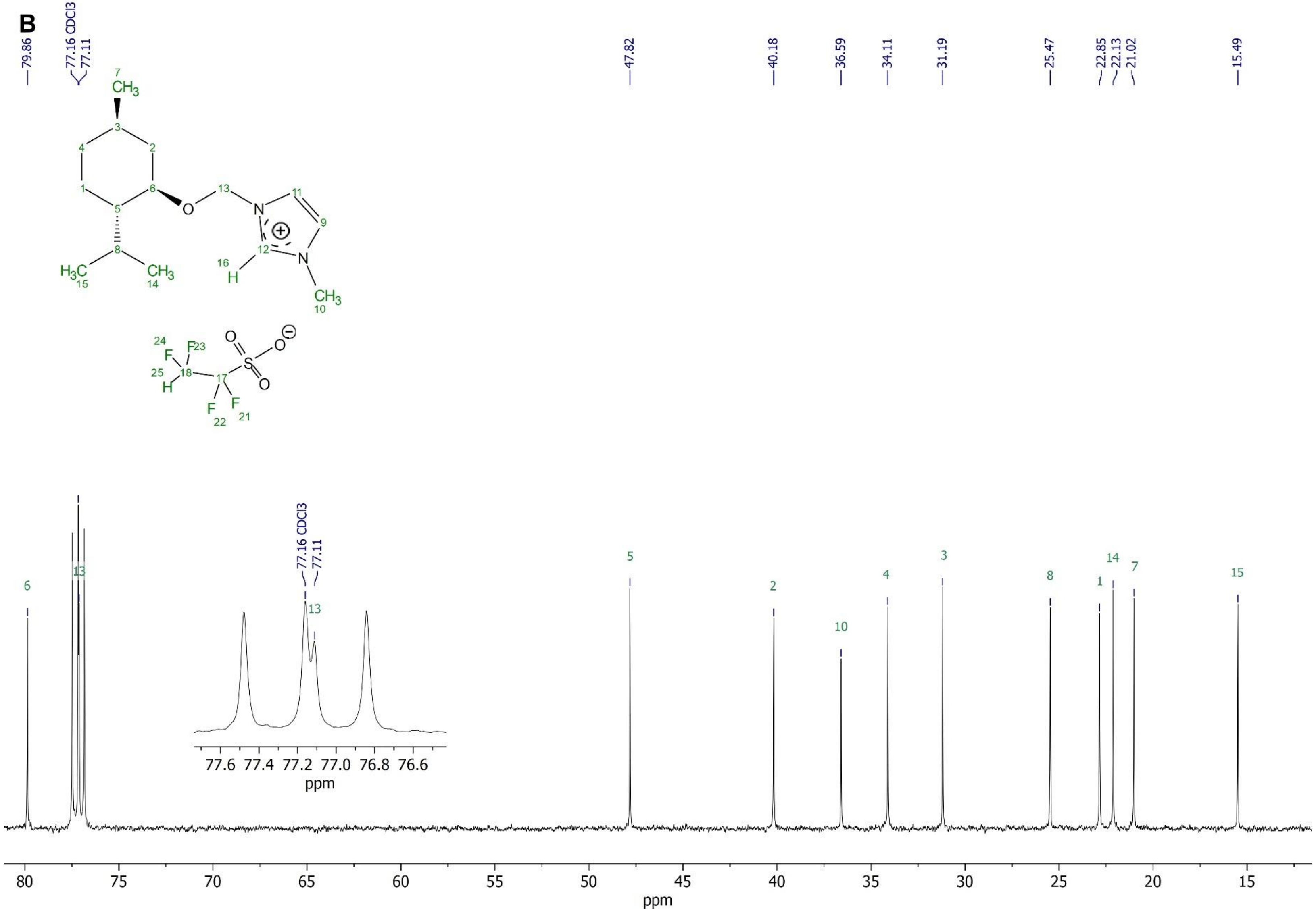

B
79.86
77.16 CDCl3
77.11
47.82
40.18
36.59
34.11
31.19
25.47
22.85
22.13
21.02
15.49
77.6 77.4 77.2 77.0 76.8 76.6
ppm
80 75 70 65 60 55 50 45 40 35 30 25 20 15
ppm

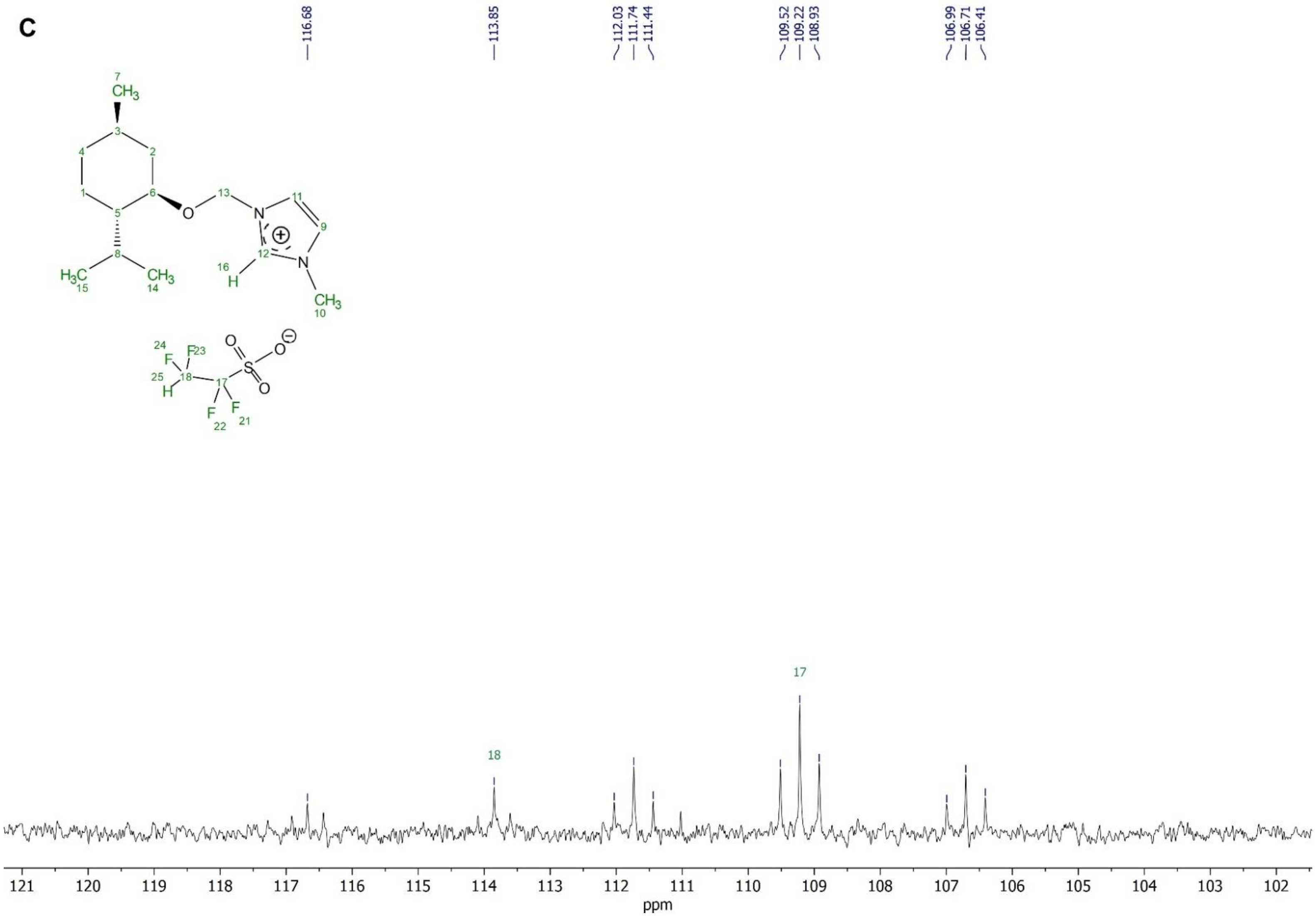

Figure S24. ^{13}C NMR (100 MHz) spectra of [Men-Im-C_1][TFES] (**4a**) in $CDCl_3$. **A.** region from 0.0 ppm to 170.0 ppm. **B.** region from 14.0 ppm to 80.0 ppm. **C.** region from 102.0 ppm to 121.0 ppm.

A

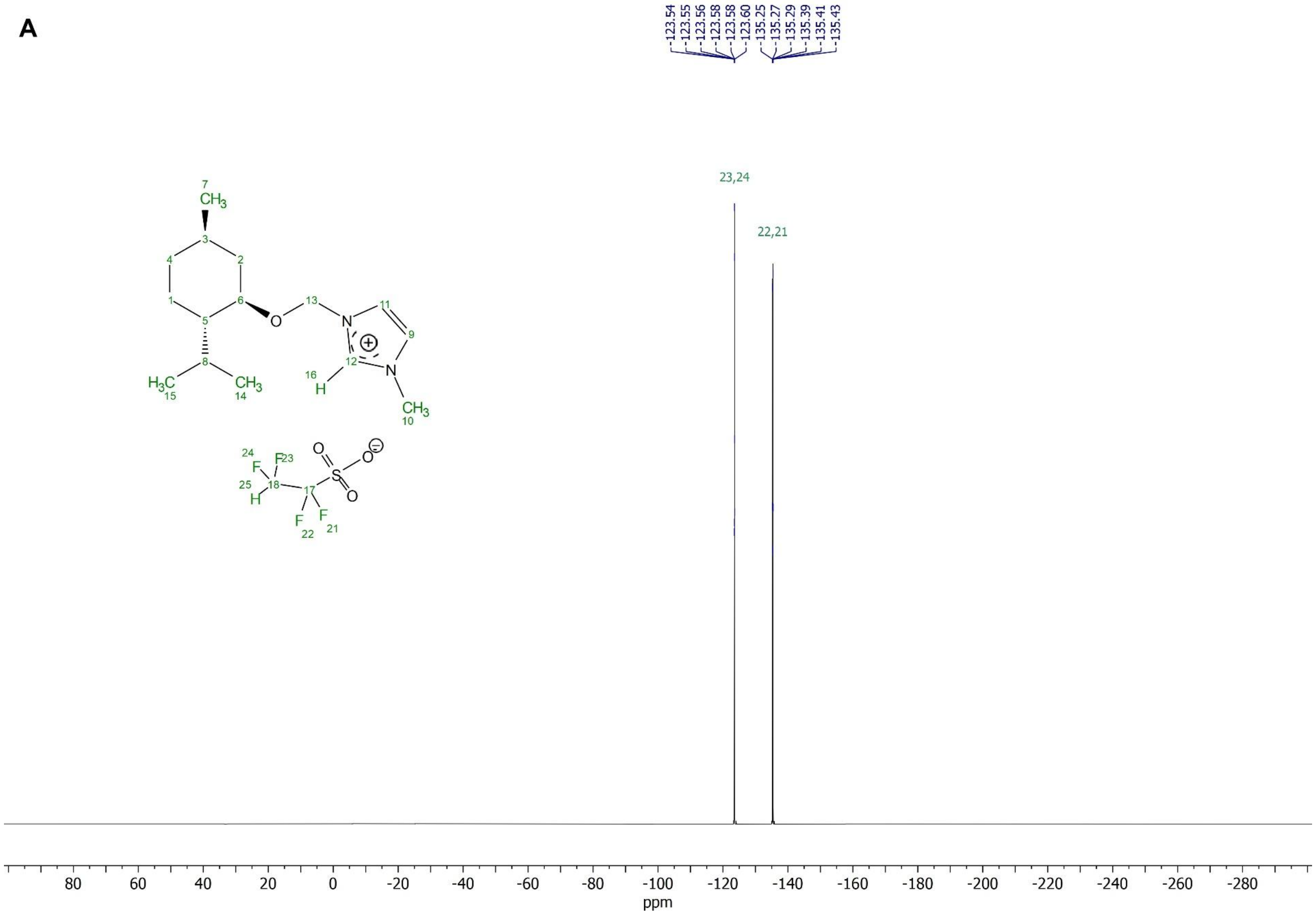

-123.54
-123.55
-123.56
-123.58
-123.58
-123.60
-135.25
-135.27
-135.29
-135.39
-135.41
-135.43
23,24
22,21
80 60 40 20 0 -20 -40 -60 -80 -100 -120 -140 -160 -180 -200 -220 -240 -260 -280
ppm

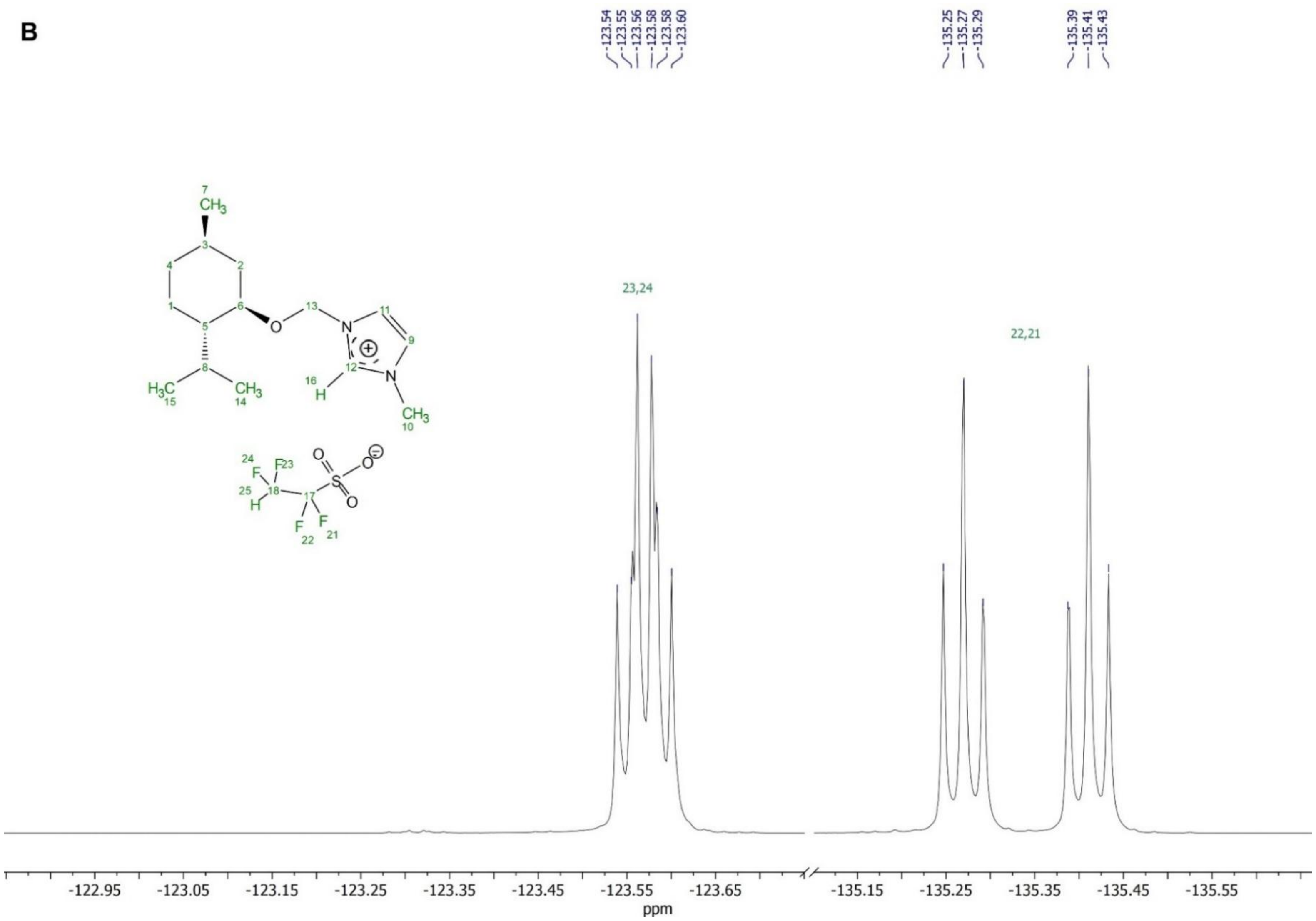

Figure S25. ^{19}F NMR (100 MHz) spectra of [Men-Im-C_1][TFES] (**4a**) in $CDCl_3$. **A.** region from -280.0 ppm to 80.0 ppm. **B.** region from -135.5 ppm to -122.9 ppm.

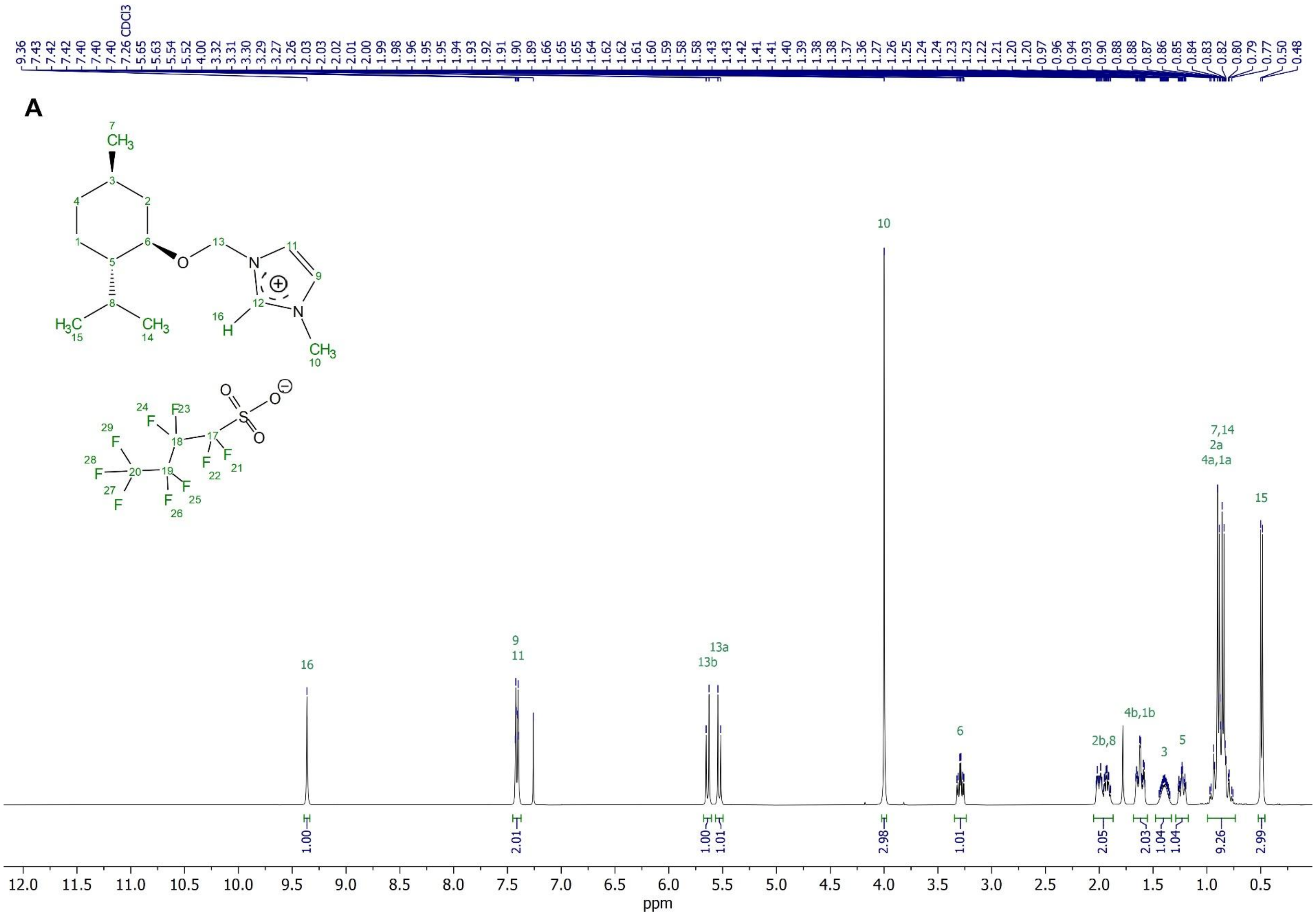
A
ppm

B

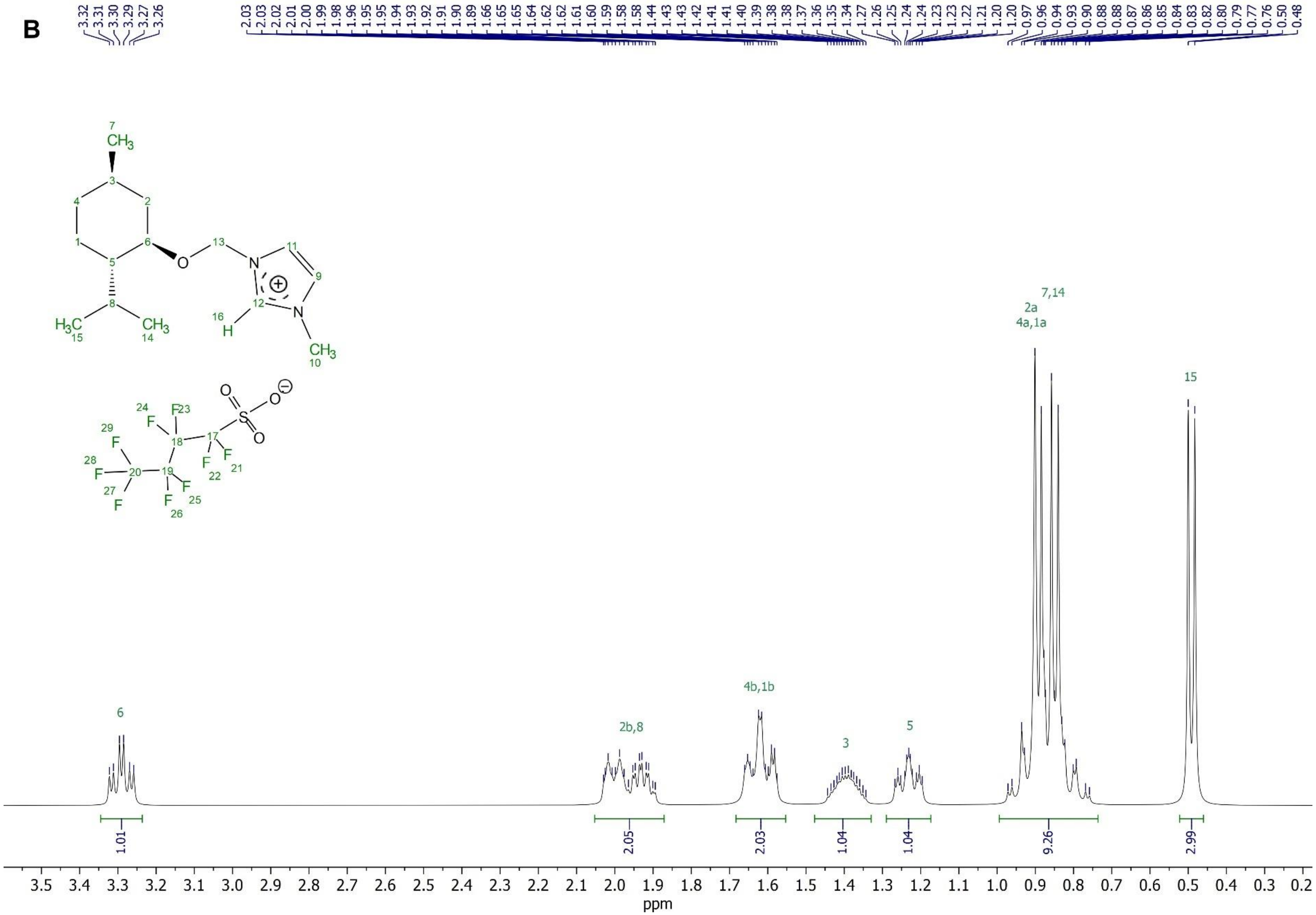

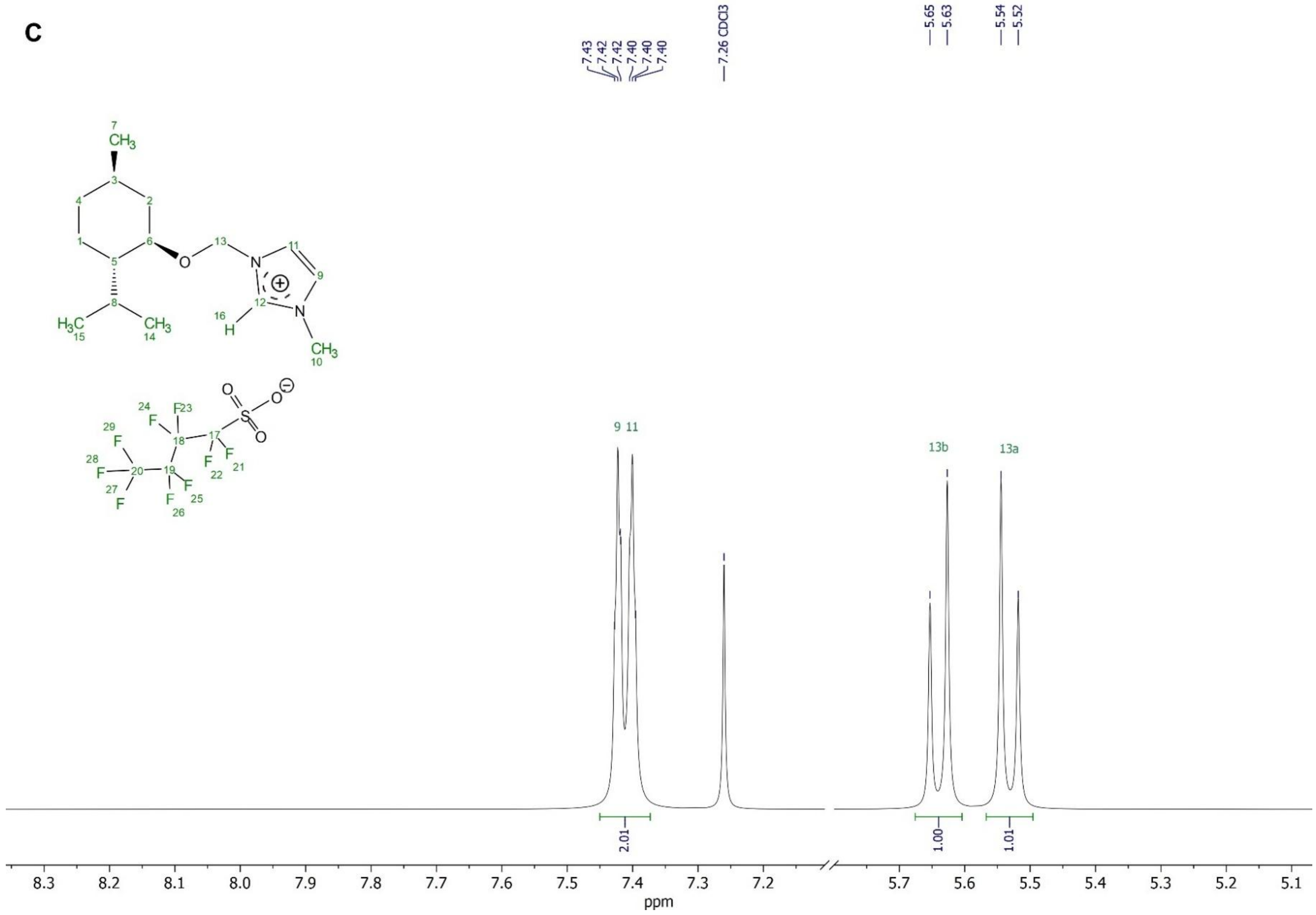

Figure S26. ^{1}H NMR (400 MHz) spectra of [Men-Im-C_1][PFBS] (**4b**) in $CDCl_3$. **A.** region from 0.0 ppm to 12.0 ppm. **B.** region from 0.2 ppm to 3.5 ppm. **C.** region from 5.1 ppm to 8.3 ppm.

A

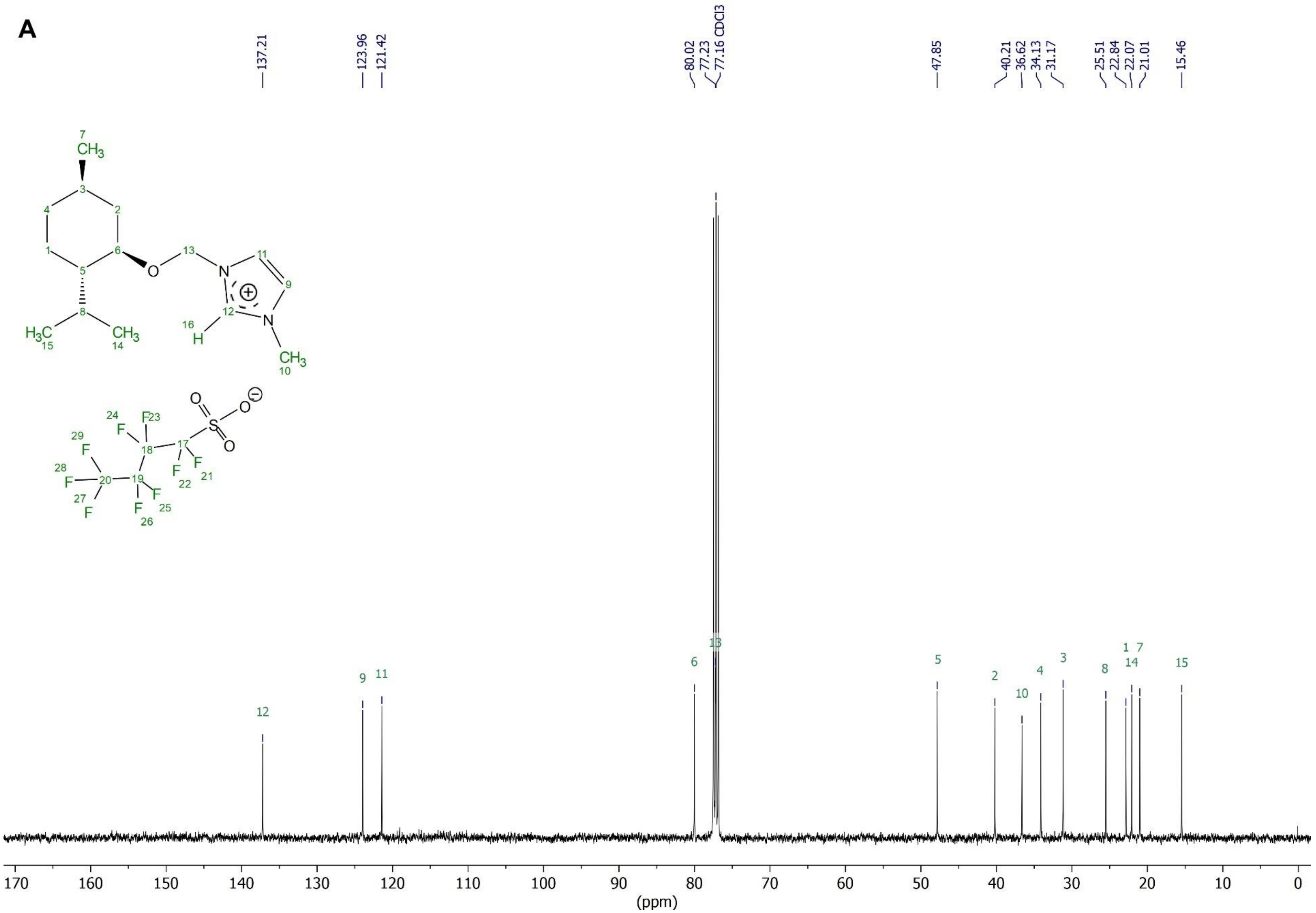

137.21
123.96
121.42
80.02
77.23
77.16 CDCl3
47.85
40.21
36.62
34.13
31.17
25.51
22.84
22.07
21.01
15.46
(ppm)

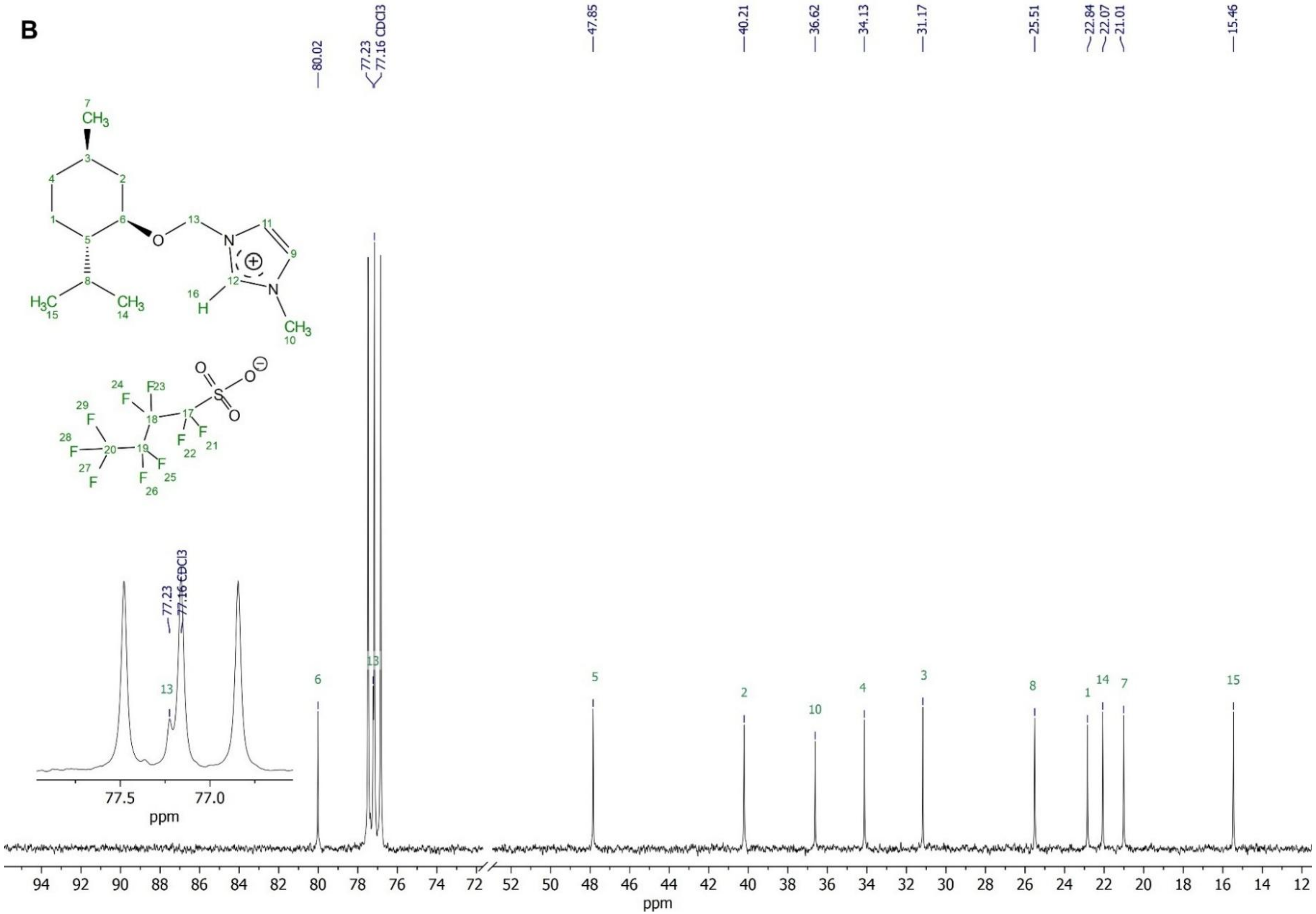

Figure S27. ^{13}C NMR (100 MHz) spectra of [Men-Im-C_1][PFBS] (**4b**) in $CDCl_3$. **A.** region from 0.0 ppm to 170.0 ppm. **B.** region from 12.0 ppm to 94.0 ppm.

A

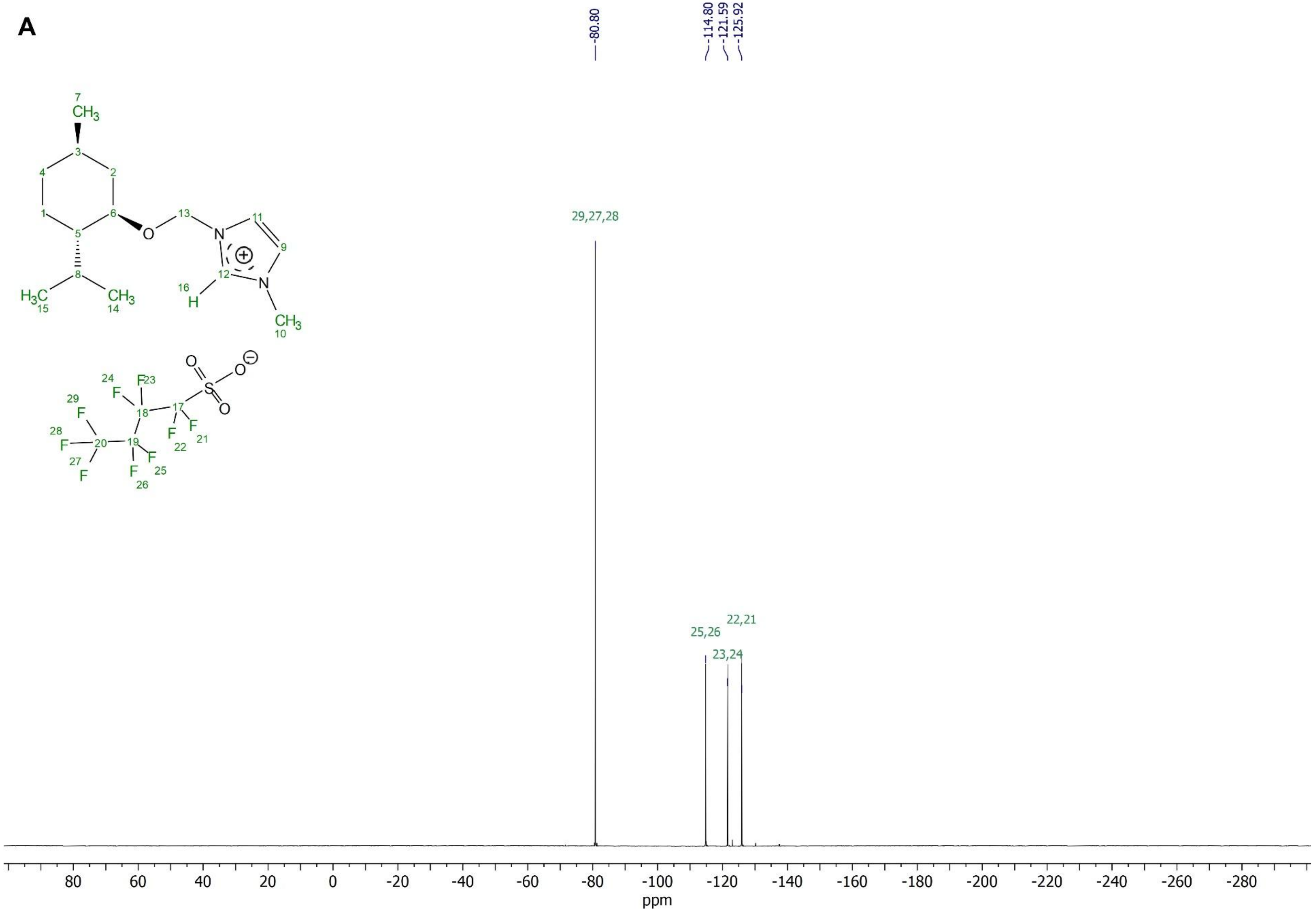

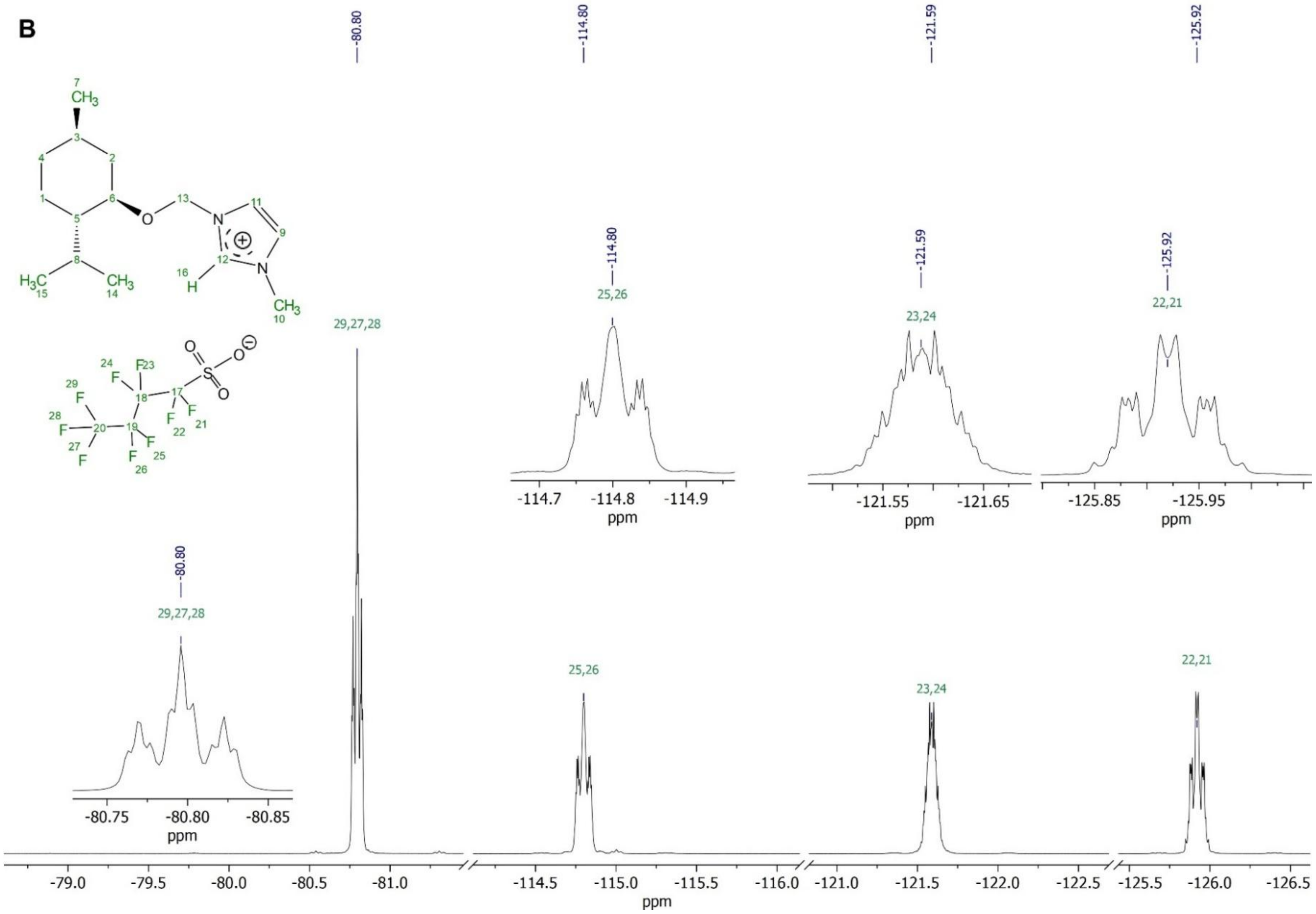

Figure S28. ^{19}F NMR (100 MHz) spectra of [Men-Im-C_1][PFBS] (**4b**) in $CDCl_3$. **A.** region from -280.0 ppm to 80.0 ppm. **B.** region from 126.5 ppm to -80.5 ppm.

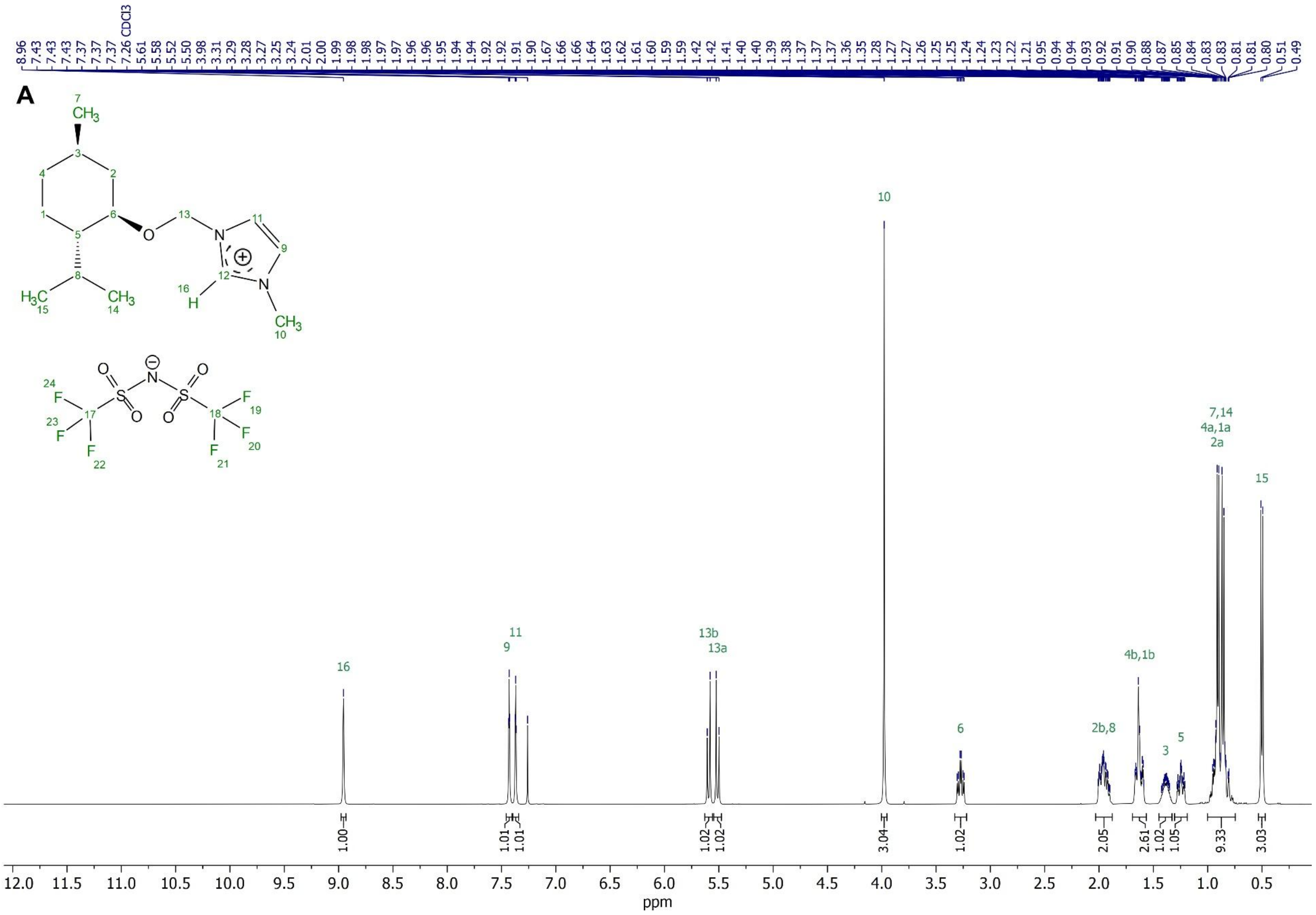

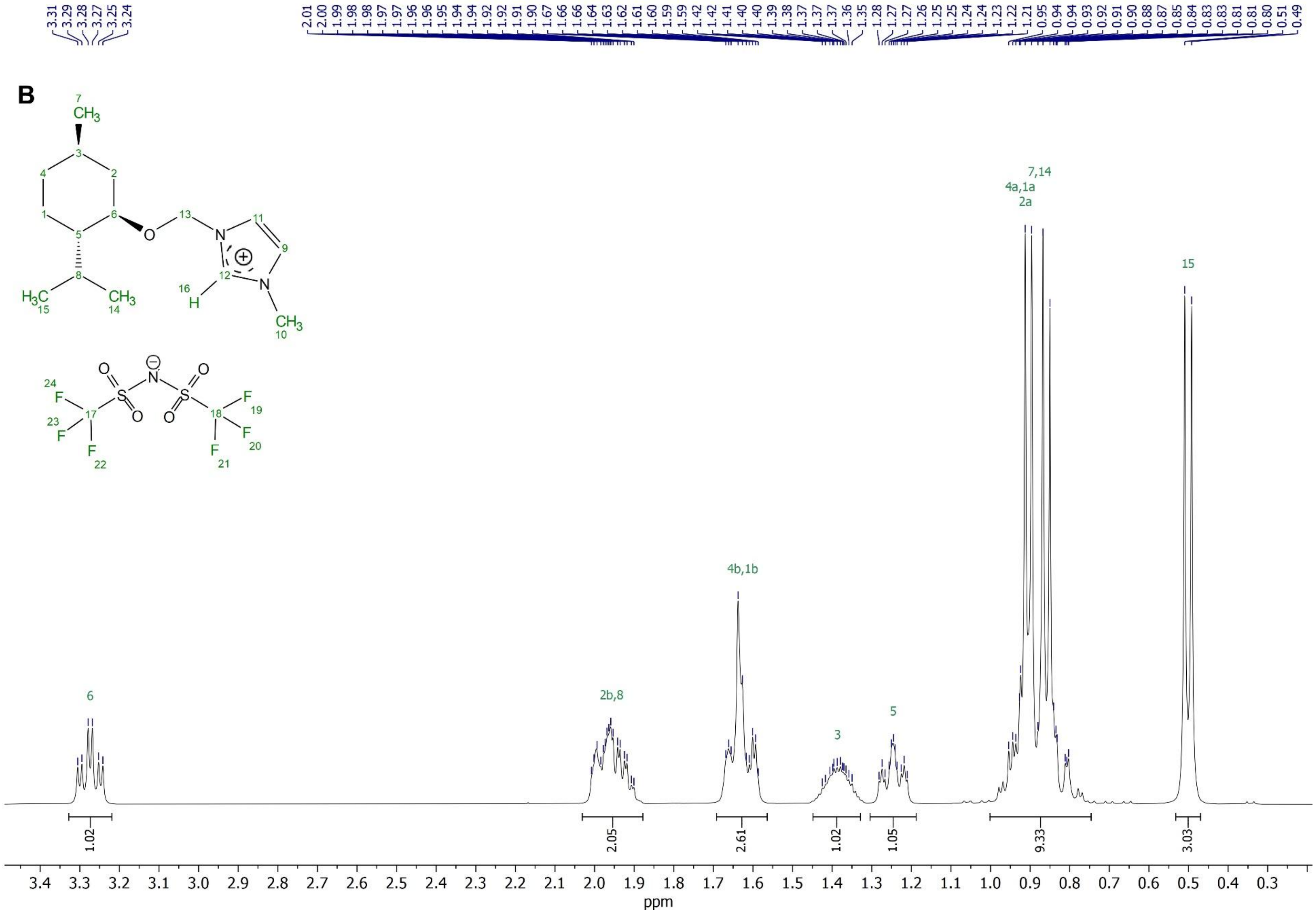
B
4a,1a
2a
7,14
15
4b,1b
6
2b,8
3
5
1.02
2.05
2.61
1.02
1.05
9.33
3.03
ppm

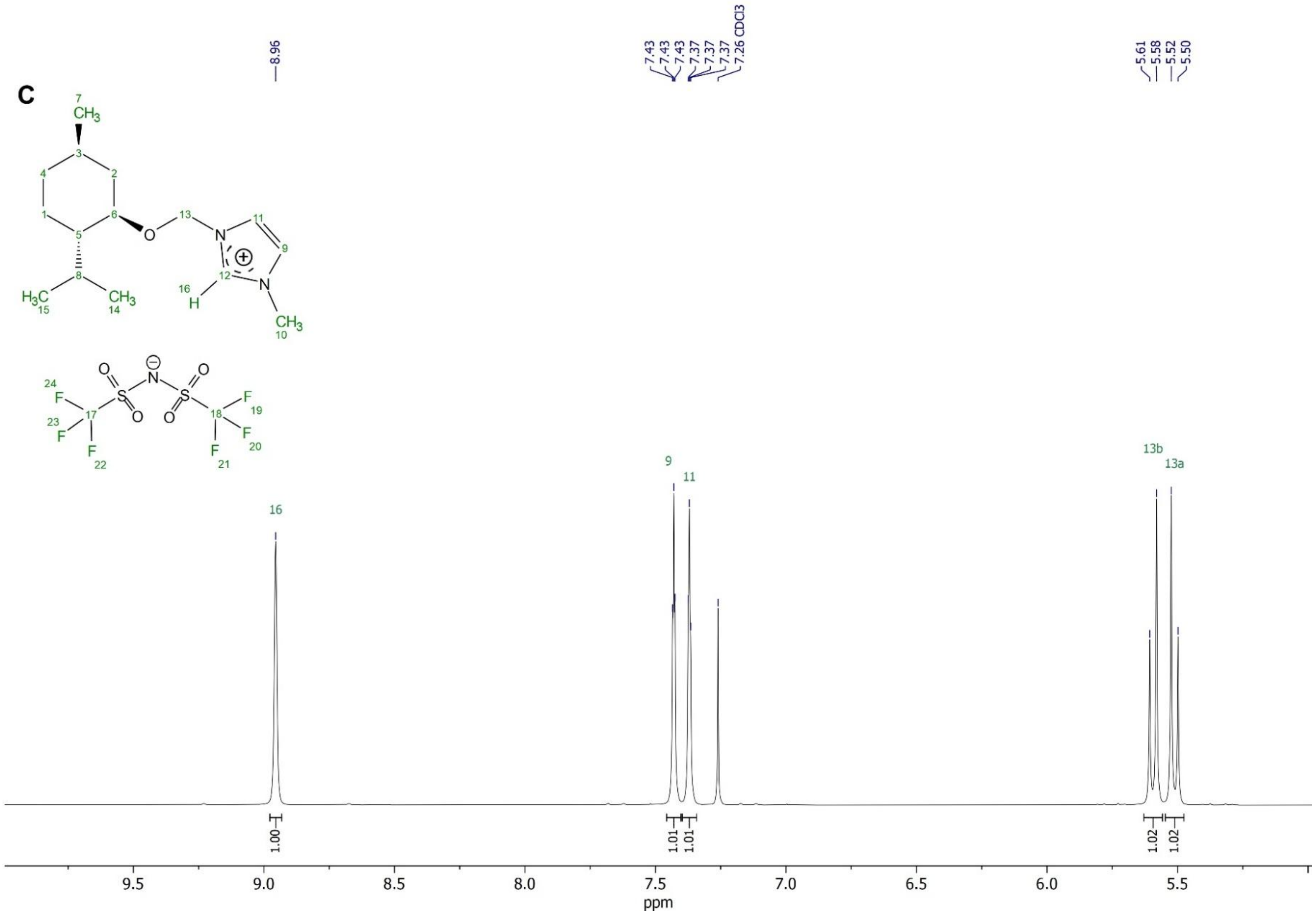

Figure S29. ^{1}H NMR (400 MHz) spectra of [Men-Im-C_1][TFSI] (**4c**) in $CDCl_3$. **A.** region from 0.0 ppm to 12.0 ppm. **B.** region from 0.3 ppm to 3.4 ppm. **C.** region from 5.0 ppm to 9.5 ppm.

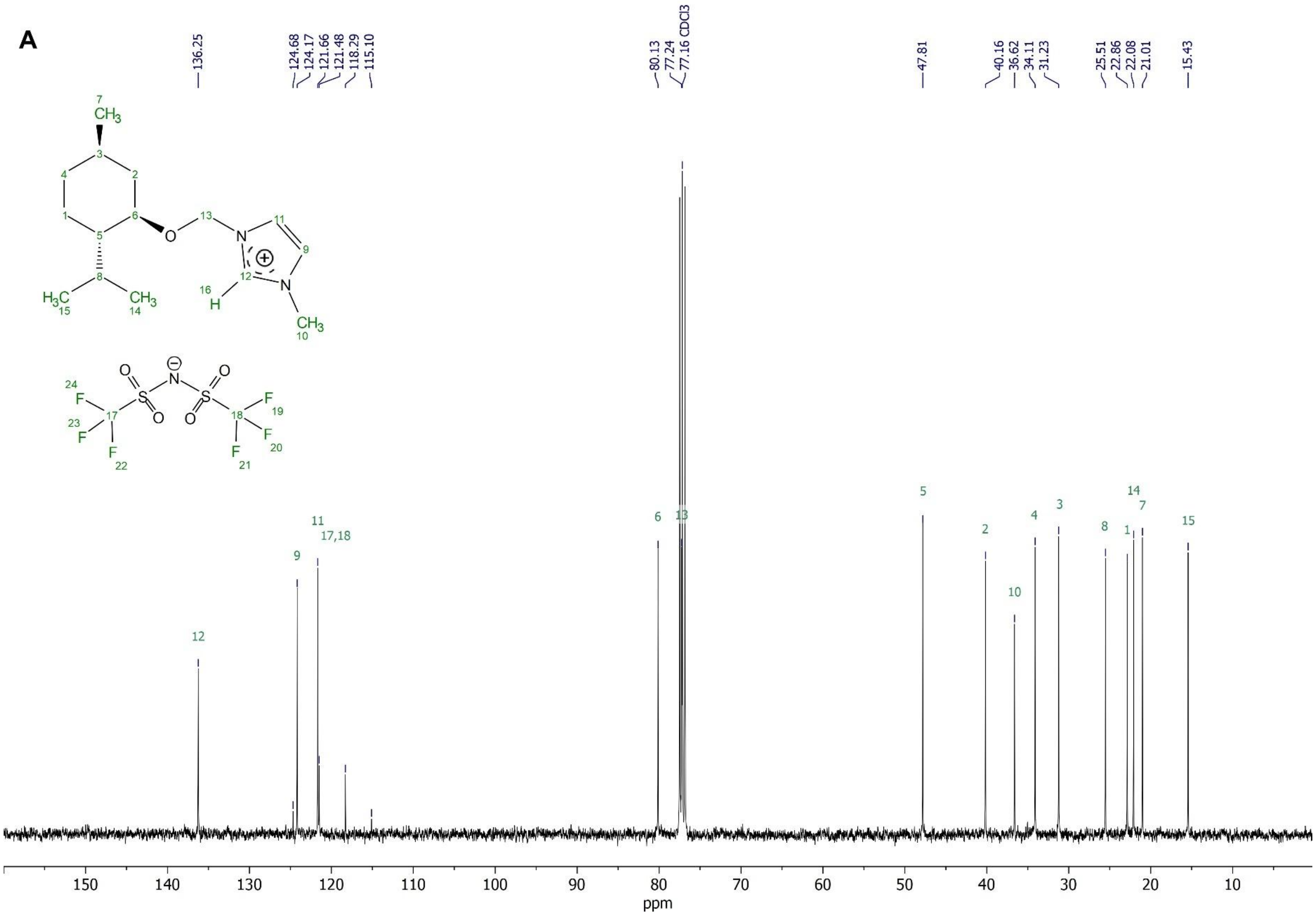
A
136.25
124.68
124.17
121.66
121.48
118.29
115.10
80.13
77.24
77.16 CDCl3
47.81
40.16
36.62
34.11
31.23
25.51
22.86
22.08
21.01
15.43
ppm
150
140
130
120
110
100
90
80
70
60
50
40
30
20
10

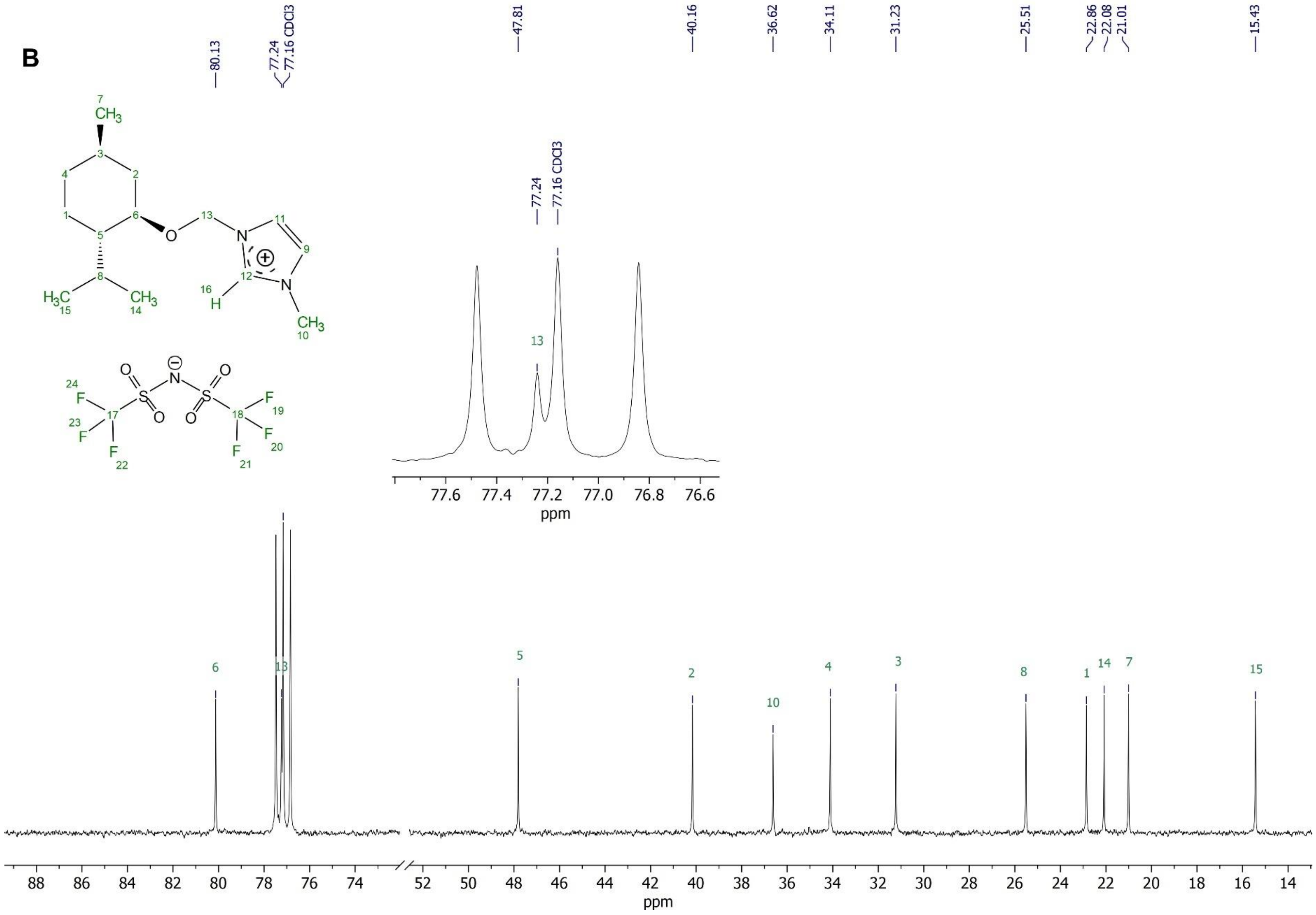
B
80.13
77.24
77.16 CDCl3
47.81
40.16
36.62
34.11
31.23
25.51
22.86
22.08
21.01
15.43
ppm
ppm

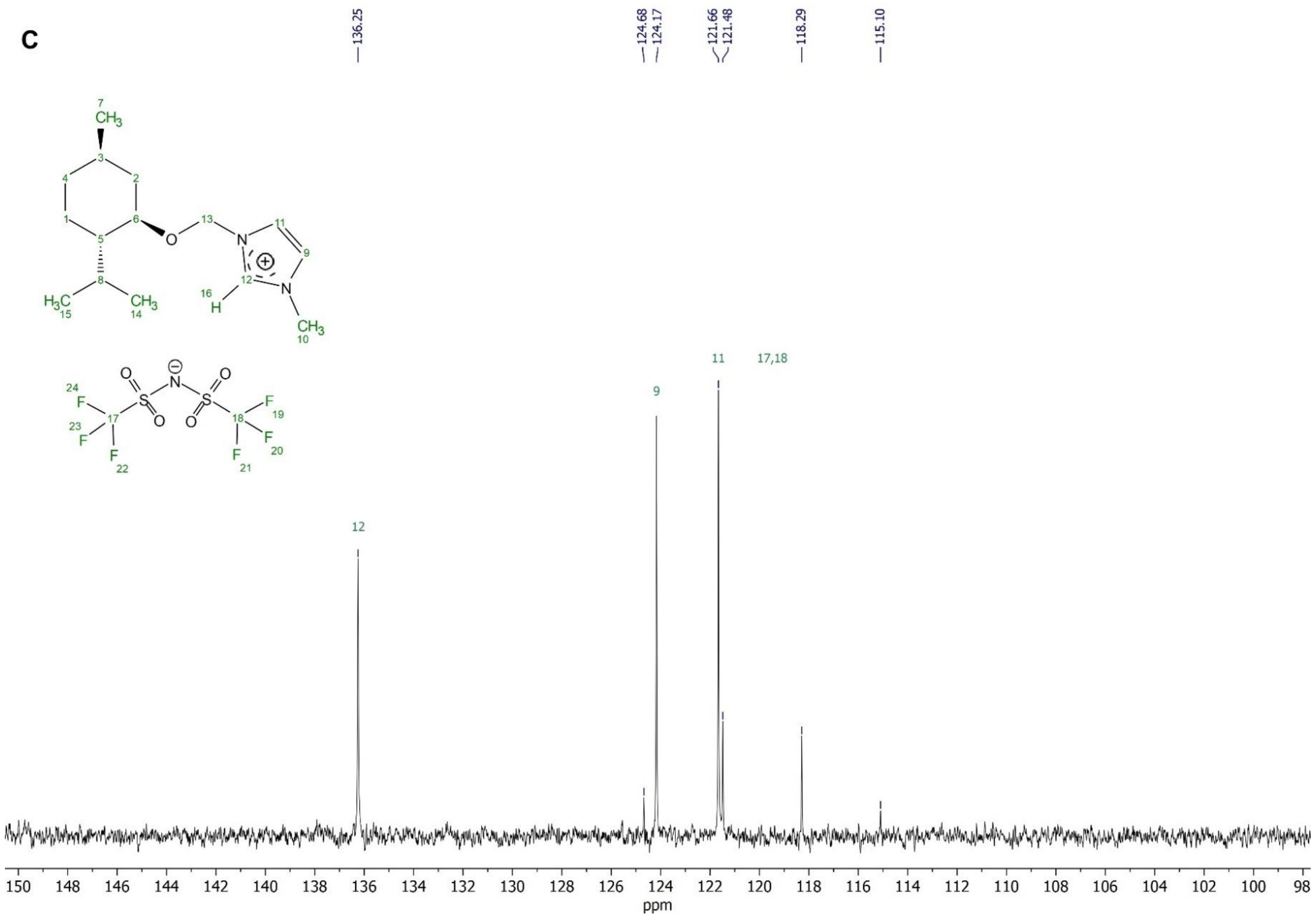

Figure S30. ^{13}C NMR (100 MHz) spectra of [Men-Im-C_1][TFSI] (**4c**) in $CDCl_3$. **A.** region from 0.0 ppm to 150.0 ppm. **B.** region from 14.0 ppm to 88.0 ppm. **C.** region from 98.0 ppm to 150.0 ppm.

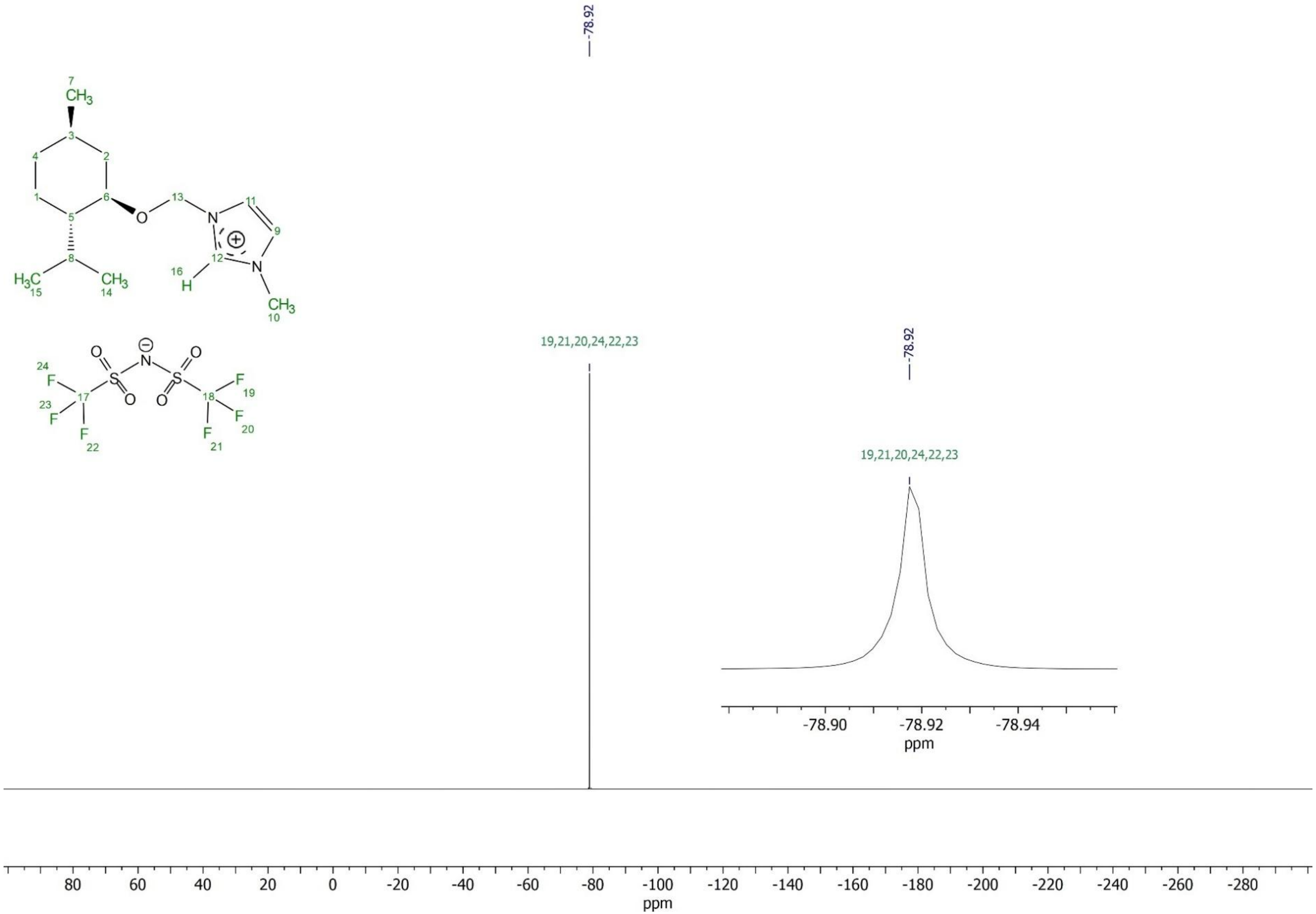

Figure S31. ^{19}F NMR (100 MHz) spectrum of [Men-Im-C_1][TFSI] (**4c**) in $CDCl_3$.

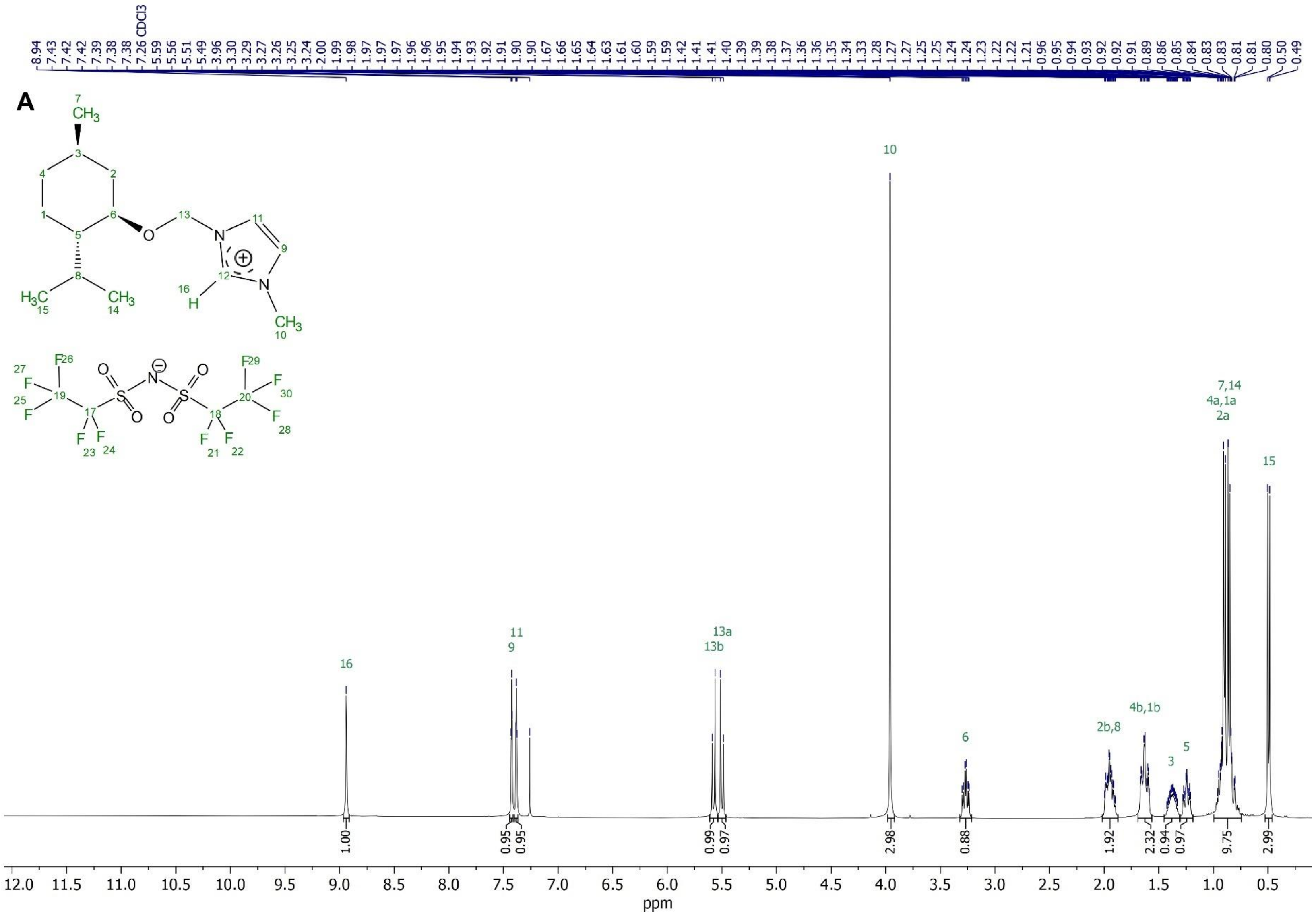
A
10
16
11
9
13a
13b
6
2b,8
4b,1b
3
5
7,14
4a,1a
2a
15
CDCl3
ppm

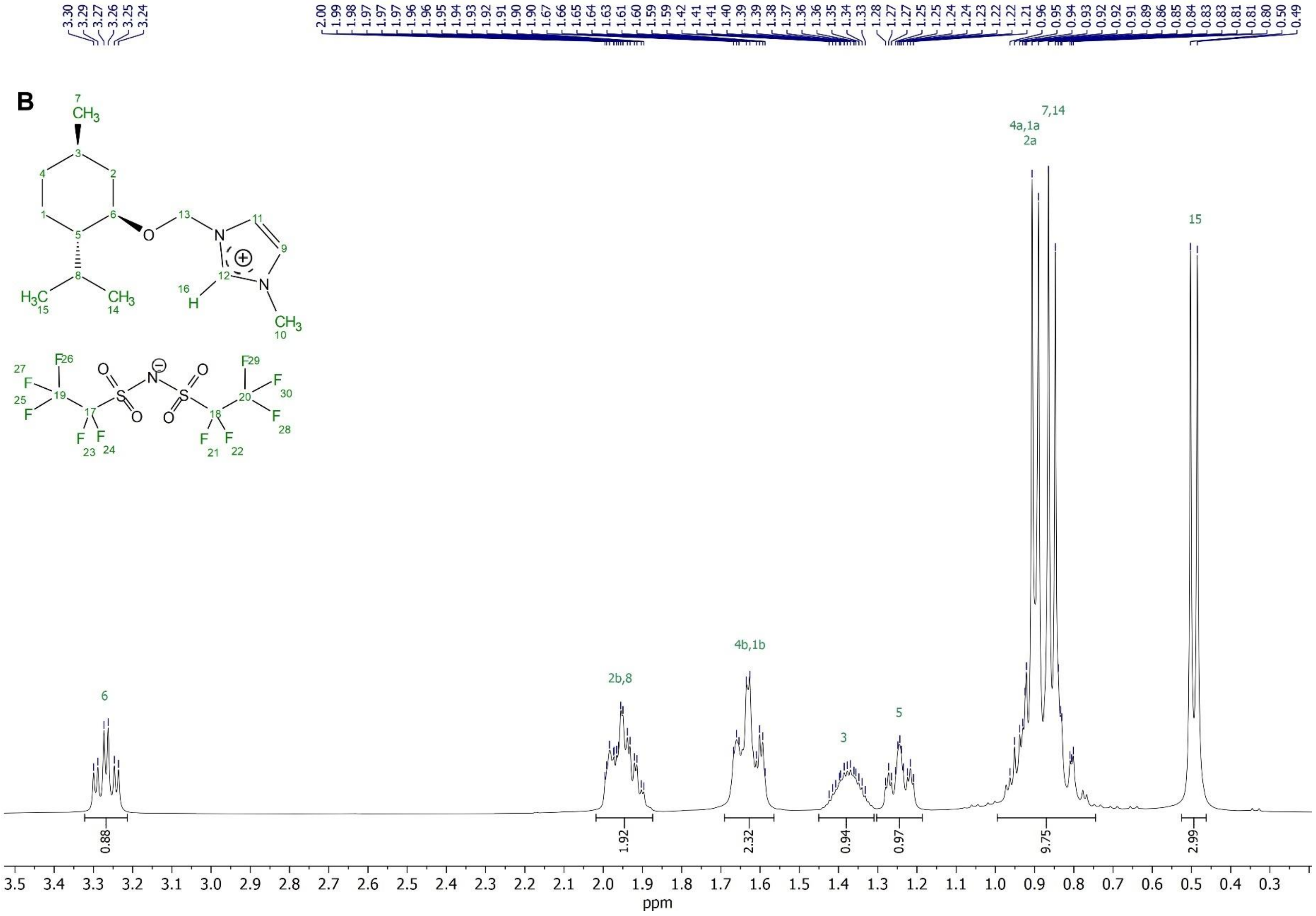
B
ppm
6
2b,8
4b,1b
3
5
4a,1a
2a
7,14
15
0.88
1.92
2.32
0.94
0.97
9.75
2.99

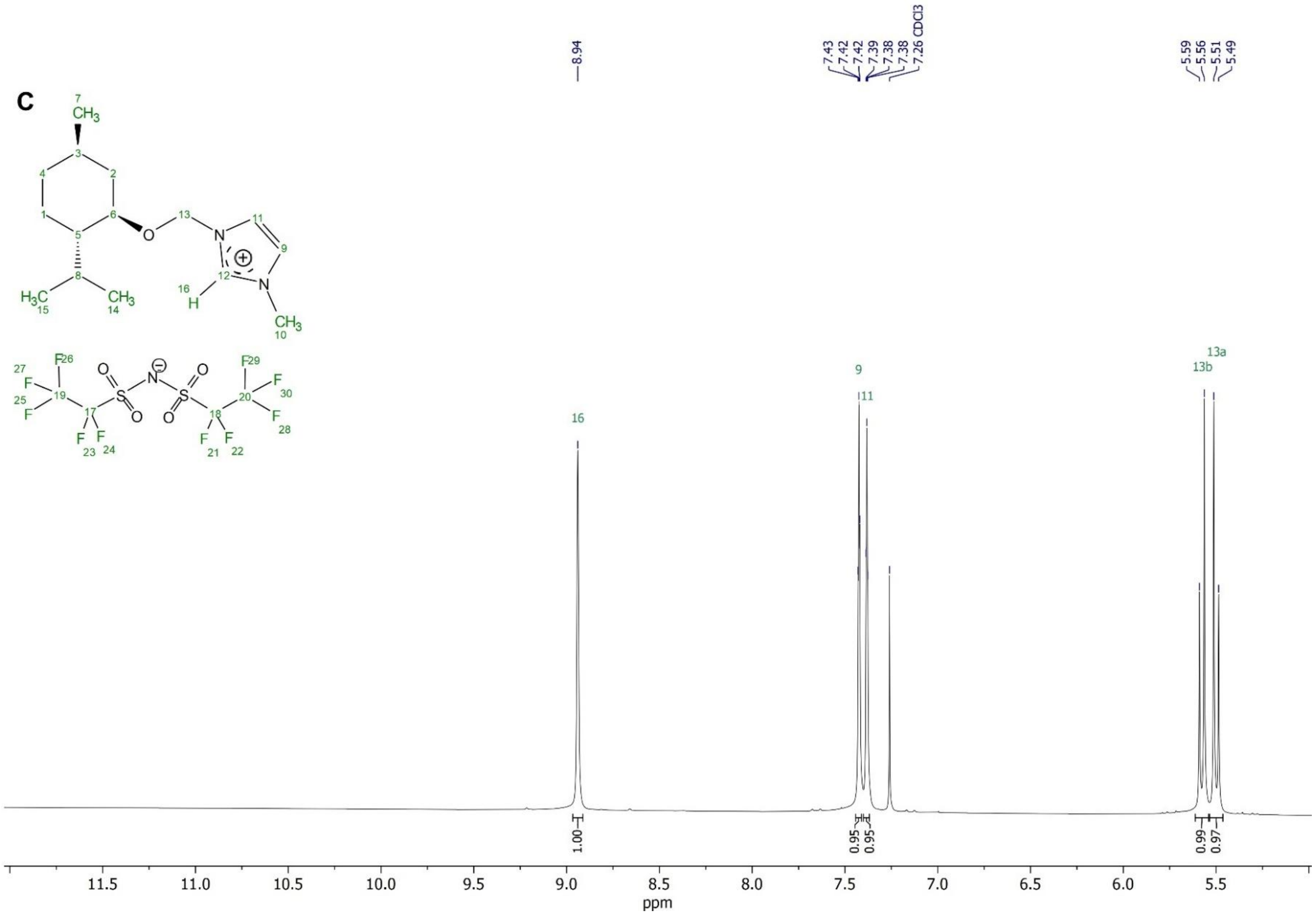

Figure S32. ^{1}H NMR (400 MHz) spectra of [Men-Im-C_1][PFSI] (**4d**) in $CDCl_3$. **A.** region from 0.0 ppm to 12.0 ppm. **B.** region from 0.3 ppm to 3.5 ppm. **C.** region from 5.0 ppm to 12.0 ppm.

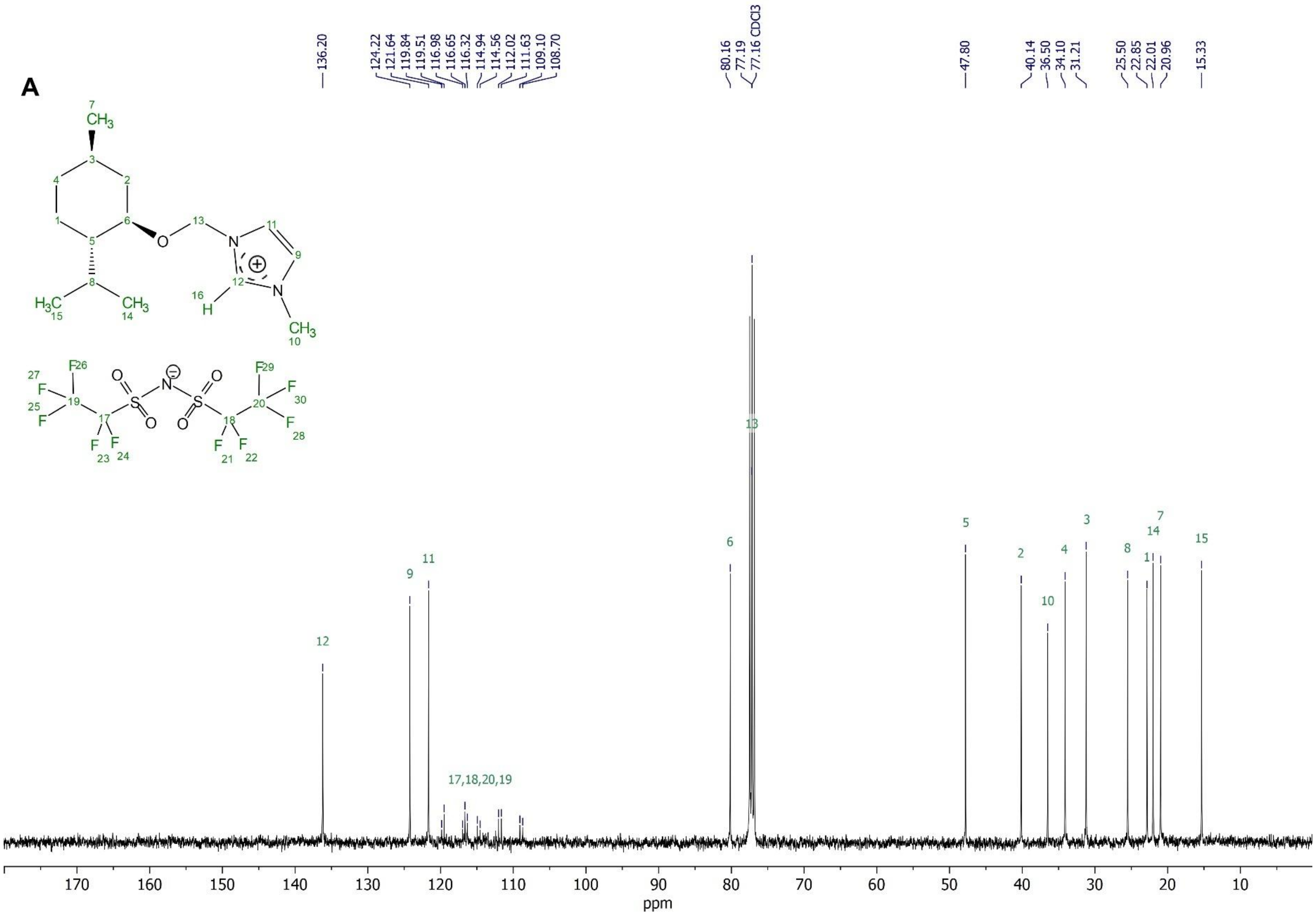
A
136.20
124.22
121.64
119.84
119.51
116.98
116.65
116.32
114.94
114.56
112.02
111.63
109.10
108.70
80.16
77.19
77.16 CDCl3
47.80
40.14
36.50
34.10
31.21
25.50
22.85
22.01
20.96
15.33
17,18,20,19
ppm

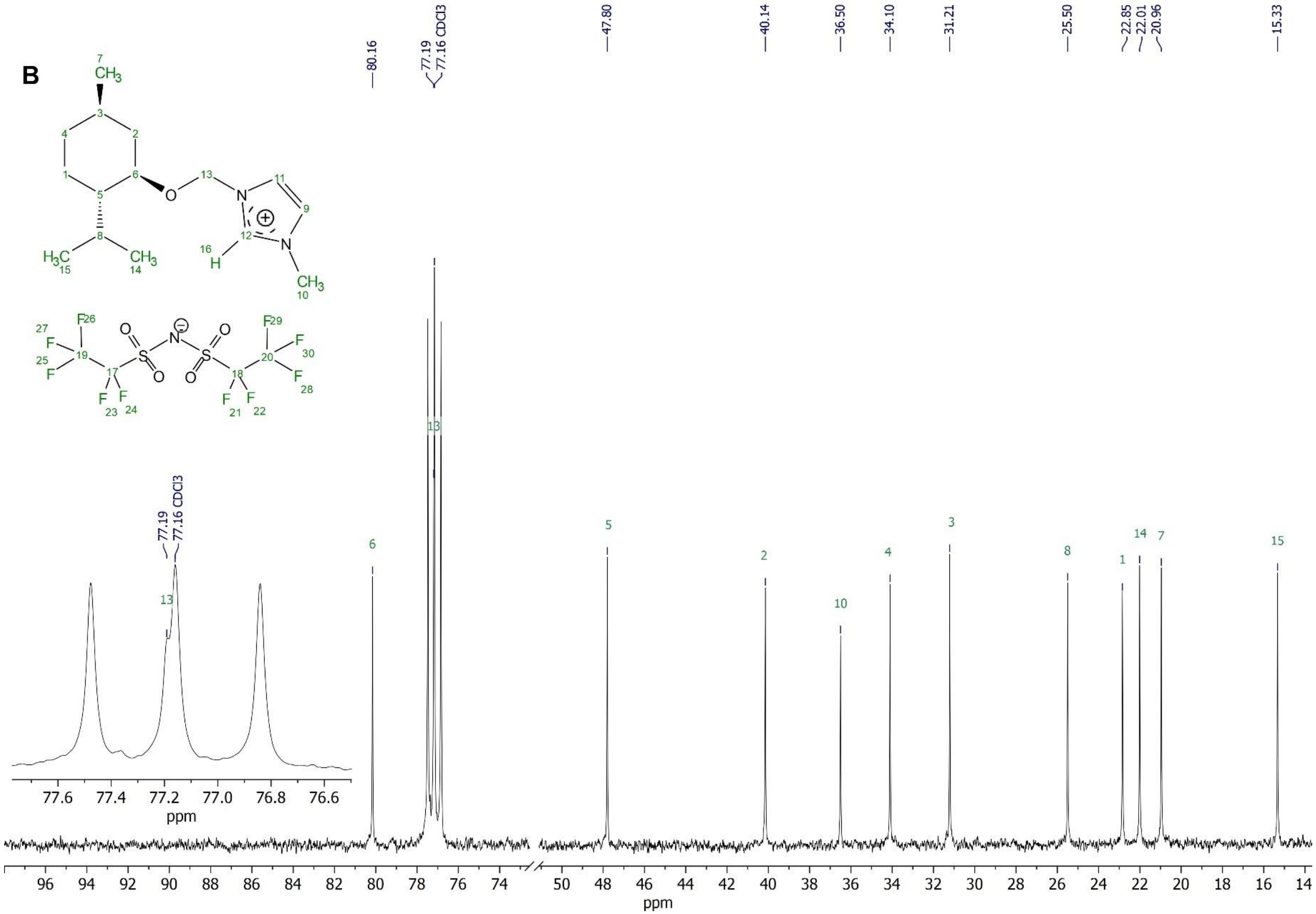

B
80.16
77.19
77.16 CDCl3
47.80
40.14
36.50
34.10
31.21
25.50
22.85
22.01
20.96
15.33
ppm

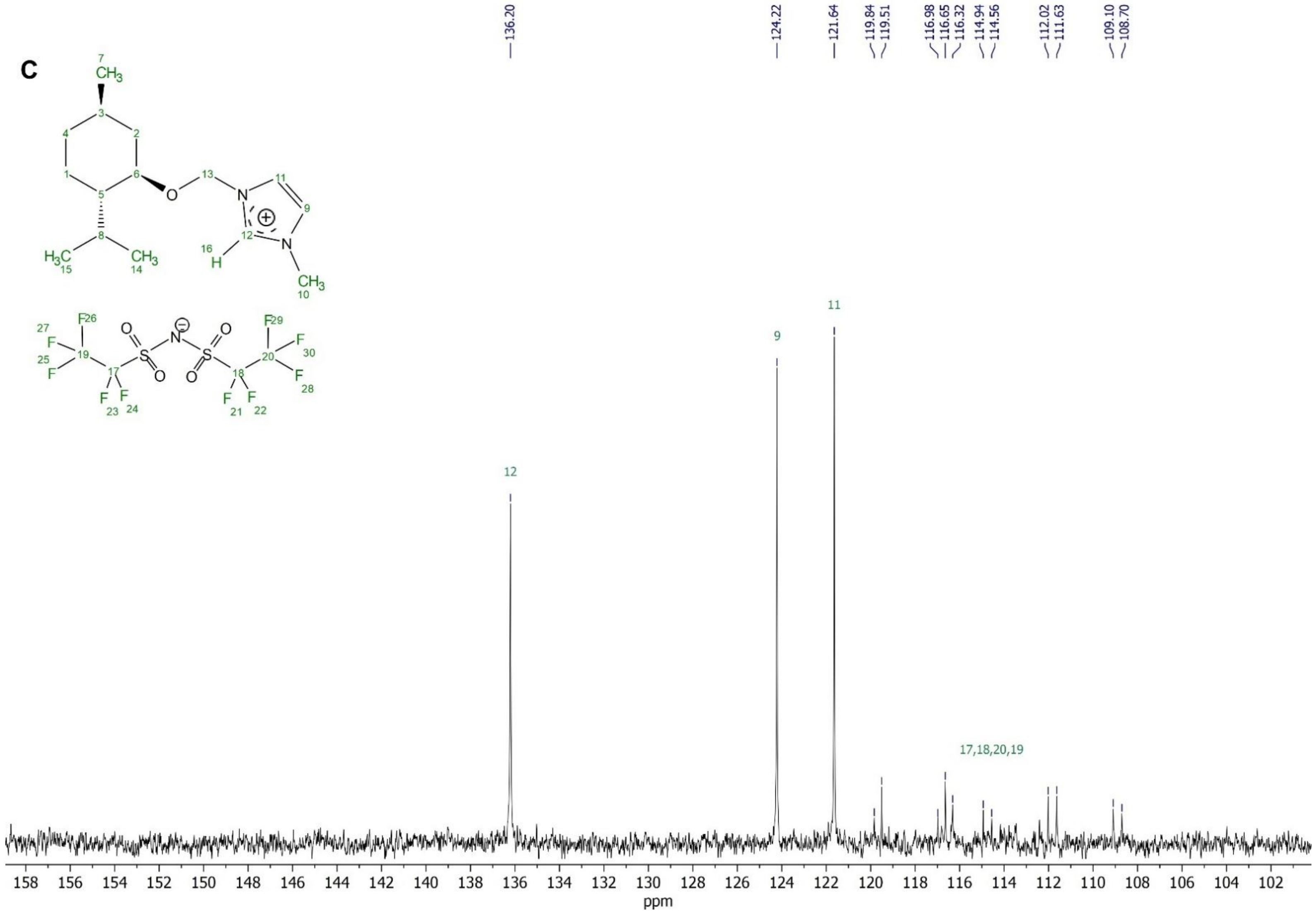

Figure S33. ^{13}C NMR (100 MHz) spectra of [Men-Im-C$_1$][PFSI] (**4d**) in $CDCl_3$. **A.** region from 0.0 ppm to 170.0 ppm. **B.** region from 14.0 ppm to 98.0 ppm. **C.** region from 102.0 ppm to 158.0 ppm.

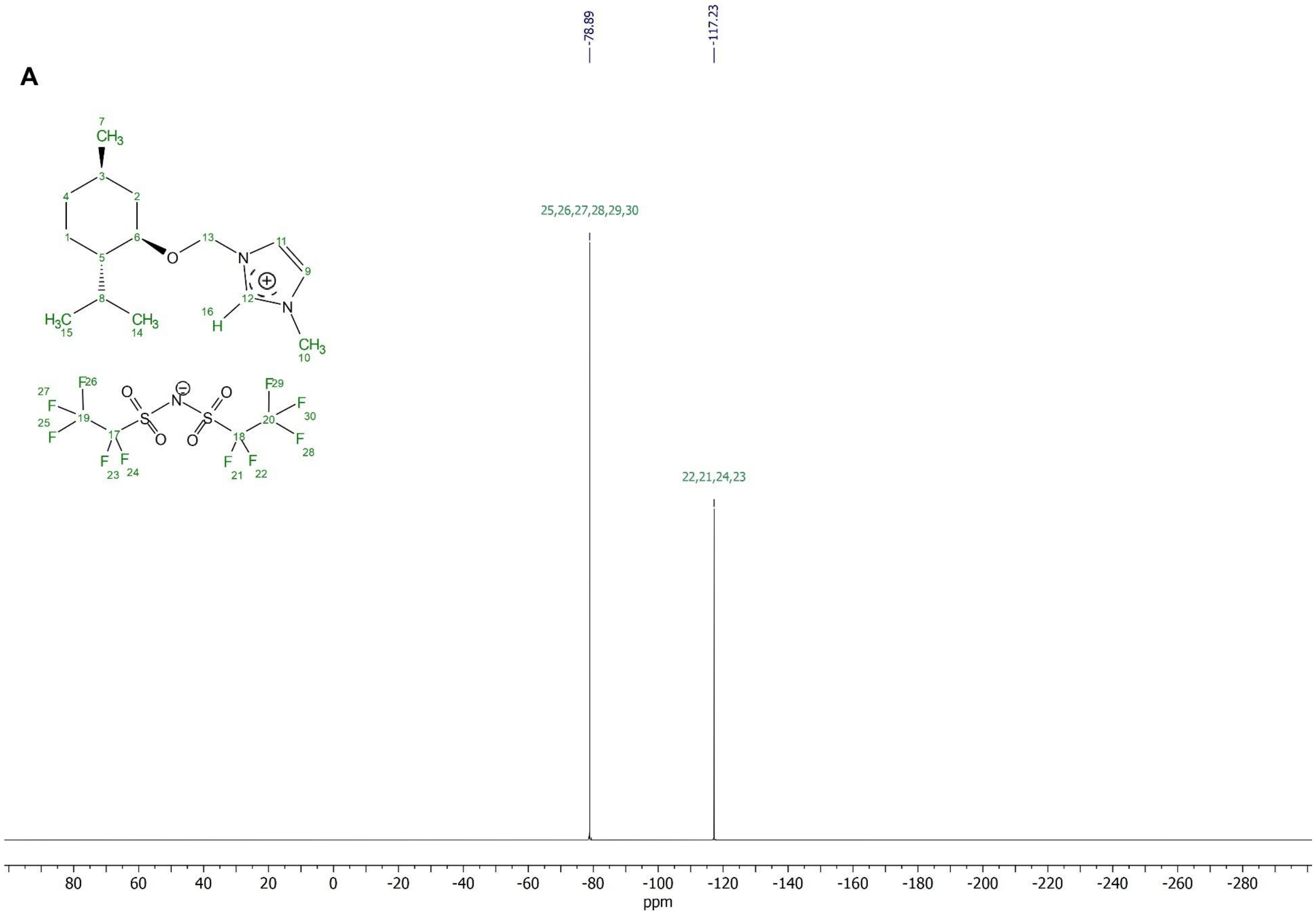

A
-78.89
-117.23
25,26,27,28,29,30
22,21,24,23
80
60
40
20
0
-20
-40
-60
-80
-100
-120
-140
-160
-180
-200
-220
-240
-260
-280
ppm

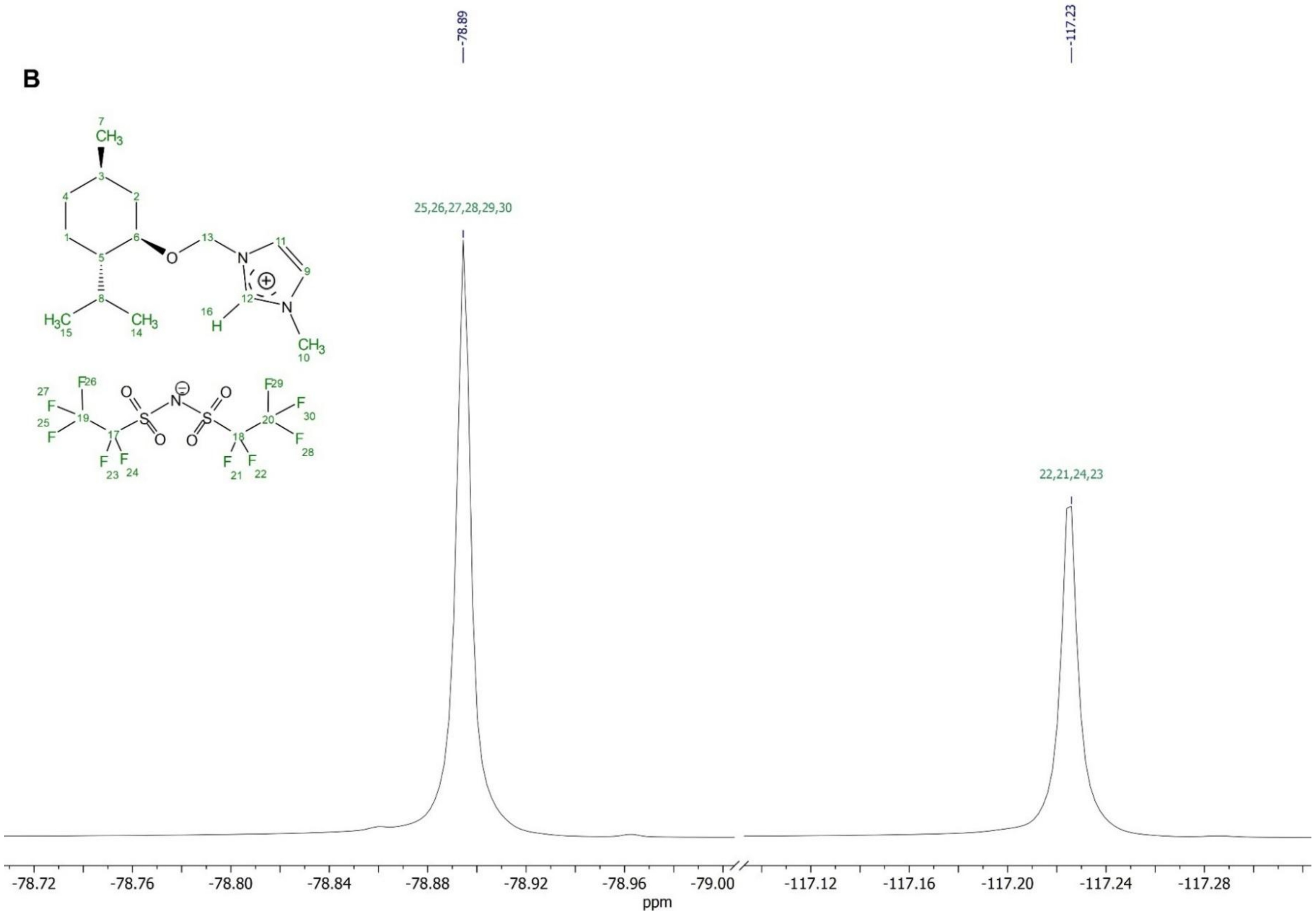

Figure S34. ^{19}F NMR (100 MHz) spectra of [Men-Im-C_1][PFSI] (**4d**) in $CDCl_3$. **A.** region from -280.0 ppm to 70.0 ppm. **B.** region from 117.2 ppm to -78.7 ppm.

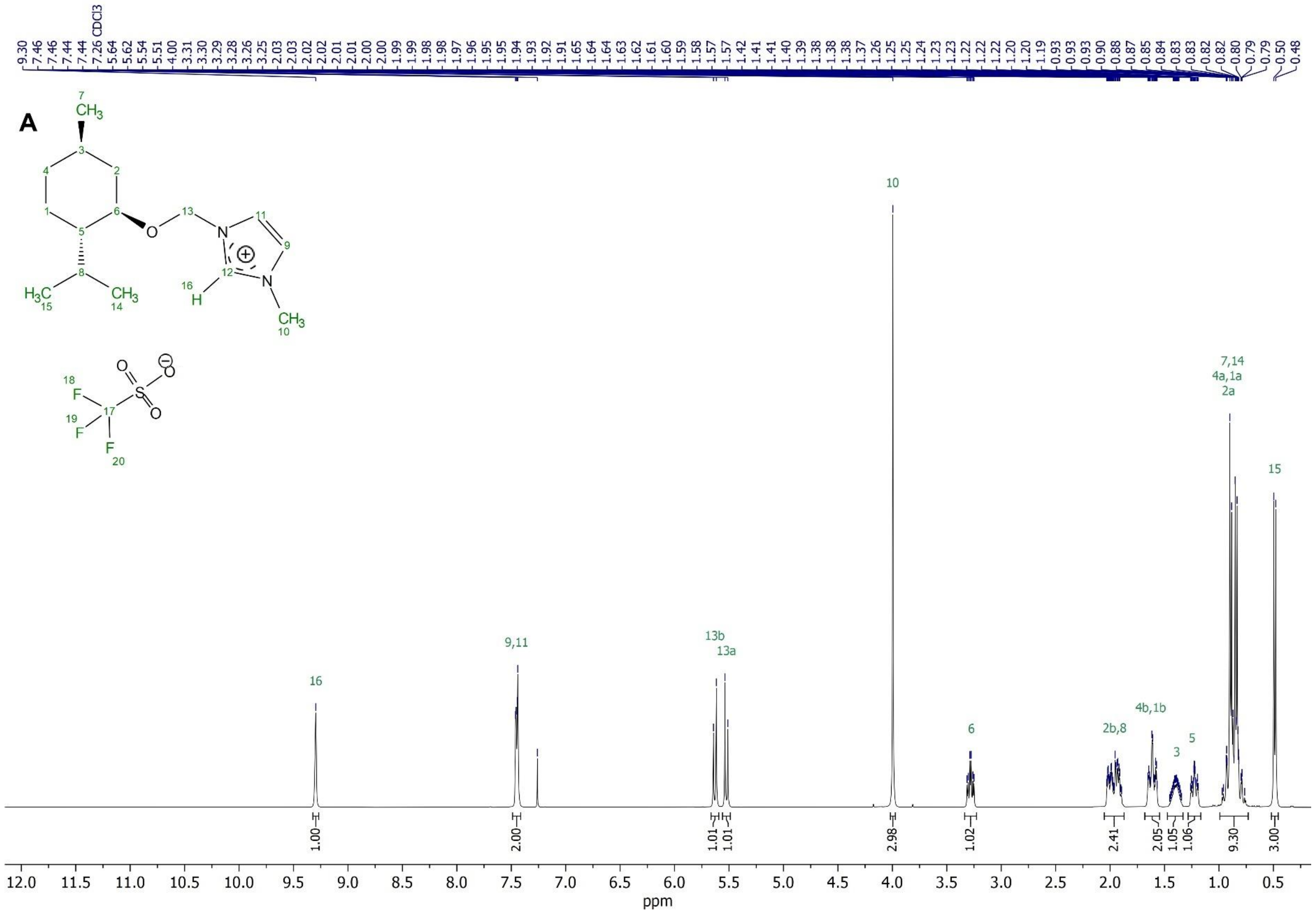

A
ppm
16
9,11
13b
13a
10
6
2b,8
4b,1b
3
5
7,14
4a,1a
2a
15

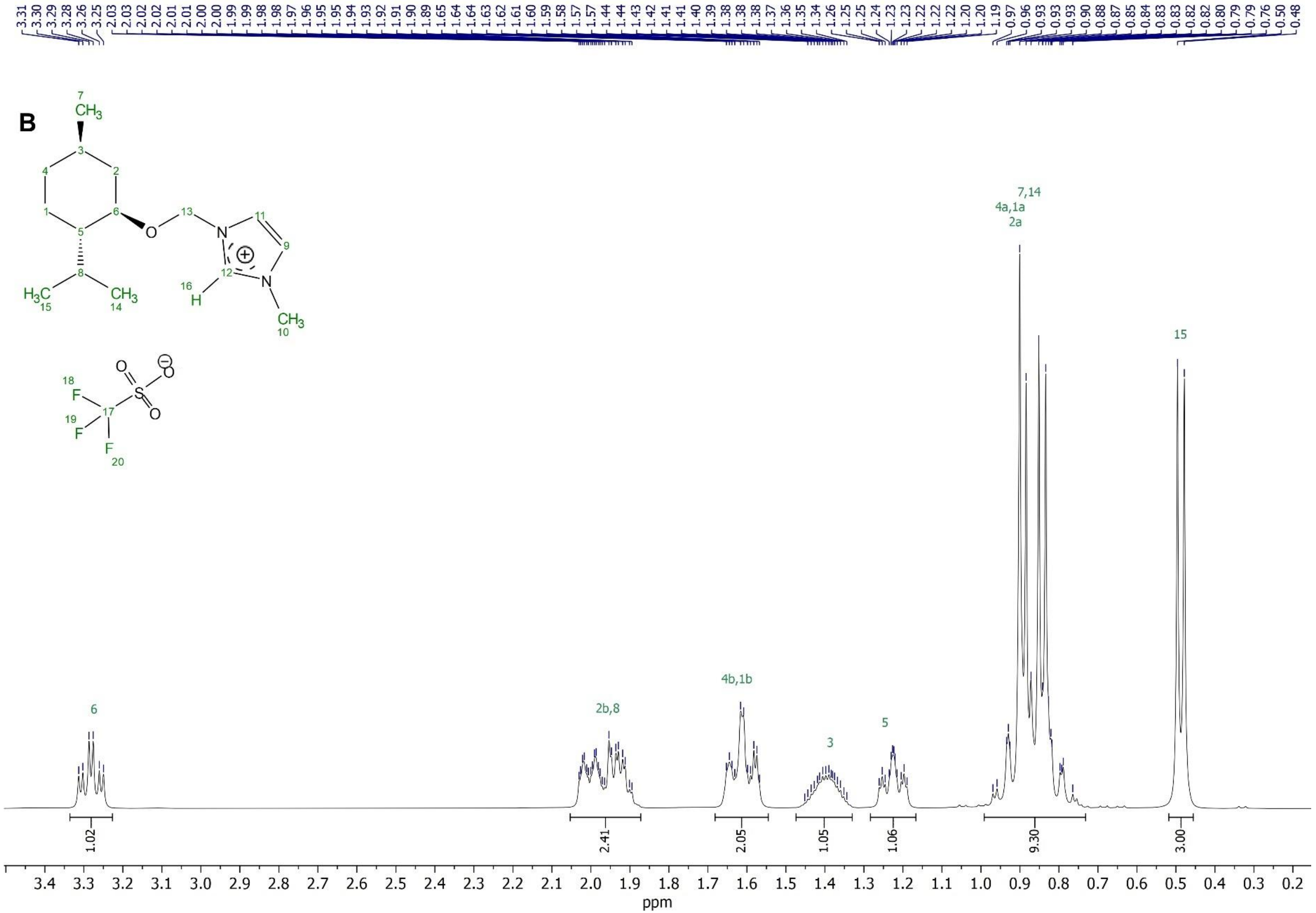
B
6
2b,8
4b,1b
3
5
7,14
4a,1a
2a
15
1.02
2.41
2.05
1.05
1.06
9.30
3.00
ppm

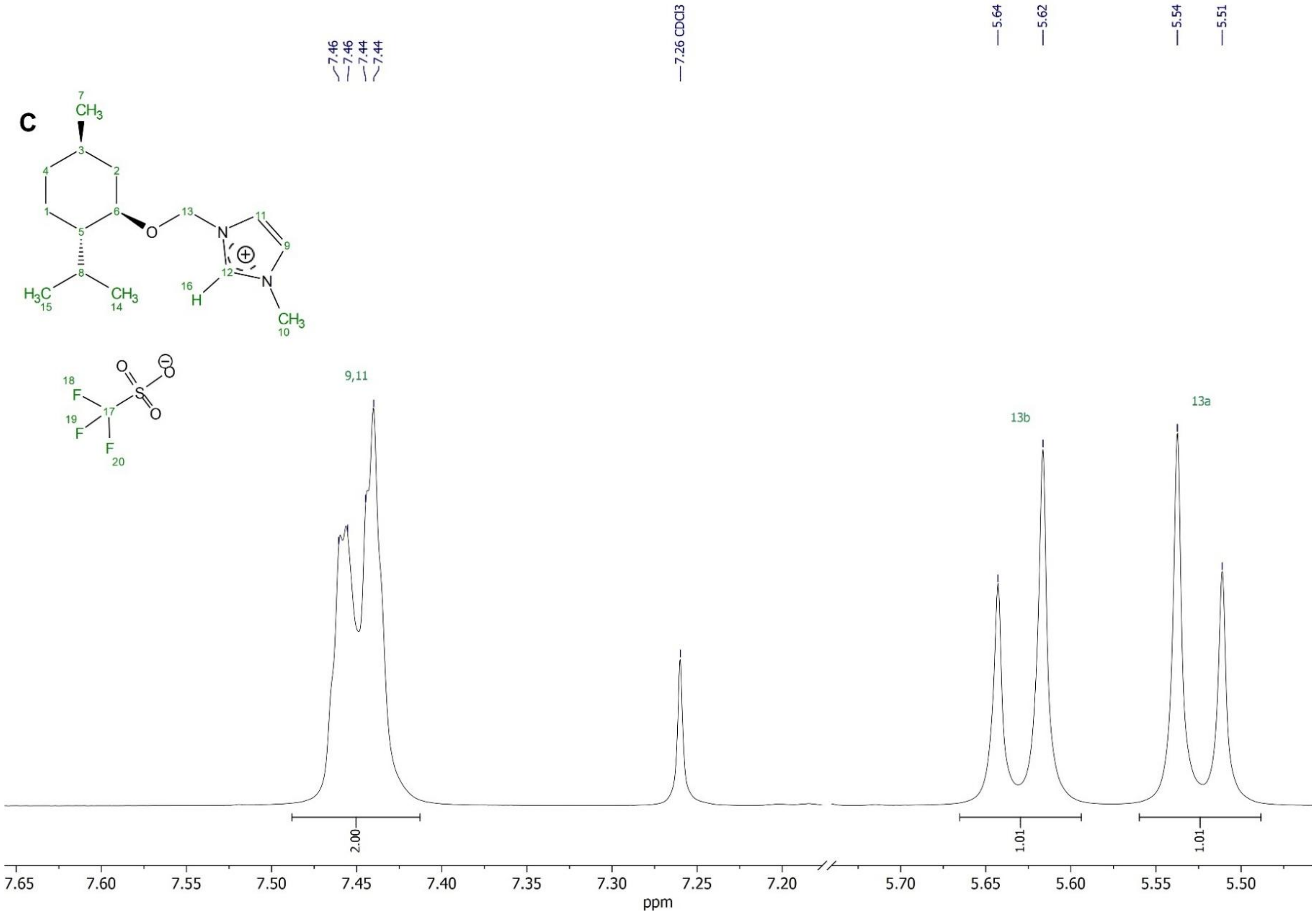

Figure S35. ^{1}H NMR (400 MHz) spectra of [Men-Im-C$_1$][OTF] (**4e**) in $CDCl_3$. **A.** region from 0.0 ppm to 12.0 ppm. **B.** region from 0.2 ppm to 3.4 ppm. **C.** region from 5.5 ppm to 7.6 ppm.

A

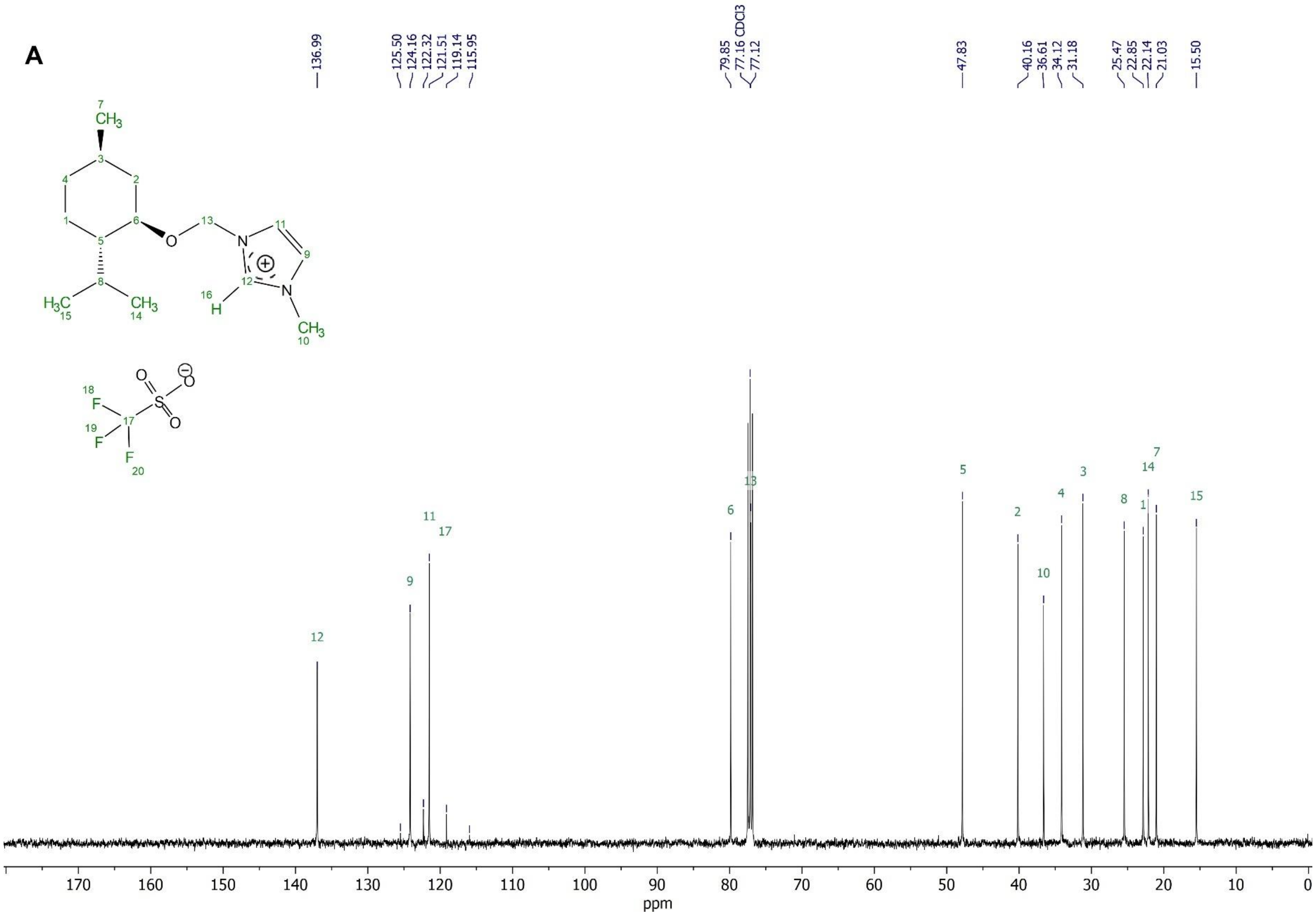

136.99
125.50
124.16
122.32
121.51
119.14
115.95
79.85
77.16 CDCl3
77.12
47.83
40.16
36.61
34.12
31.18
25.47
22.85
22.14
21.03
15.50
ppm

B

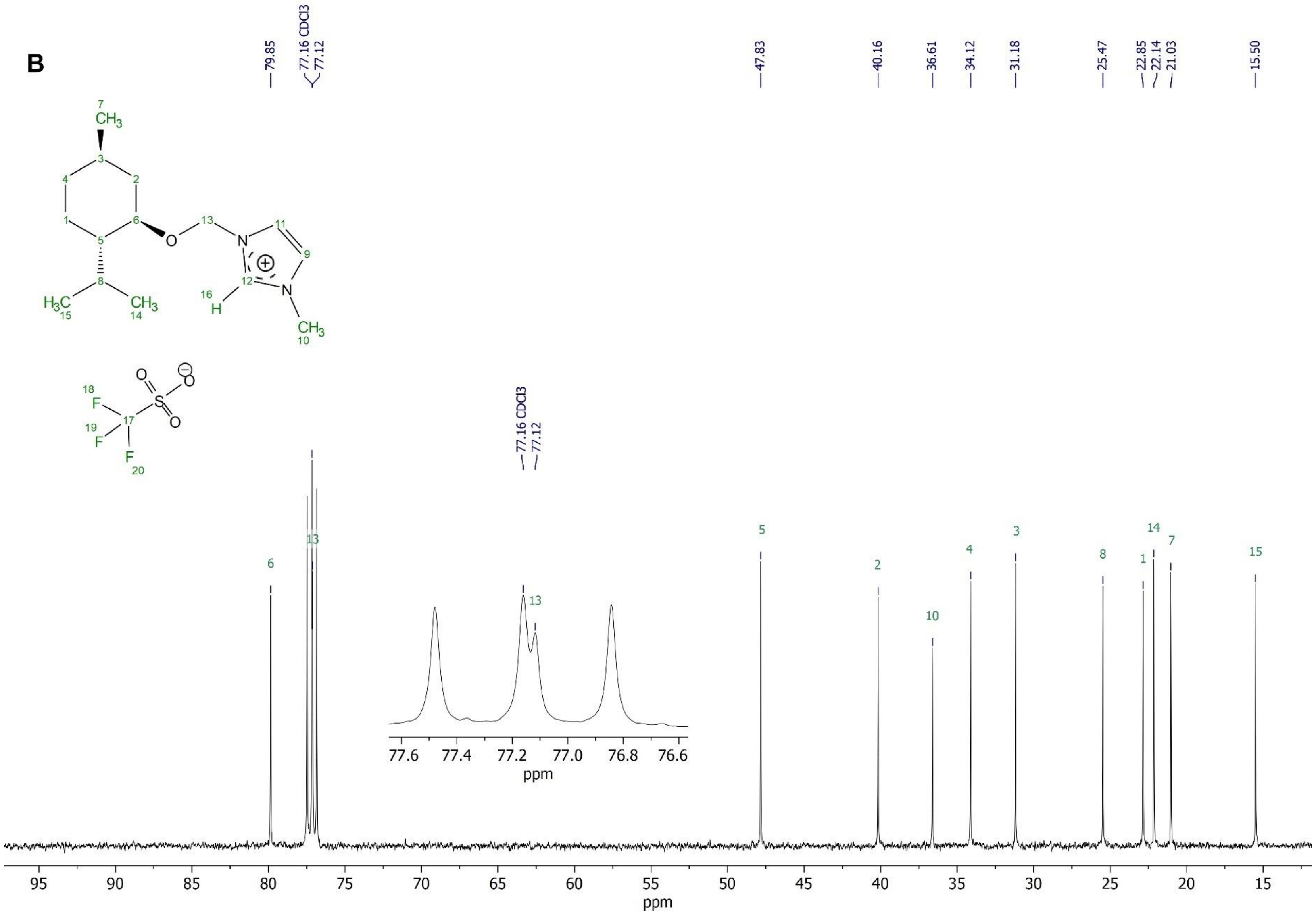
79.85
77.16 CDCl3
77.12
47.83
40.16
36.61
34.12
31.18
25.47
22.85
22.14
21.03
15.50
ppm

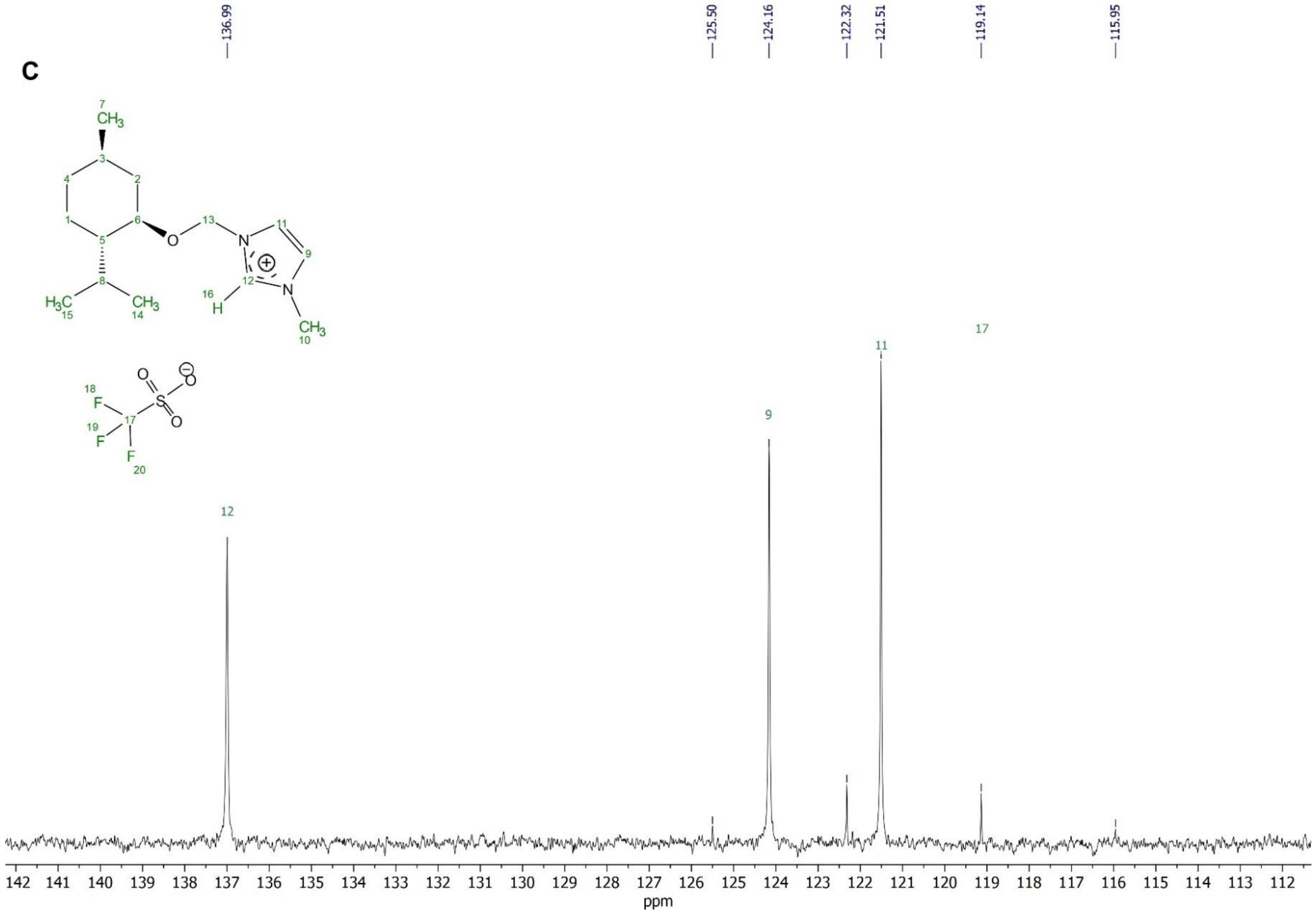

Figure S36. ^{13}C NMR (100 MHz) spectra of [Men-Im-C_1][OTF] (**4e**) in $CDCl_3$. **A.** region from 0.0 ppm to 170.0 ppm. **B.** region from 15.0 ppm to 95.0 ppm. **C.** region from 112.0 ppm to 142.0 ppm.

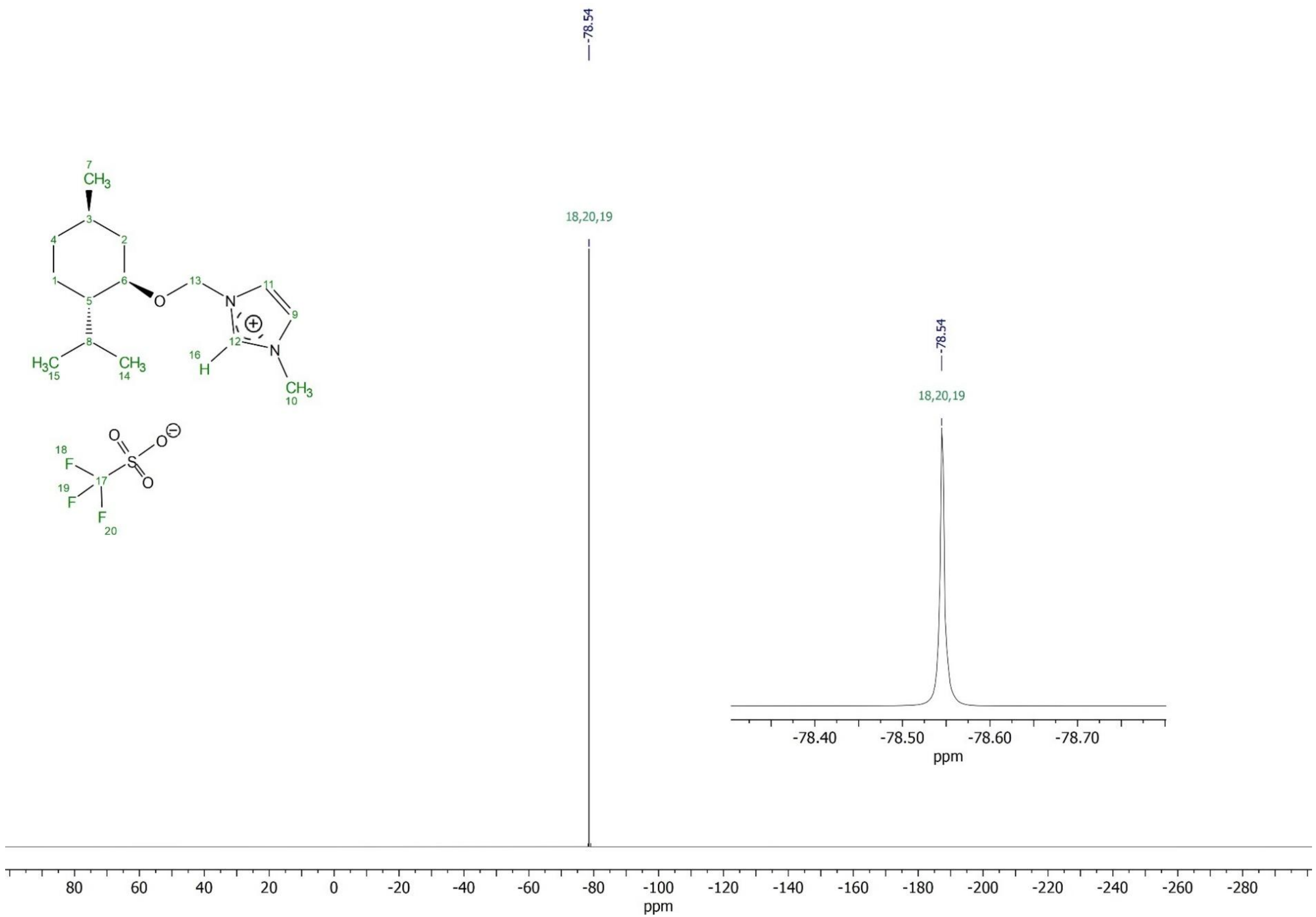

Figure S37. ^{19}F NMR (100 MHz) spectrum of [Men-Im-C_1][OTF] (**4e**) in $CDCl_3$.

A

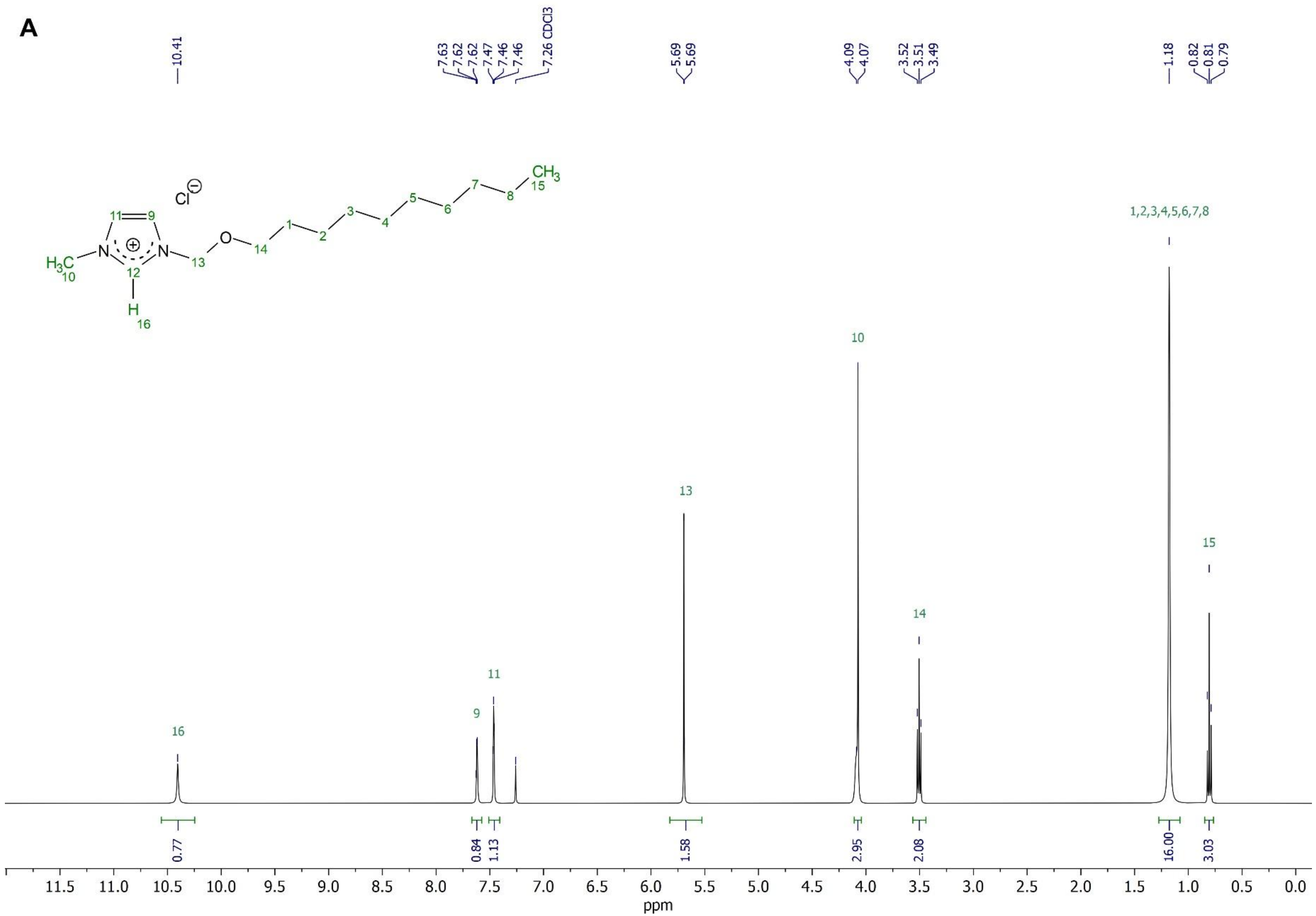

10.41
7.63
7.62
7.62
7.47
7.46
7.46
7.26 CDCl3
5.69
5.69
4.09
4.07
3.52
3.51
3.49
1.18
0.82
0.81
0.79
1,2,3,4,5,6,7,8
10
13
15
14
11
9
16
0.77
0.84
1.13
1.58
2.95
2.08
16.00
3.03
ppm

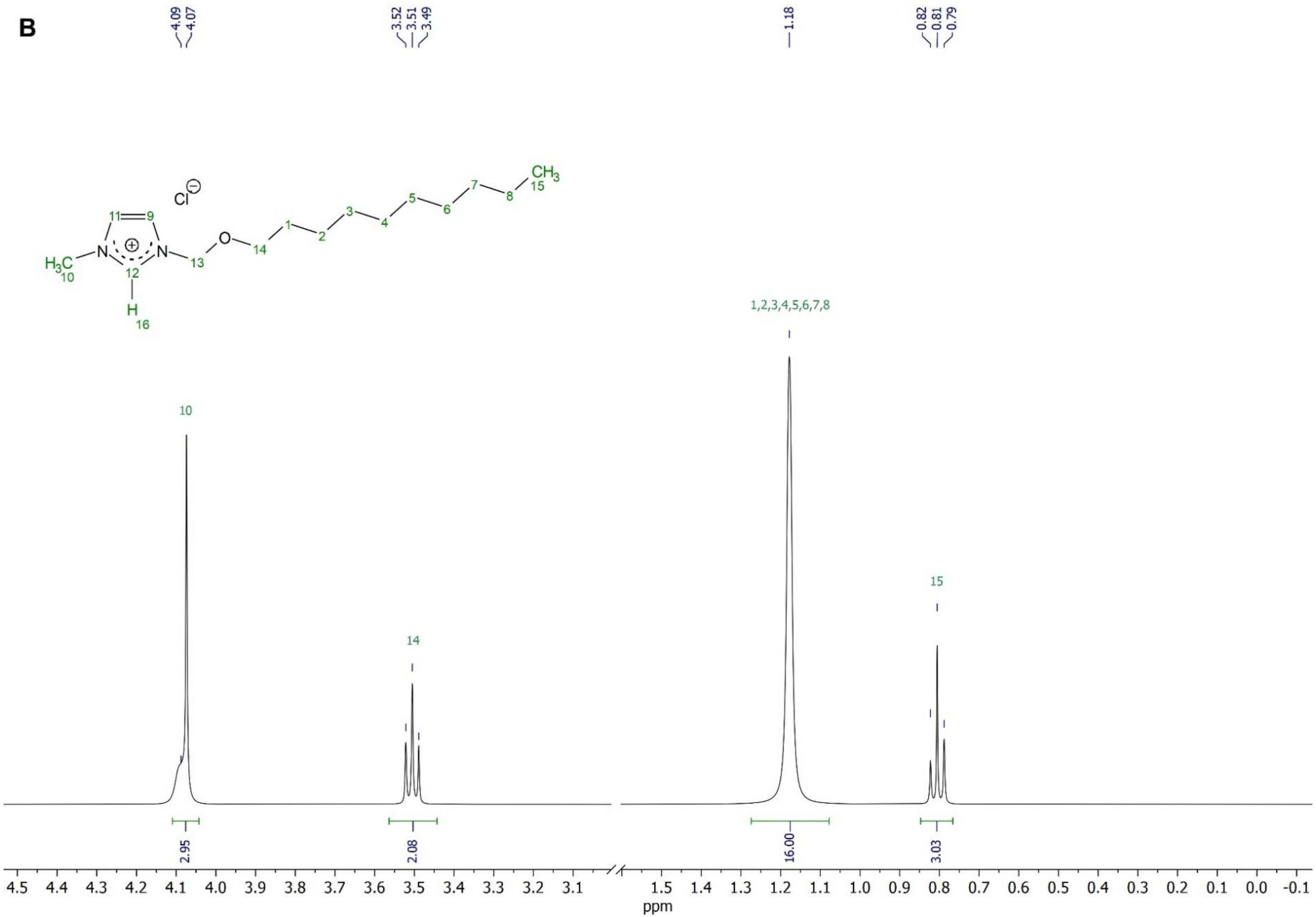

Figure S38. ^{1}H NMR (400 MHz) spectra of [C_{10}-Im-C_1][Cl] (**7**) in $CDCl_3$. **A.** region from 0.0 ppm to 12.0 ppm. **B.** region from 0.6 ppm to 4.5 ppm.

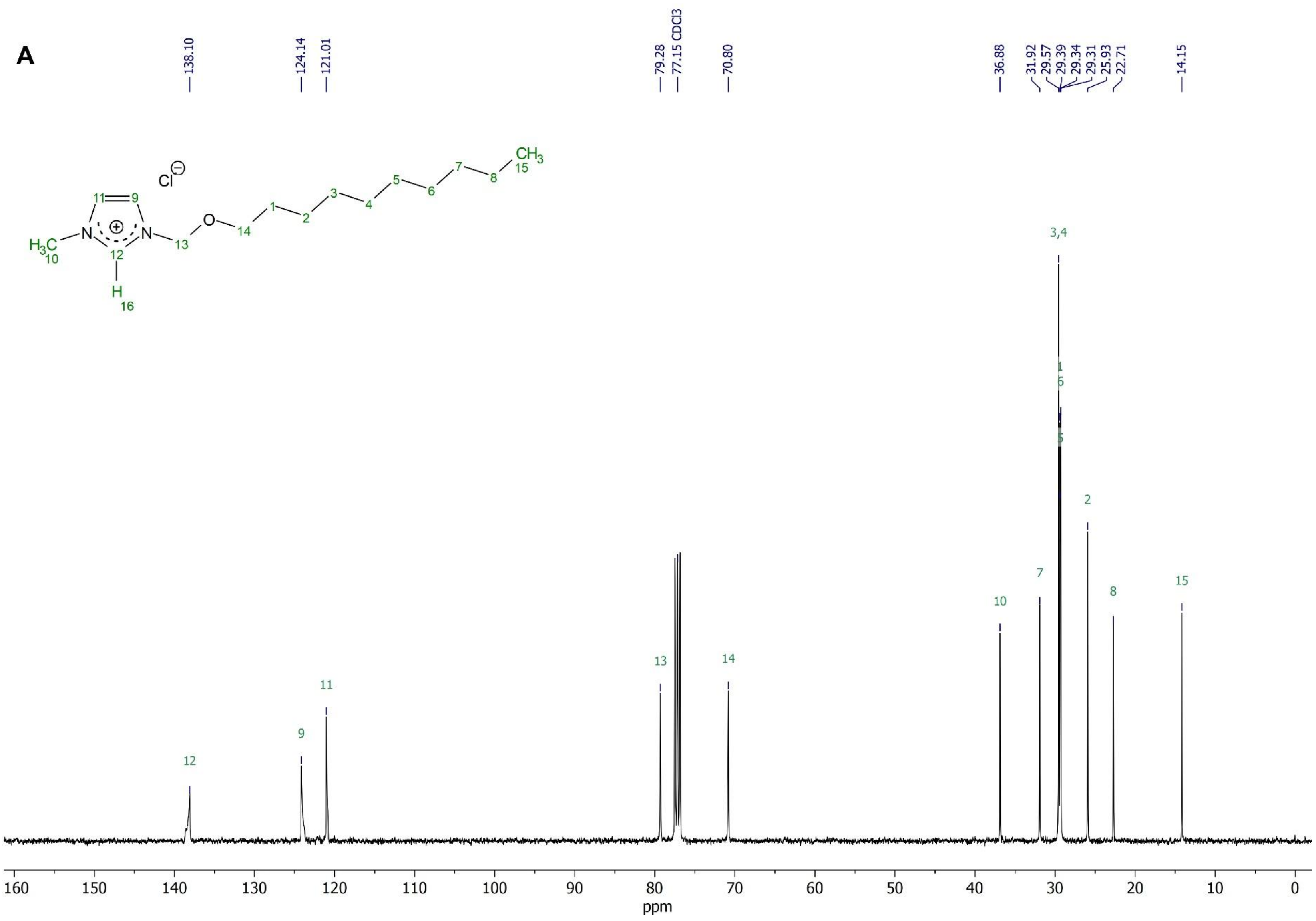
A
138.10
124.14
121.01
79.28
77.15 CDCl3
70.80
36.88
31.92
29.57
29.39
29.34
29.31
25.93
22.71
14.15
Cl
N
N
O
H3C
CH3
H
ppm

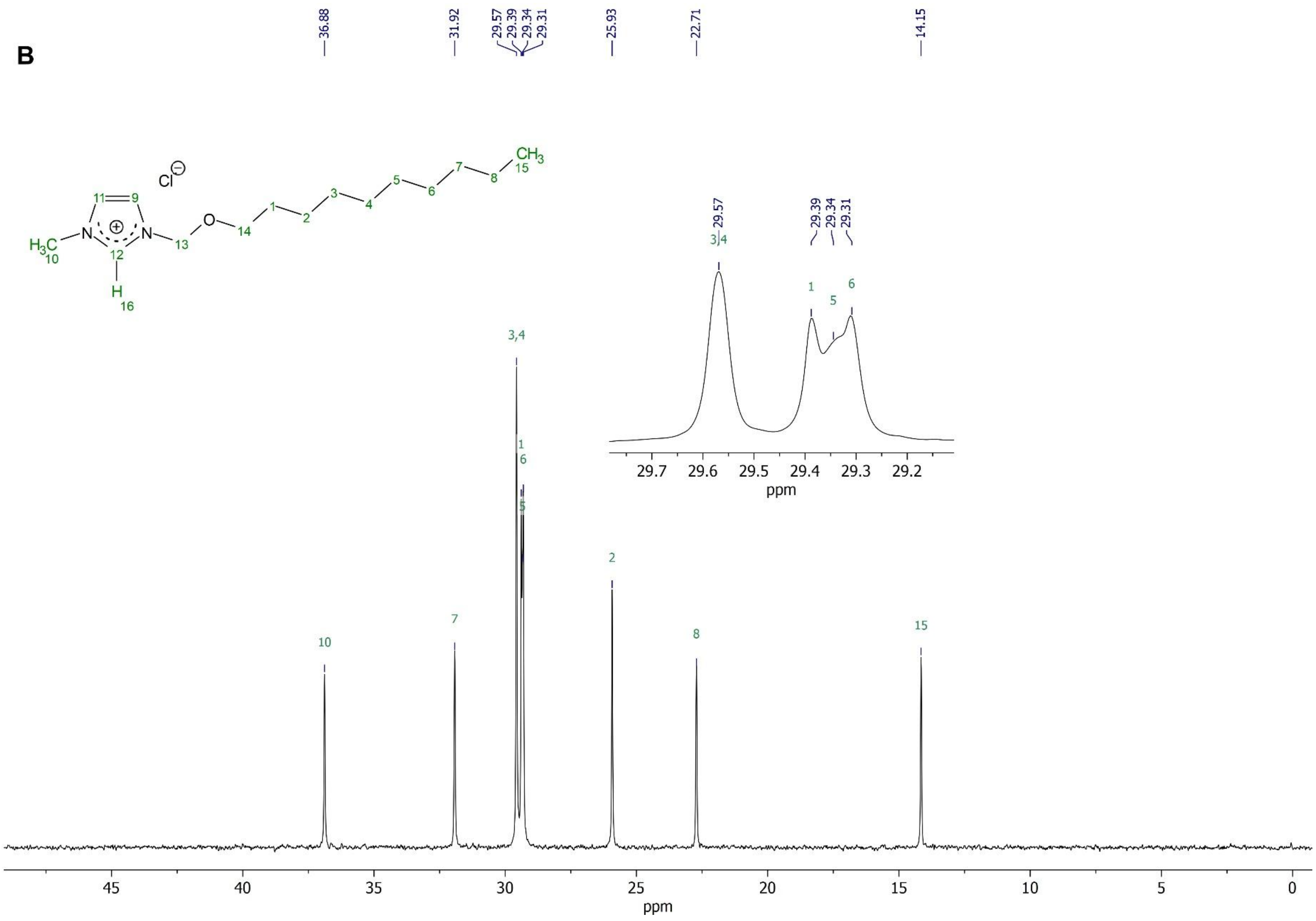

Figure S39. ^{13}C NMR (100 MHz) spectra of [C_{10}-Im-C_1][Cl] (**7**) in $CDCl_3$. **A.** region from 0.0 ppm to 170.0 ppm. **B.** region from 0.0 ppm to 50.0 ppm.

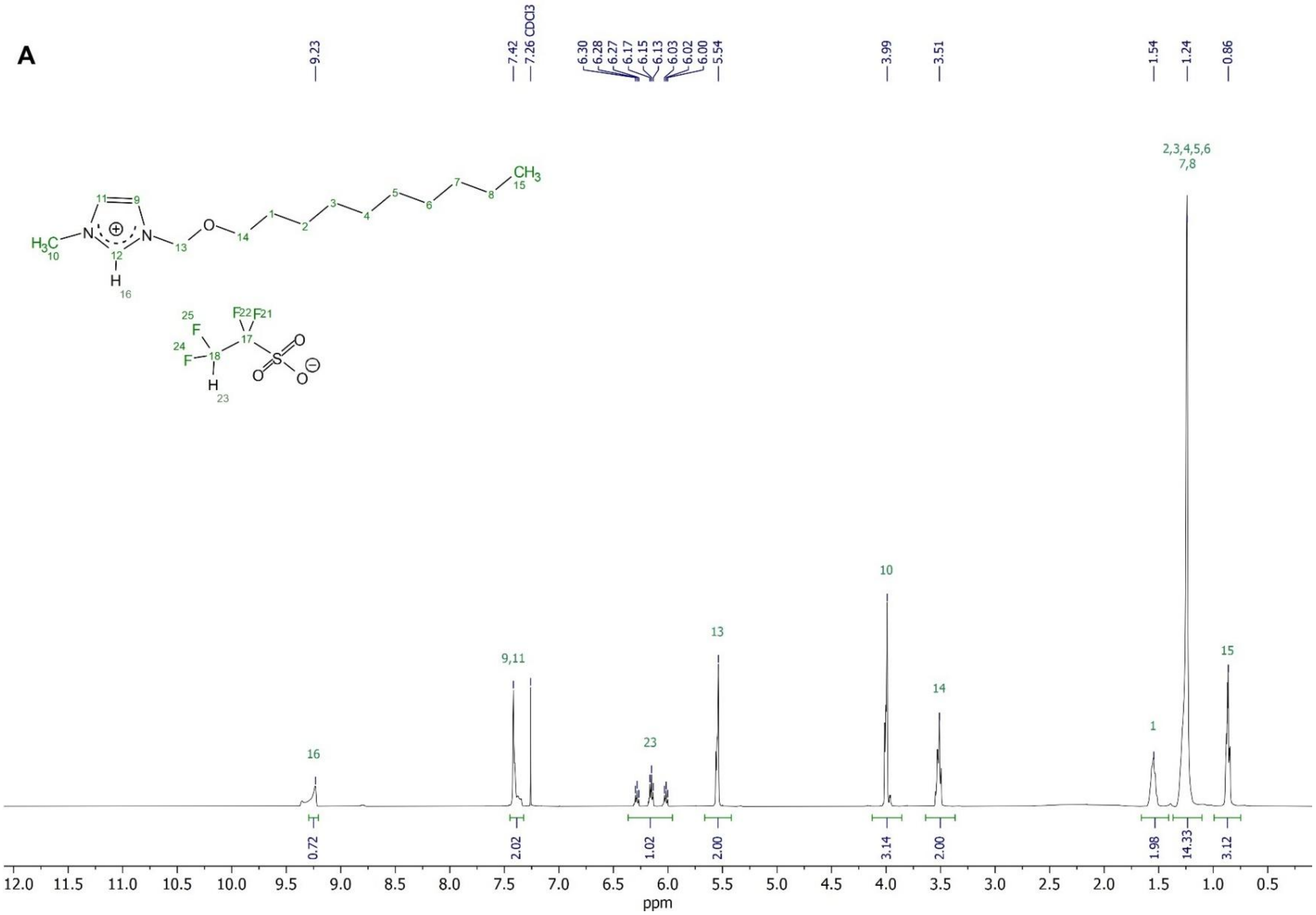
A
9.23
7.42
7.26 CDCl3
6.30
6.28
6.27
6.17
6.15
6.13
6.03
6.02
6.00
5.54
3.99
3.51
1.54
1.24
0.86
2,3,4,5,6
7,8
16
9,11
23
13
10
14
1
15
0.72
2.02
1.02
2.00
3.14
2.00
1.98
14.33
3.12
12.0 11.5 11.0 10.5 10.0 9.5 9.0 8.5 8.0 7.5 7.0 6.5 6.0 5.5 5.0 4.5 4.0 3.5 3.0 2.5 2.0 1.5 1.0 0.5
ppm

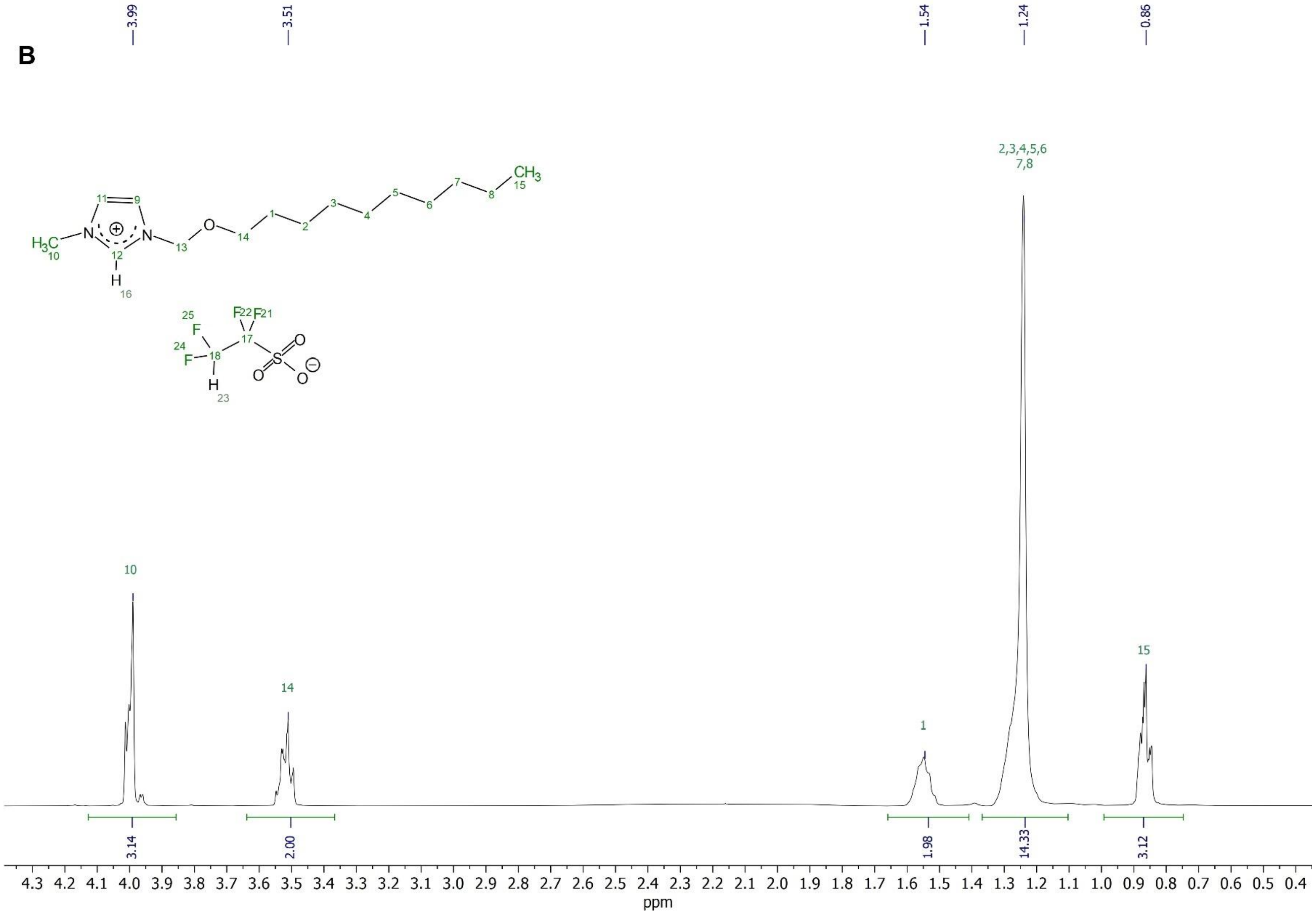
B
3.99
3.51
1.54
1.24
0.86
2,3,4,5,6
7,8
10
14
1
15
3.14
2.00
1.98
14.33
3.12
ppm

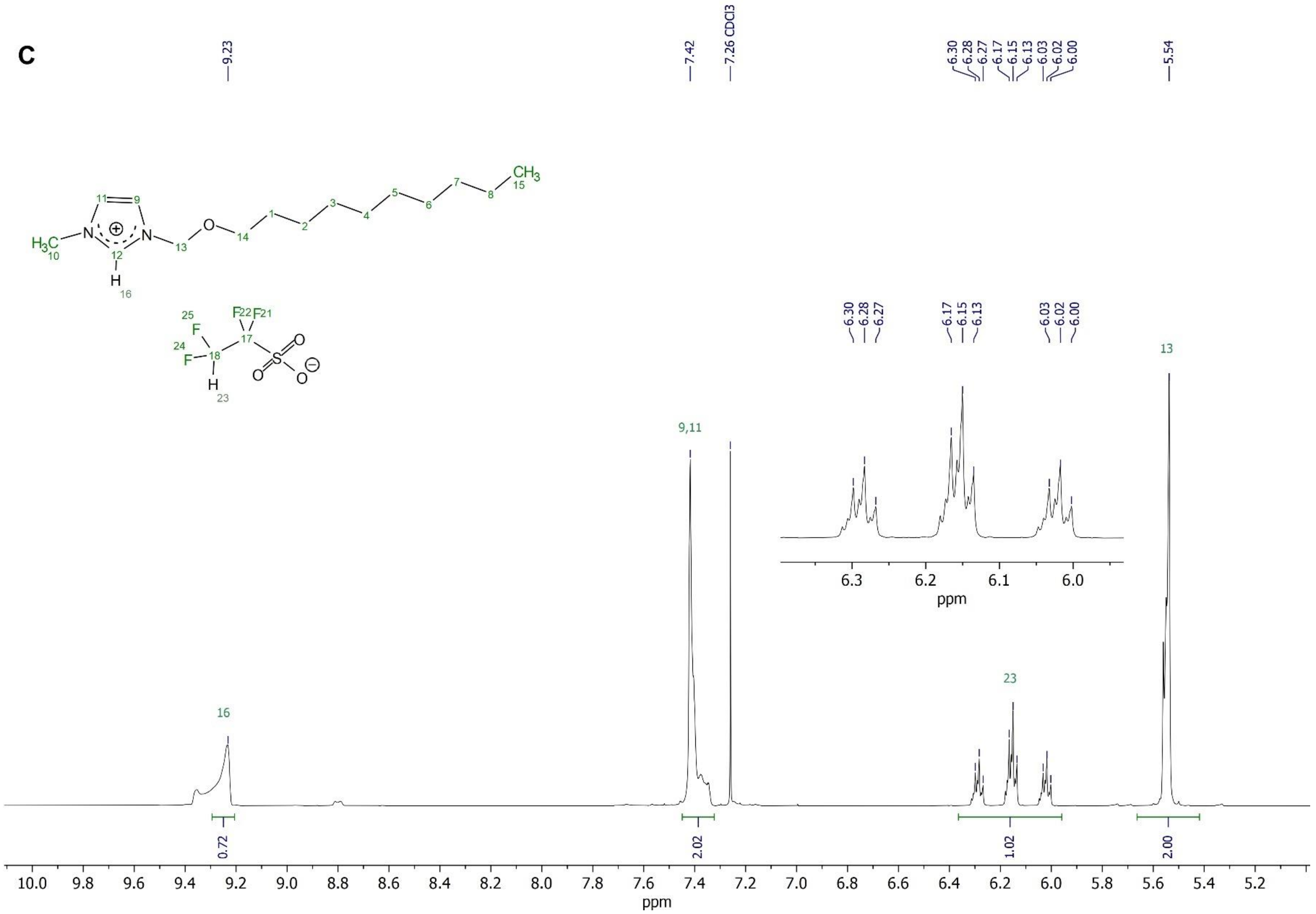

Figure S40. ^{1}H NMR (400 MHz) spectra of [C_{10}-Im-C_1][TFES] (**8a**) in $CDCl_3$. **A.** region from 0.0 ppm to 12.0 ppm. **B.** region from 0.4 ppm to 4.3 ppm. **C.** region from 5.2 ppm to 10.0 ppm.

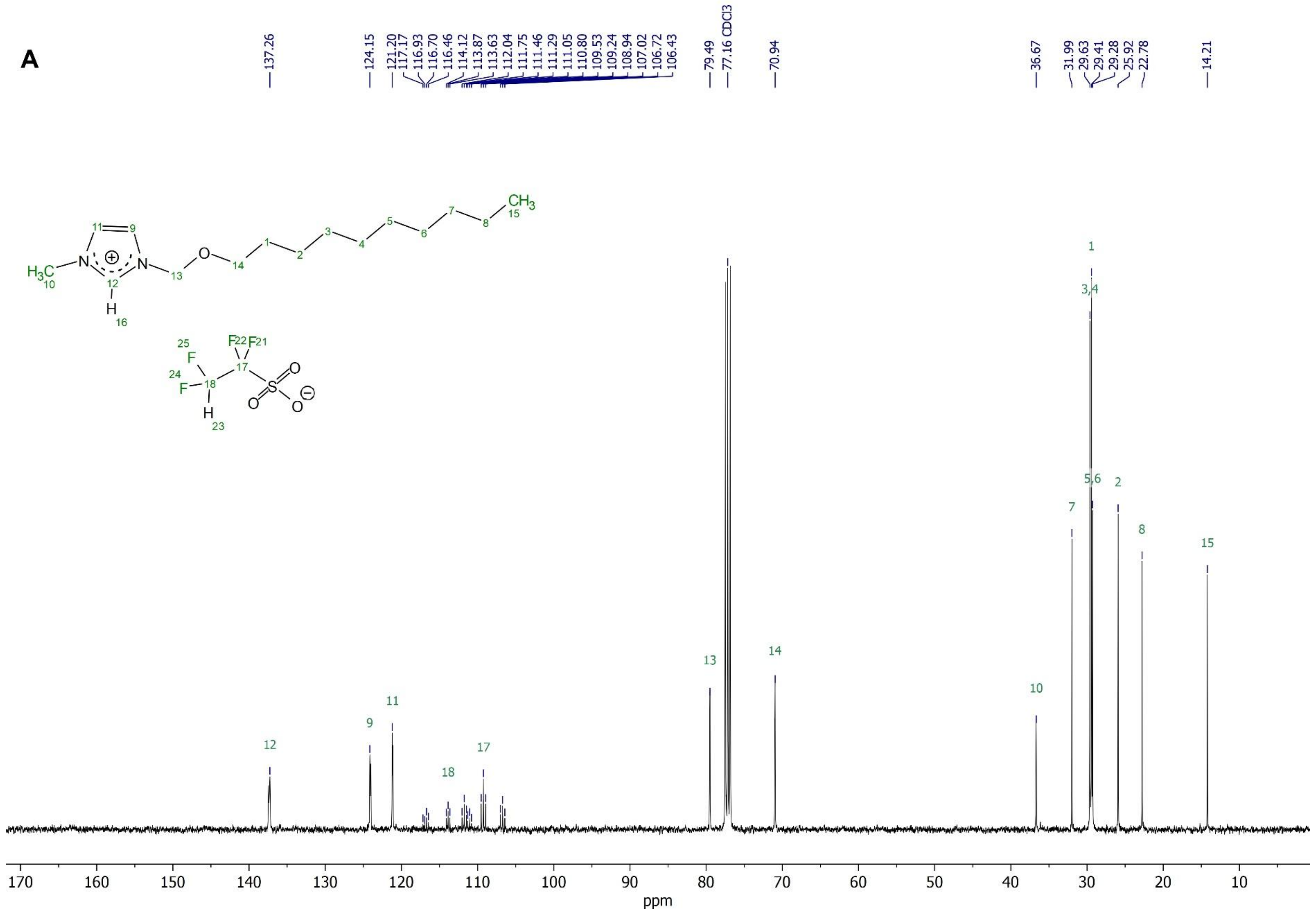

A
137.26
124.15
121.20
117.17
116.93
116.70
116.46
114.12
113.87
113.63
112.04
111.75
111.46
111.29
111.05
110.80
109.53
109.24
108.94
107.02
106.72
106.43
79.49
77.16 CDCl3
70.94
36.67
31.99
29.63
29.41
29.28
25.92
22.78
14.21
ppm

B

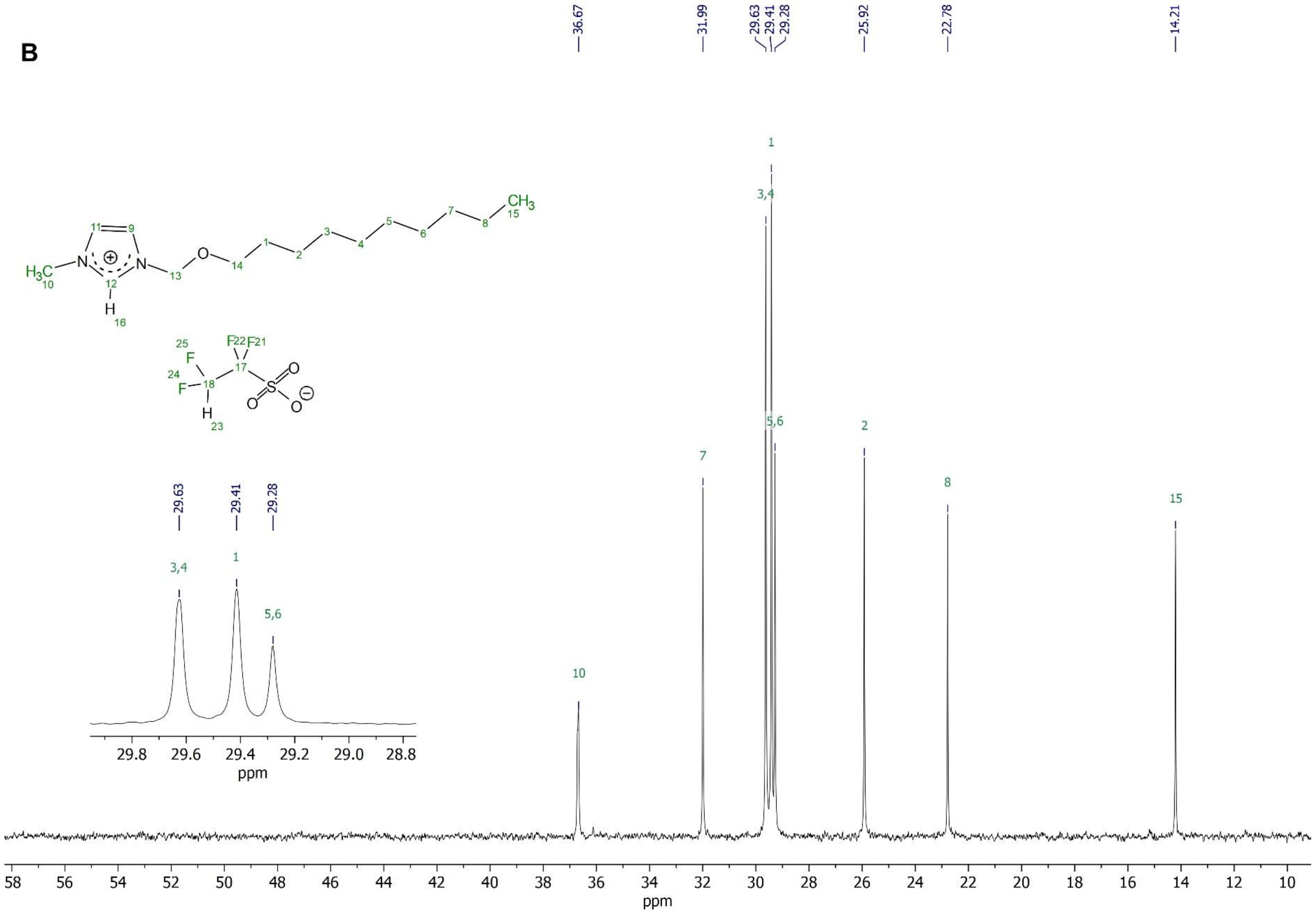
36.67
31.99
29.63
29.41
29.28
25.92
22.78
14.21
1
3,4
5,6
2
7
8
15
10
ppm
ppm

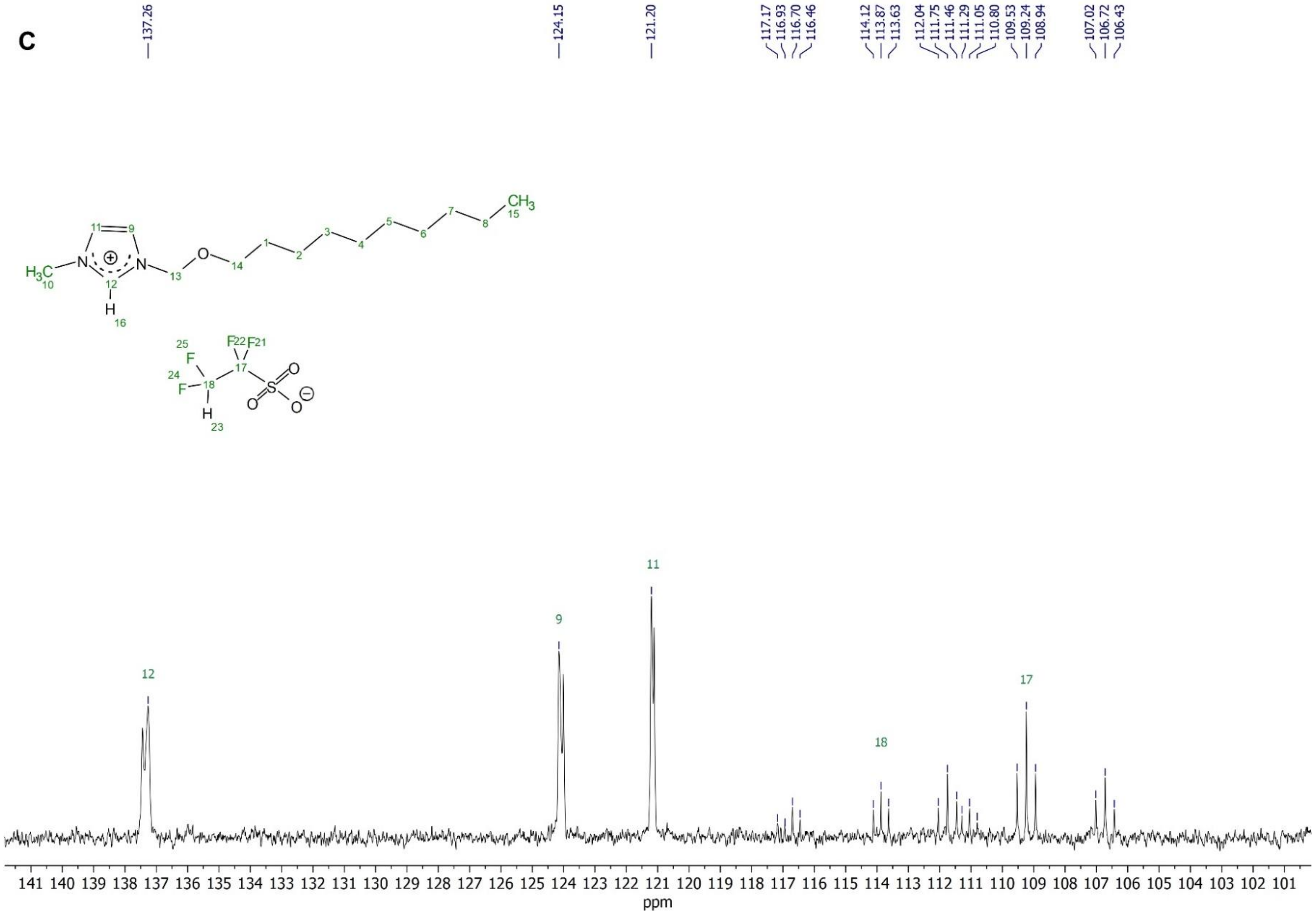

Figure S41. ^{13}C NMR (100 MHz) spectra of [C_{10}-Im-C_1][TFES] (**8a**) in $CDCl_3$. **A.** region from 0.0 ppm to 170.0 ppm. **B.** region from 10.0 ppm to 58.0 ppm. **C.** region from 101.0 ppm to 141.0 ppm.

A

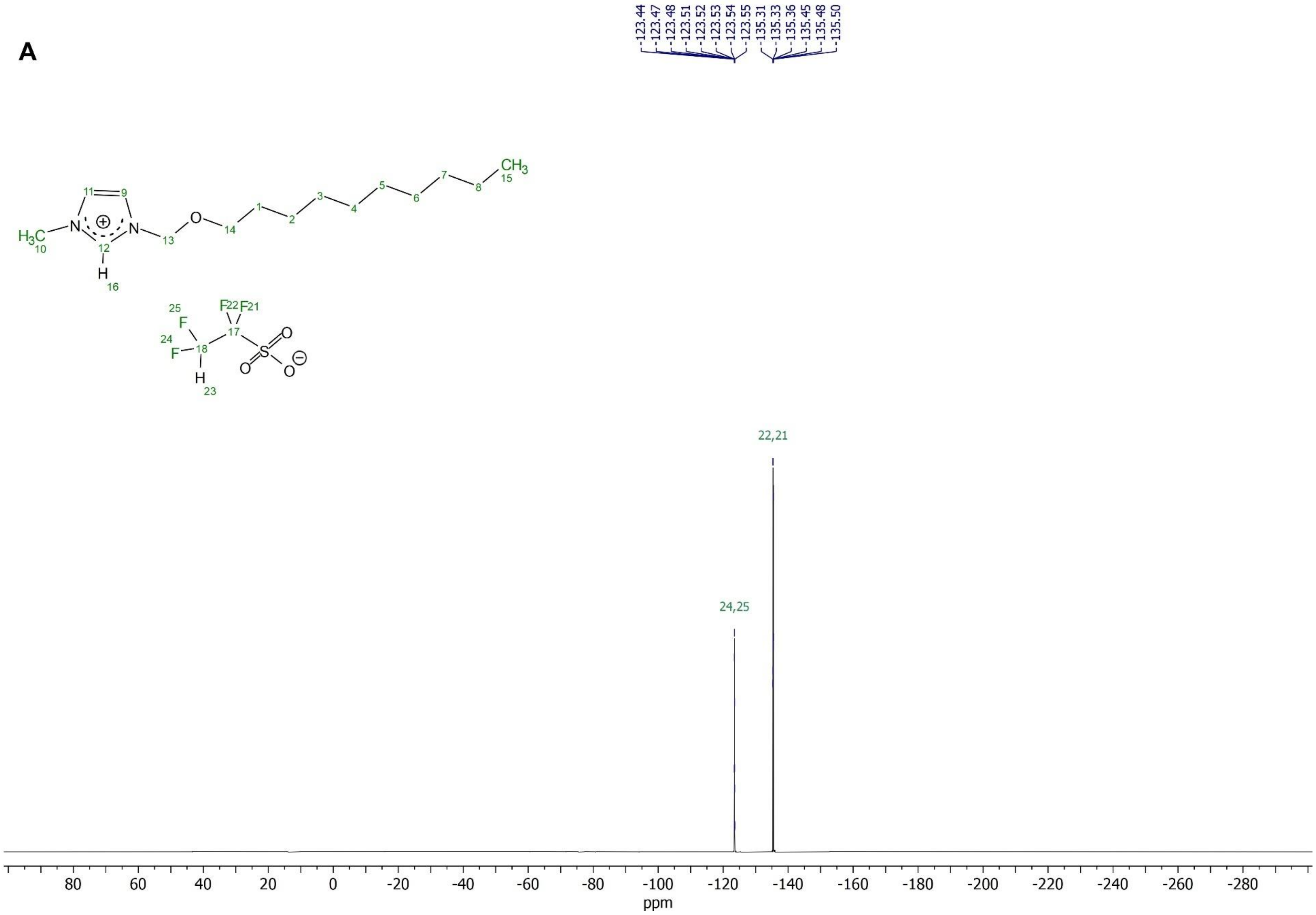

-123.44
-123.47
-123.48
-123.51
-123.52
-123.53
-123.54
-123.55
-135.31
-135.33
-135.36
-135.45
-135.48
-135.50
22,21
24,25
80
60
40
20
0
-20
-40
-60
-80
-100
-120
-140
-160
-180
-200
-220
-240
-260
-280
ppm

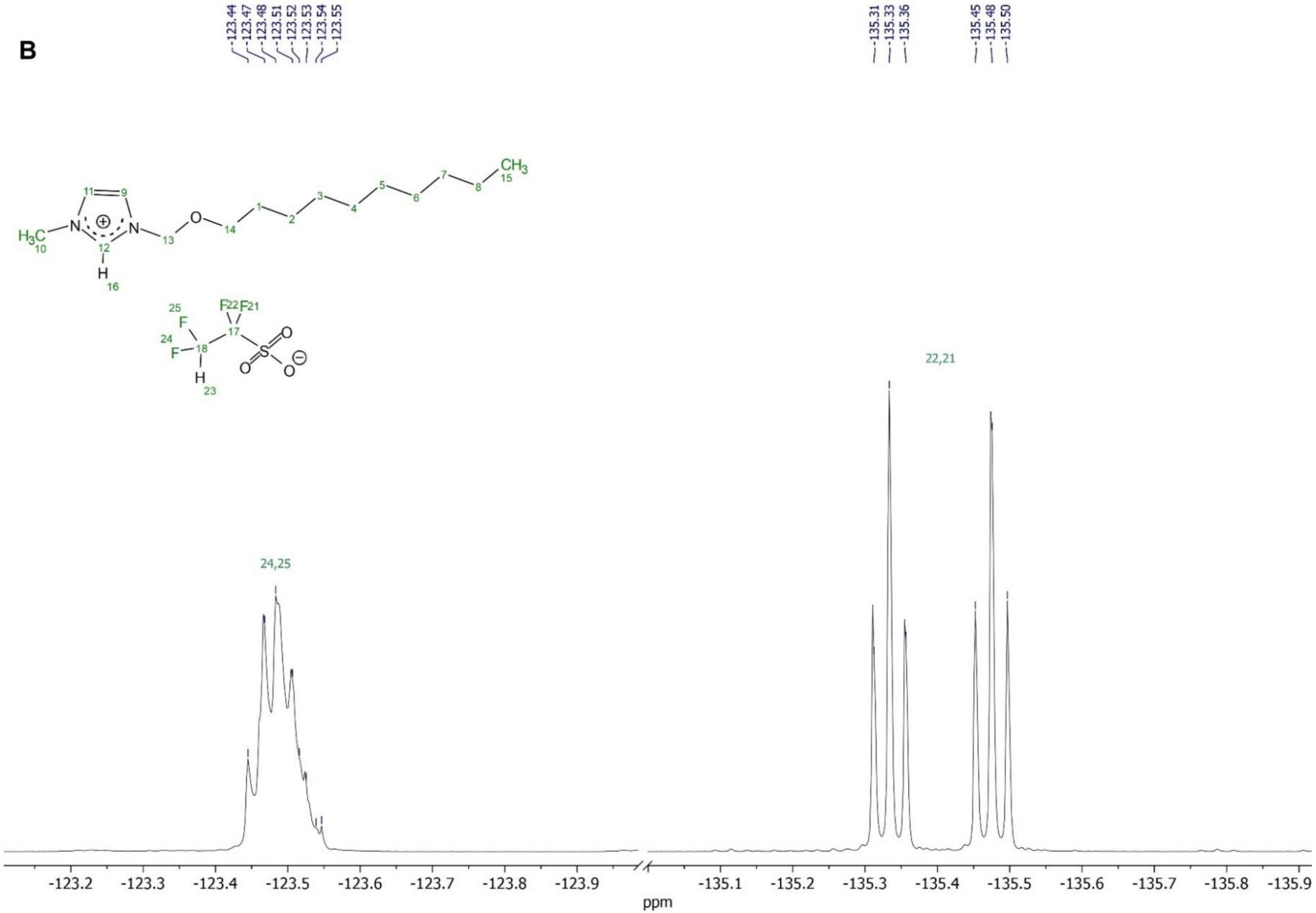

Figure S42. ^{19}F NMR (100 MHz) spectra of [C_{10}-Im-C_1][TFES] (**8a**) in $CDCl_3$. **A.** region from -280.0 ppm to 90.0 ppm. **B.** region from -135.9 ppm to -123.2 ppm.

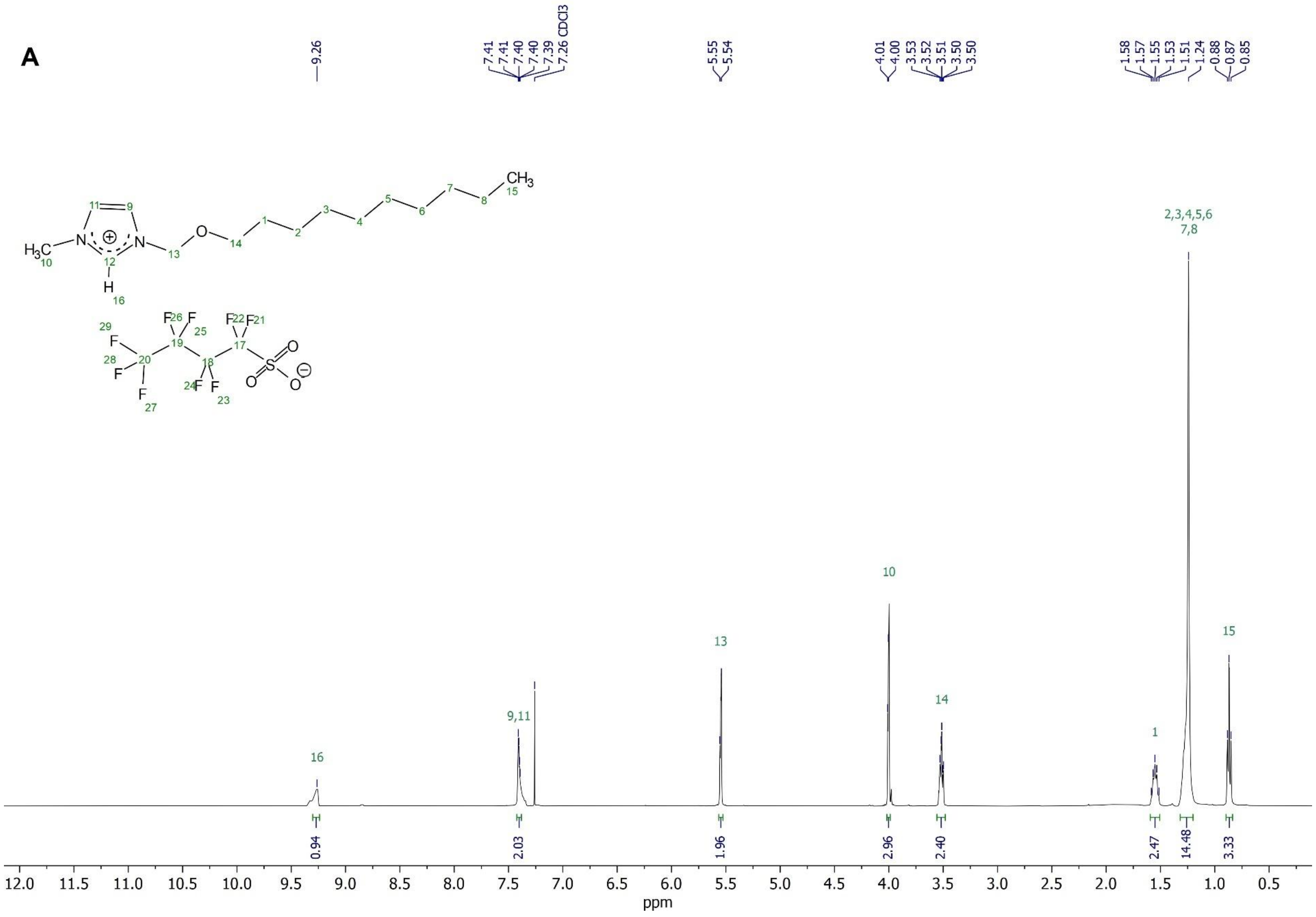
A
9.26
7.41
7.41
7.40
7.40
7.39
7.26 CDCl3
5.55
5.54
4.01
4.00
3.53
3.52
3.51
3.50
3.50
1.58
1.57
1.55
1.53
1.51
1.24
0.88
0.87
0.85
2,3,4,5,6
7,8
10
13
15
14
9,11
16
1
0.94
2.03
1.96
2.96
2.40
2.47
14.48
3.33
12.0 11.5 11.0 10.5 10.0 9.5 9.0 8.5 8.0 7.5 7.0 6.5 6.0 5.5 5.0 4.5 4.0 3.5 3.0 2.5 2.0 1.5 1.0 0.5
ppm

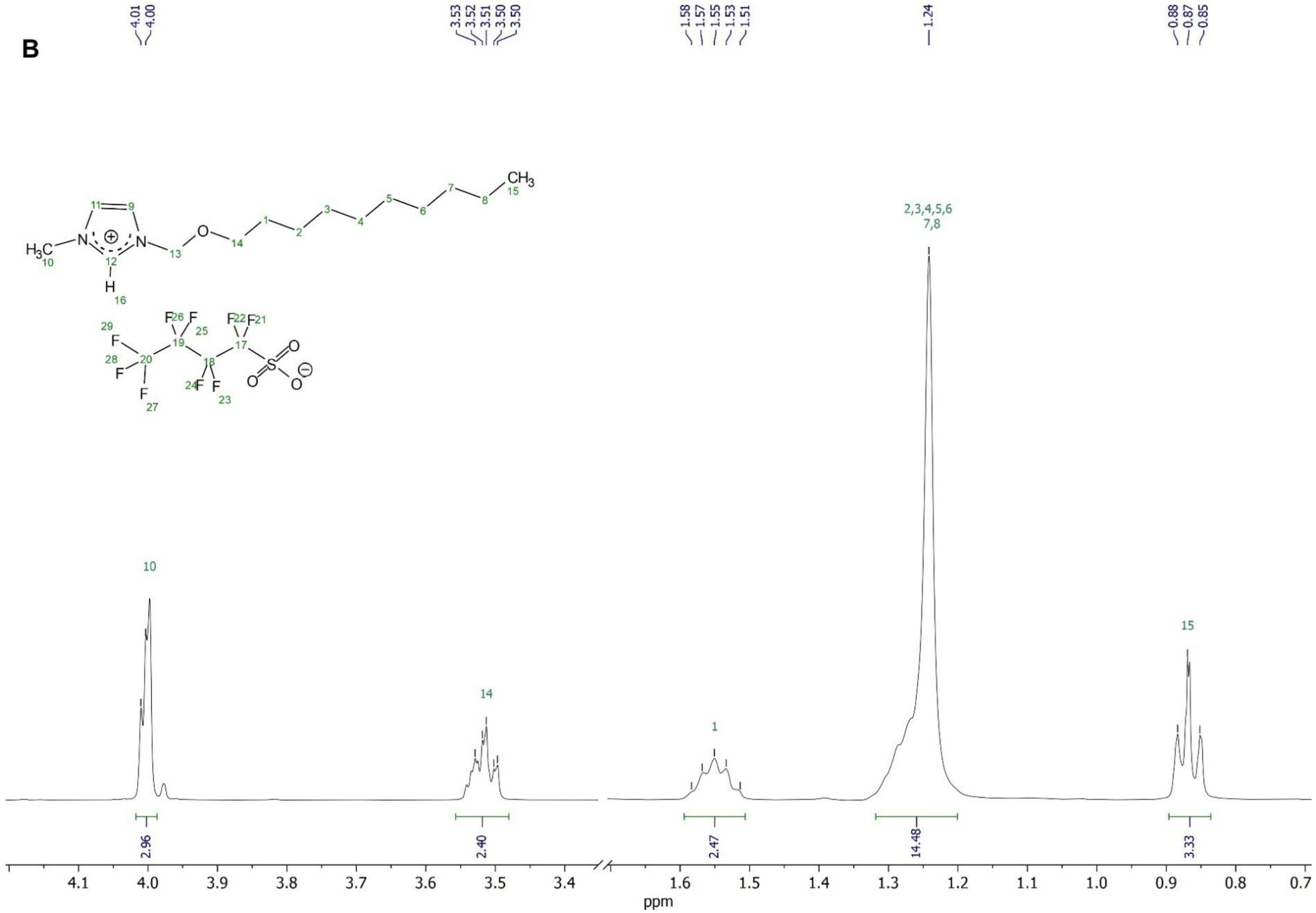

Figure S43. ^{1}H NMR (400 MHz) spectra of [C$_{10}$-Im-C$_{1}$][PFBS] (**8b**) in $CDCl_3$. **A.** region from 0.0 ppm to 12.0 ppm. **B.** region from 0.7 ppm to 4.2 ppm.

A

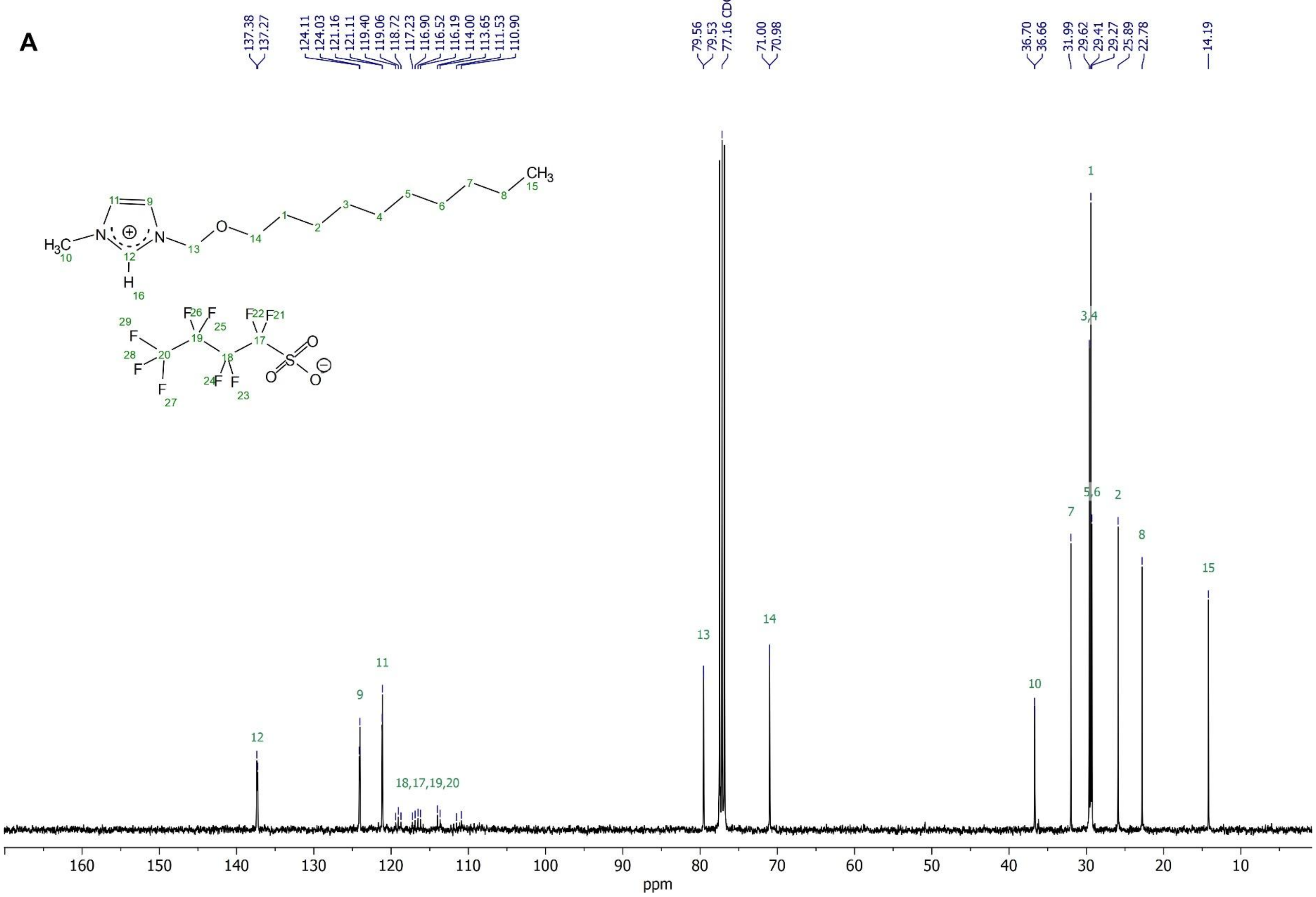

137.38
137.27
124.11
124.03
121.16
121.11
119.40
119.06
118.72
117.23
116.90
116.52
116.19
114.00
113.65
111.53
110.90
79.56
79.53
77.16 CDCl3
71.00
70.98
36.70
36.66
31.99
29.62
29.41
29.27
25.89
22.78
14.19
12
9
11
18,17,19,20
13
14
10
7
5,6
2
8
15
3,4
1
ppm

B

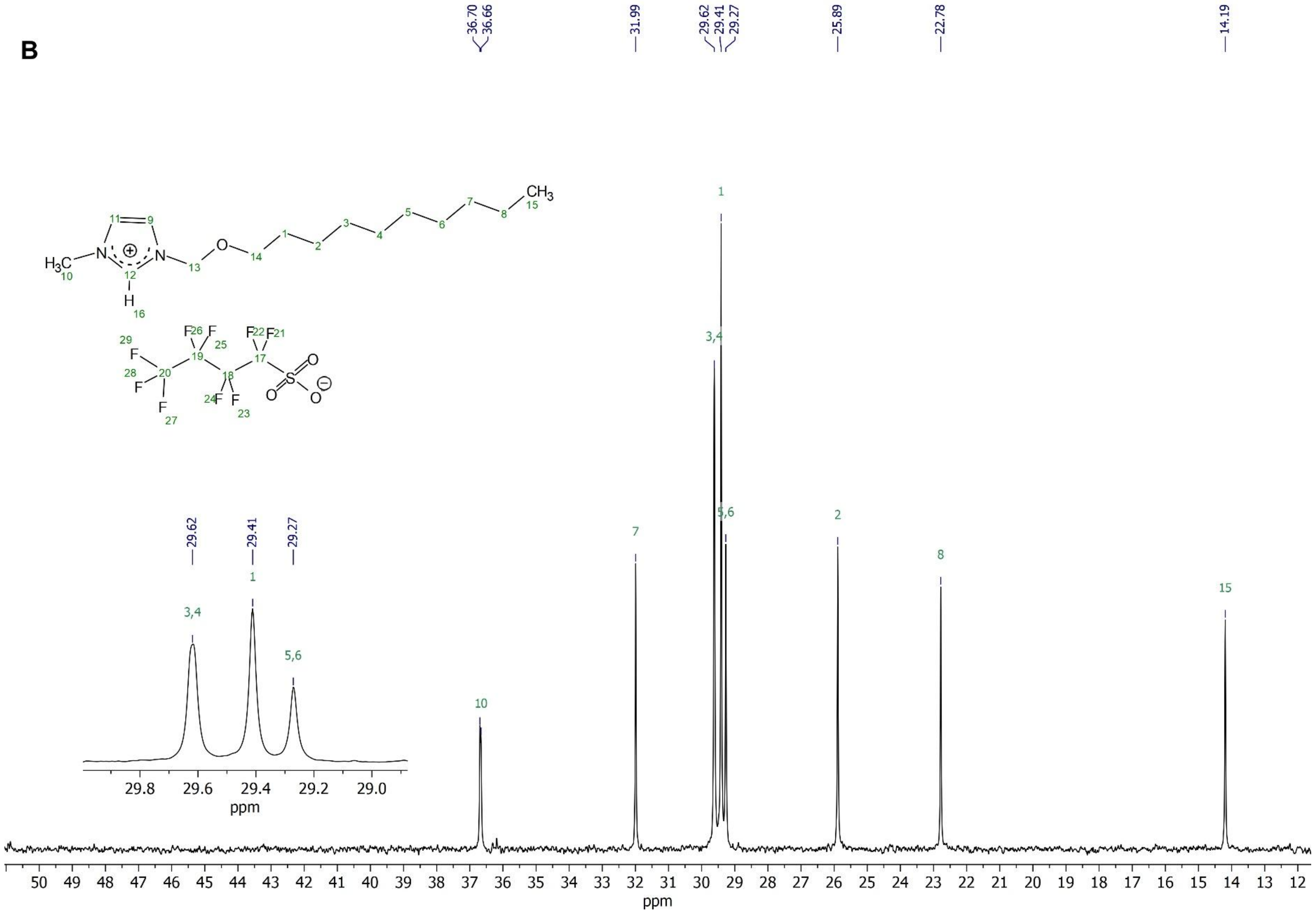

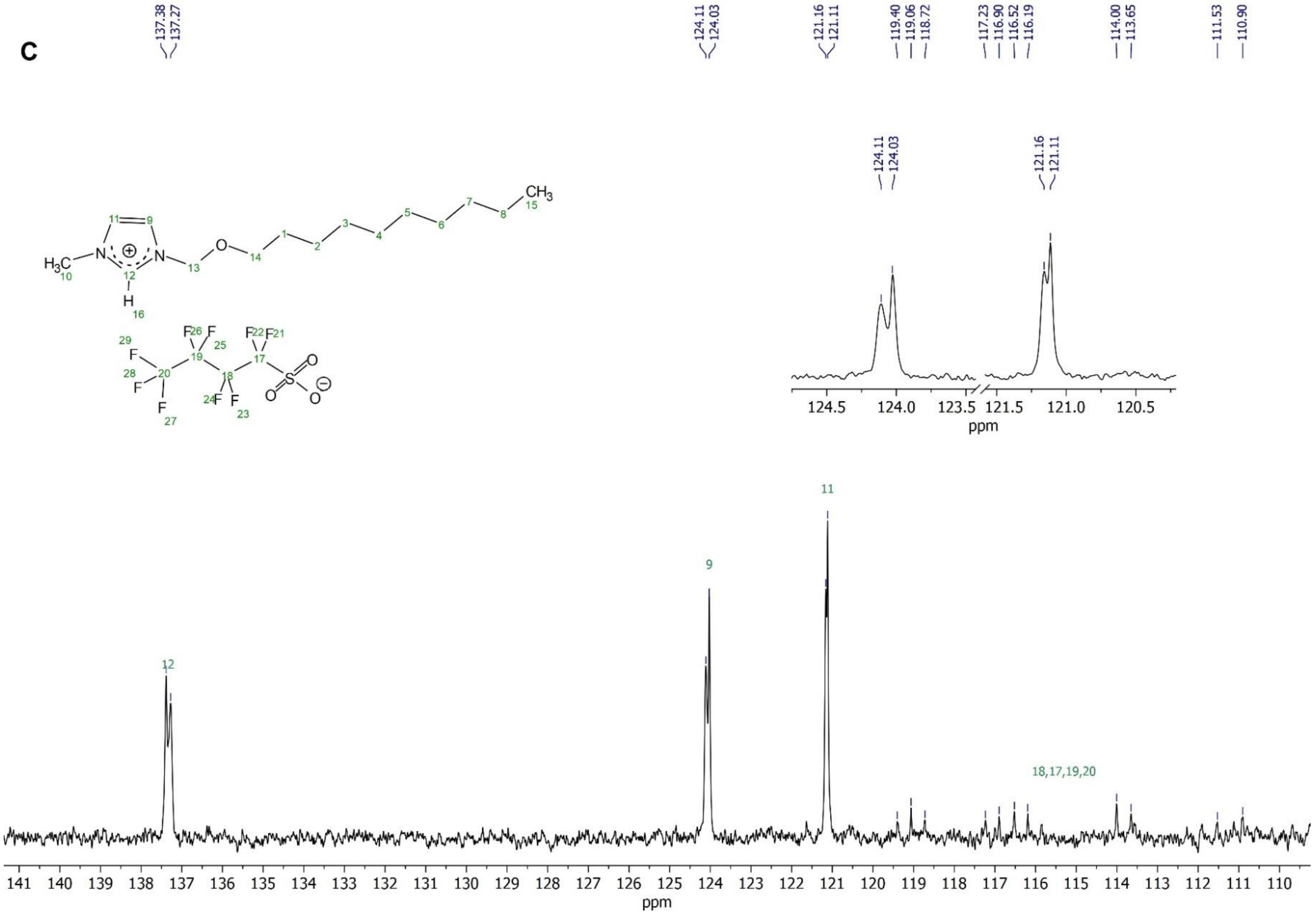

Figure S44. ^{13}C NMR (100 MHz) spectra of [C_{10}-Im-C_1][PFBS] (**8b**) in $CDCl_3$. **A.** region from 0.0 ppm to 170.0 ppm. **B.** region from 12.0 ppm to 51.0 ppm. **C.** region from 110.0 ppm to 141.0 ppm.

A

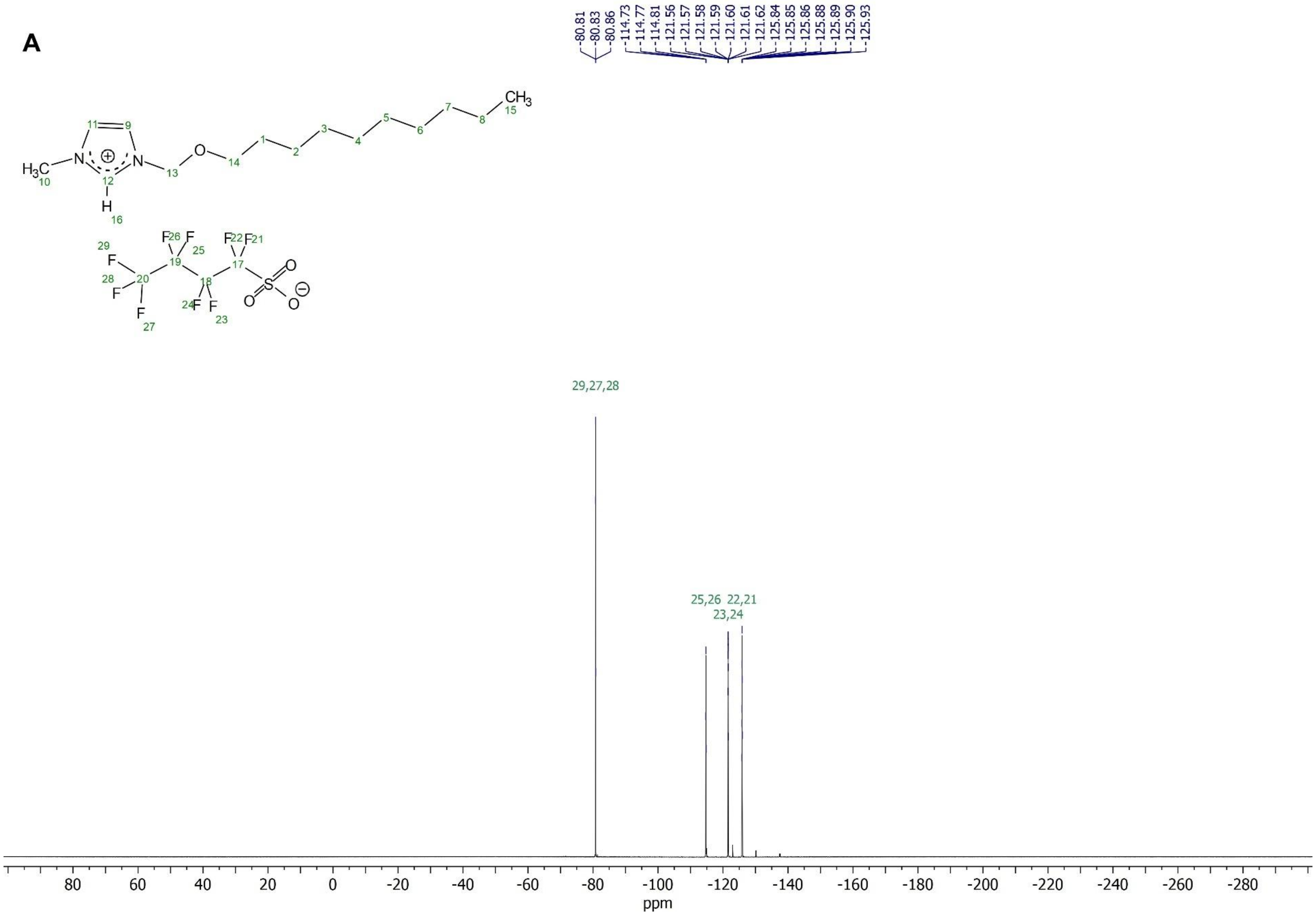
-80.81
-80.83
-80.86
-114.73
-114.77
-114.81
-121.56
-121.57
-121.58
-121.59
-121.60
-121.61
-121.62
-125.84
-125.85
-125.86
-125.88
-125.89
-125.90
-125.93
29,27,28
25,26
23,24
22,21
80
60
40
20
0
-20
-40
-60
-80
-100
-120
-140
-160
-180
-200
-220
-240
-260
-280
ppm

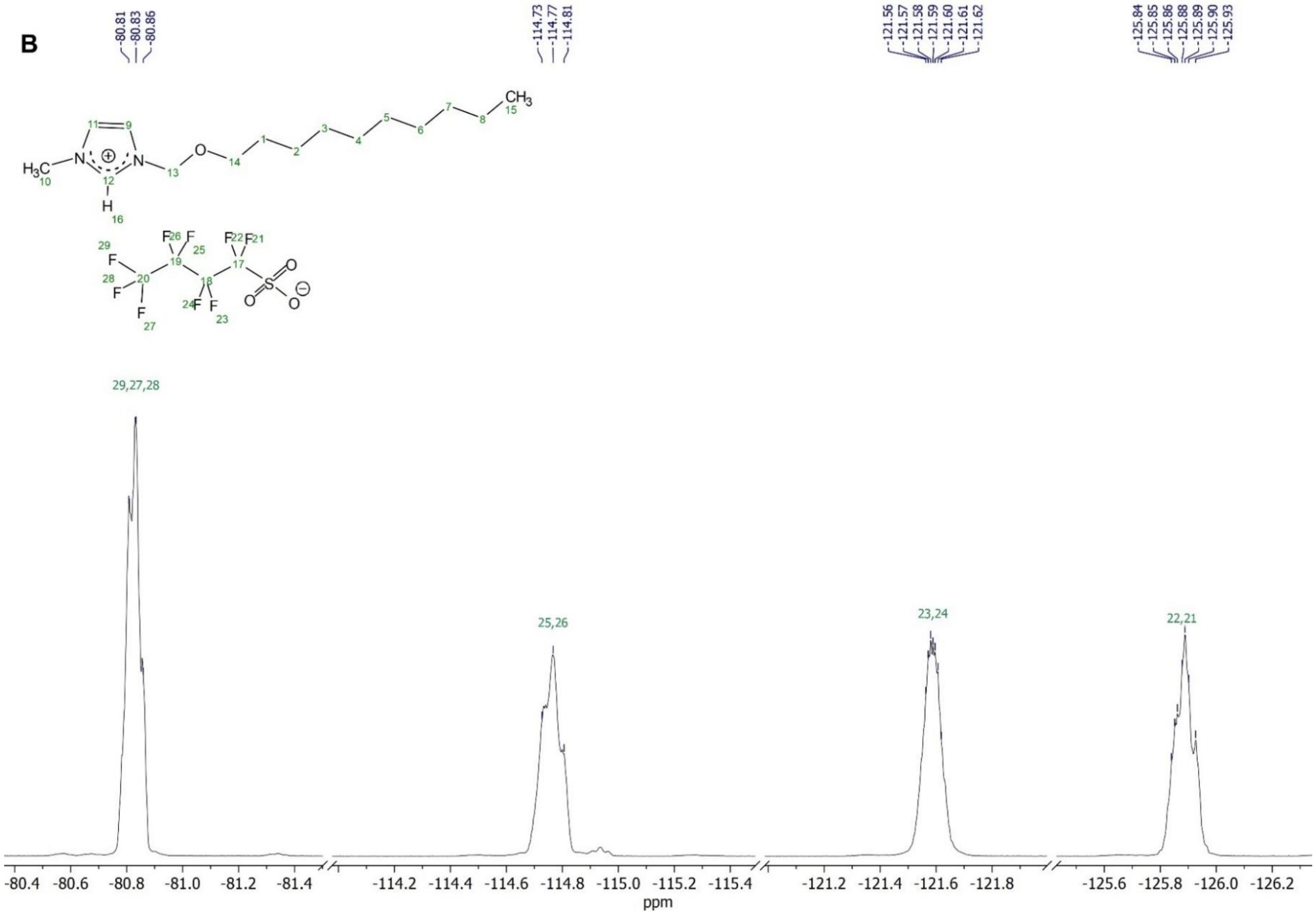

Figure S45. ^{19}F NMR (100 MHz) spectrum of [C_{10}-Im-C_1][PFBS] (**8b**) in $CDCl_3$. **A.** region from -280.0 ppm to 90.0 ppm. **B.** region from -126.0 ppm to -80.4 ppm.

Cation moiety-dependent shifts in ^{1}H NMR spectra of studied of 1-[(1*R*,2*S*,5*R*)-(–)-menthoxymethyl]-3-methylimidazolium [Men-Im-C_1][X] salts (3, 4a–4e) and 1-decyloxymethyl-3-methylimidazolium [C_{10}-Im-C_1][X] (7, 8a, 8b) salts

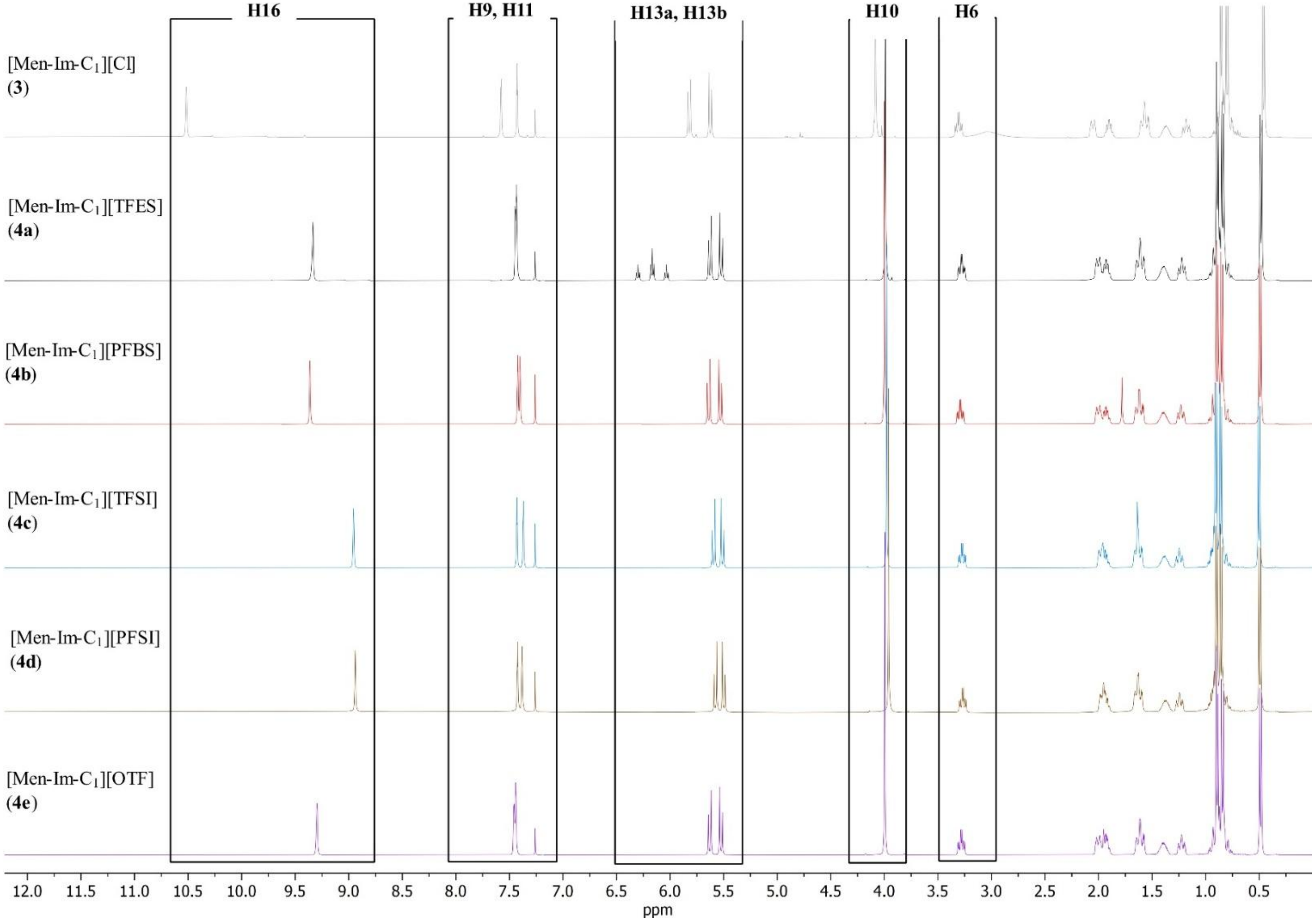

Figure S46. Stacked ^{1}H NMR spectra of functionalised ionic liquids containing the (1*R*,2*S*,5*R*)-(–)-menthol moiety in cation, highlighting differences in the chemical shifts of selected protons.

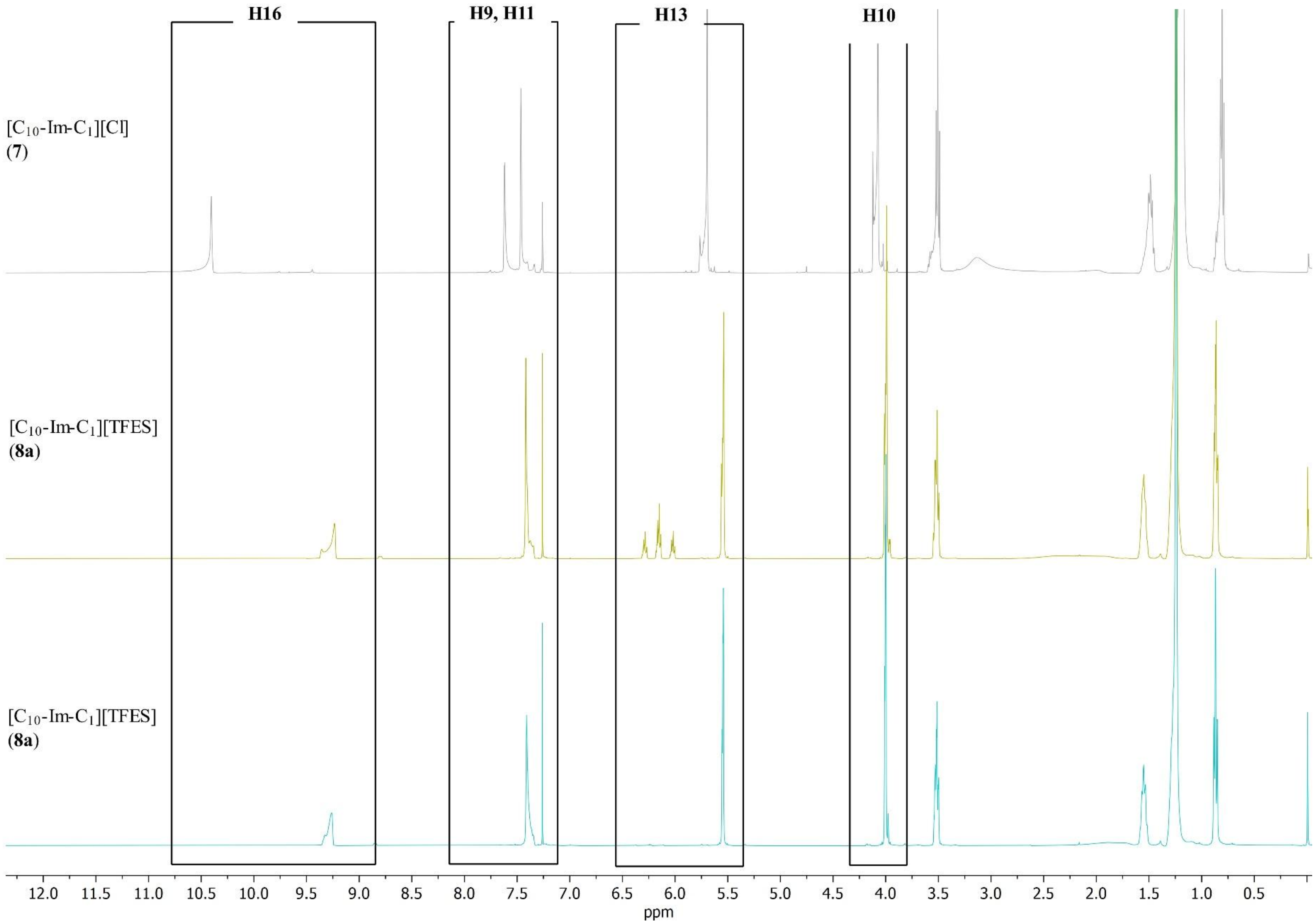

Figure S47. Stacked ^{1}H NMR spectra of functionalised ionic liquids containing the 1-decanol moiety in cation, highlighting differences in the chemical shifts of selected protons.

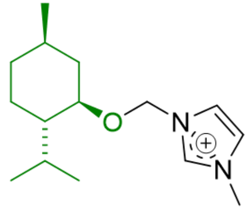

[Men-Im-C_1]$^+$

$V_m = 430.77$ Å^3

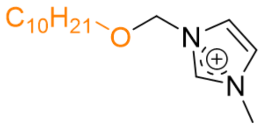

[C_{10}-Im-C_1]$^+$

$V_m = 465.68$ Å^3

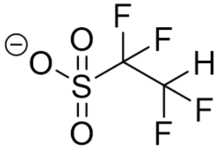

[TFES]$^-$

$V_m = 146.27$ Å^3

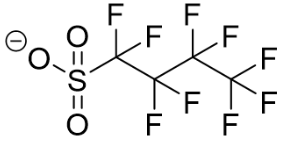

[PFBS]$^-$

$V_m = 214.28$ Å^3

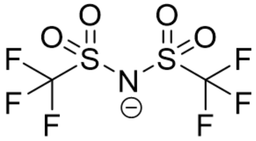

[TFSI]$^-$

$V_m = 208.86$ Å^3

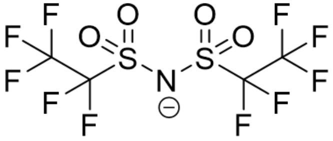

[PFSI]$^-$

$V_m = 275.34$ Å^3

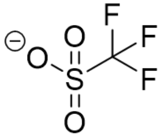

[OTF]$^-$

$V_m = 115.62$ Å^3

Figure S48. Structures and molecular volumes (Å^3) of the cationic and anionic constituents of FILs presented in this work.

PROPERTIES

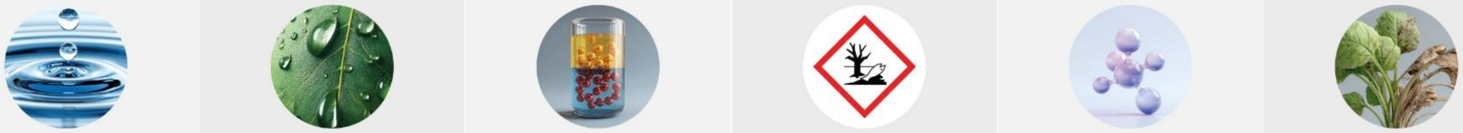

Anion variation: [Men-Im-C$_1$]$^+$ series

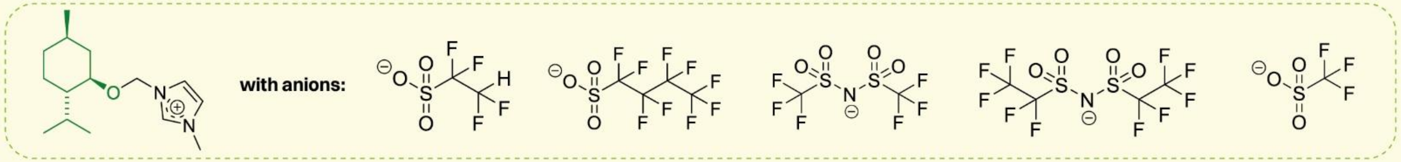

	Water solubility limit [mol · dm⁻³]	Surface tension [mN · m⁻¹]	Log K_{ow} [-]	Log BCF [-]	Molecular volume [Å³]	Phytotoxicity [mg · kg⁻¹ soil DW]
[Men-Im-C$_1$][TFES] (**4a**)	0.011	38.50	0.53	0.06	577.04	299.10
[Men-Im-C$_1$][PFBS] (**4b**)	0.0014	33.70	3.63	2.46	645.05	182.00
[Men-Im-C$_1$][TFSI] (**4c**)	0.0040	50.10	2.49	1.35	639.63	74.67
[Men-Im-C$_1$][PFSI] (**4d**)	0.00019	55.00	4.43	3.23	706.11	107.30
[Men-Im-C$_1$][OTF] (**4e**)	0.012	40.60	1.33	0.38	546.39	131.90

most preferred · **more preferred** · **medium** · **less preferred** · **least preferred**

Figure S49. Heatmap visualisation of structure–property–toxicity relationships for [Men-Im-C$_1$]$^+$ salts (anion-dependent comparisons). A five-level, series-specific preference scale (least preferred → less preferred → medium → more preferred → most preferred) is used (colours are not comparable to Figure 5). Preference was defined after sign orientation of descriptors: higher EC_{50} and higher water solubility are more preferred; lower log K_{OW}, lower log BCF, and lower molecular volume are more preferred; higher surface tension (γ) is treated as more preferred.

References:

(1) *Vogel's Textbook of Practical Organic Chemistry*, New. ed., 5. ed., rev. [Nachdr.].; Furniss, B. S., Vogel, A. I., Eds.; Pearson/Prentice Hall: Harlow, 2009.

(2) Hanwell, M. D.; Curtis, D. E.; Lonie, D. C.; Vandermeersch, T.; Zurek, E.; Hutchison, G. R. Avogadro: An Advanced Semantic Chemical Editor, Visualization, and Analysis Platform. *J Cheminform* **2012**, *4* (1). https://doi.org/10.1186/1758-2946-4-17.

(3) Lu, T.; Chen, F. Multiwfn: A Multifunctional Wavefunction Analyzer. *J Comput Chem* **2012**, *33* (5), 580–592. https://doi.org/10.1002/jcc.22885.

(4) Wileńska, D.; Anusiewicz, I.; Freza, S.; Bobrowski, M.; Laux, E.; Uhl, S.; Keppner, H.; Skurski, P. Predicting the Viscosity and Electrical Conductivity of Ionic Liquids on the Basis of Theoretically Calculated Ionic Volumes. *Molecular Physics* **2015**, *113* (6), 630–639. https://doi.org/10.1080/00268976.2014.964344.

(5) Thormählen, I.; Straub, J.; Grigull, U. Refractive Index of Water and Its Dependence on Wavelength, Temperature, and Density. *Journal of Physical and Chemical Reference Data* **1985**, *14* (4), 933–945. https://doi.org/10.1063/1.555743.

(6) Wohlfarth, Ch. Refractive Index of Water: Data Extract from Landolt-Börnstein III/47: Optical Constants. In *Refractive Indices of Pure Liquids and Binary Liquid Mixtures (Supplement to III/38)*; Lechner, M. D., Ed.; Martienssen, W., Series Ed.; Landolt-Börnstein - Group III Condensed Matter; Springer Berlin Heidelberg: Berlin, Heidelberg, 2008; Vol. 47, pp 37–41. https://doi.org/10.1007/978-3-540-75291-2_4.

(7) Andresová, A.; Bendová, M.; Schwarz, J.; Wagner, Z.; Feder-Kubis, J. Influence of the Alkyl Side Chain Length on the Thermophysical Properties of Chiral Ionic Liquids with a (1*R* ,2*S*5*R*)-(–)-Menthol Substituent and Data Analysis by Means of Mathematical Gnostics. *Journal of Molecular Liquids* **2017**, *242*, 336–348. https://doi.org/10.1016/j.molliq.2017.07.012.

(8) Feder-Kubis, J. NMR Spectroscopy in Studies of New Chiral Ionic Liquids. *Polimery* **2011**, *56* (09), 676–681. https://doi.org/10.14314/polimery.2011.676.

(9) Davidowski, S. K.; Thompson, F.; Huang, W.; Hasani, M.; Amin, S. A.; Angell, C. A.; Yarger, J. L. NMR Characterization of Ionicity and Transport Properties for a Series of Diethylmethylamine Based Protic Ionic Liquids. *J. Phys. Chem. B* **2016**, *120* (18), 4279–4285. https://doi.org/10.1021/acs.jpcb.6b01203.

(10) Cremer, T.; Kolbeck, C.; Lovelock, K. R. J.; Paape, N.; Wölfel, R.; Schulz, P. S.; Wasserscheid, P.; Weber, H.; Thar, J.; Kirchner, B.; Maier, F.; Steinrück, H. Towards a Molecular Understanding of Cation–Anion Interactions—Probing the Electronic Structure of Imidazolium Ionic Liquids by NMR Spectroscopy, X-ray Photoelectron Spectroscopy and Theoretical Calculations. *Chemistry A European J* **2010**, *16* (30), 9018–9033. https://doi.org/10.1002/chem.201001032.

(11) Bica, K.; Deetlefs, M.; Schröder, C.; Seddon, K. R. Polarisabilities of Alkylimidazolium Ionic Liquids. *Phys. Chem. Chem. Phys.* **2013**, *15* (8), 2703. https://doi.org/10.1039/c3cp43867h.

(12) Gano, M.; Janus, E.; Cybulska, K. Effect of Terpene Alcohol Fragment and Type of Anion in the Pyrrolidinium Salts on the Physicochemical Properties and Antibacterial Activity. *Journal of Molecular Liquids* **2024**, *407*, 125254. https://doi.org/10.1016/j.molliq.2024.125254.

(13) Pike, S. J.; Hutchinson, J. J.; Hunter, C. A. H-Bond Acceptor Parameters for Anions. *J. Am. Chem. Soc.* **2017**, *139* (19), 6700–6706. https://doi.org/10.1021/jacs.7b02008.

(14) Stark, A.; Brehm, M.; Brüssel, M.; Lehmann, S. B. C.; Pensado, A. S.; Schöppke, M.; Kirchner, B. A Theoretical and Experimental Chemist's Joint View on Hydrogen Bonding in Ionic Liquids and Their Binary Mixtures. In *Electronic Effects in Organic Chemistry*; Kirchner, B., Ed.; Topics in Current Chemistry; Springer Berlin Heidelberg: Berlin, Heidelberg, 2013; Vol. 351, pp 149–187. https://doi.org/10.1007/128_2013_485.

(15) Larriba, M.; García, S.; García, J.; Torrecilla, J. S.; Rodríguez, F. Thermophysical Properties of 1-Ethyl-3-Methylimidazolium 1,1,2,2-Tetrafluoroethanesulfonate and 1-Ethyl-3-Methylimidazolium Ethylsulfate Ionic Liquids as a Function of Temperature. *J. Chem. Eng. Data* **2011**, *56* (9), 3589–3597. https://doi.org/10.1021/je2004462.

(16) Tariq, M.; Forte, P. A. S.; Gomes, M. F. C.; Lopes, J. N. C.; Rebelo, L. P. N. Densities and Refractive Indices of Imidazolium- and Phosphonium-Based Ionic Liquids: Effect of

Temperature, Alkyl Chain Length, and Anion. *The Journal of Chemical Thermodynamics* **2009**, *41* (6), 790–798. https://doi.org/10.1016/j.jct.2009.01.012.

(17) Rahman, M. M.; Jahan, M. S.; Islam, Md. M.; Susan, Md. A. B. H. Dissolution of Cellulose in Imidazolium-Based Double Salt Ionic Liquids. *International Journal of Biological Macromolecules* **2024**, *267*, 131331. https://doi.org/10.1016/j.ijbiomac.2024.131331.

(18) Bastos, J. C.; Carvalho, S. F.; Welton, T.; Canongia Lopes, J. N.; Rebelo, L. P. N.; Shimizu, K.; Araújo, J. M. M.; Pereiro, A. B. Design of Task-Specific Fluorinated Ionic Liquids: Nanosegregation *versus* Hydrogen-Bonding Ability in Aqueous Solutions. *Chem. Commun.* **2018**, *54* (28), 3524–3527. https://doi.org/10.1039/C8CC00361K.

(19) Diejomaoh Abafe, O. T.; Azim, M. M.; Martincigh, B. S.; Stark, A. Cation-Fluorinated Ionic Liquids: Synthesis, Physicochemical Properties and Comparison with Non-Fluorinated Analogues. *Journal of Molecular Liquids* **2022**, *349*, 118104. https://doi.org/10.1016/j.molliq.2021.118104.

(20) Pernak, J.; Feder-Kubis, J.; Cieniecka-Rosłonkiewicz, A.; Fischmeister, C.; Griffin, S. T.; Rogers, R. D. Synthesis and Properties of Chiral Imidazolium Ionic Liquids with a (1*R*,2*S*,5*R*)-(−)-Menthoxymethyl Substituent. *New J. Chem.* **2007**, *31* (6), 879–892. https://doi.org/10.1039/B616215K.

(21) Wojcieszak, M.; Syguda, A.; Zięba, S.; Mizera, A.; Łapiński, A.; Materna, K. Effect of Surface-Active Ionic Liquids Structure on Their Synthesis, Physicochemical Properties, and Potential Use as Crop Protection Agents. *Journal of Molecular Liquids* **2023**, *383*, 122050. https://doi.org/10.1016/j.molliq.2023.122050.
